# Supplementary material for: Policy options for surgical mentoring: Lessons from Zambia based on stakeholder consultation and systems science
Source: PLoS One. 2021 Sep 29;16(9):e0257597. doi: 10.1371/journal.pone.0257597 (PMC8480833; doi:10.1371/journal.pone.0257597)
Supplement: S1 Data — (ZIP) [file pone.0257597.s001.zip › S1_Broekhuizen.PDF]

Stimulating `HRM` until i=100

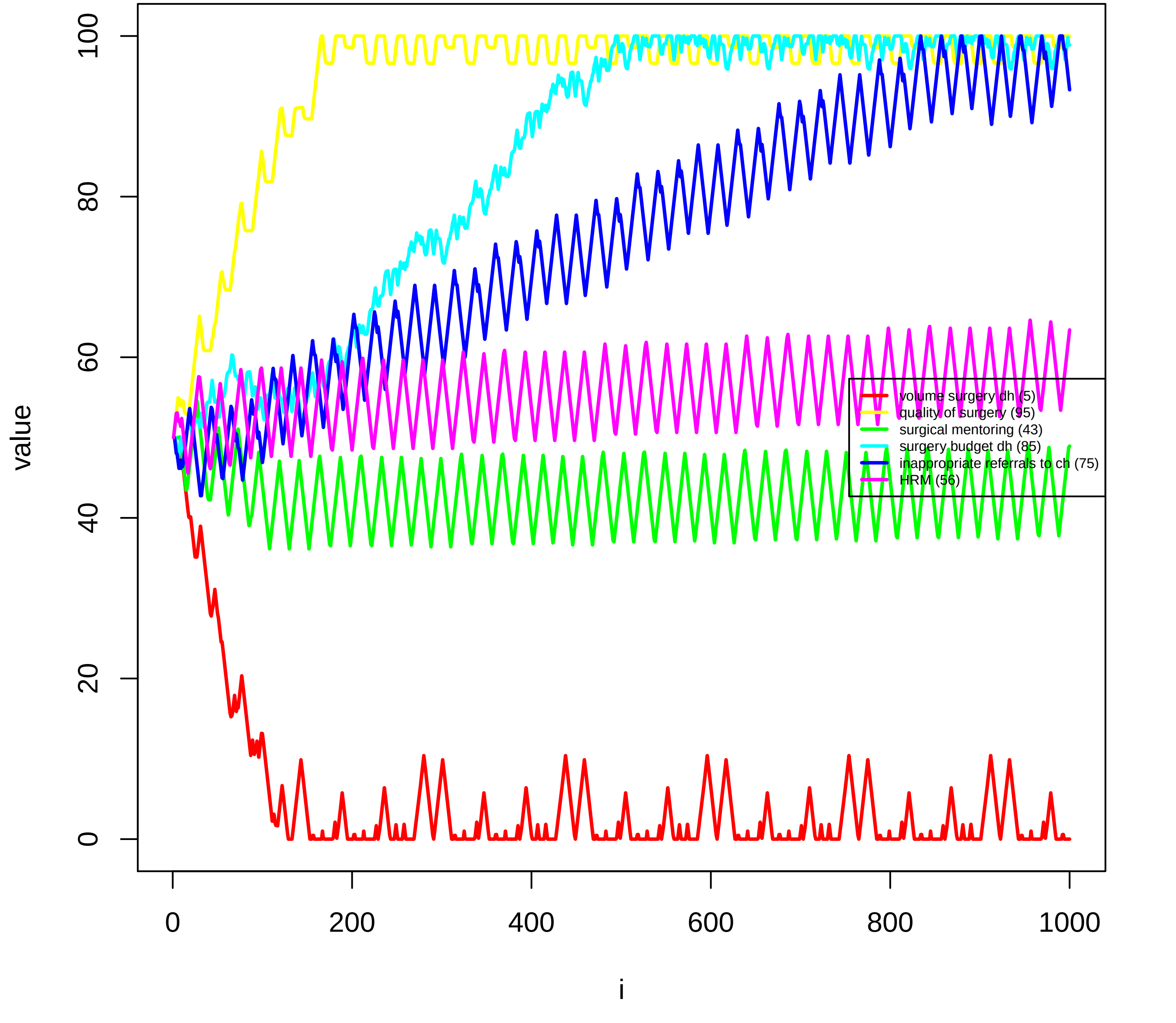

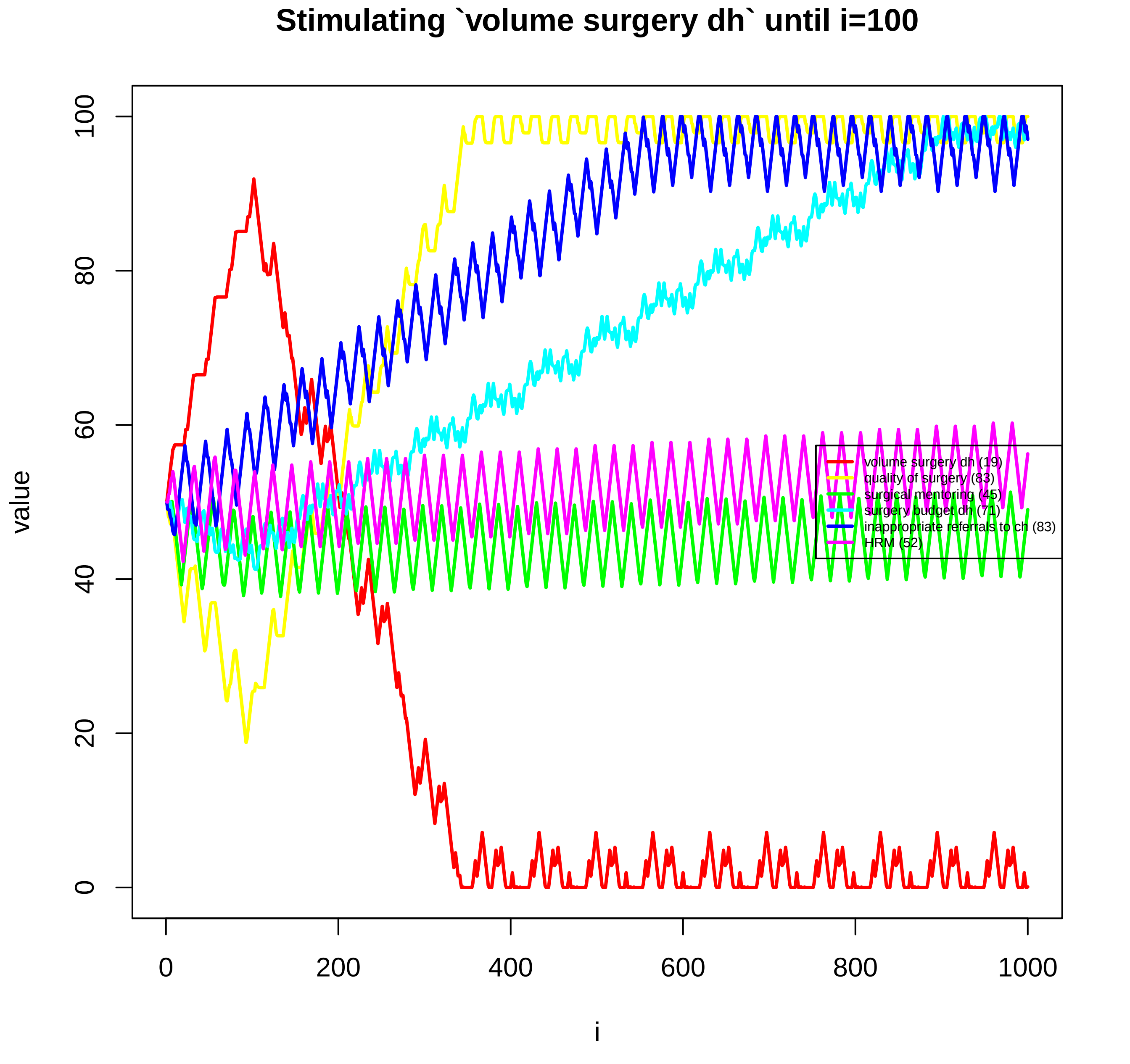

Stimulating `volume surgery dh` until i=100
value
i
0
20
40
60
80
100
0
200
400
600
800
1000
volume surgery dh (19)
quality of surgery (83)
surgical mentoring (45)
surgery budget dh (71)
inappropriate referrals to ch (83)
HRM (52)

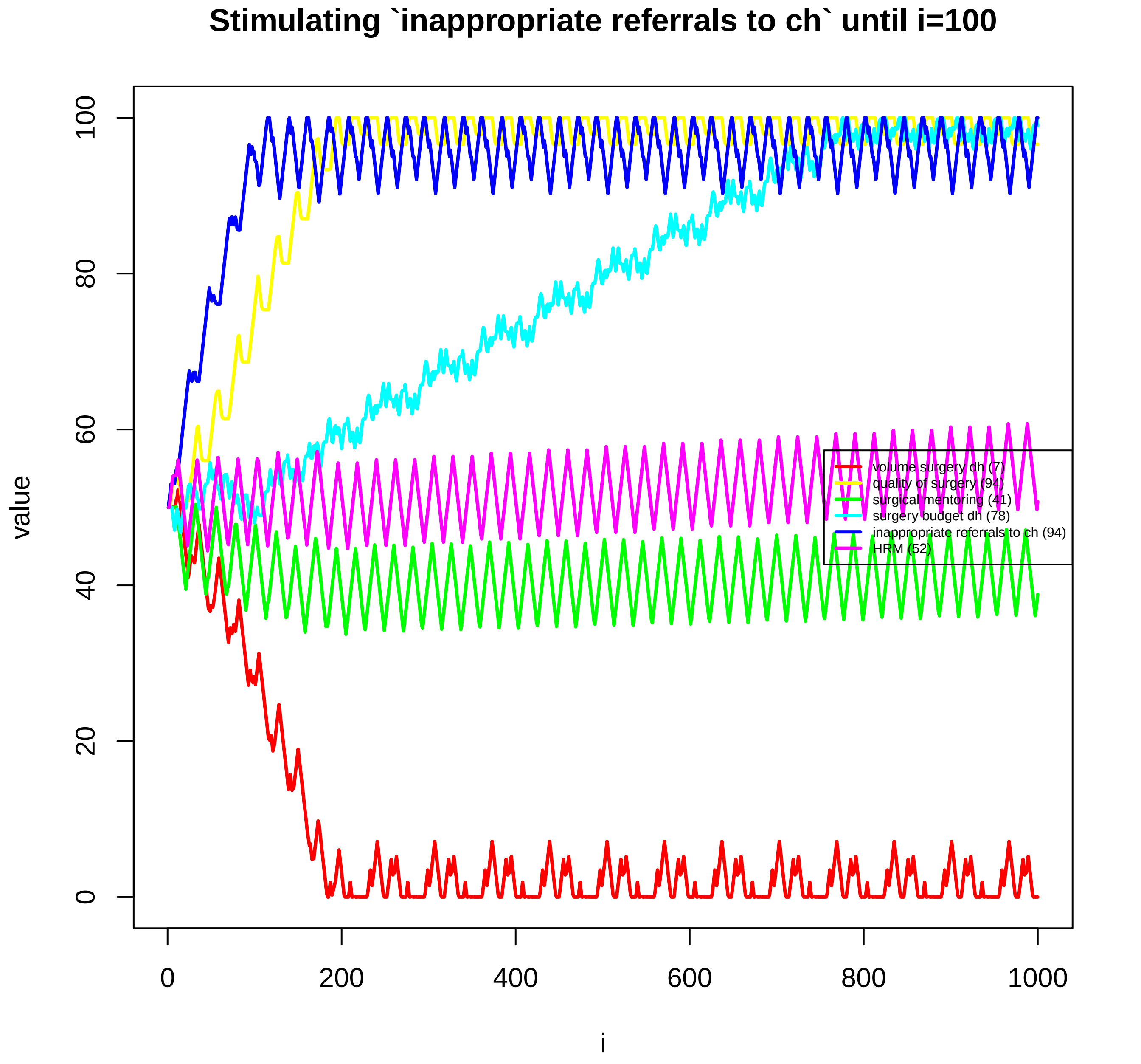
Stimulating `inappropriate referrals to ch` until i=100
value
i
0
20
40
60
80
100
0
200
400
600
800
1000
volume surgery dh (7)
quality of surgery (94)
surgical mentoring (41)
surgery budget dh (78)
inappropriate referrals to ch (94)
HRM (52)

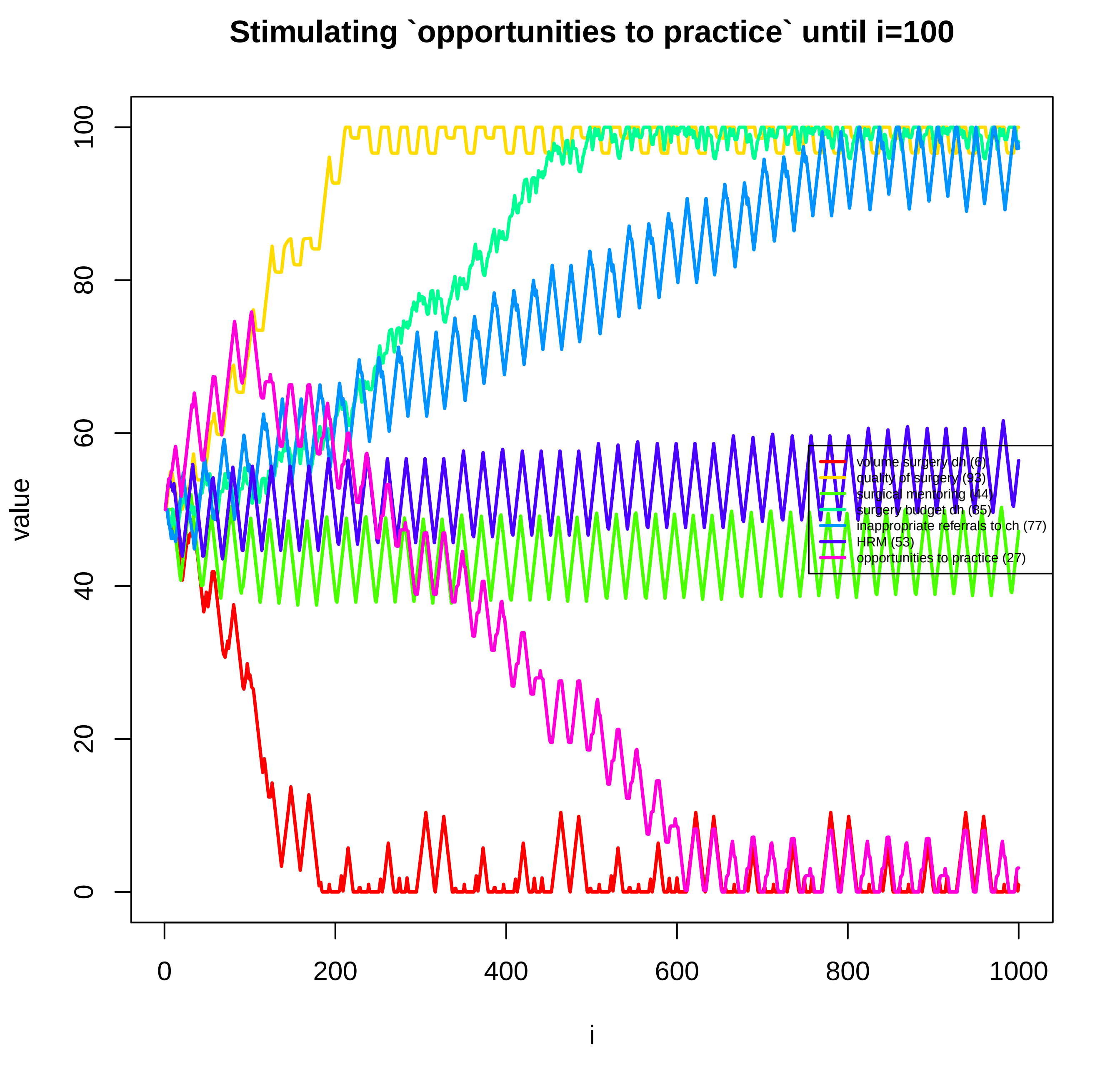
Stimulating `opportunities to practice` until i=100
value
i
0
20
40
60
80
100
0
200
400
600
800
1000
volume surgery dh (6)
quality of surgery (93)
surgical mentoring (44)
surgery budget dh (85)
inappropriate referrals to ch (77)
HRM (53)
opportunities to practice (27)

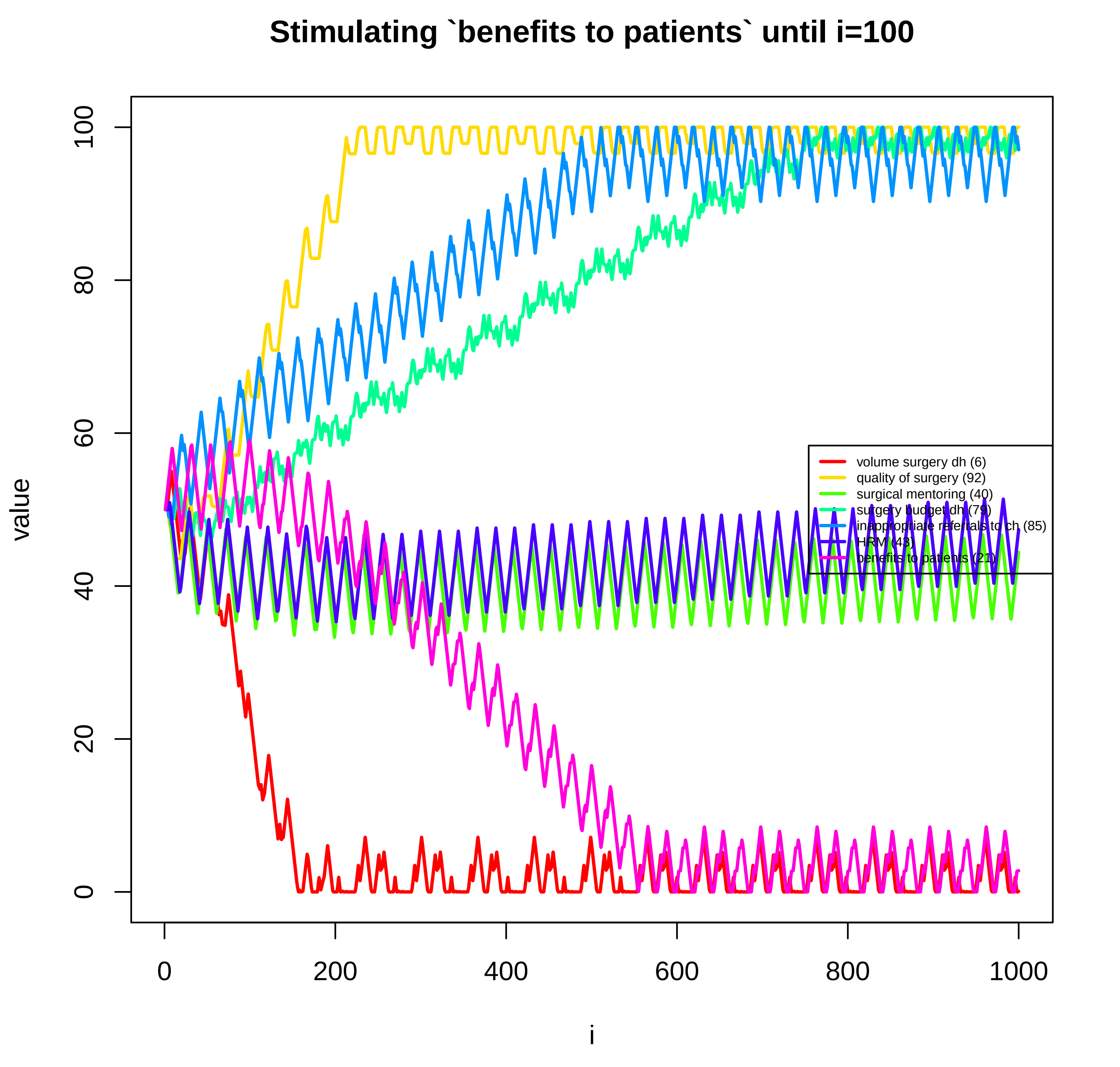

Stimulating `benefits to patients` until i=100
value
i
0
20
40
60
80
100
0
200
400
600
800
1000
volume surgery dh (6)
quality of surgery (92)
surgical mentoring (40)
surgery budget dh (79)
inappropriate referrals to ch (85)
HRM (43)
benefits to patients (21)

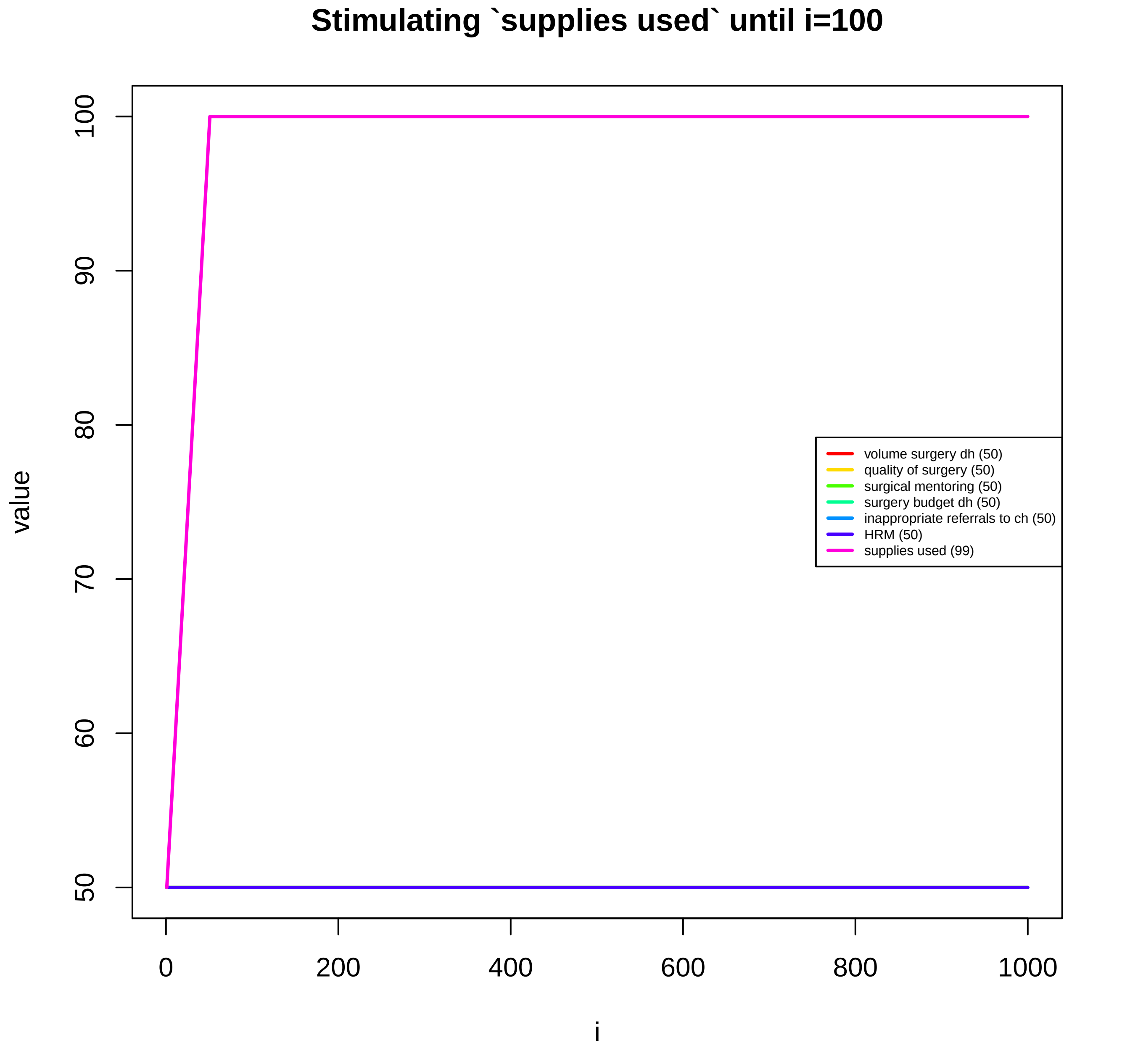
Stimulating `supplies used` until i=100
value
100
90
80
70
60
50
0
200
400
600
800
1000
i
volume surgery dh (50)
quality of surgery (50)
surgical mentoring (50)
surgery budget dh (50)
inappropriate referrals to ch (50)
HRM (50)
supplies used (99)

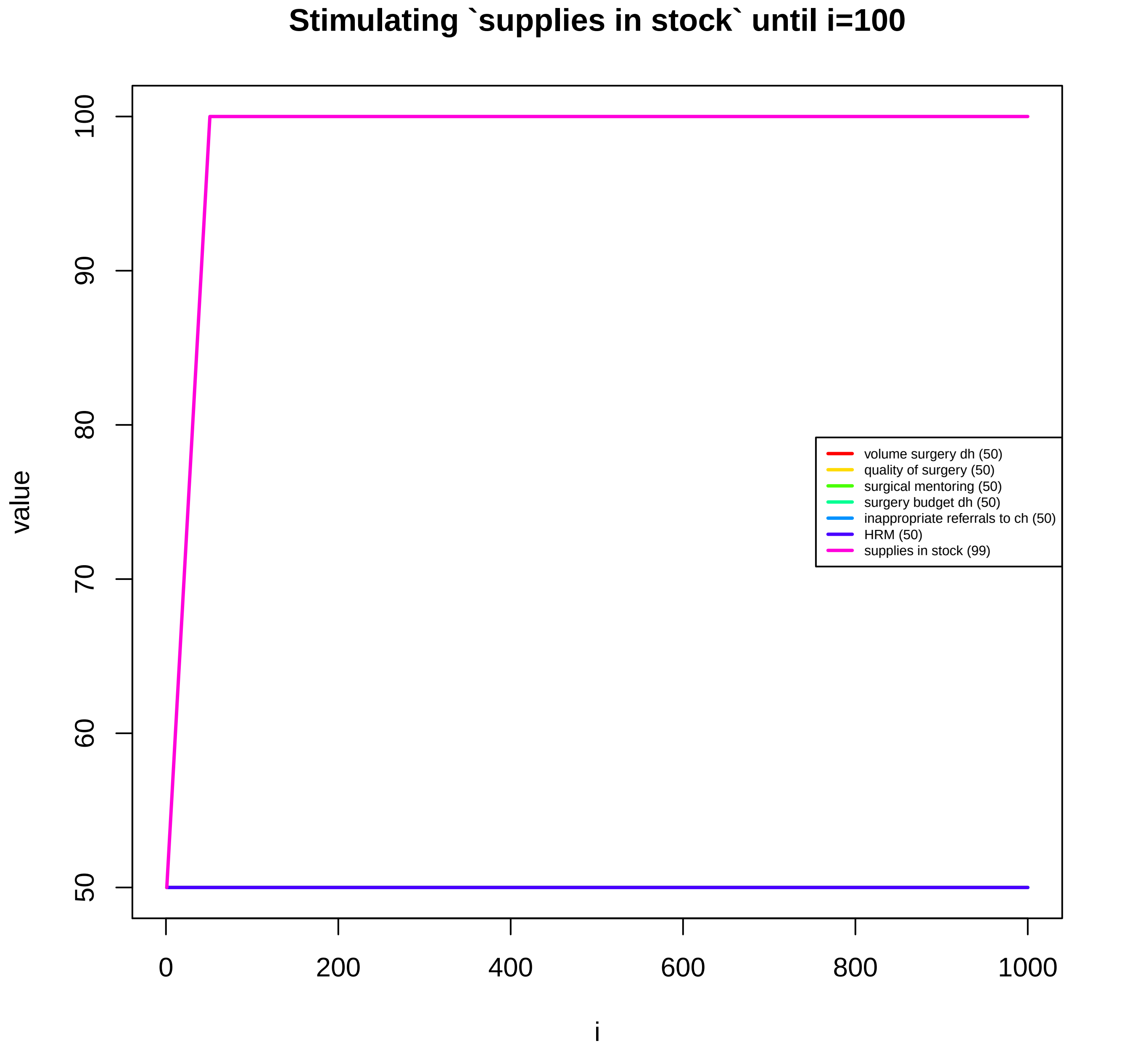
Stimulating `supplies in stock` until i=100
value
i
100
90
80
70
60
50
0
200
400
600
800
1000
volume surgery dh (50)
quality of surgery (50)
surgical mentoring (50)
surgery budget dh (50)
inappropriate referrals to ch (50)
HRM (50)
supplies in stock (99)

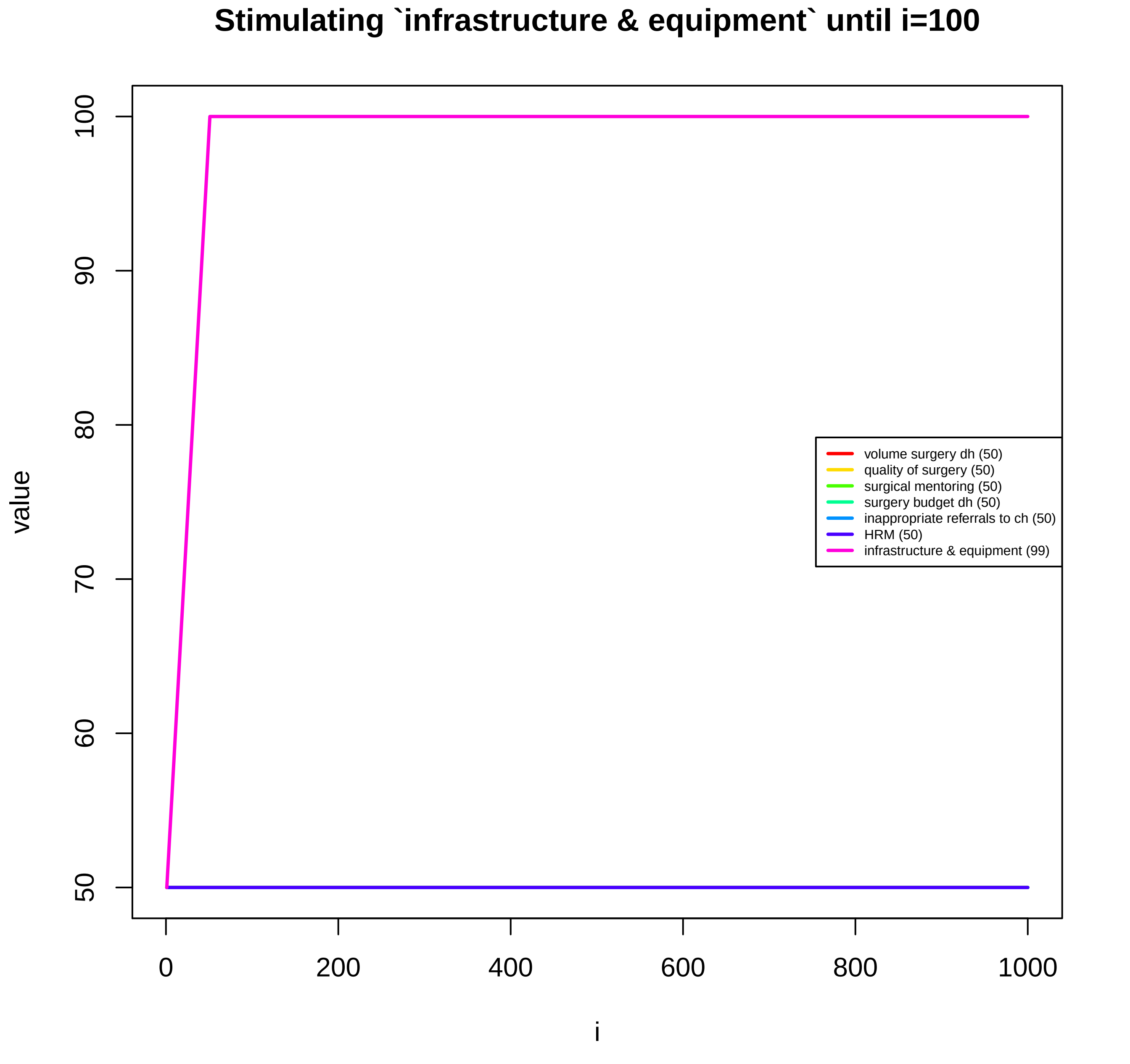
Stimulating `infrastructure & equipment` until i=100
value
i
volume surgery dh (50)
quality of surgery (50)
surgical mentoring (50)
surgery budget dh (50)
inappropriate referrals to ch (50)
HRM (50)
infrastructure & equipment (99)

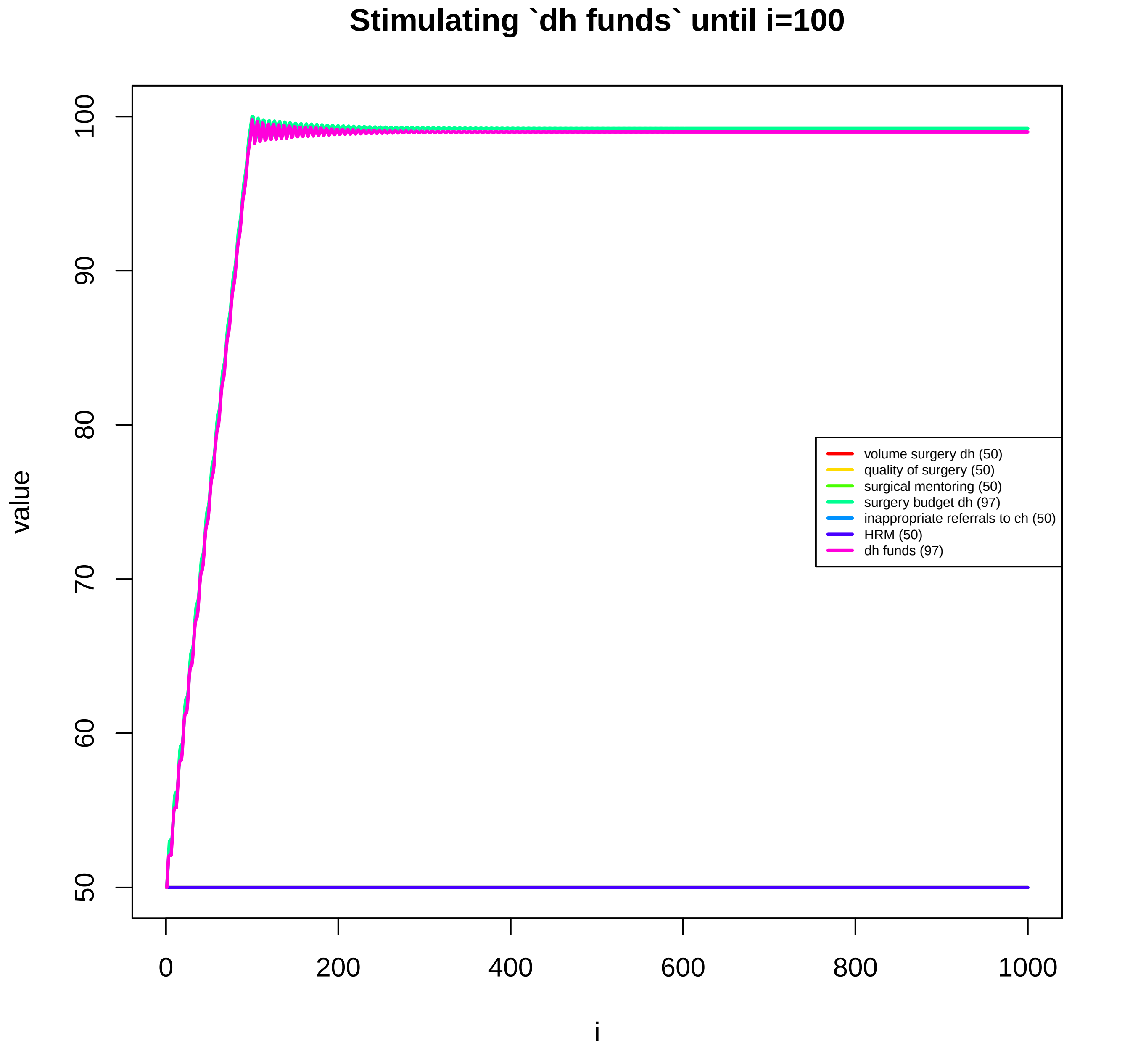
Stimulating `dh funds` until i=100
value
i
0
200
400
600
800
1000
50
60
70
80
90
100
volume surgery dh (50)
quality of surgery (50)
surgical mentoring (50)
surgery budget dh (97)
inappropriate referrals to ch (50)
HRM (50)
dh funds (97)

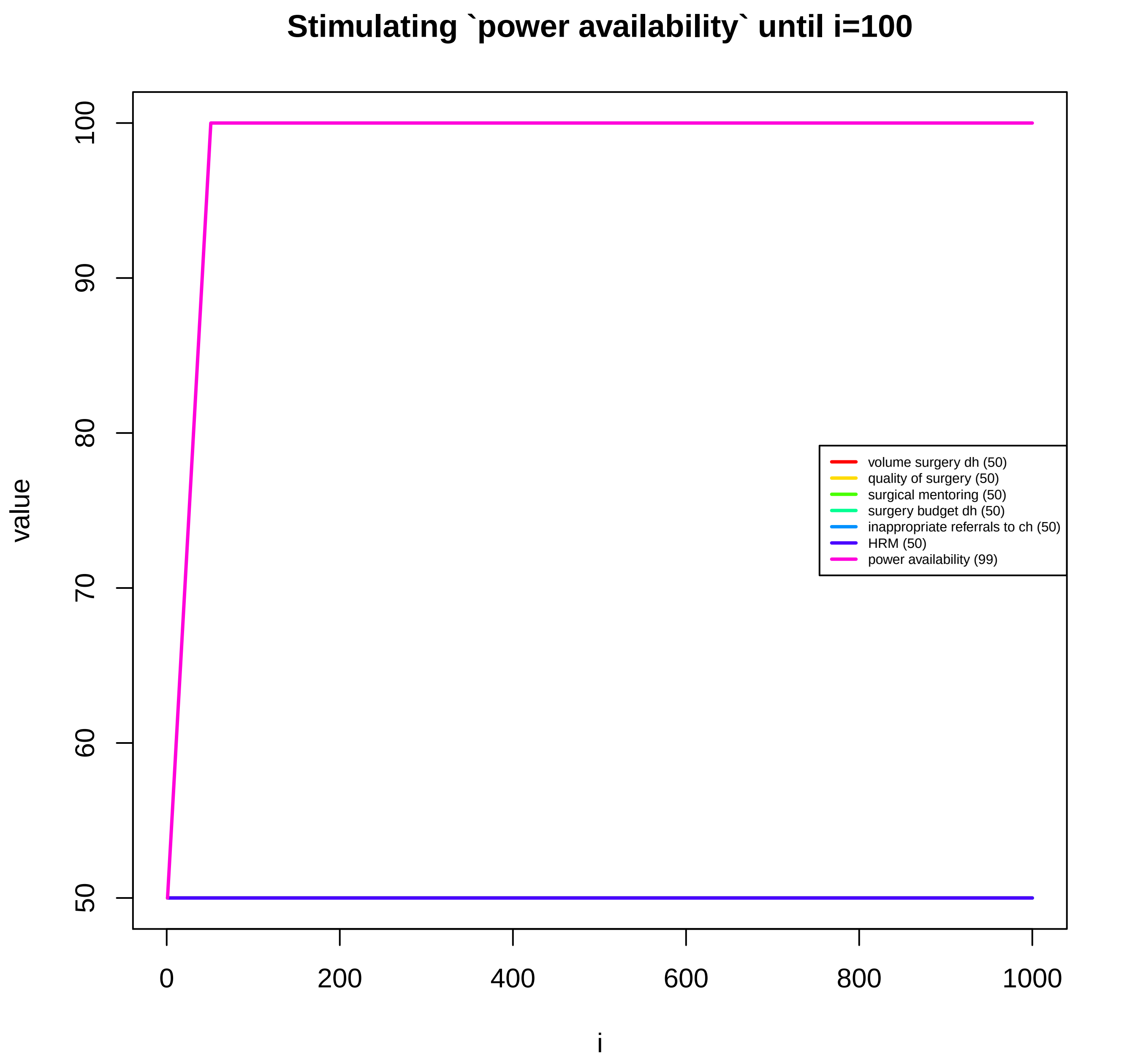
Stimulating `power availability` until i=100
value
i
0
200
400
600
800
1000
50
60
70
80
90
100
volume surgery dh (50)
quality of surgery (50)
surgical mentoring (50)
surgery budget dh (50)
inappropriate referrals to ch (50)
HRM (50)
power availability (99)

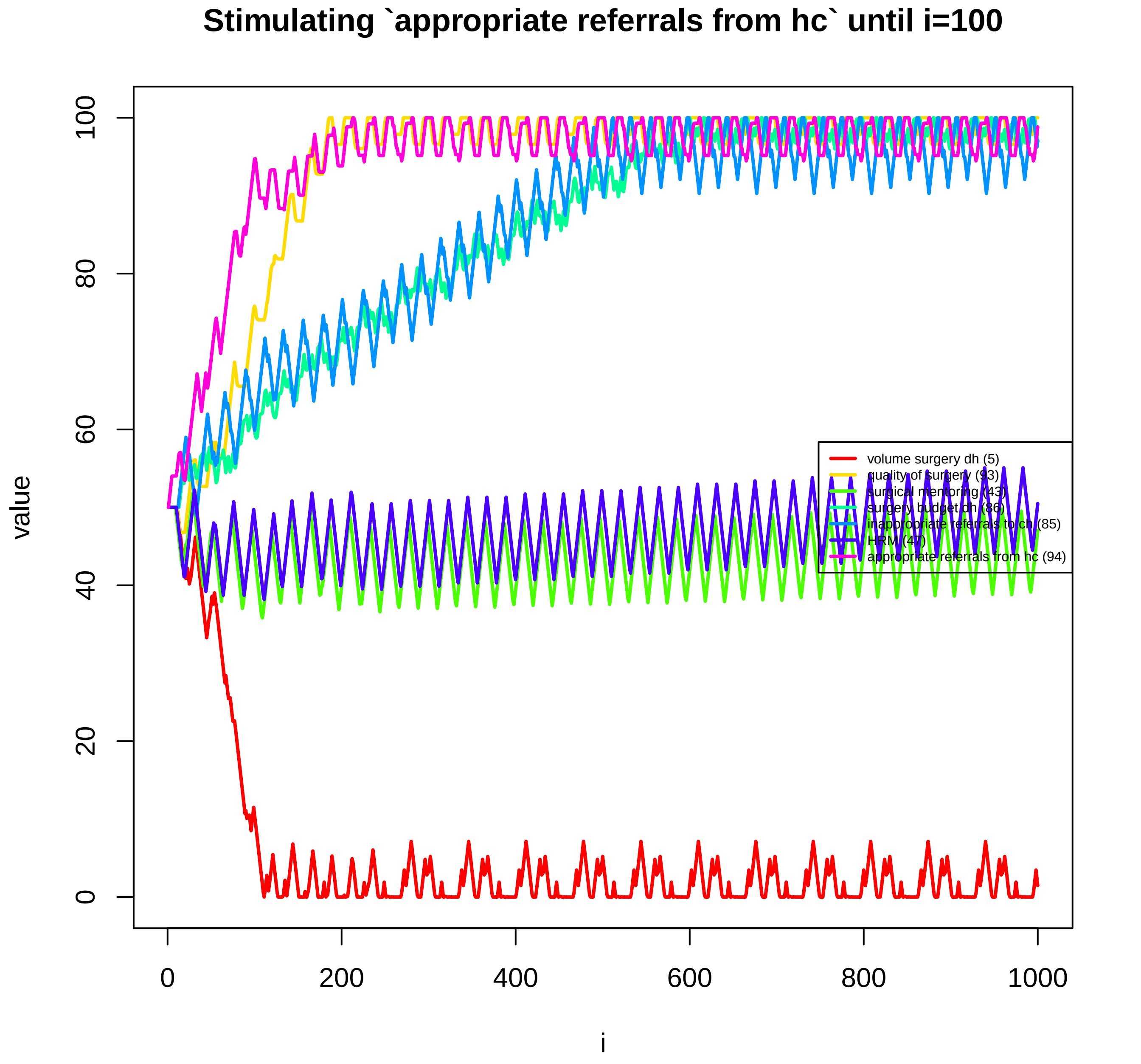
Stimulating `appropriate referrals from hc` until i=100
value
i
0
20
40
60
80
100
0
200
400
600
800
1000
volume surgery dh (5)
quality of surgery (93)
surgical mentoring (43)
surgery budget dh (86)
inappropriate referrals to ch (85)
HRM (47)
appropriate referrals from hc (94)

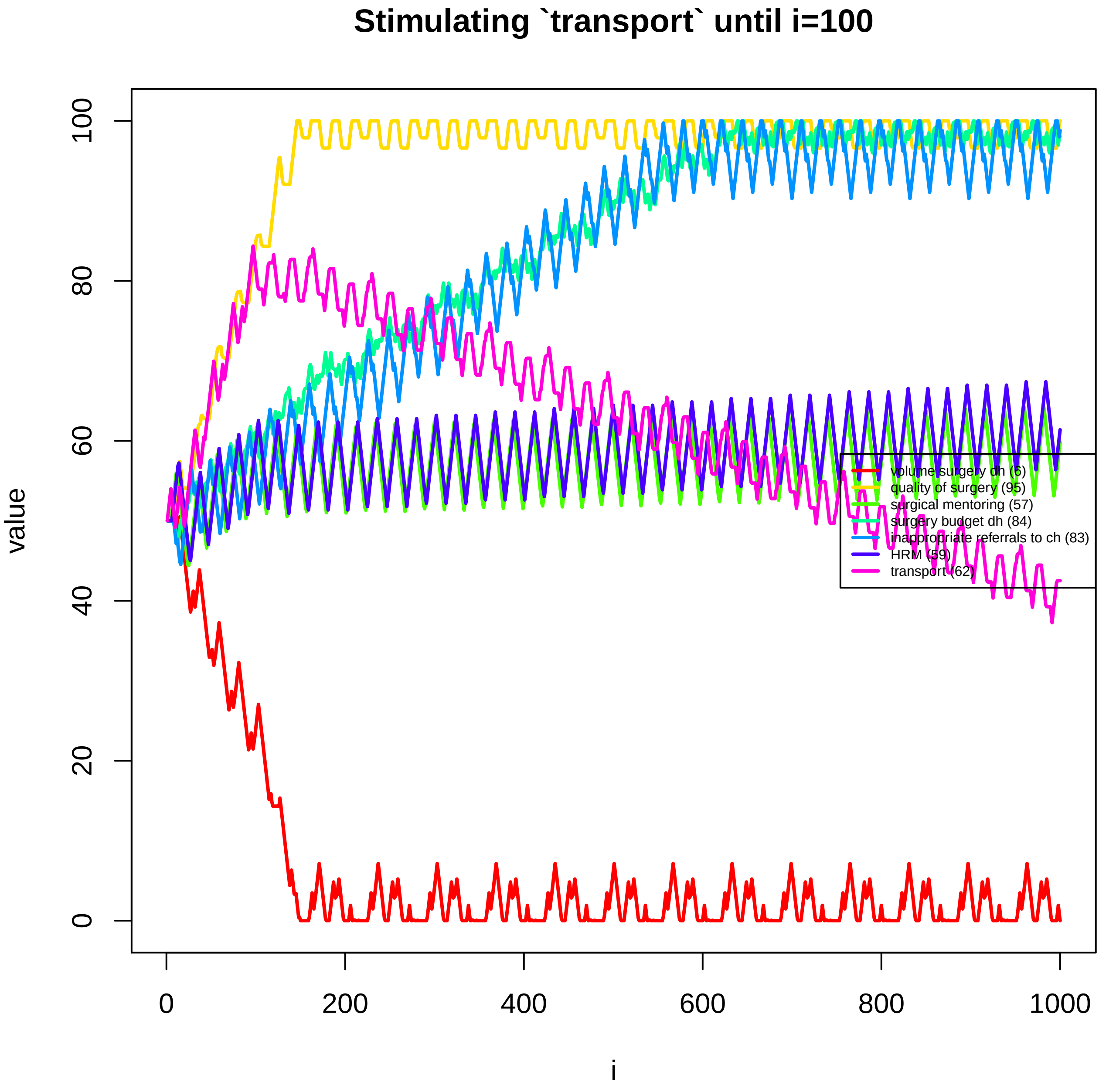
Stimulating `transport` until i=100
value
i
0
20
40
60
80
100
0
200
400
600
800
1000
volume surgery dh (6)
quality of surgery (95)
surgical mentoring (57)
surgery budget dh (84)
inappropriate referrals to ch (83)
HRM (59)
transport (62)

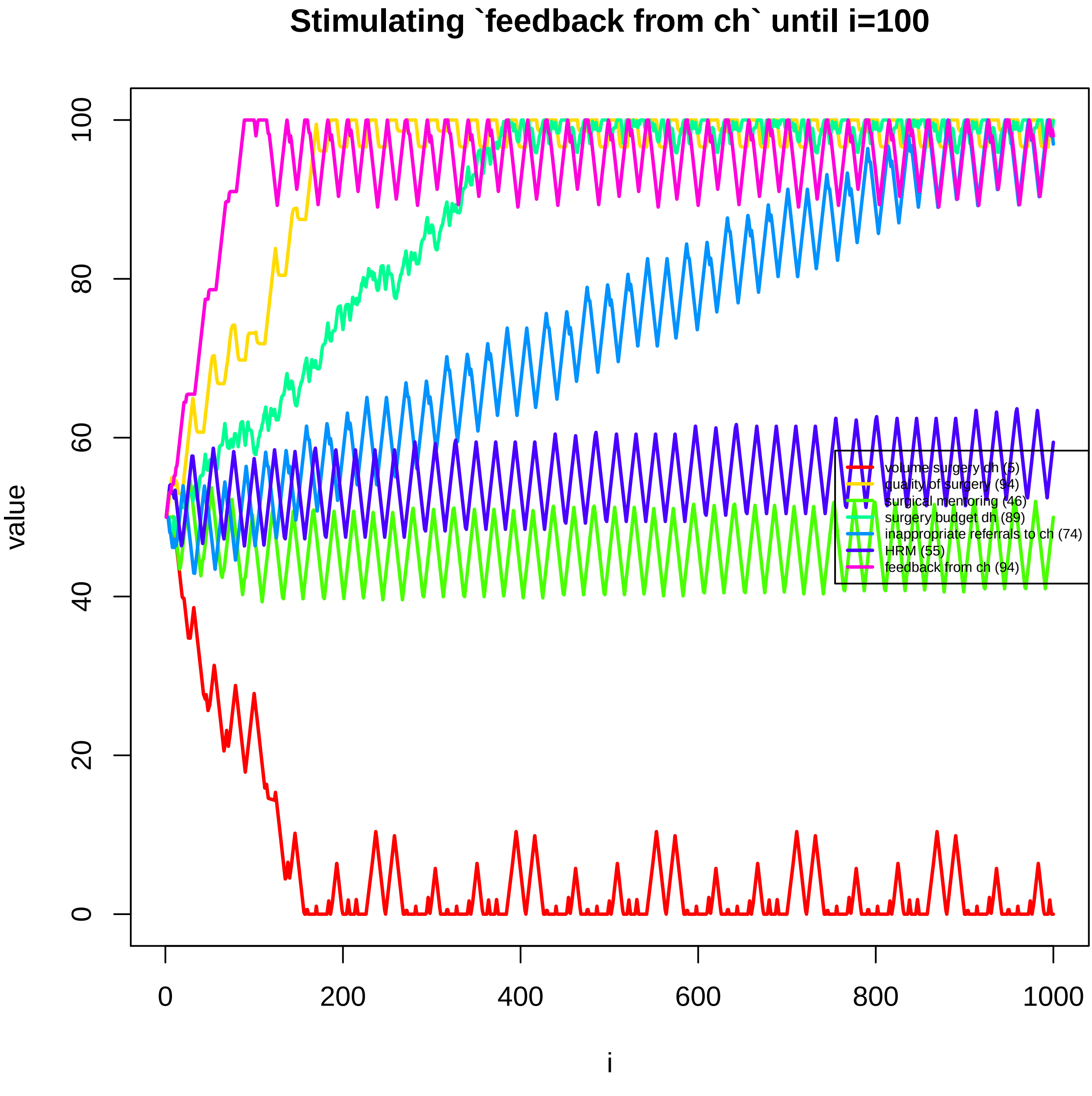
Stimulating `feedback from ch` until i=100
value
i
0
20
40
60
80
100
0
200
400
600
800
1000
volume surgery dh (5)
quality of surgery (94)
surgical mentoring (46)
surgery budget dh (89)
inappropriate referrals to ch (74)
HRM (55)
feedback from ch (94)

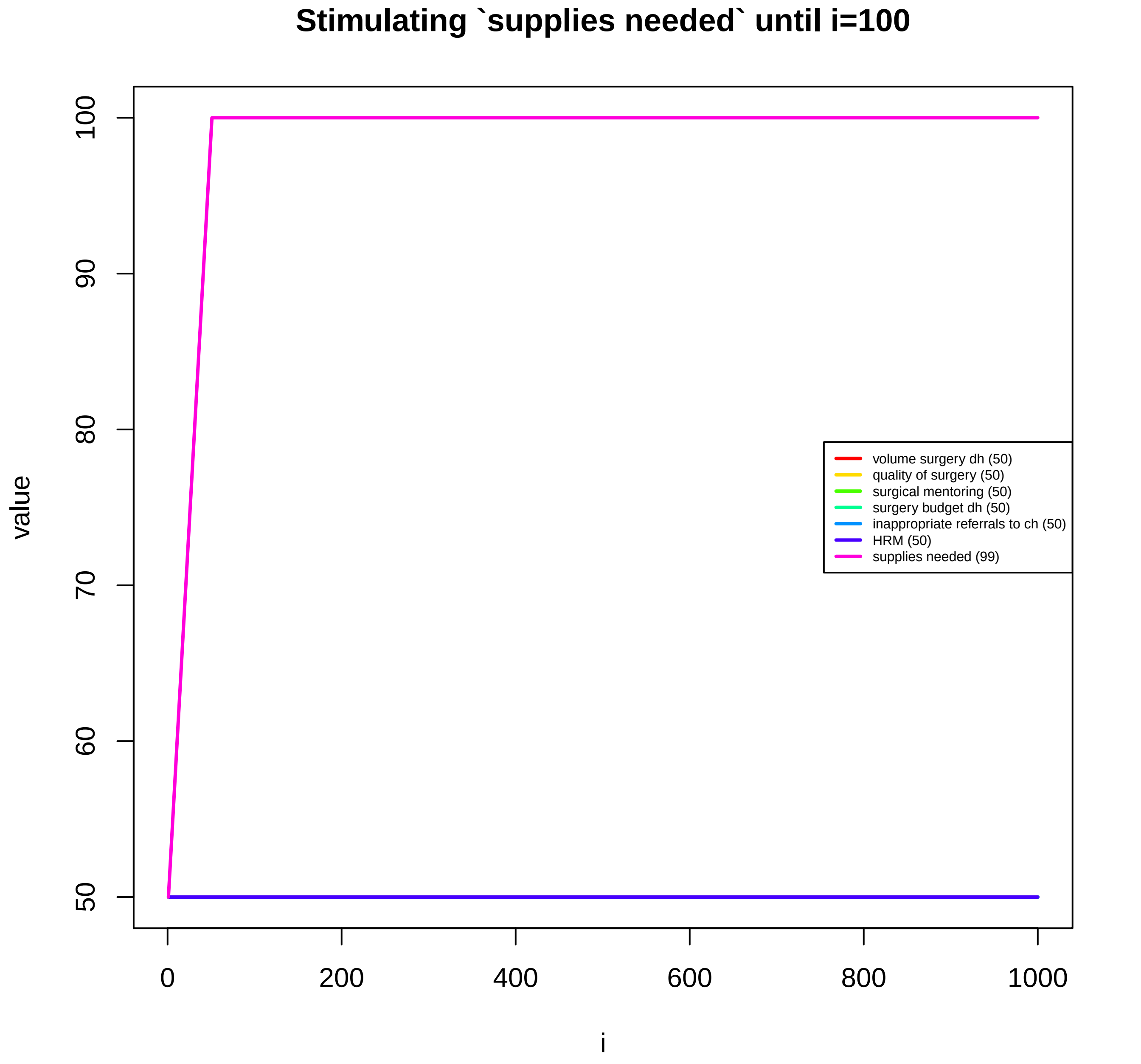
Stimulating `supplies needed` until i=100
value
i
100
90
80
70
60
50
0
200
400
600
800
1000
volume surgery dh (50)
quality of surgery (50)
surgical mentoring (50)
surgery budget dh (50)
inappropriate referrals to ch (50)
HRM (50)
supplies needed (99)

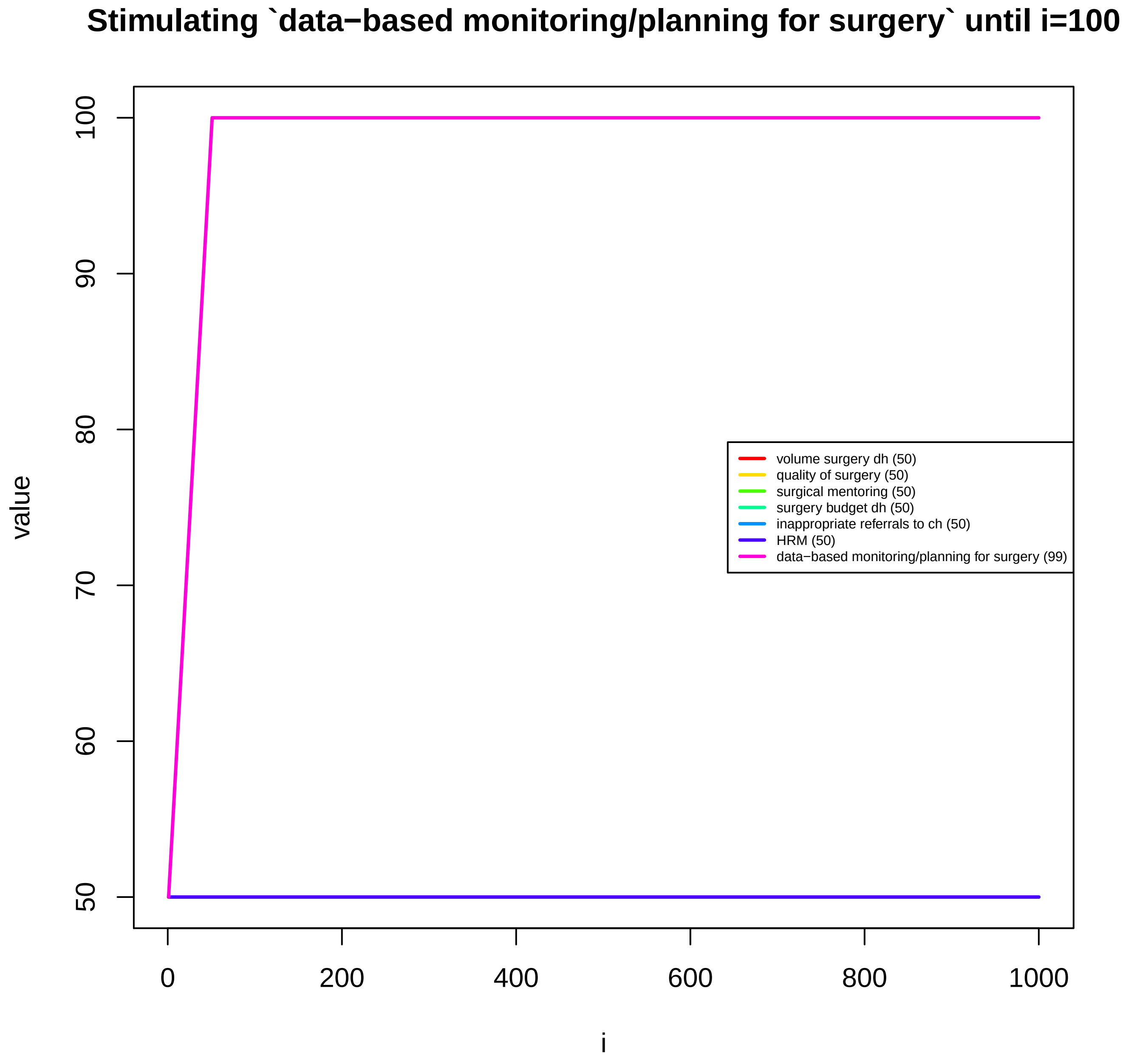

Stimulating `data−based monitoring/planning for surgery` until i=100
value
i
100
90
80
70
60
50
0
200
400
600
800
1000
volume surgery dh (50)
quality of surgery (50)
surgical mentoring (50)
surgery budget dh (50)
inappropriate referrals to ch (50)
HRM (50)
data−based monitoring/planning for surgery (99)

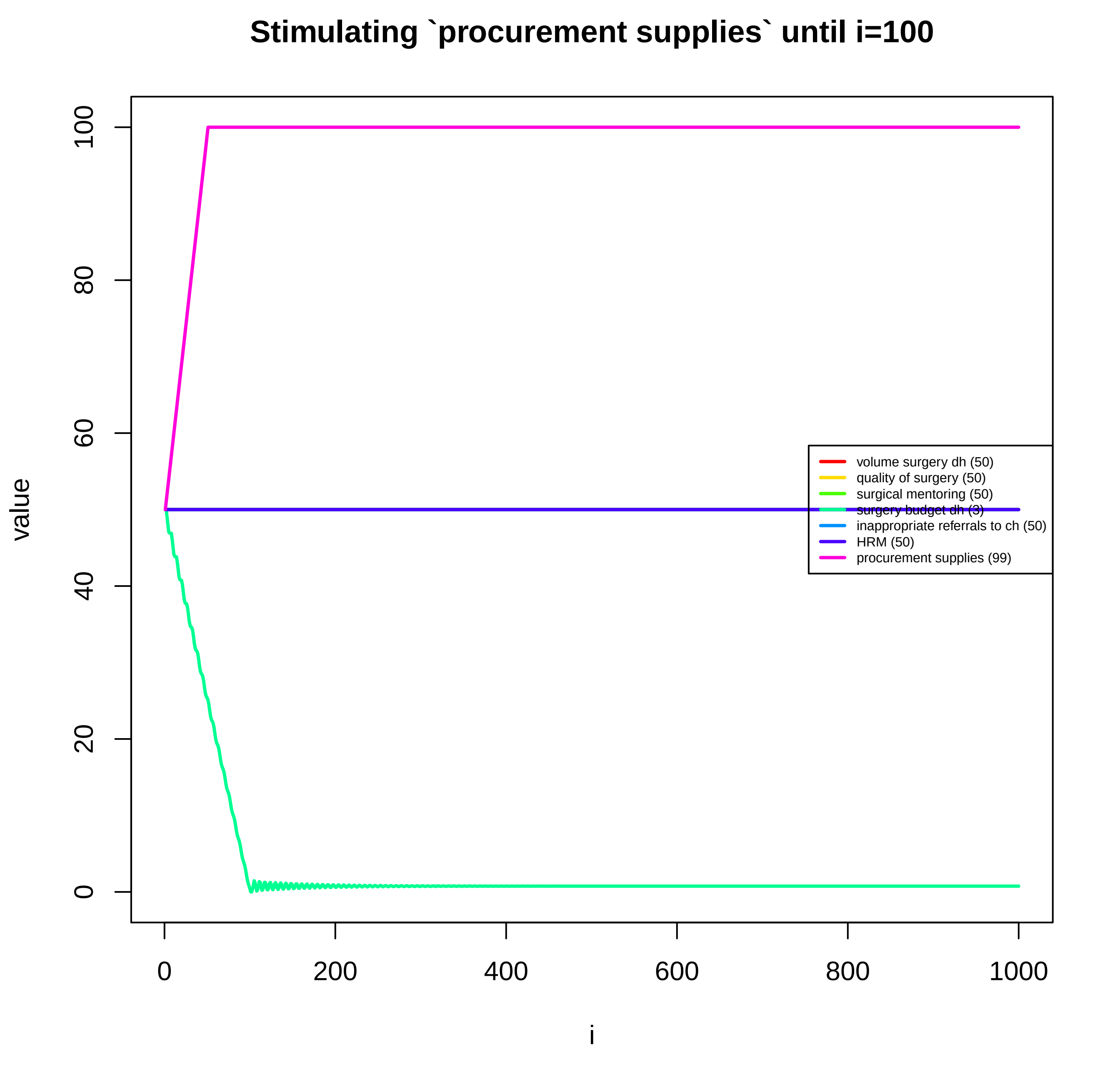

Stimulating `procurement supplies` until i=100
value
i
0
20
40
60
80
100
0
200
400
600
800
1000
volume surgery dh (50)
quality of surgery (50)
surgical mentoring (50)
surgery budget dh (3)
inappropriate referrals to ch (50)
HRM (50)
procurement supplies (99)

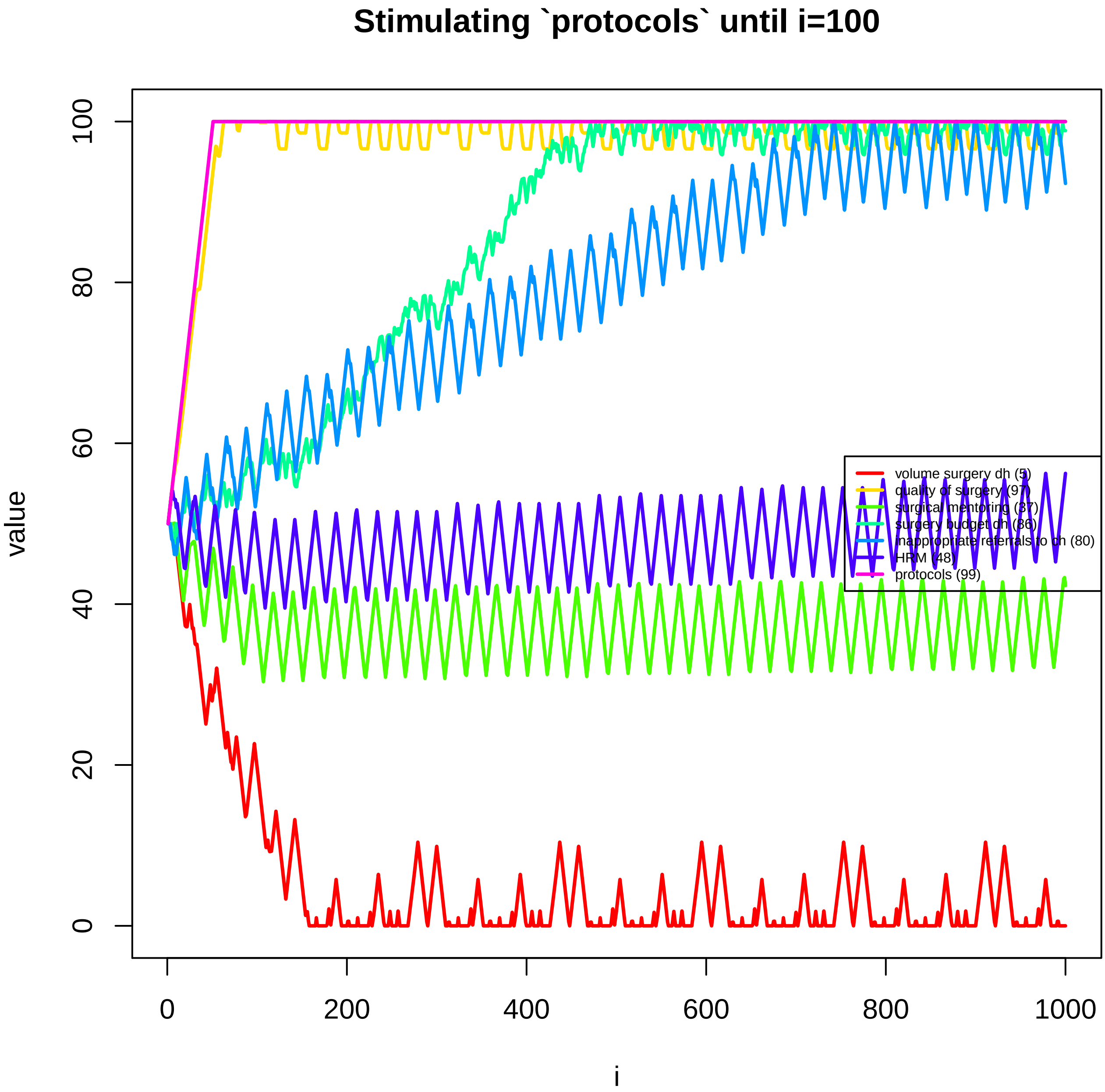
Stimulating `protocols` until i=100
value
i
0
20
40
60
80
100
0
200
400
600
800
1000
volume surgery dh (5)
quality of surgery (97)
surgical mentoring (37)
surgery budget dh (86)
inappropriate referrals to ch (80)
HRM (48)
protocols (99)

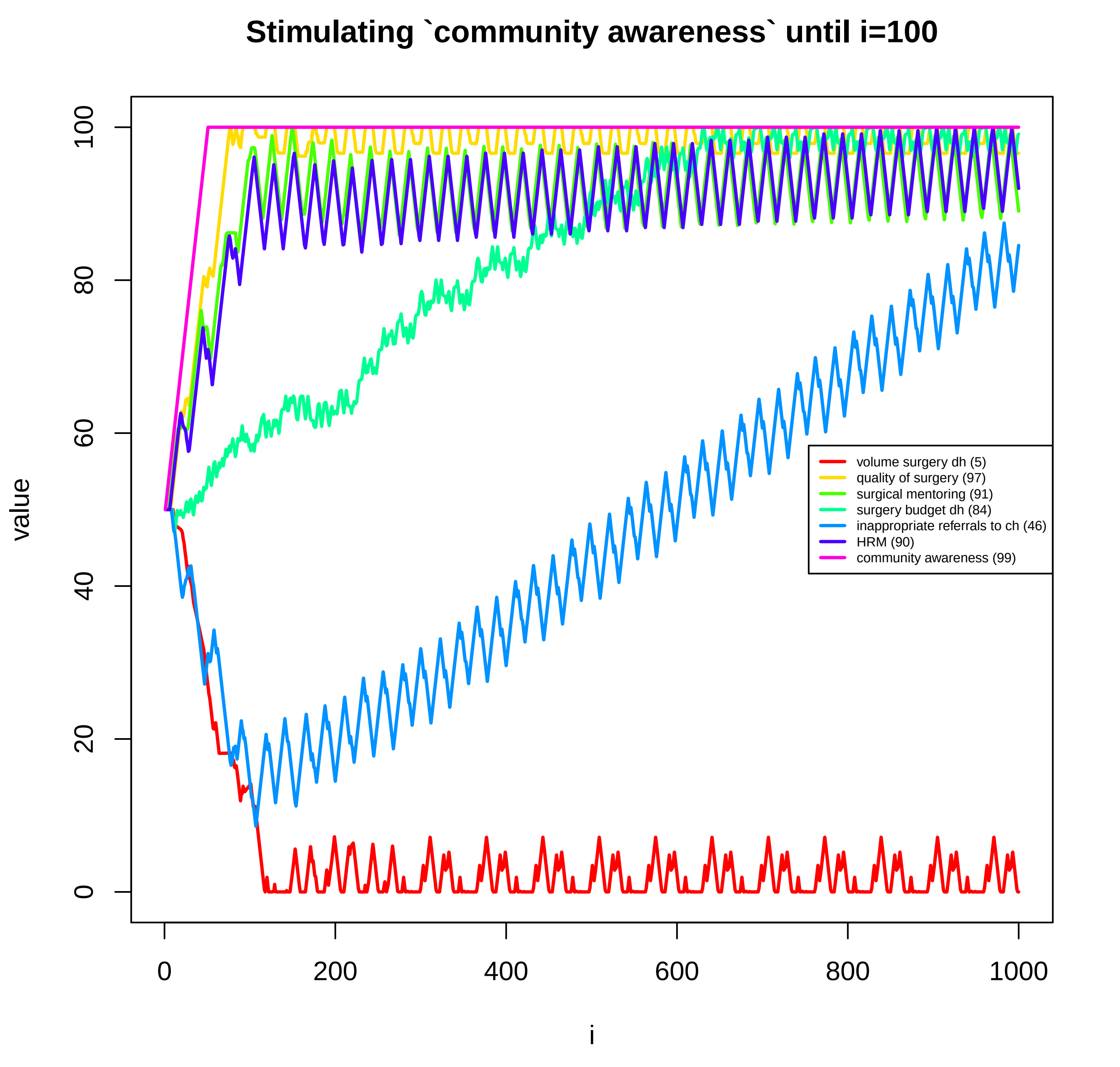
Stimulating `community awareness` until i=100
value
i
0
20
40
60
80
100
0
200
400
600
800
1000
volume surgery dh (5)
quality of surgery (97)
surgical mentoring (91)
surgery budget dh (84)
inappropriate referrals to ch (46)
HRM (90)
community awareness (99)

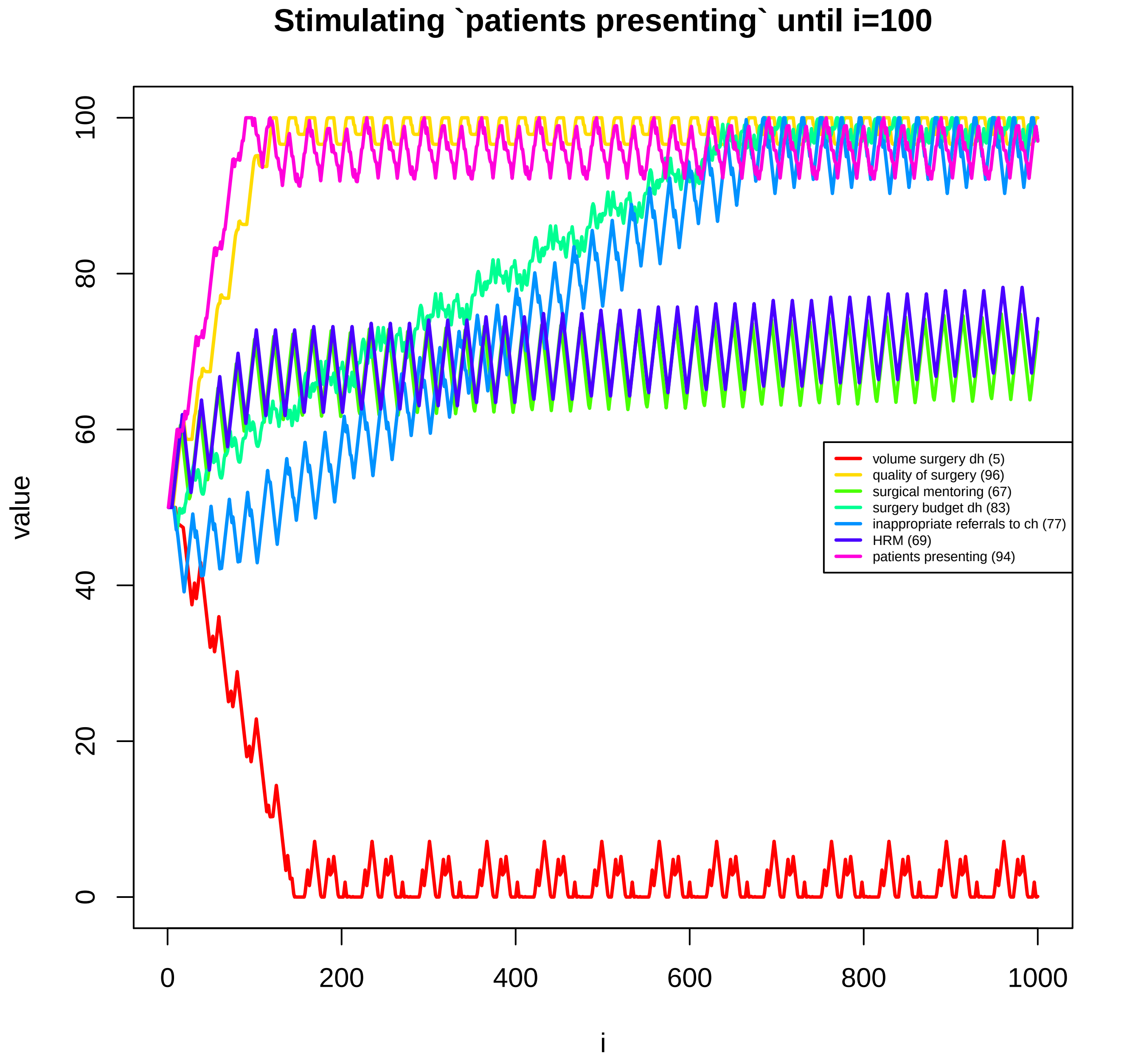
Stimulating `patients presenting` until i=100
value
i
0
20
40
60
80
100
0
200
400
600
800
1000
volume surgery dh (5)
quality of surgery (96)
surgical mentoring (67)
surgery budget dh (83)
inappropriate referrals to ch (77)
HRM (69)
patients presenting (94)

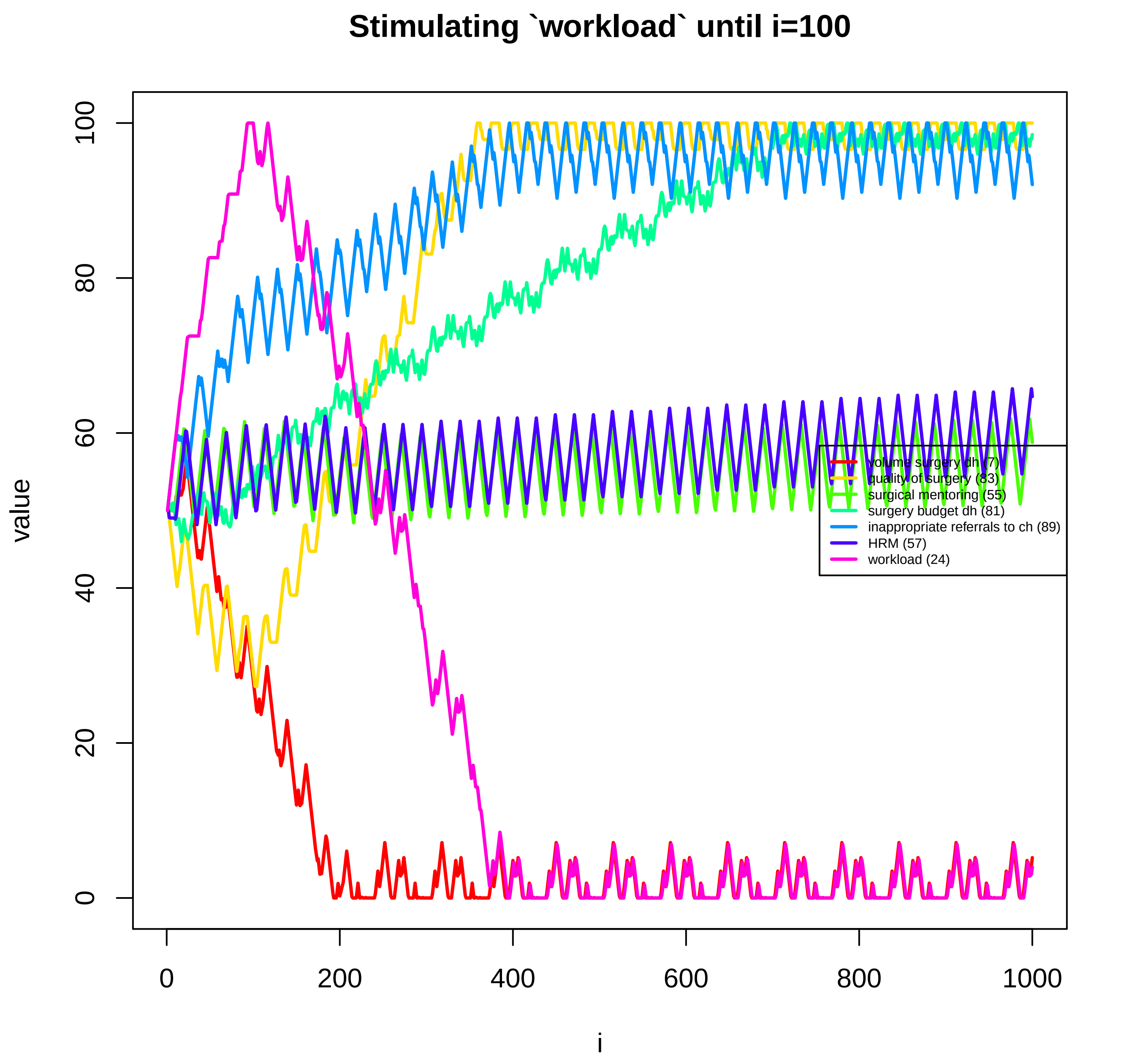
Stimulating `workload` until i=100
value
i
0
20
40
60
80
100
0
200
400
600
800
1000
volume surgery dh (7)
quality of surgery (83)
surgical mentoring (55)
surgery budget dh (81)
inappropriate referrals to ch (89)
HRM (57)
workload (24)

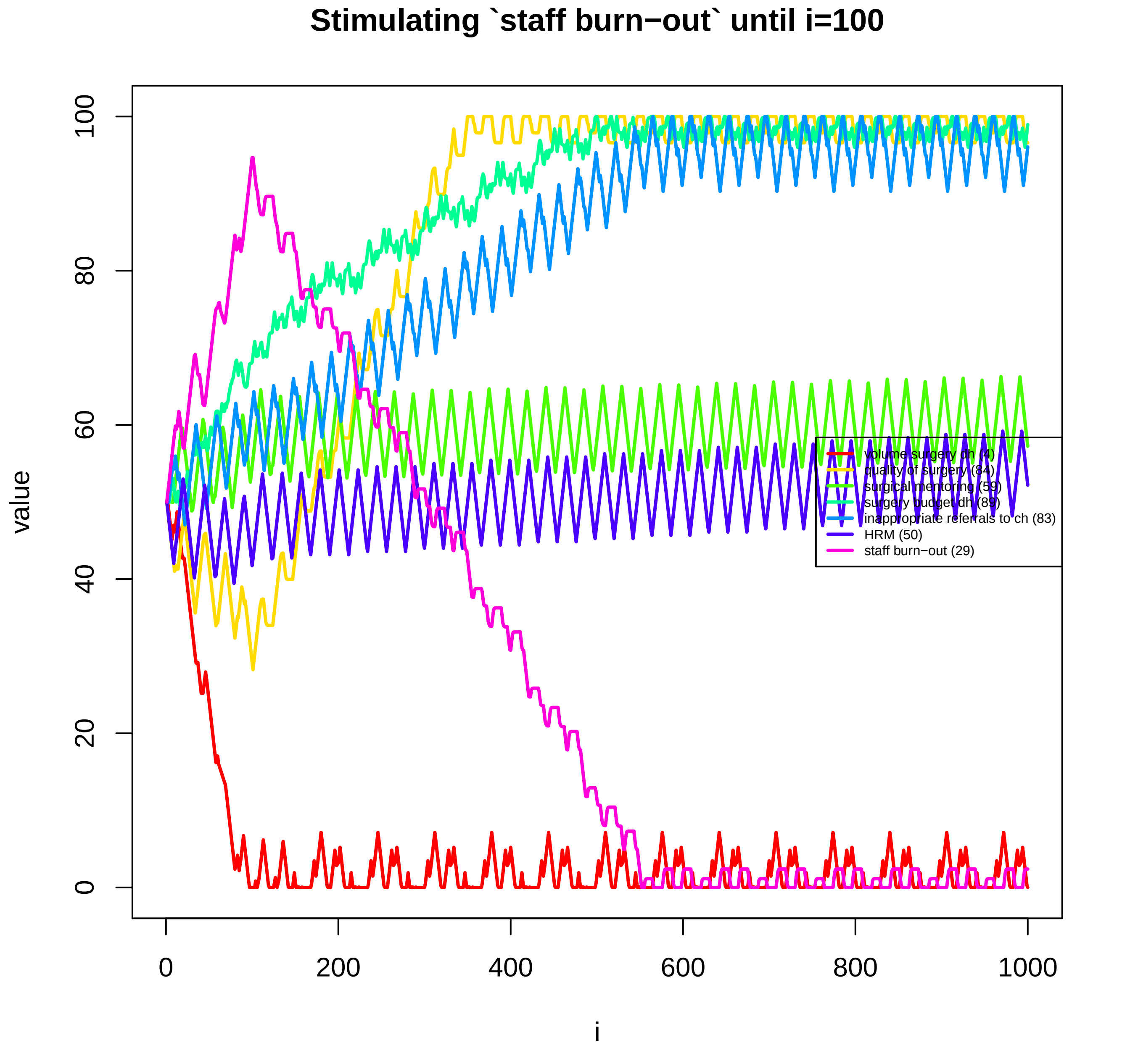

Stimulating `staff burn−out` until i=100
value
i
0
20
40
60
80
100
0
200
400
600
800
1000
volume surgery dh (4)
quality of surgery (84)
surgical mentoring (59)
surgery budget dh (89)
inappropriate referrals to ch (83)
HRM (50)
staff burn−out (29)

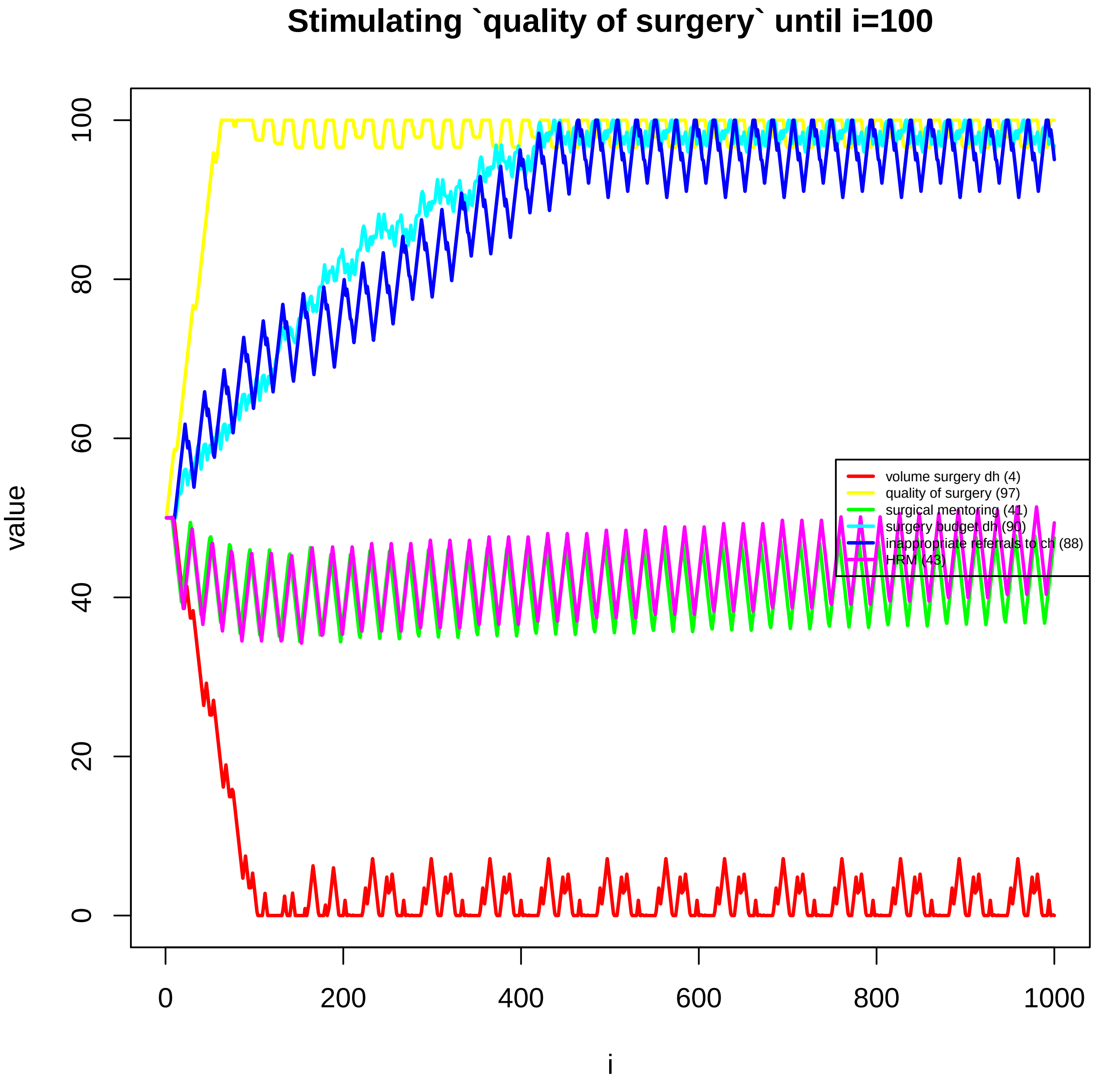

Stimulating `quality of surgery` until i=100
value
i
0
20
40
60
80
100
0
200
400
600
800
1000
volume surgery dh (4)
quality of surgery (97)
surgical mentoring (41)
surgery budget dh (90)
inappropriate referrals to ch (88)
HRM (43)

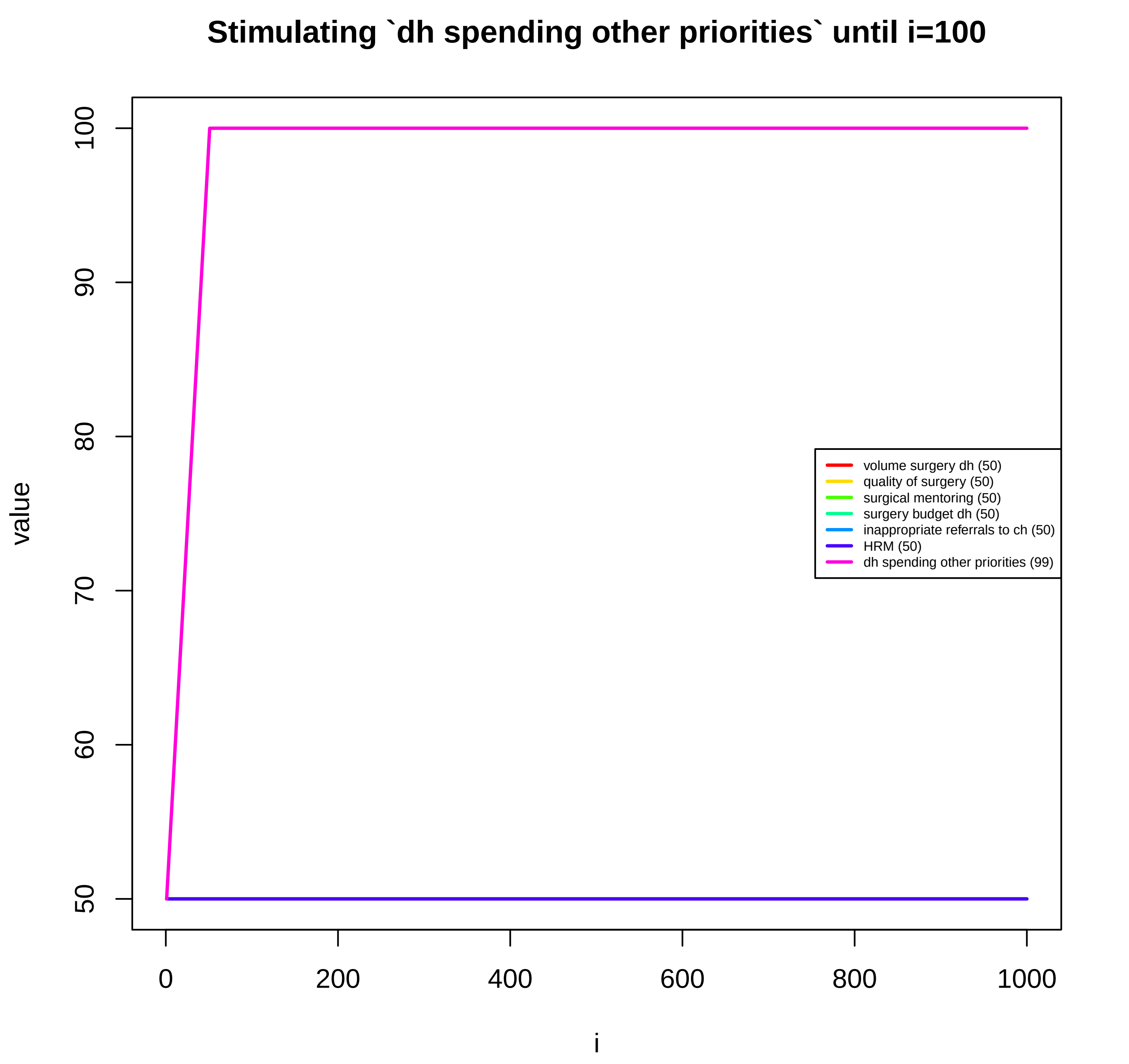
Stimulating `dh spending other priorities` until i=100
value
100
90
80
70
60
50
0
200
400
600
800
1000
i
volume surgery dh (50)
quality of surgery (50)
surgical mentoring (50)
surgery budget dh (50)
inappropriate referrals to ch (50)
HRM (50)
dh spending other priorities (99)

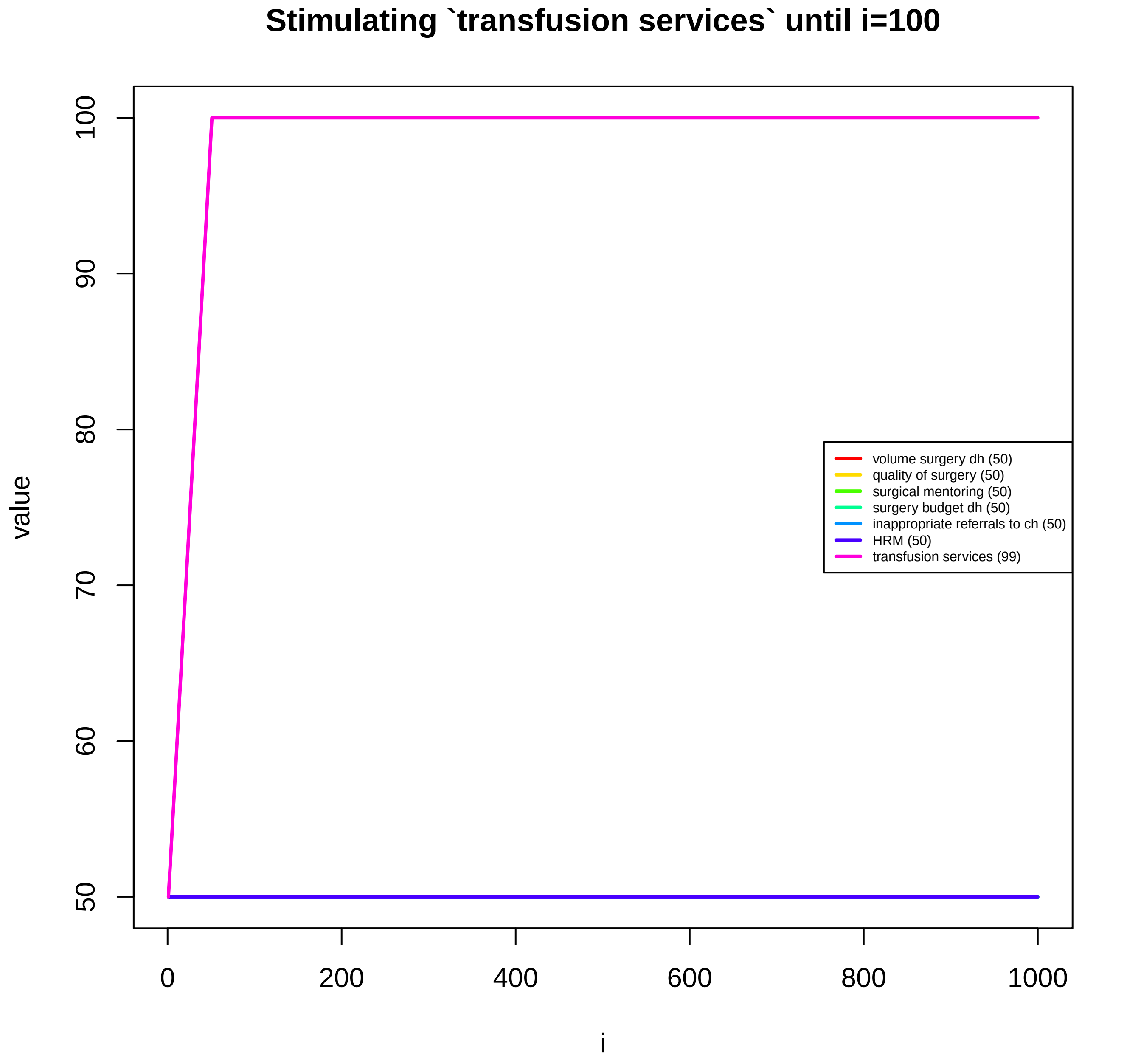

Stimulating `transfusion services` until i=100
value
100
90
80
70
60
50
0
200
400
600
800
1000
i
volume surgery dh (50)
quality of surgery (50)
surgical mentoring (50)
surgery budget dh (50)
inappropriate referrals to ch (50)
HRM (50)
transfusion services (99)

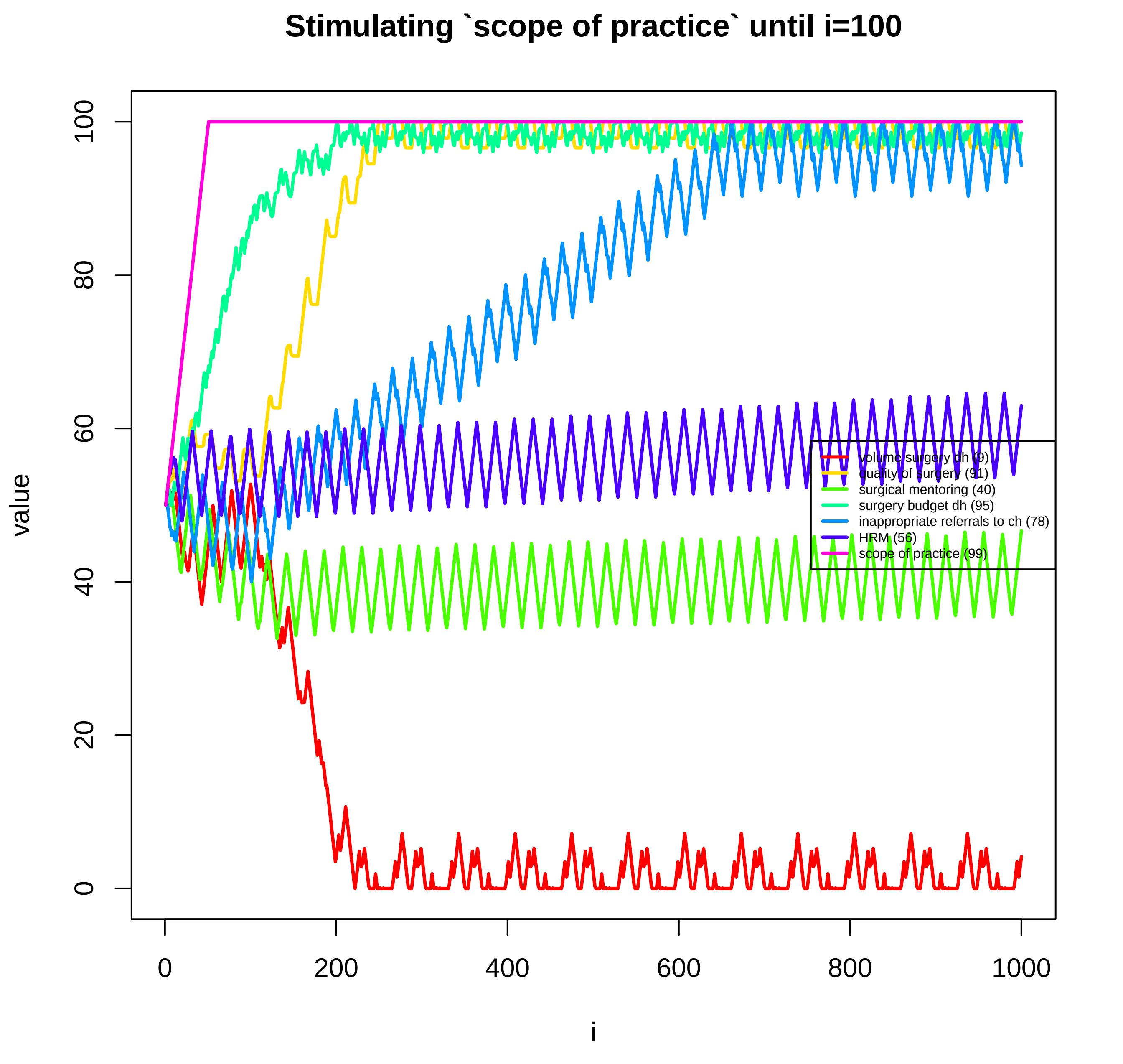

Stimulating `scope of practice` until i=100
value
i
0
20
40
60
80
100
0
200
400
600
800
1000
volume surgery dh (9)
quality of surgery (91)
surgical mentoring (40)
surgery budget dh (95)
inappropriate referrals to ch (78)
HRM (56)
scope of practice (99)

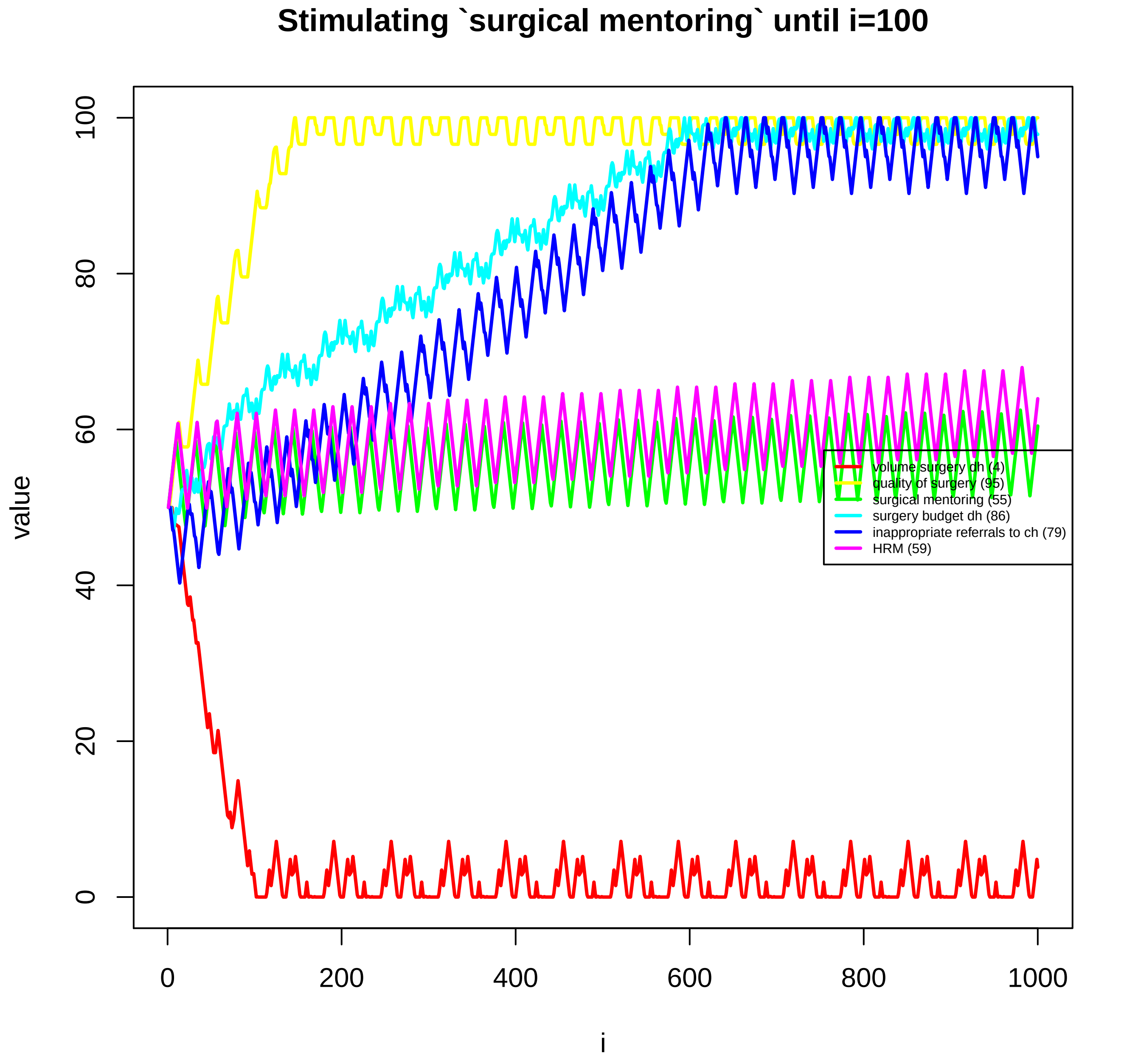

Stimulating `surgical mentoring` until i=100
value
i
0
20
40
60
80
100
0
200
400
600
800
1000
volume surgery dh (4)
quality of surgery (95)
surgical mentoring (55)
surgery budget dh (86)
inappropriate referrals to ch (79)
HRM (59)

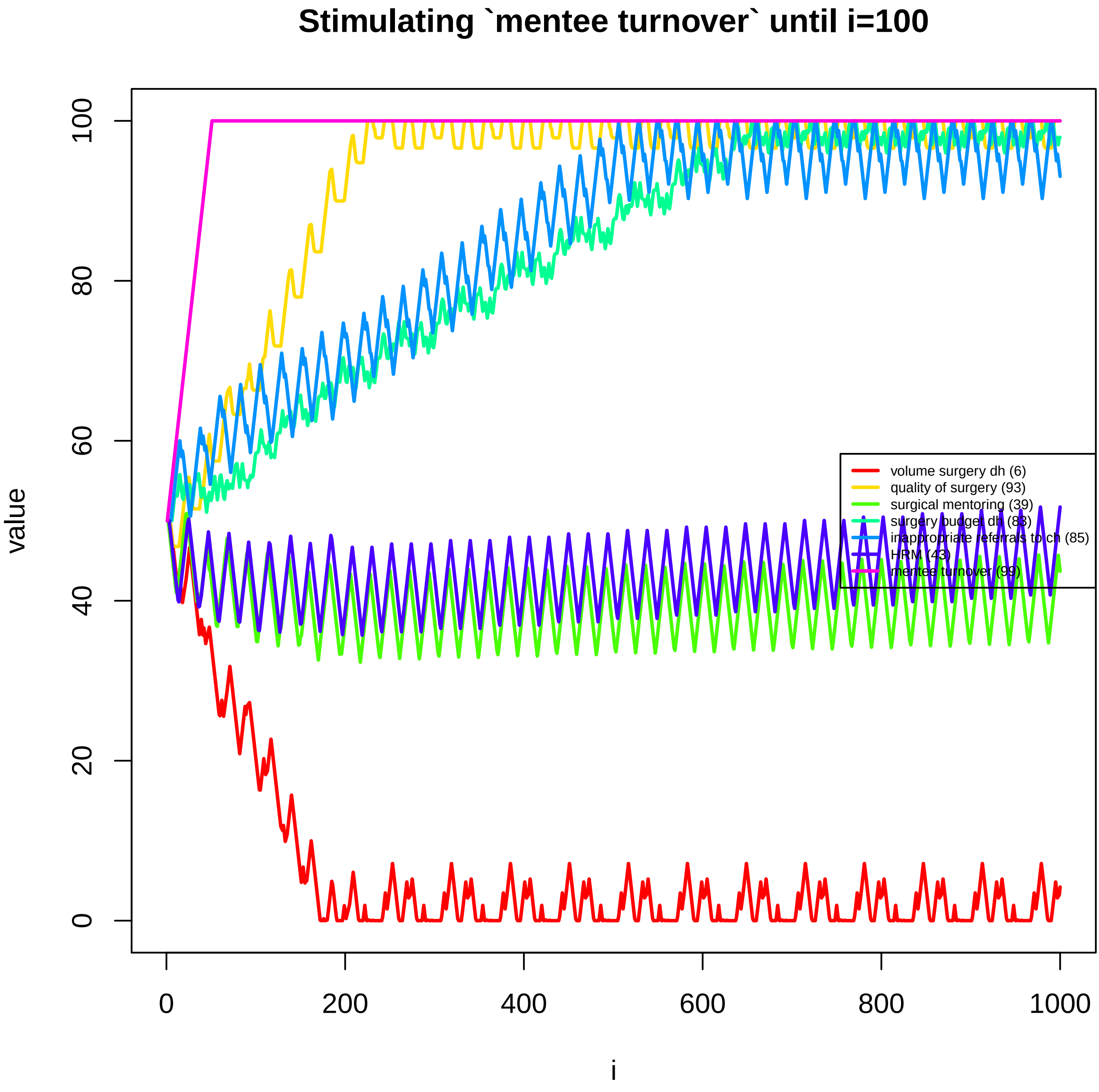

Stimulating `mentee turnover` until i=100
value
i
0
20
40
60
80
100
0
200
400
600
800
1000
volume surgery dh (6)
quality of surgery (93)
surgical mentoring (39)
surgery budget dh (83)
inappropriate referrals to ch (85)
HRM (43)
mentee turnover (99)

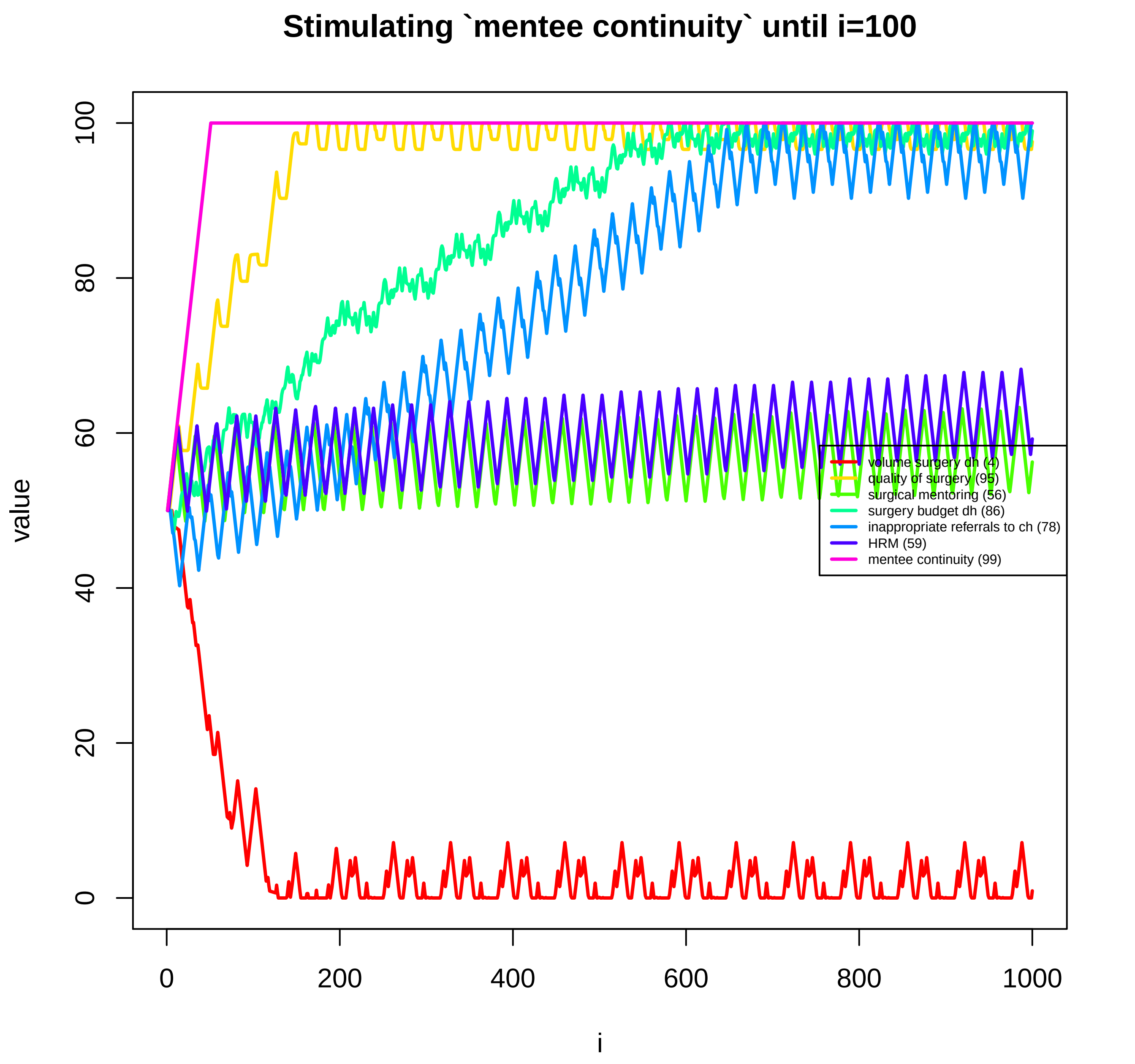

Stimulating `mentee continuity` until i=100
value
i
0
20
40
60
80
100
0
200
400
600
800
1000
volume surgery dh (4)
quality of surgery (95)
surgical mentoring (56)
surgery budget dh (86)
inappropriate referrals to ch (78)
HRM (59)
mentee continuity (99)

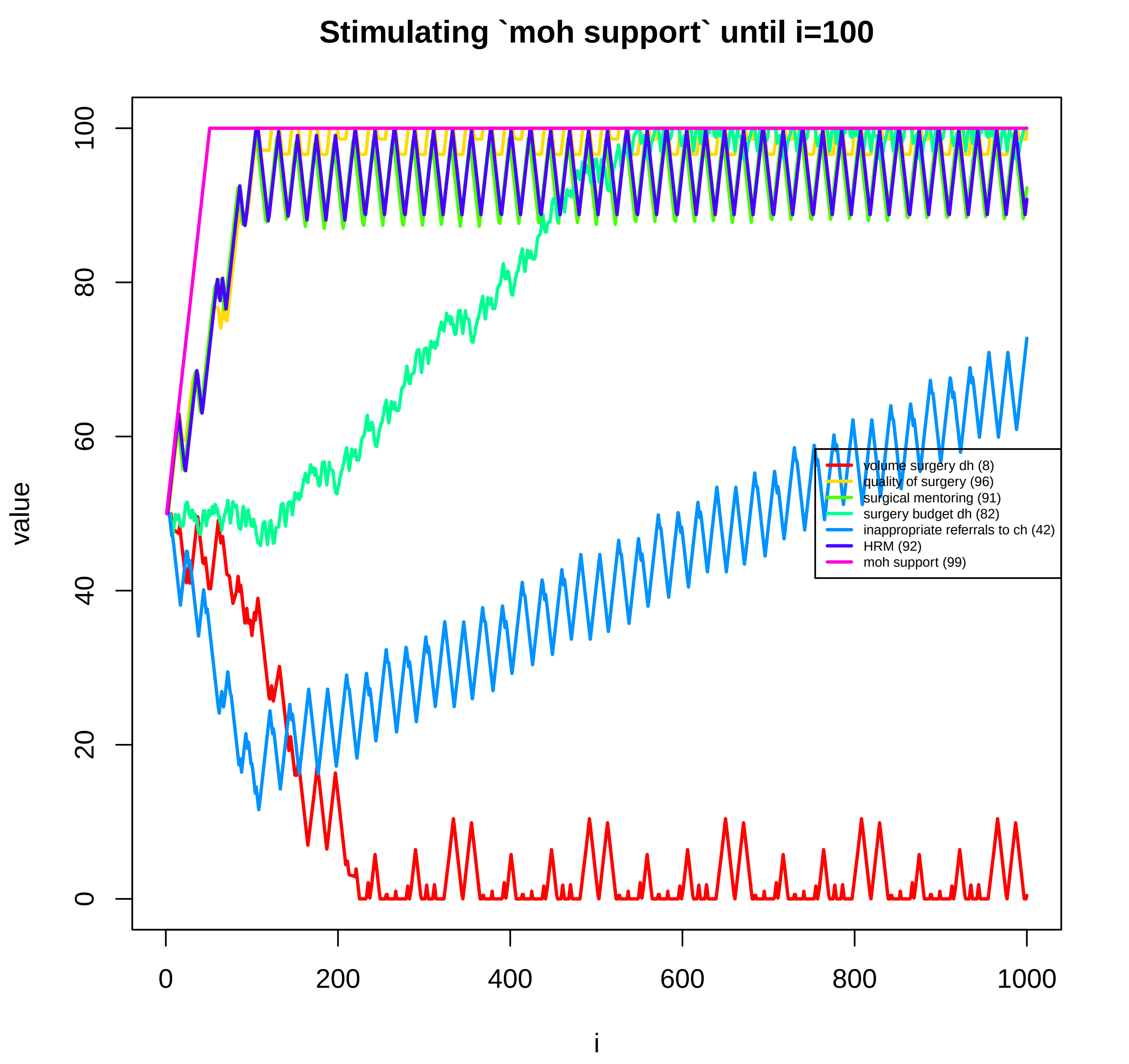

Stimulating `moh support` until i=100
value
i
0
20
40
60
80
100
0
200
400
600
800
1000
volume surgery dh (8)
quality of surgery (96)
surgical mentoring (91)
surgery budget dh (82)
inappropriate referrals to ch (42)
HRM (92)
moh support (99)

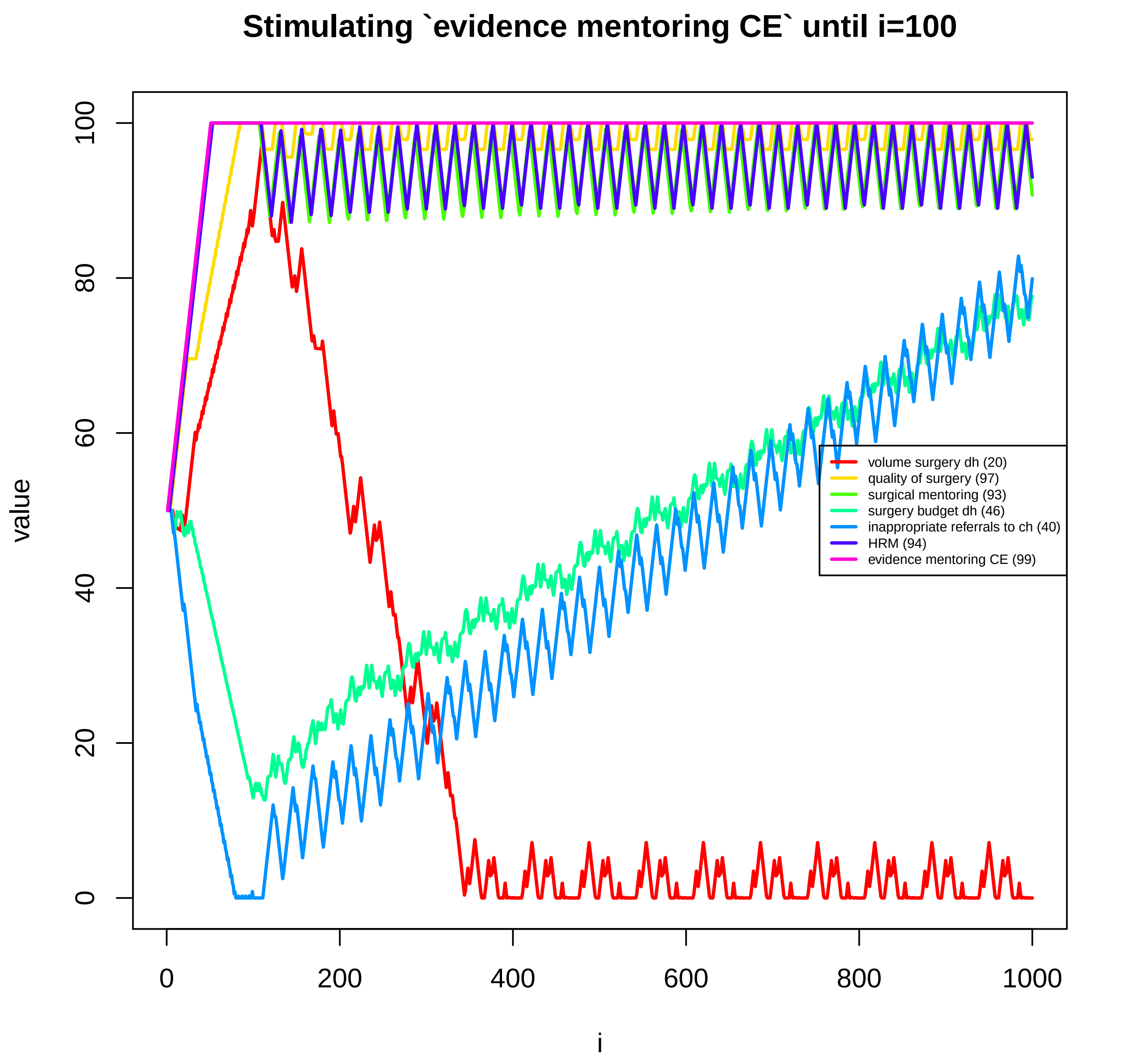
Stimulating `evidence mentoring CE` until i=100
value
i
0
20
40
60
80
100
0
200
400
600
800
1000
volume surgery dh (20)
quality of surgery (97)
surgical mentoring (93)
surgery budget dh (46)
inappropriate referrals to ch (40)
HRM (94)
evidence mentoring CE (99)

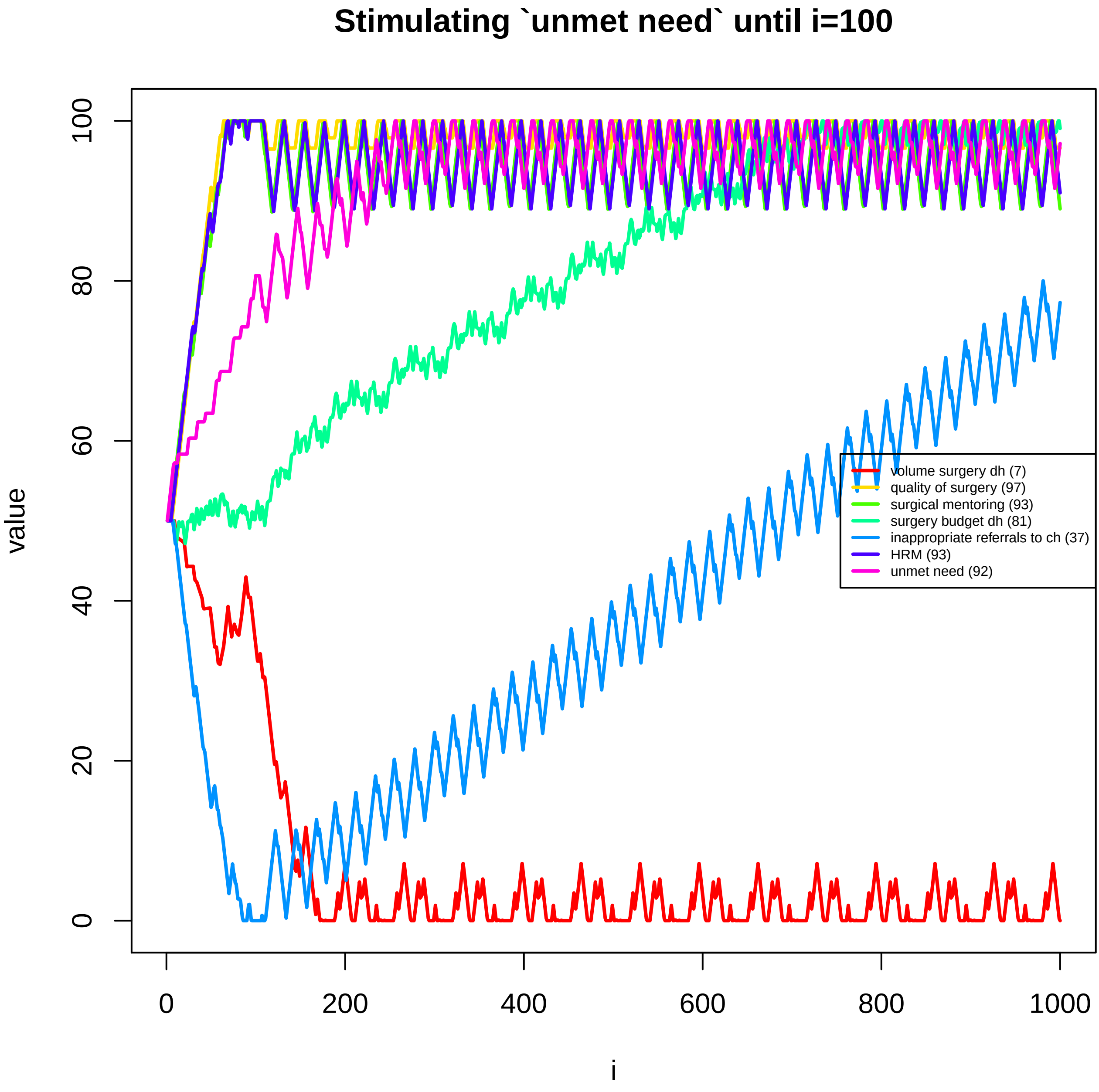

Stimulating `unmet need` until i=100
value
i
0
20
40
60
80
100
0
200
400
600
800
1000
volume surgery dh (7)
quality of surgery (97)
surgical mentoring (93)
surgery budget dh (81)
inappropriate referrals to ch (37)
HRM (93)
unmet need (92)

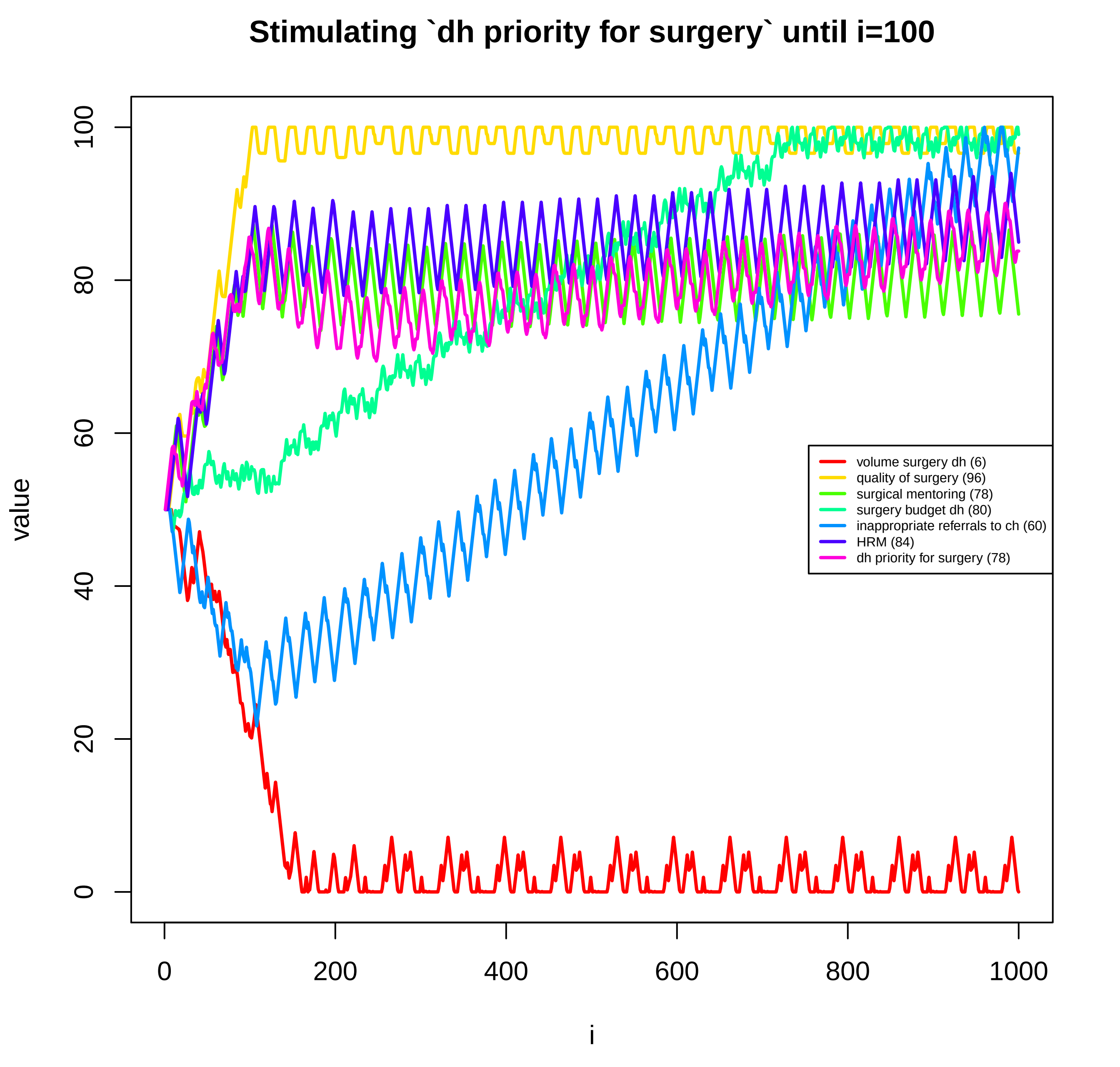

Stimulating `dh priority for surgery` until i=100
value
i
0
20
40
60
80
100
0
200
400
600
800
1000
volume surgery dh (6)
quality of surgery (96)
surgical mentoring (78)
surgery budget dh (80)
inappropriate referrals to ch (60)
HRM (84)
dh priority for surgery (78)

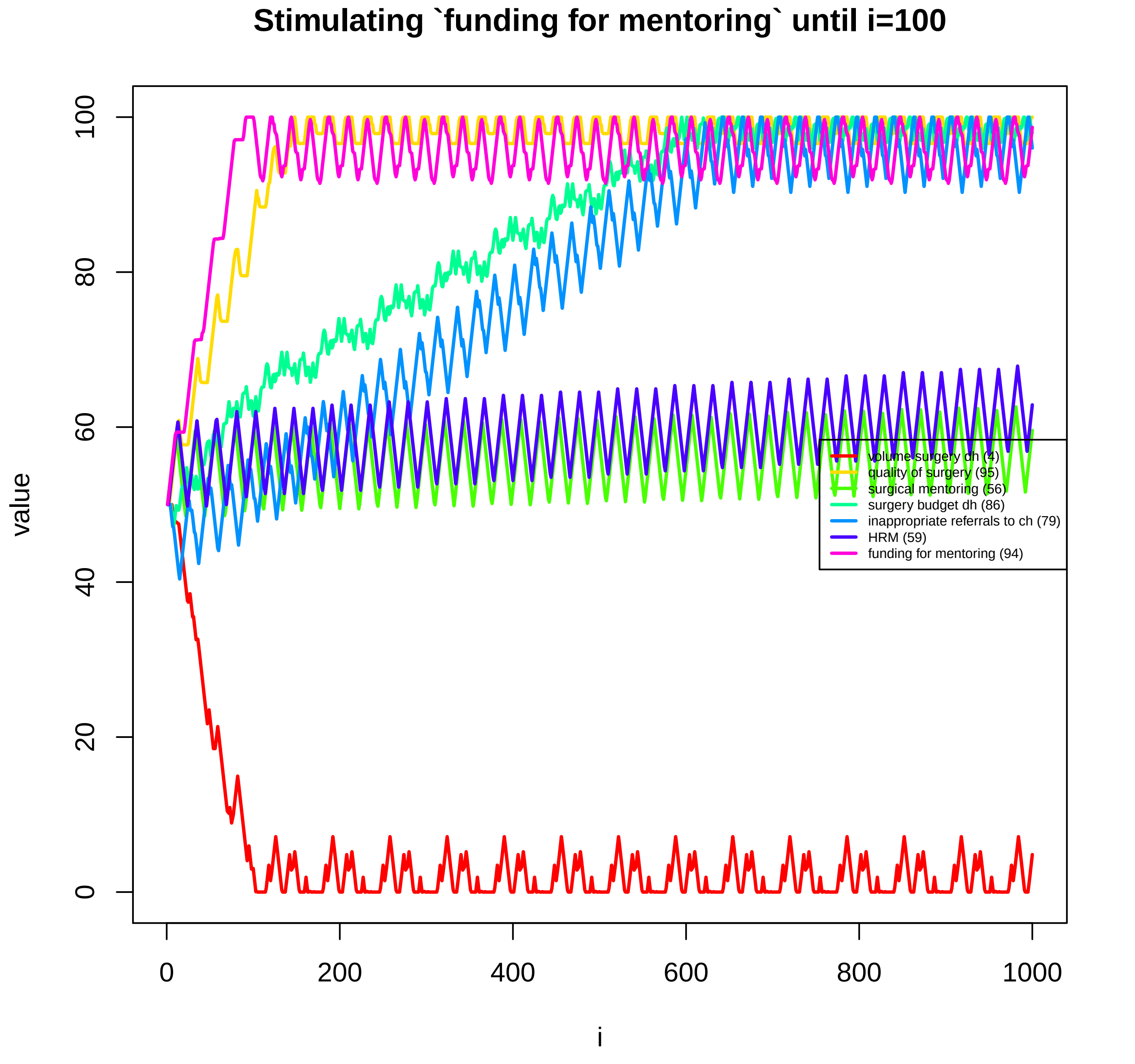

Stimulating `funding for mentoring` until i=100
value
i
0
20
40
60
80
100
0
200
400
600
800
1000
volume surgery dh (4)
quality of surgery (95)
surgical mentoring (56)
surgery budget dh (86)
inappropriate referrals to ch (79)
HRM (59)
funding for mentoring (94)

Stimulating `mngmnt information system w/ clear surgical indicators` until i=

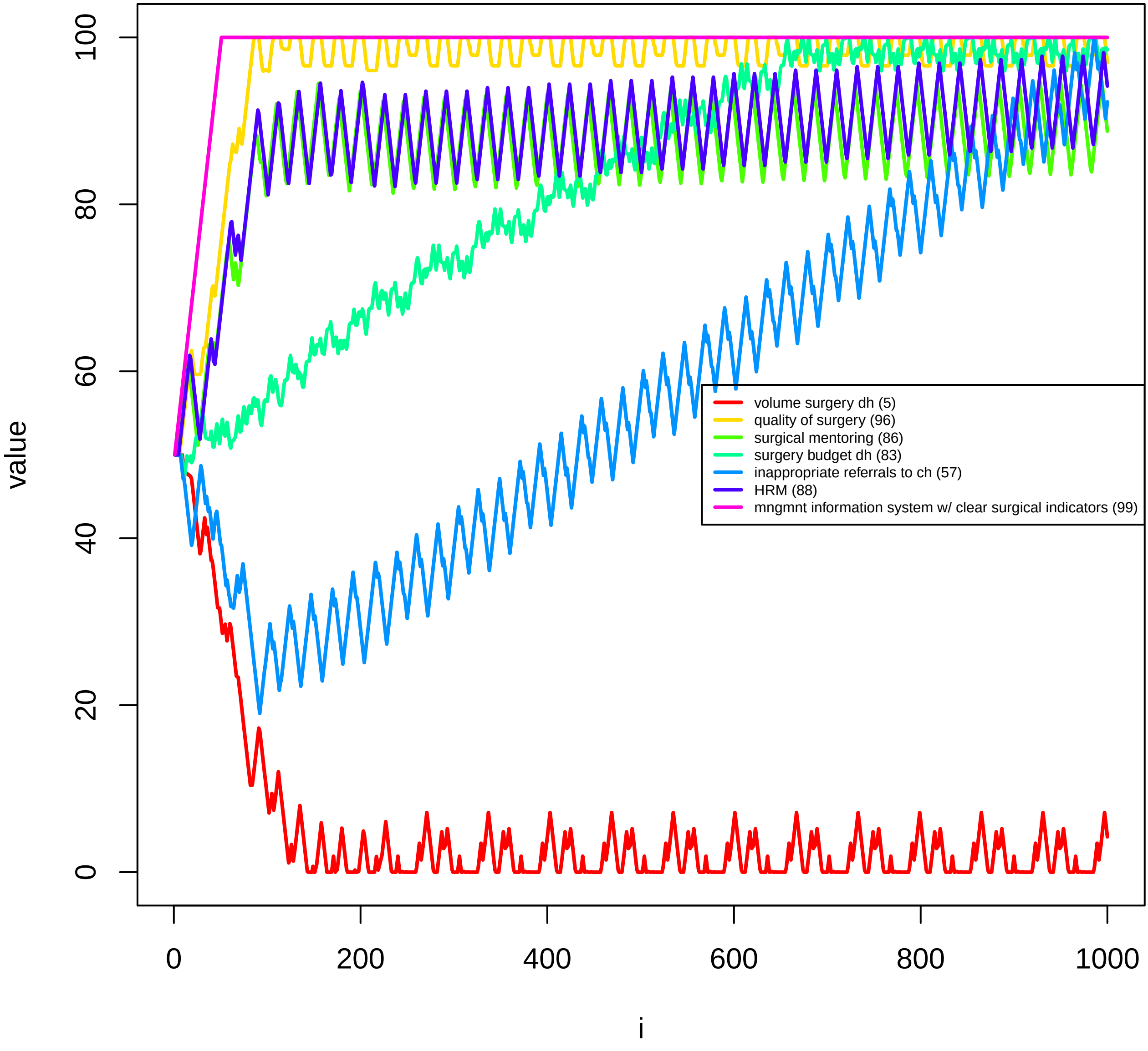

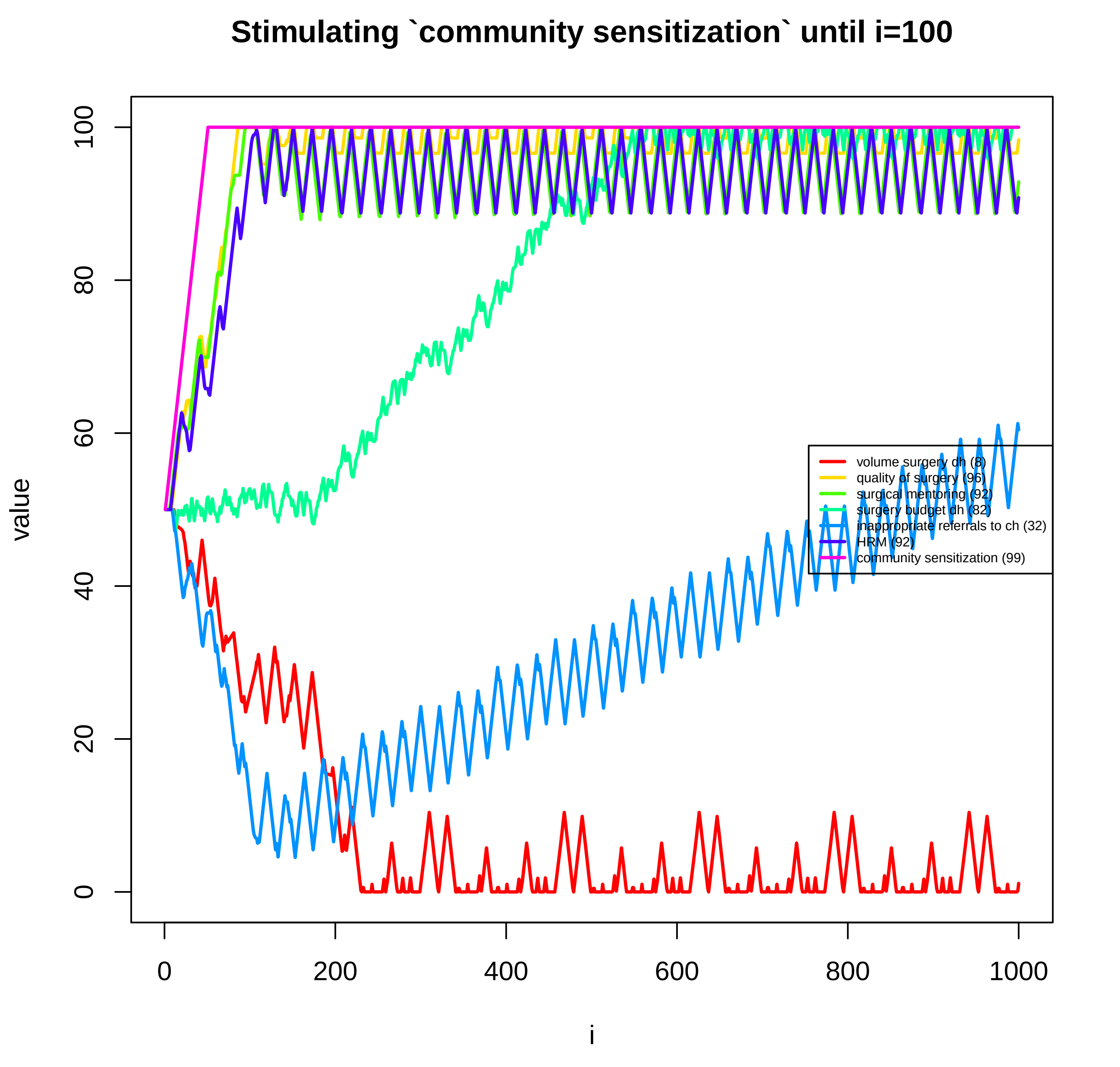

Stimulating `community sensitization` until i=100
value
i
0
20
40
60
80
100
0
200
400
600
800
1000
volume surgery dh (8)
quality of surgery (96)
surgical mentoring (92)
surgery budget dh (82)
inappropriate referrals to ch (32)
HRM (92)
community sensitization (99)

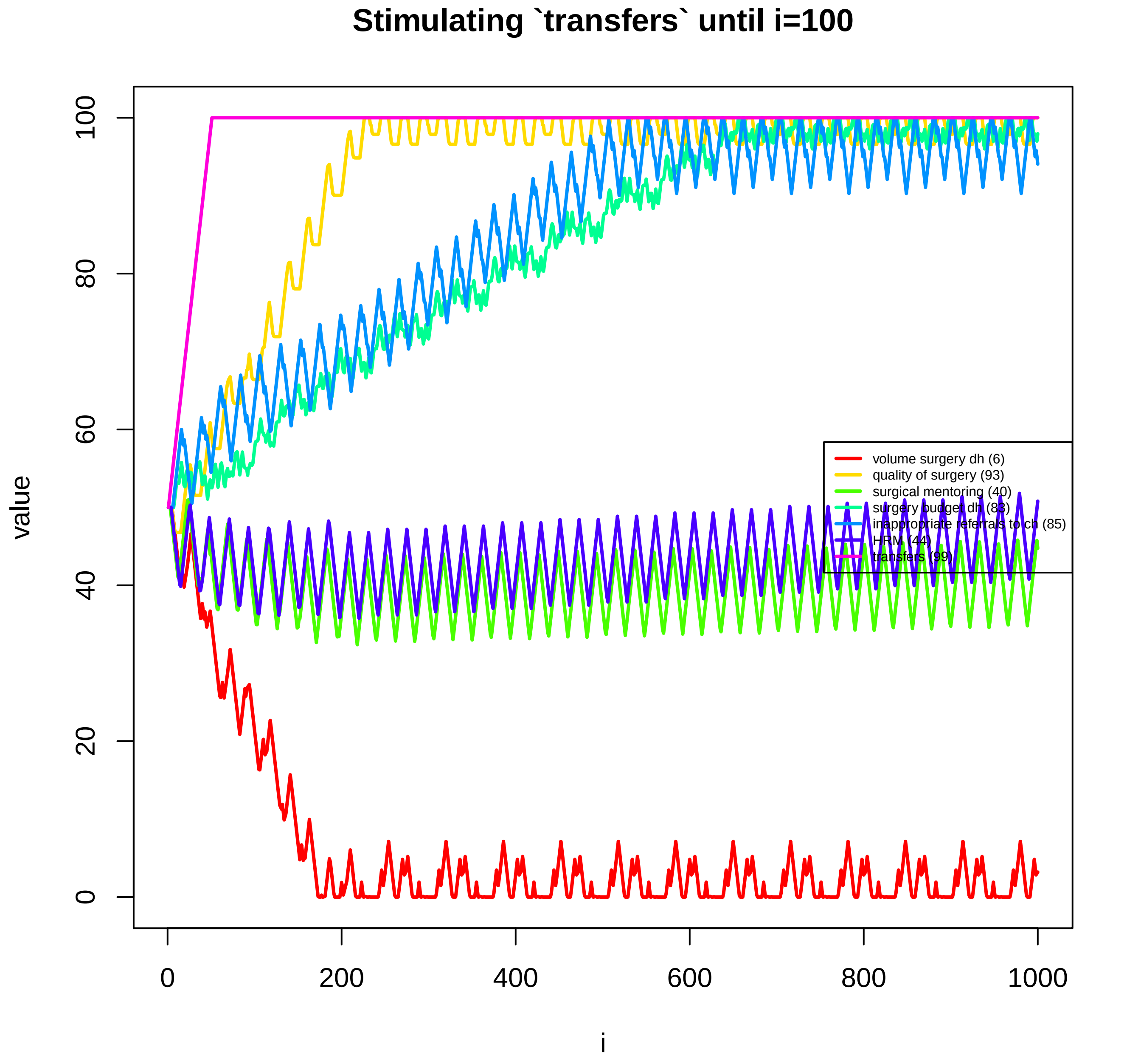

Stimulating `transfers` until i=100
value
i
0
20
40
60
80
100
0
200
400
600
800
1000
volume surgery dh (6)
quality of surgery (93)
surgical mentoring (40)
surgery budget dh (83)
inappropriate referrals to ch (85)
HRM (44)
transfers (99)

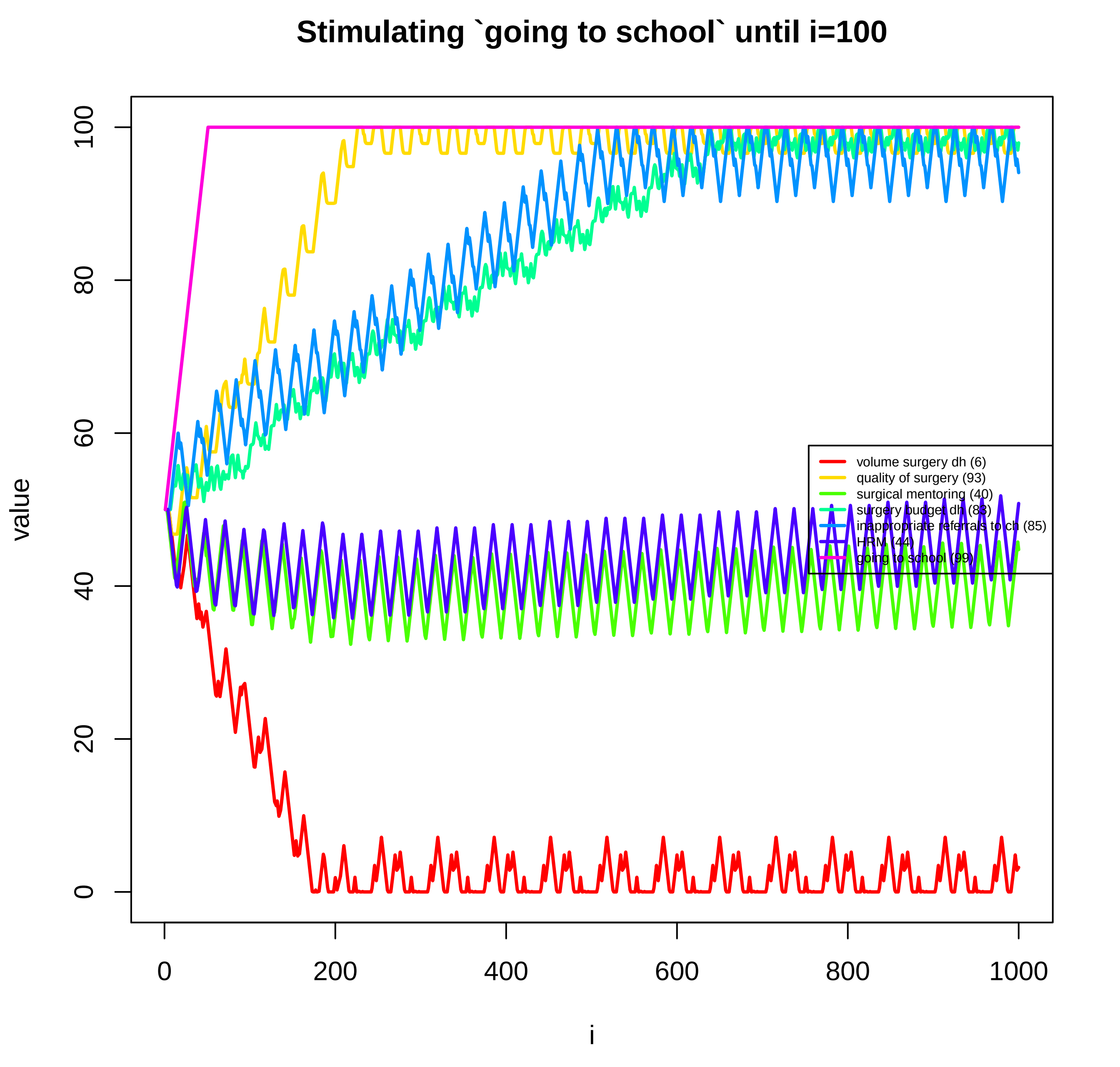
Stimulating `going to school` until i=100
value
i
0
20
40
60
80
100
0
200
400
600
800
1000
volume surgery dh (6)
quality of surgery (93)
surgical mentoring (40)
surgery budget dh (83)
inappropriate referrals to ch (85)
HRM (44)
going to school (99)

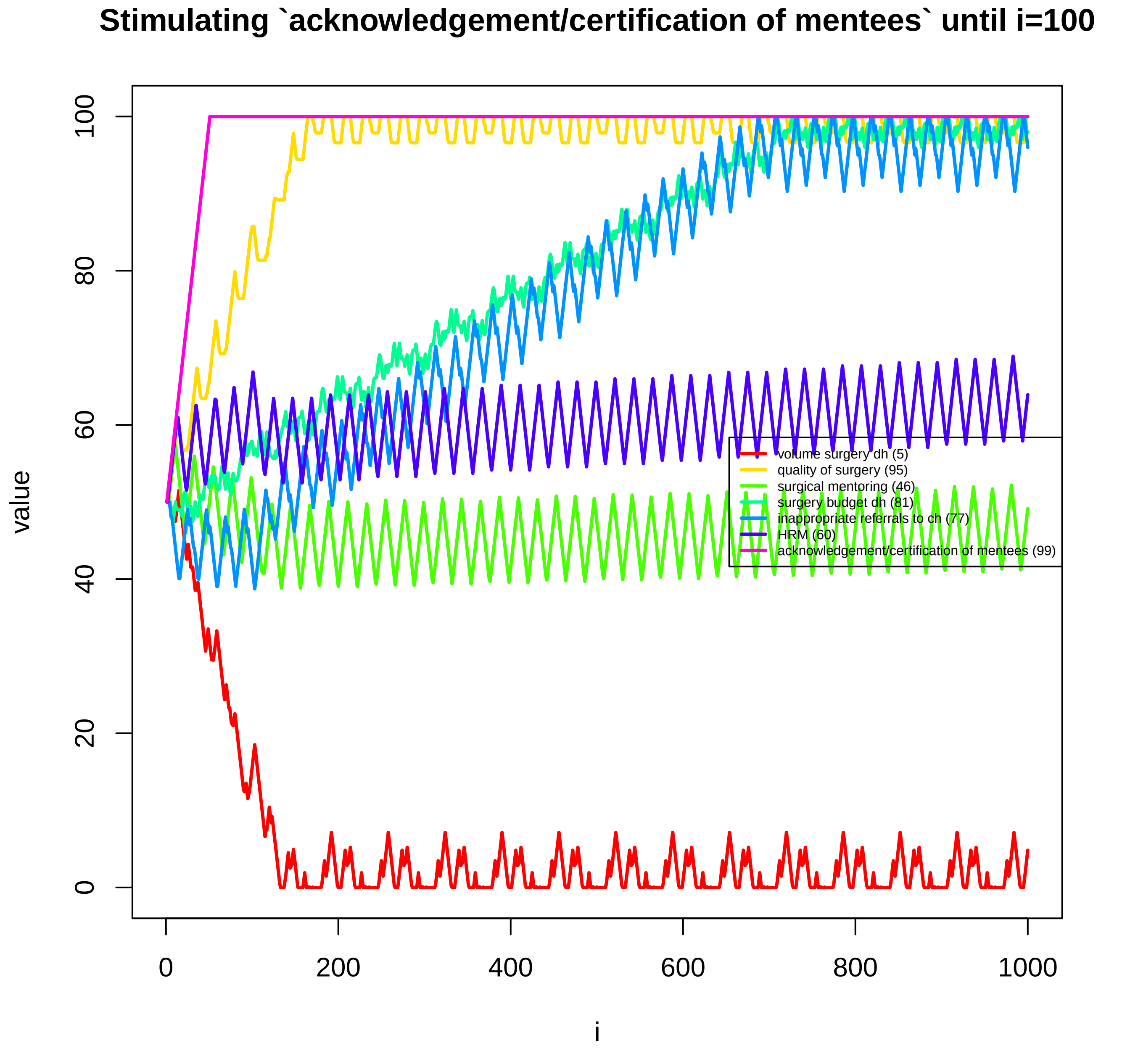

Stimulating `acknowledgement/certification of mentees` until i=100
value
i
0
20
40
60
80
100
0
200
400
600
800
1000
volume surgery dh (5)
quality of surgery (95)
surgical mentoring (46)
surgery budget dh (81)
inappropriate referrals to ch (77)
HRM (60)
acknowledgement/certification of mentees (99)

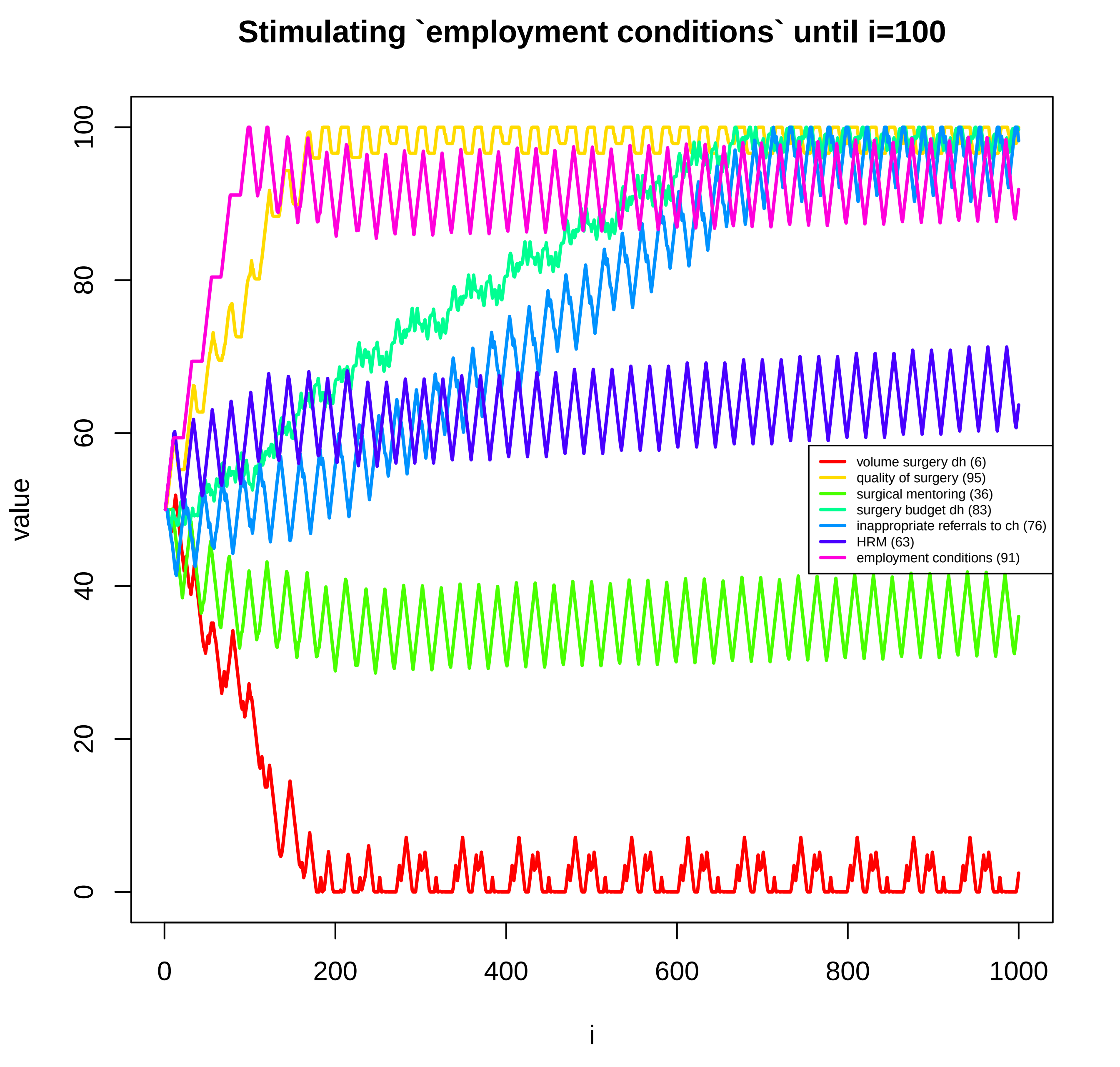

Stimulating `employment conditions` until i=100
value
i
0
20
40
60
80
100
0
200
400
600
800
1000
volume surgery dh (6)
quality of surgery (95)
surgical mentoring (36)
surgery budget dh (83)
inappropriate referrals to ch (76)
HRM (63)
employment conditions (91)

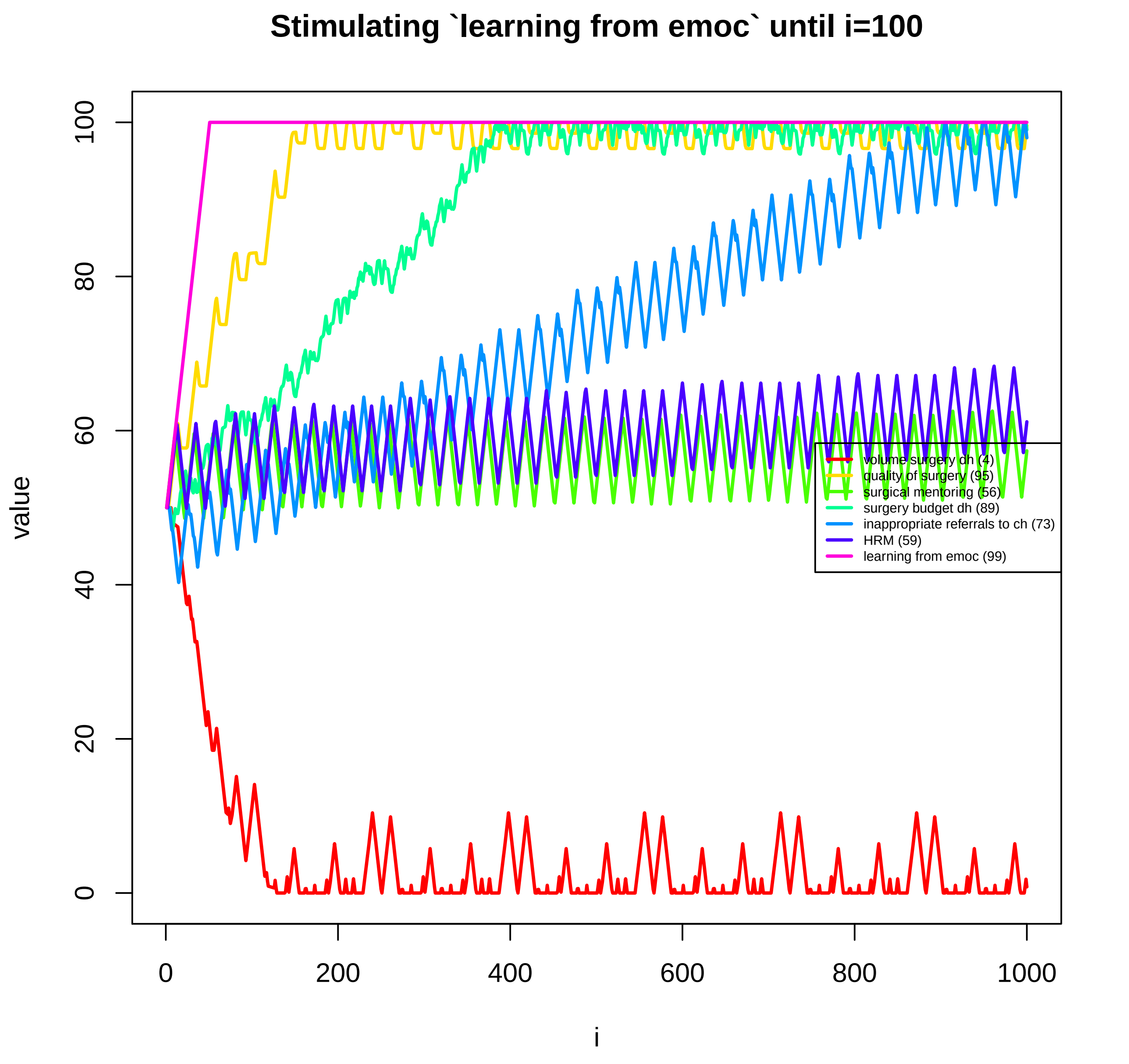
Stimulating `learning from emoc` until i=100
value
i
0
20
40
60
80
100
0
200
400
600
800
1000
volume surgery dh (4)
quality of surgery (95)
surgical mentoring (56)
surgery budget dh (89)
inappropriate referrals to ch (73)
HRM (59)
learning from emoc (99)

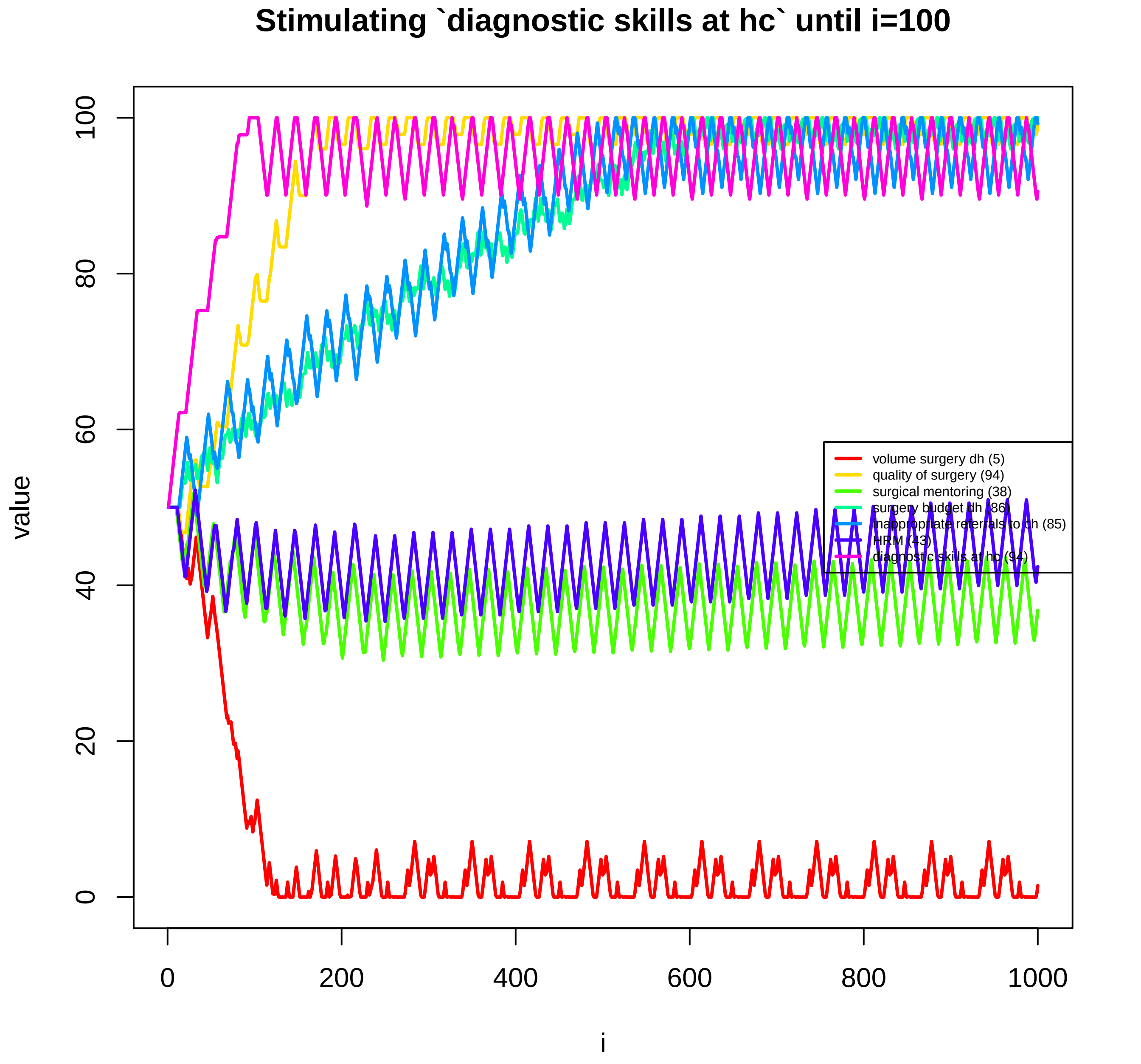

Stimulating `diagnostic skills at hc` until i=100
value
i
0
20
40
60
80
100
0
200
400
600
800
1000
volume surgery dh (5)
quality of surgery (94)
surgical mentoring (38)
surgery budget dh (86)
inappropriate referrals to dh (85)
HRM (43)
diagnostic skills at hc (94)

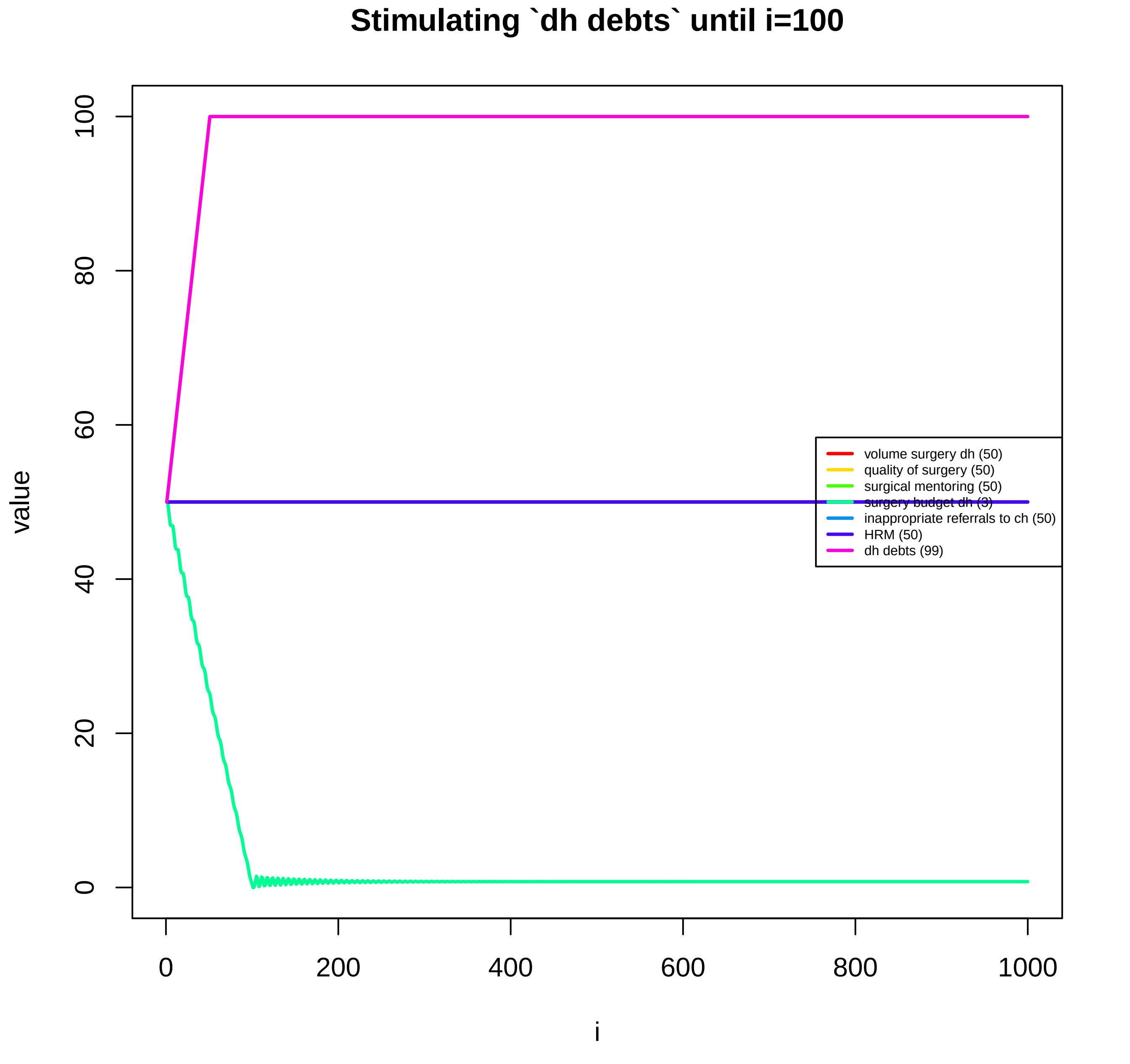

Stimulating `dh debts` until i=100
value
i
0
20
40
60
80
100
0
200
400
600
800
1000
volume surgery dh (50)
quality of surgery (50)
surgical mentoring (50)
surgery budget dh (3)
inappropriate referrals to ch (50)
HRM (50)
dh debts (99)

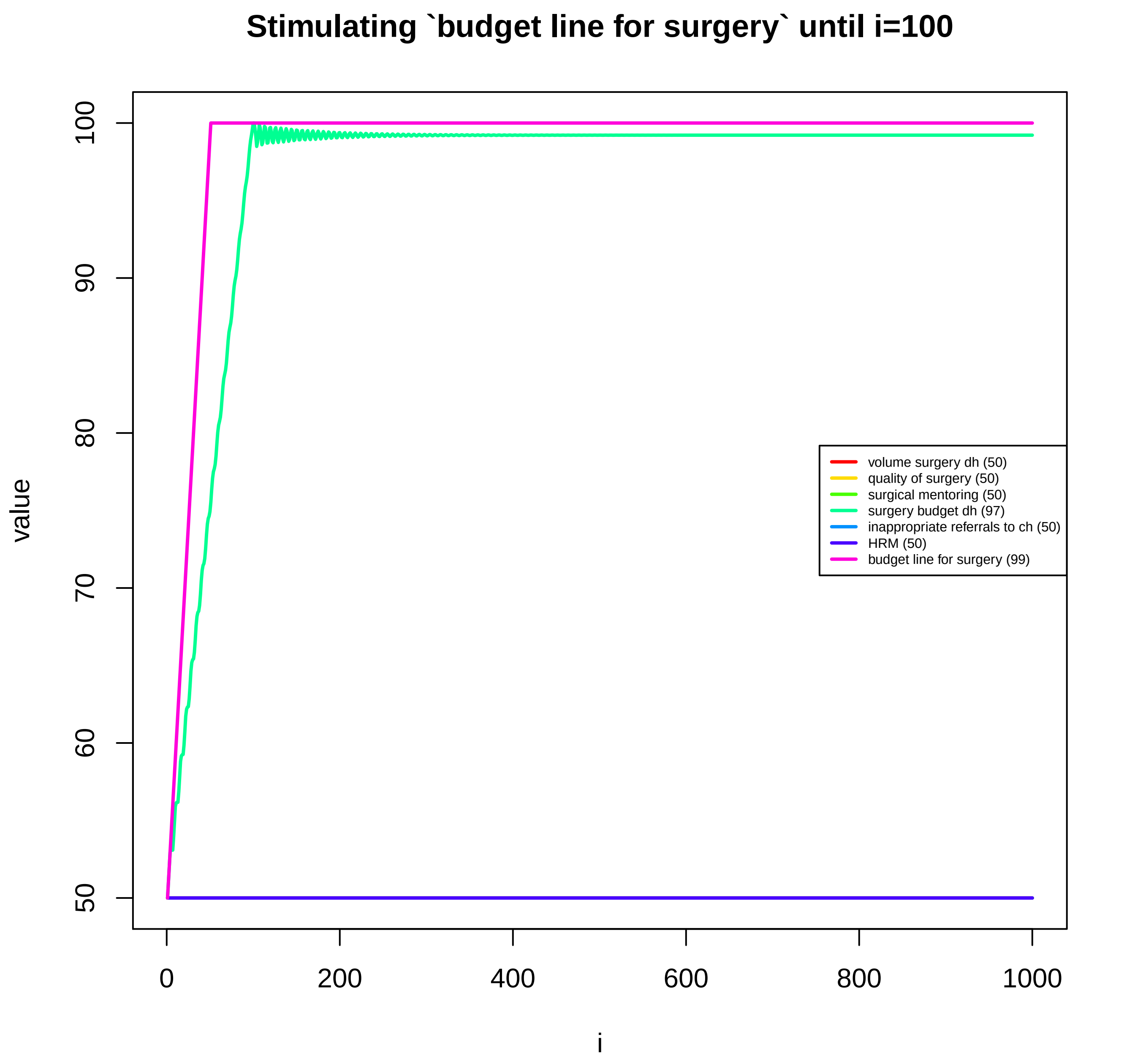

Stimulating `budget line for surgery` until i=100
value
i
100
90
80
70
60
50
0
200
400
600
800
1000
volume surgery dh (50)
quality of surgery (50)
surgical mentoring (50)
surgery budget dh (97)
inappropriate referrals to ch (50)
HRM (50)
budget line for surgery (99)

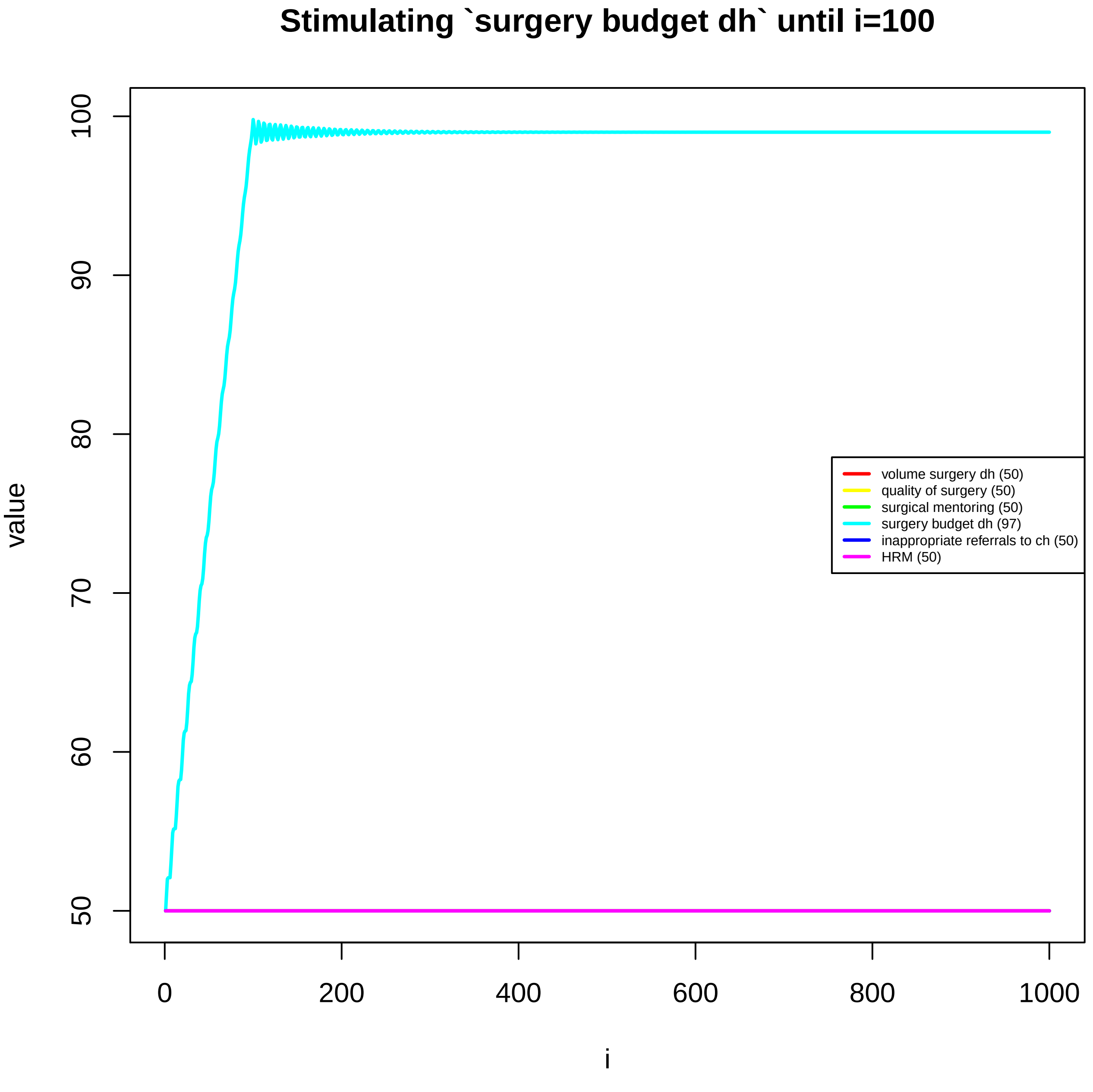

Stimulating `surgery budget dh` until i=100
value
100
90
80
70
60
50
0
200
400
600
800
1000
i
volume surgery dh (50)
quality of surgery (50)
surgical mentoring (50)
surgery budget dh (97)
inappropriate referrals to ch (50)
HRM (50)

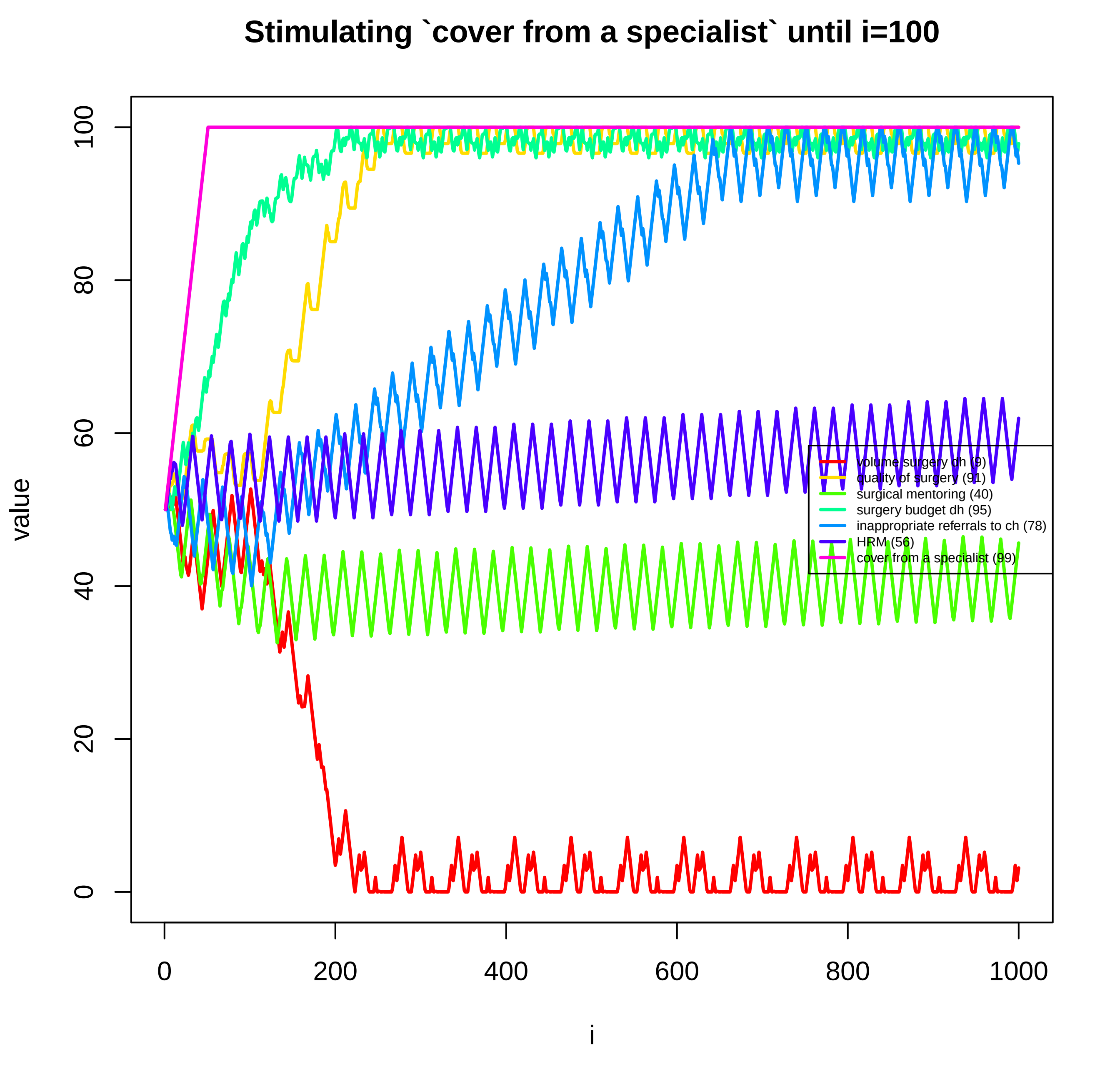

Stimulating `cover from a specialist` until i=100
value
i
0
20
40
60
80
100
0
200
400
600
800
1000
volume surgery dh (9)
quality of surgery (91)
surgical mentoring (40)
surgery budget dh (95)
inappropriate referrals to ch (78)
HRM (56)
cover from a specialist (99)

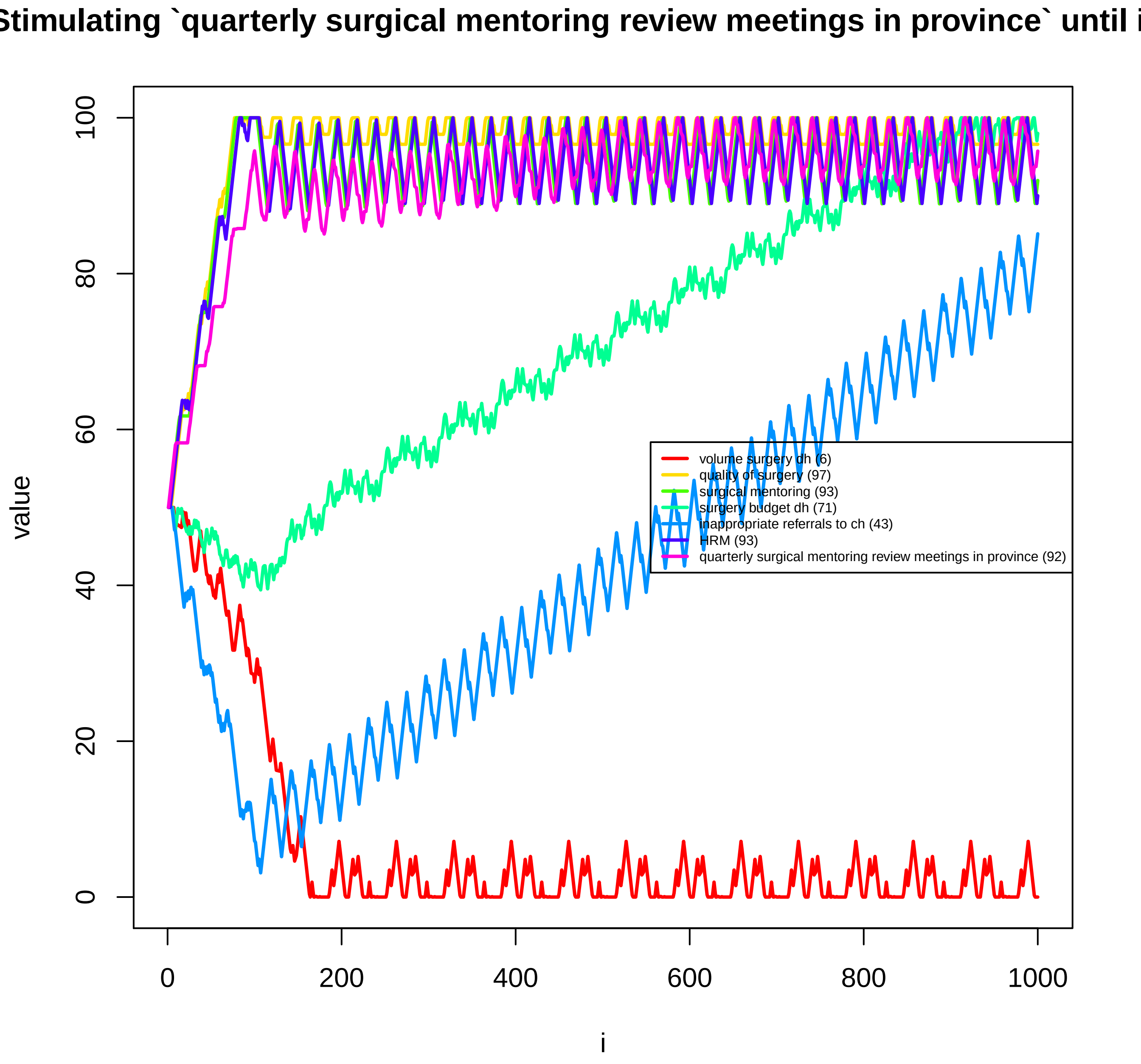
Stimulating `quarterly surgical mentoring review meetings in province` until i
value
i
0
20
40
60
80
100
0
200
400
600
800
1000
volume surgery dh (6)
quality of surgery (97)
surgical mentoring (93)
surgery budget dh (71)
inappropriate referrals to ch (43)
HRM (93)
quarterly surgical mentoring review meetings in province (92)

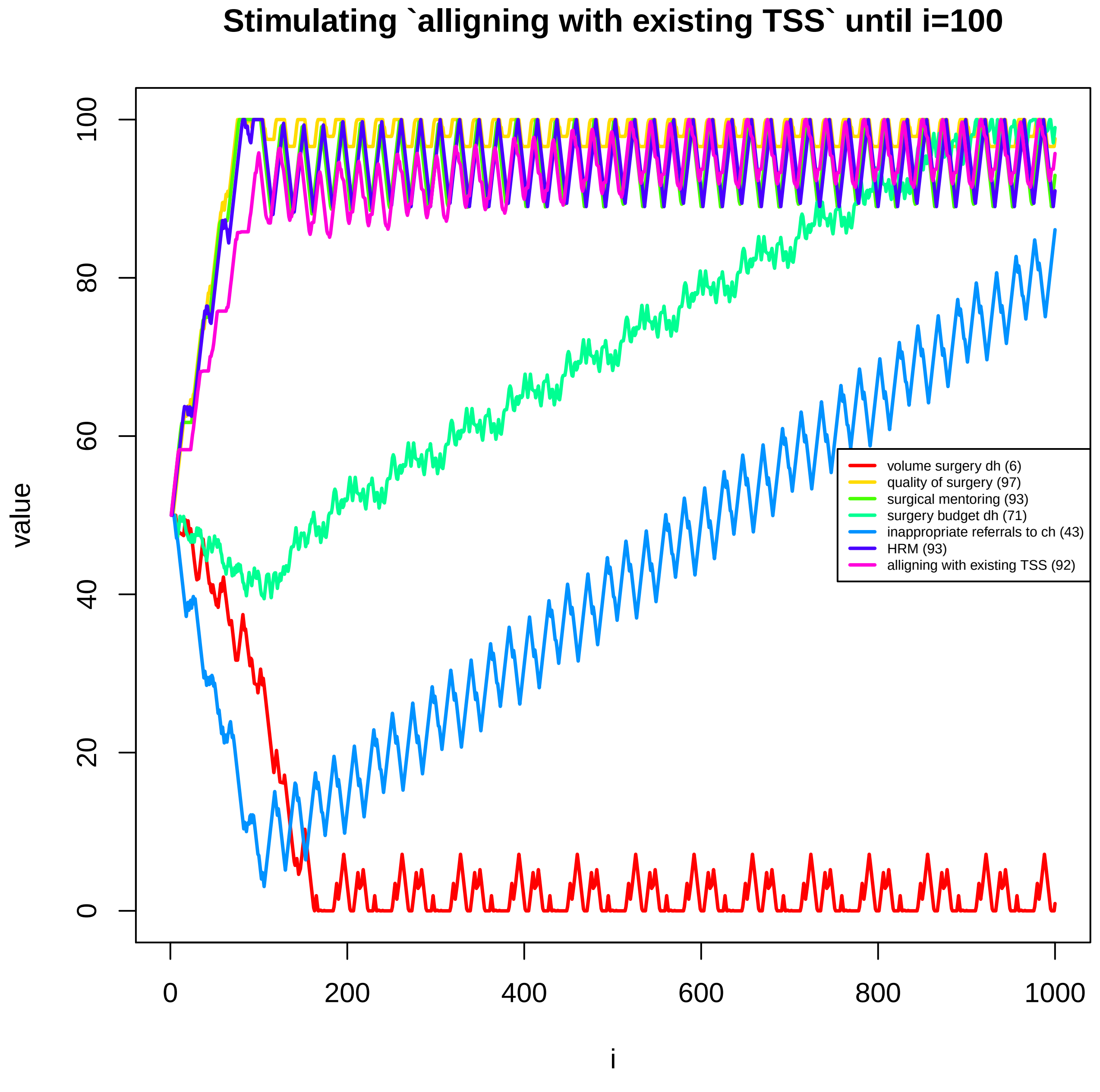
Stimulating `alligning with existing TSS` until i=100
value
i
0
20
40
60
80
100
0
200
400
600
800
1000
volume surgery dh (6)
quality of surgery (97)
surgical mentoring (93)
surgery budget dh (71)
inappropriate referrals to ch (43)
HRM (93)
alligning with existing TSS (92)

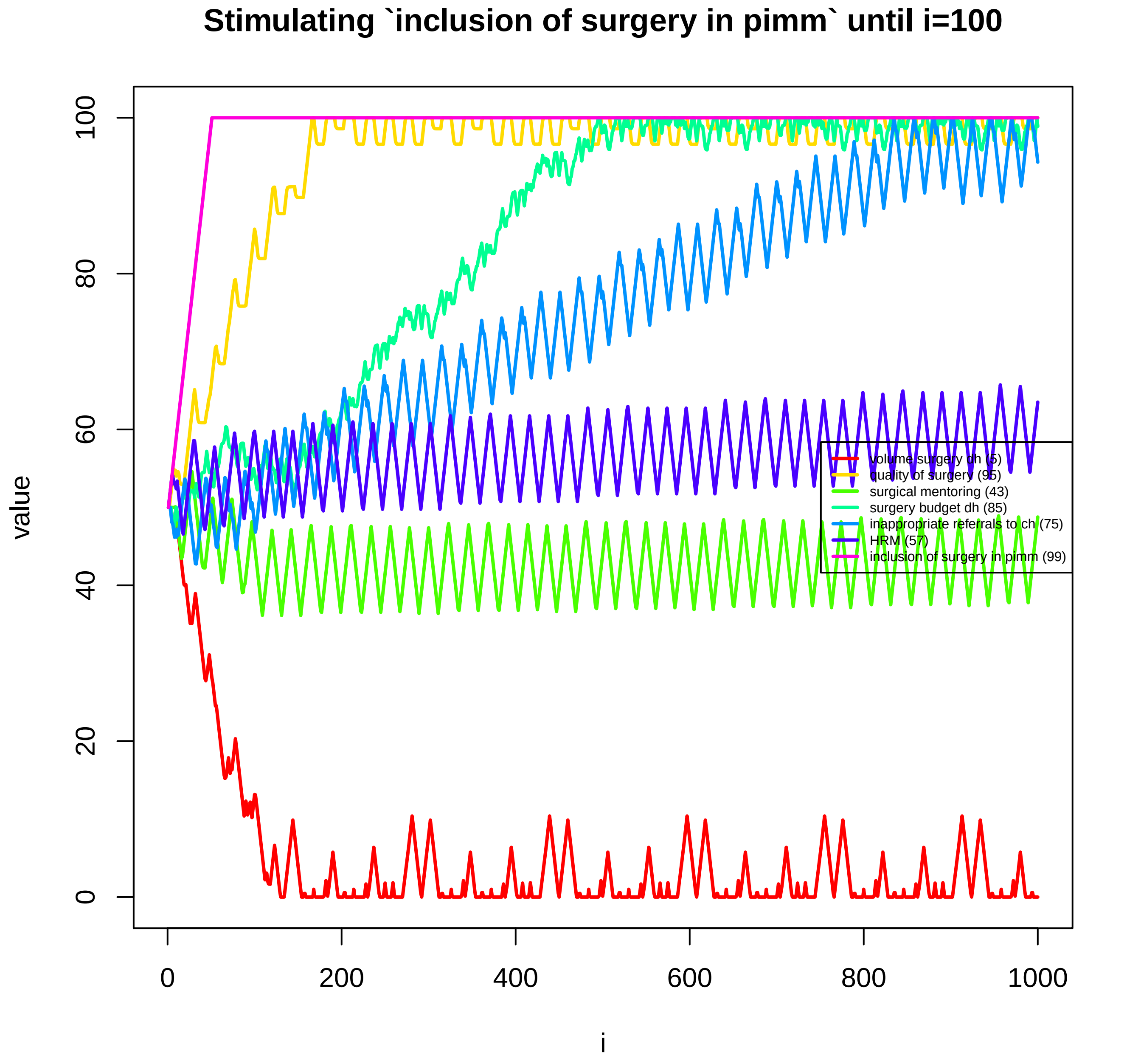

Stimulating `inclusion of surgery in pimm` until i=100
value
i
0
20
40
60
80
100
0
200
400
600
800
1000
volume surgery dh (5)
quality of surgery (95)
surgical mentoring (43)
surgery budget dh (85)
inappropriate referrals to ch (75)
HRM (57)
inclusion of surgery in pimm (99)

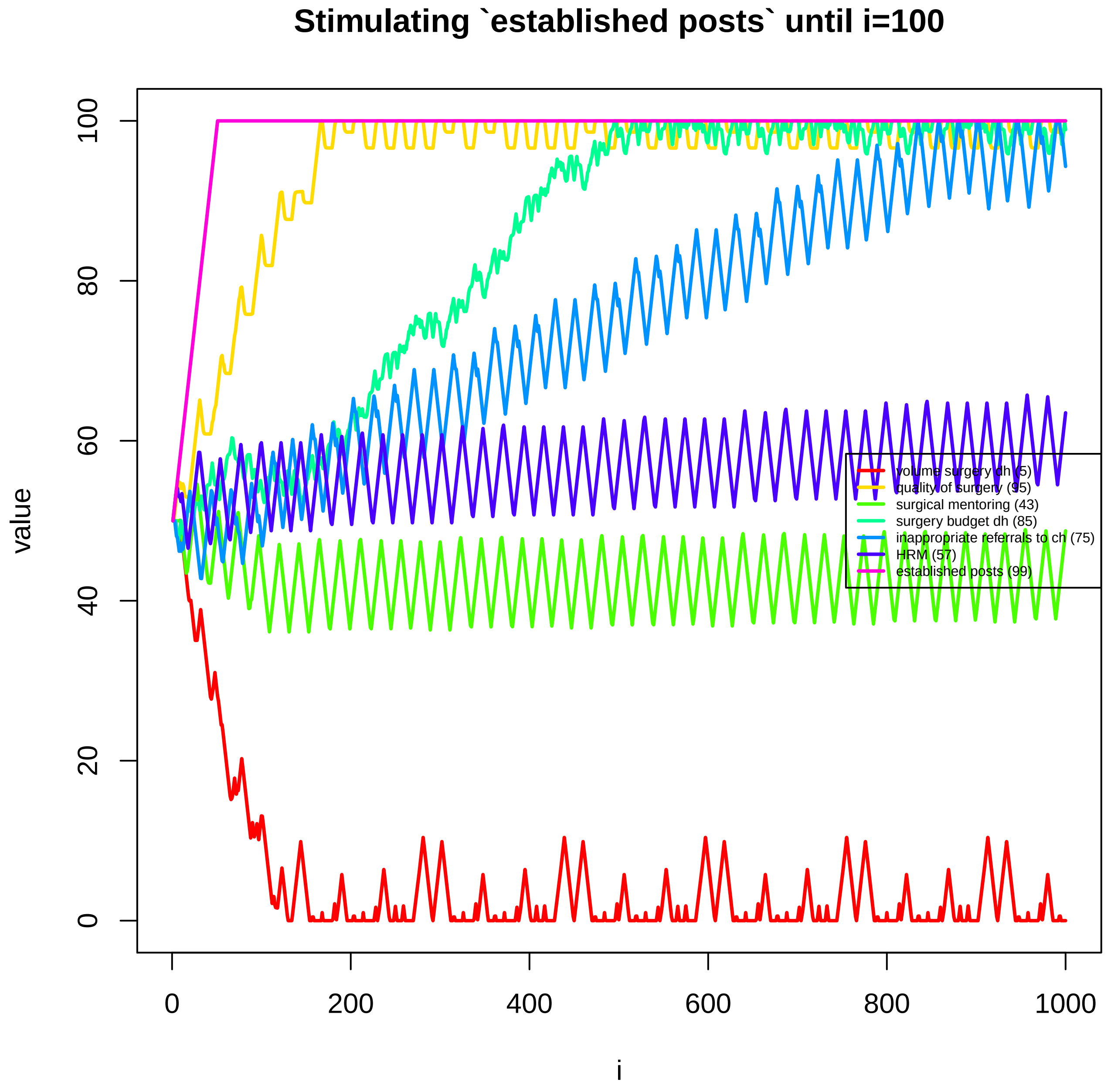

Stimulating `established posts` until i=100
value
i
0
20
40
60
80
100
0
200
400
600
800
1000
volume surgery dh (5)
quality of surgery (95)
surgical mentoring (43)
surgery budget dh (85)
inappropriate referrals to ch (75)
HRM (57)
established posts (99)

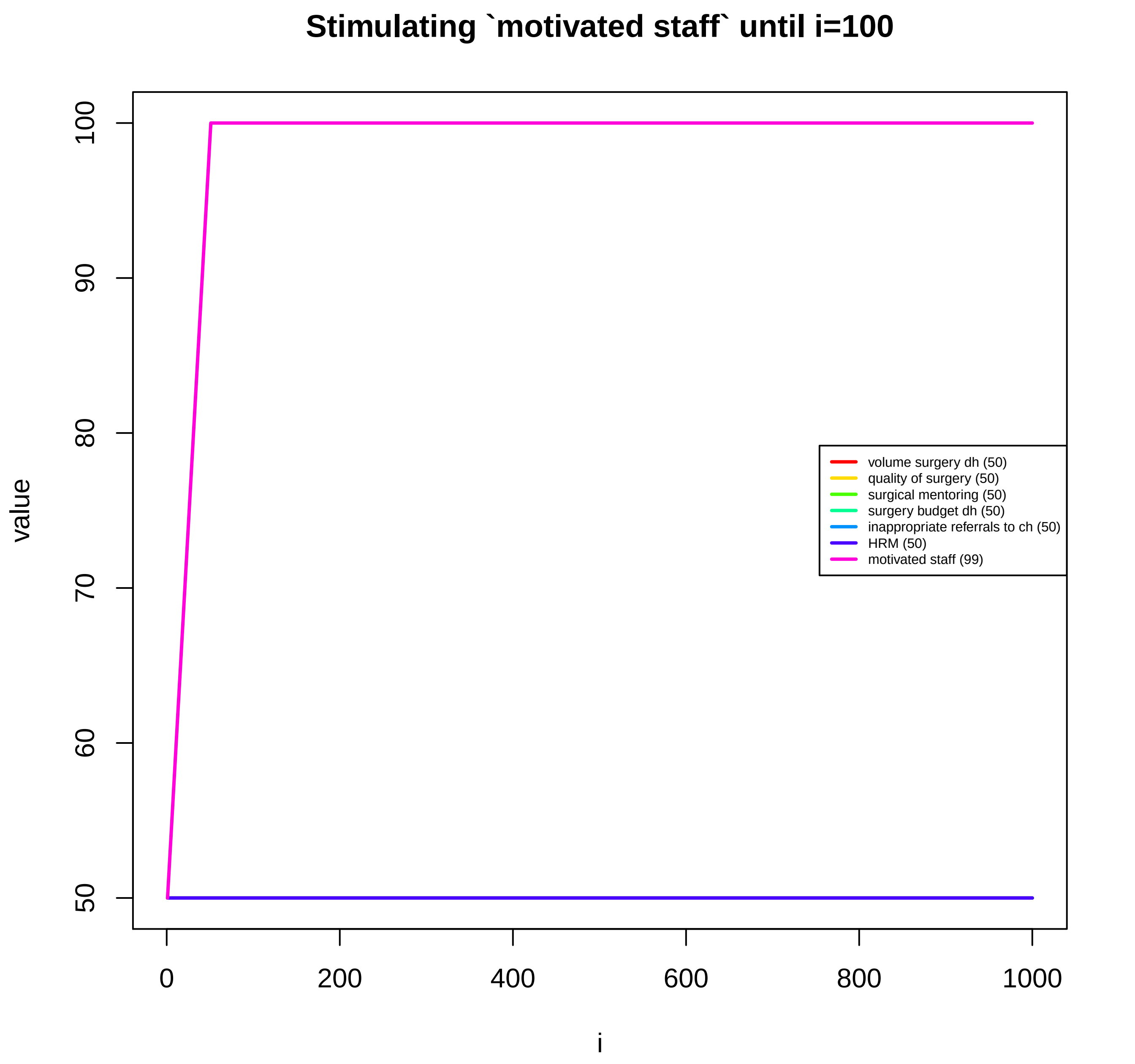
Stimulating `motivated staff` until i=100
value
100
90
80
70
60
50
0
200
400
600
800
1000
i
volume surgery dh (50)
quality of surgery (50)
surgical mentoring (50)
surgery budget dh (50)
inappropriate referrals to ch (50)
HRM (50)
motivated staff (99)

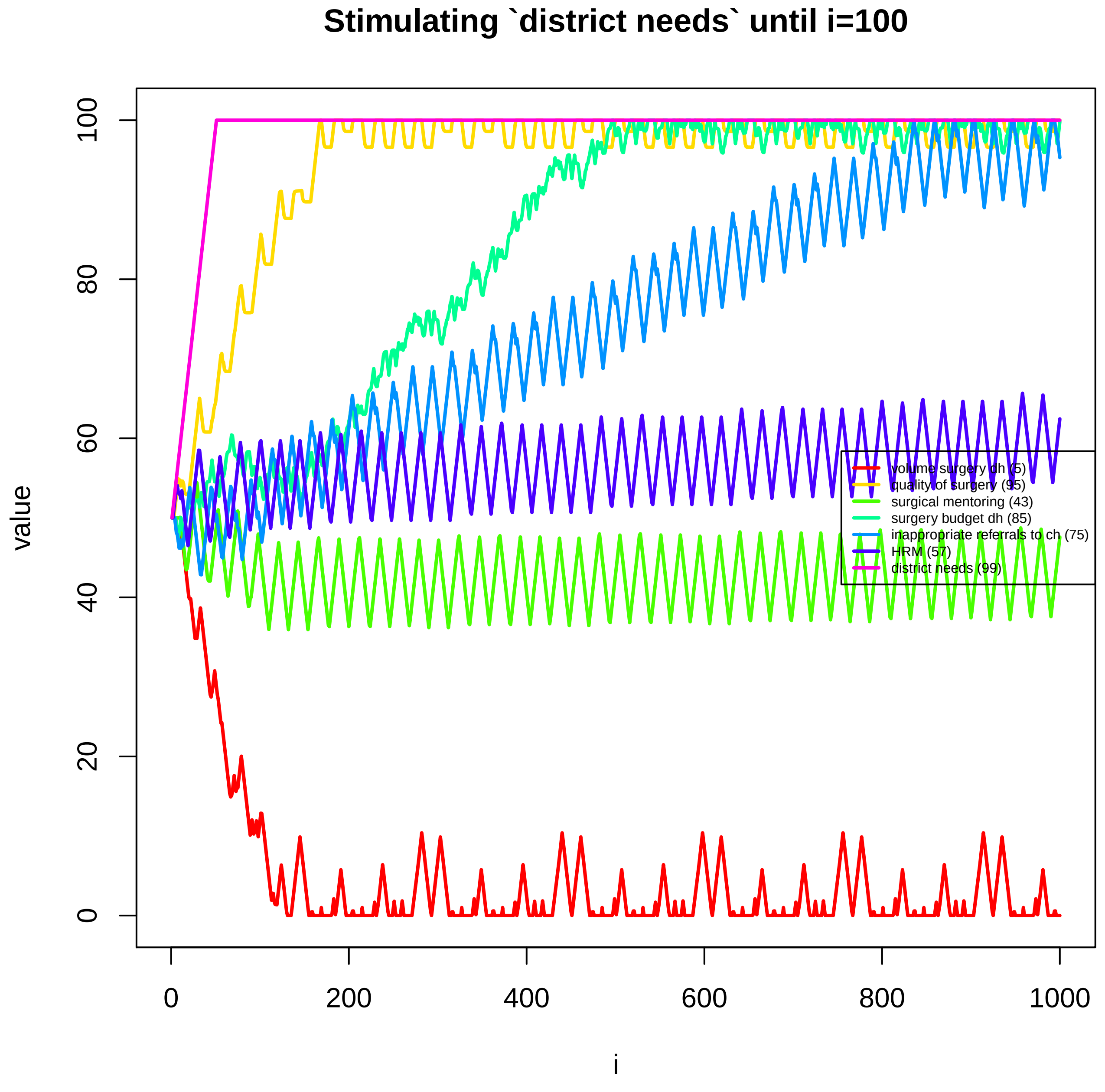
Stimulating `district needs` until i=100
value
i
0
20
40
60
80
100
0
200
400
600
800
1000
volume surgery dh (5)
quality of surgery (95)
surgical mentoring (43)
surgery budget dh (85)
inappropriate referrals to ch (75)
HRM (57)
district needs (99)

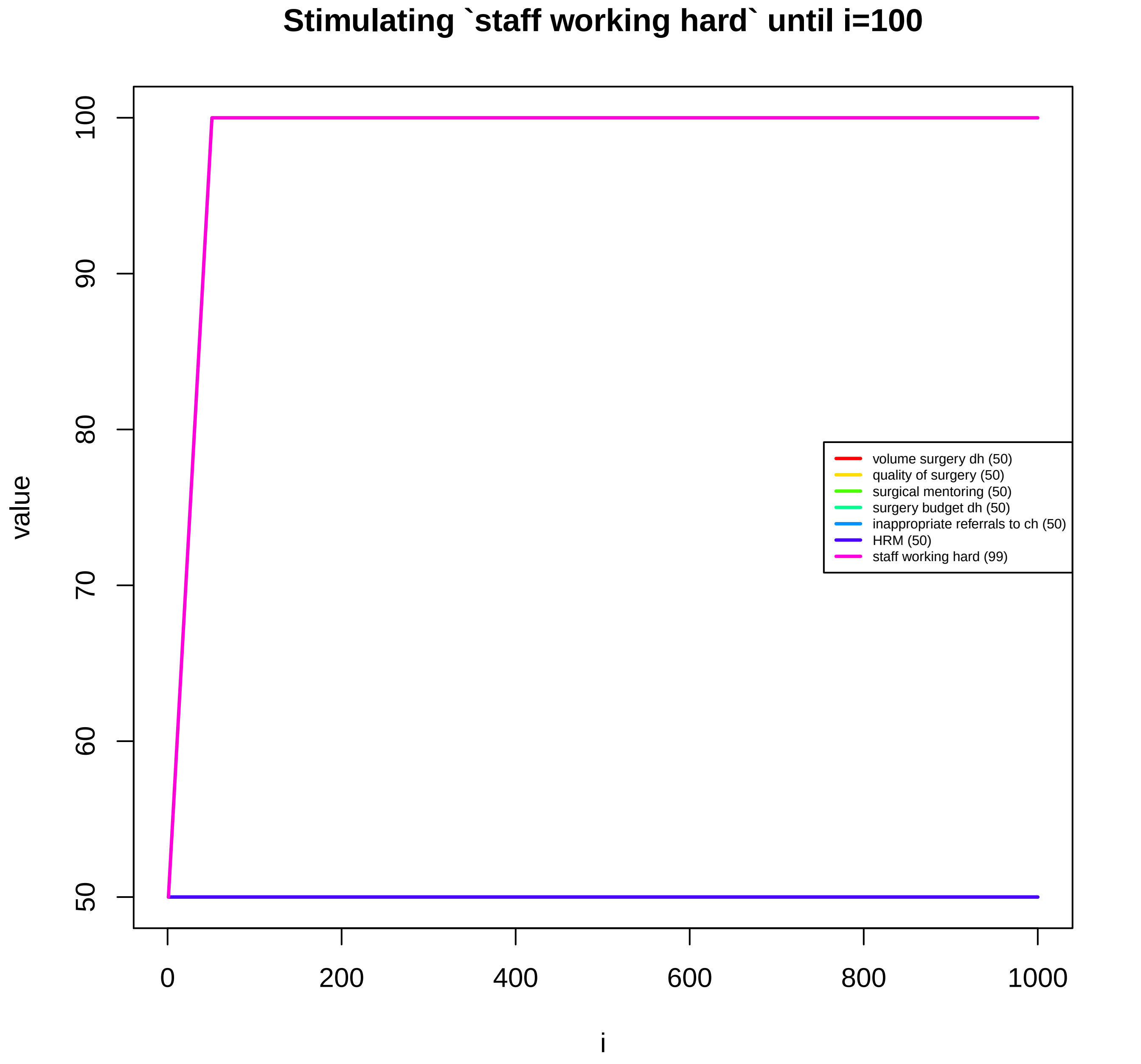

Stimulating `staff working hard` until i=100
value
100
90
80
70
60
50
0
200
400
600
800
1000
i
volume surgery dh (50)
quality of surgery (50)
surgical mentoring (50)
surgery budget dh (50)
inappropriate referrals to ch (50)
HRM (50)
staff working hard (99)

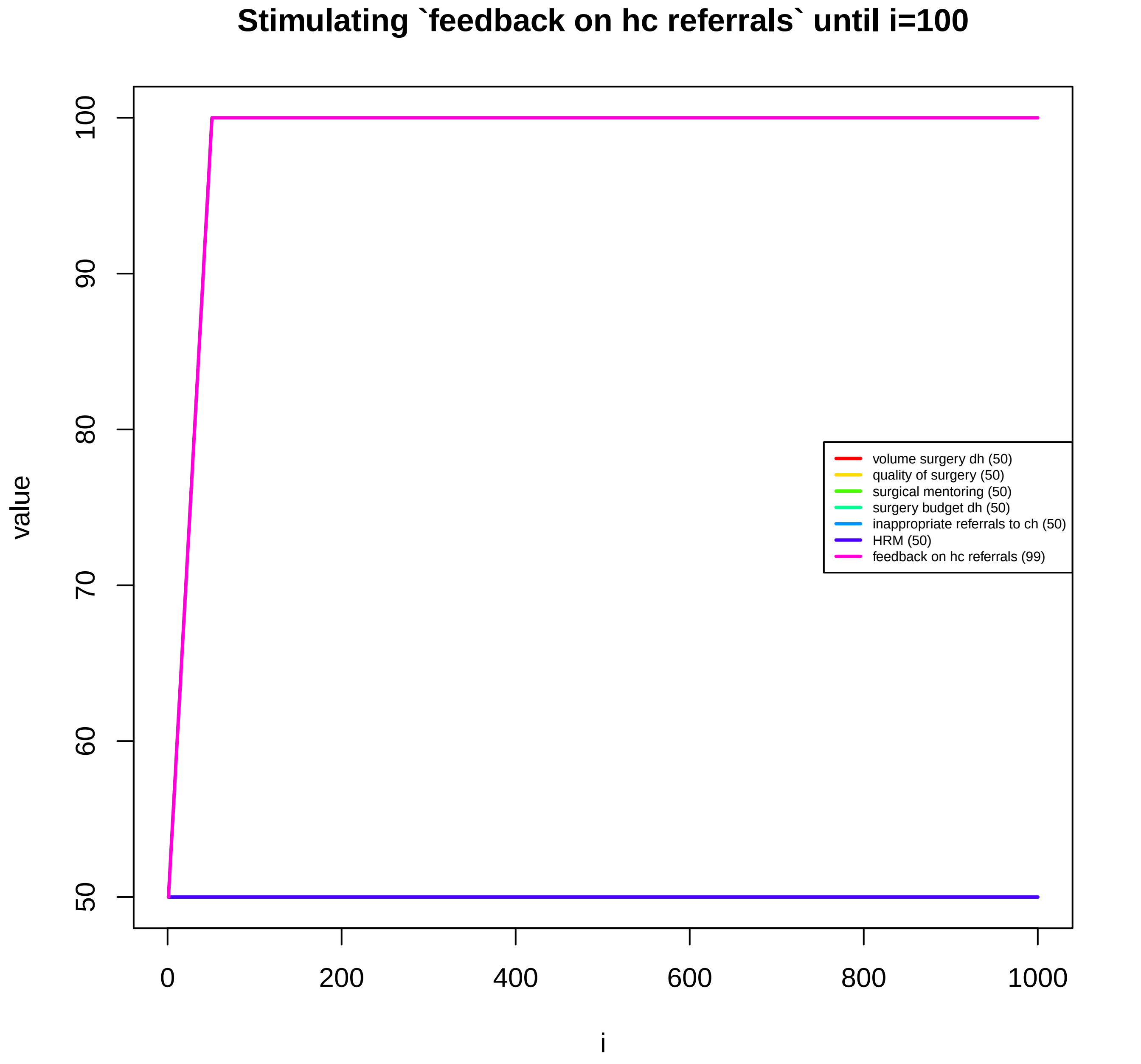
Stimulating `feedback on hc referrals` until i=100
value
100
90
80
70
60
50
0
200
400
600
800
1000
i
volume surgery dh (50)
quality of surgery (50)
surgical mentoring (50)
surgery budget dh (50)
inappropriate referrals to ch (50)
HRM (50)
feedback on hc referrals (99)

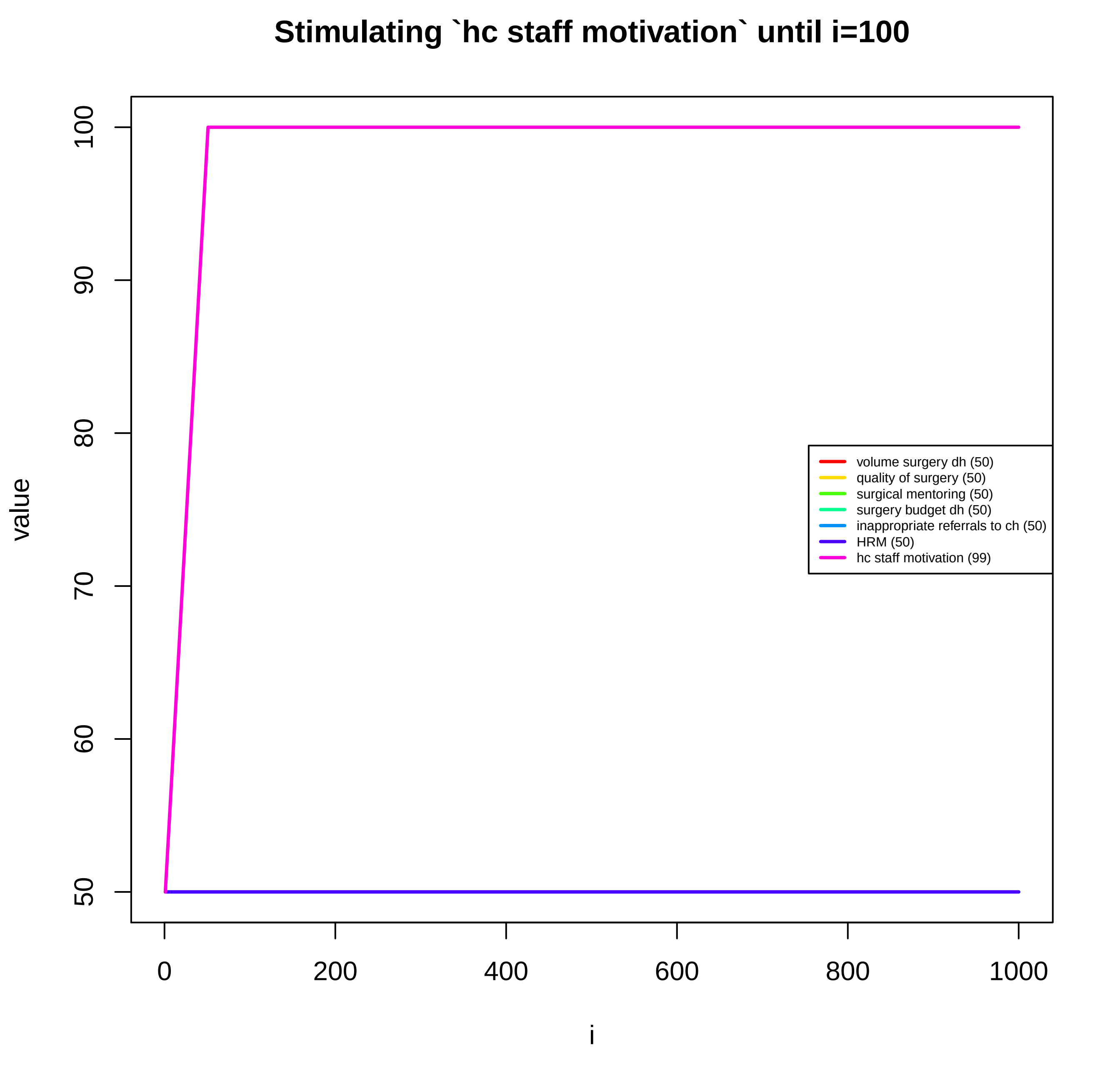

Stimulating `hc staff motivation` until i=100
value
i
100
90
80
70
60
50
0
200
400
600
800
1000
volume surgery dh (50)
quality of surgery (50)
surgical mentoring (50)
surgery budget dh (50)
inappropriate referrals to ch (50)
HRM (50)
hc staff motivation (99)

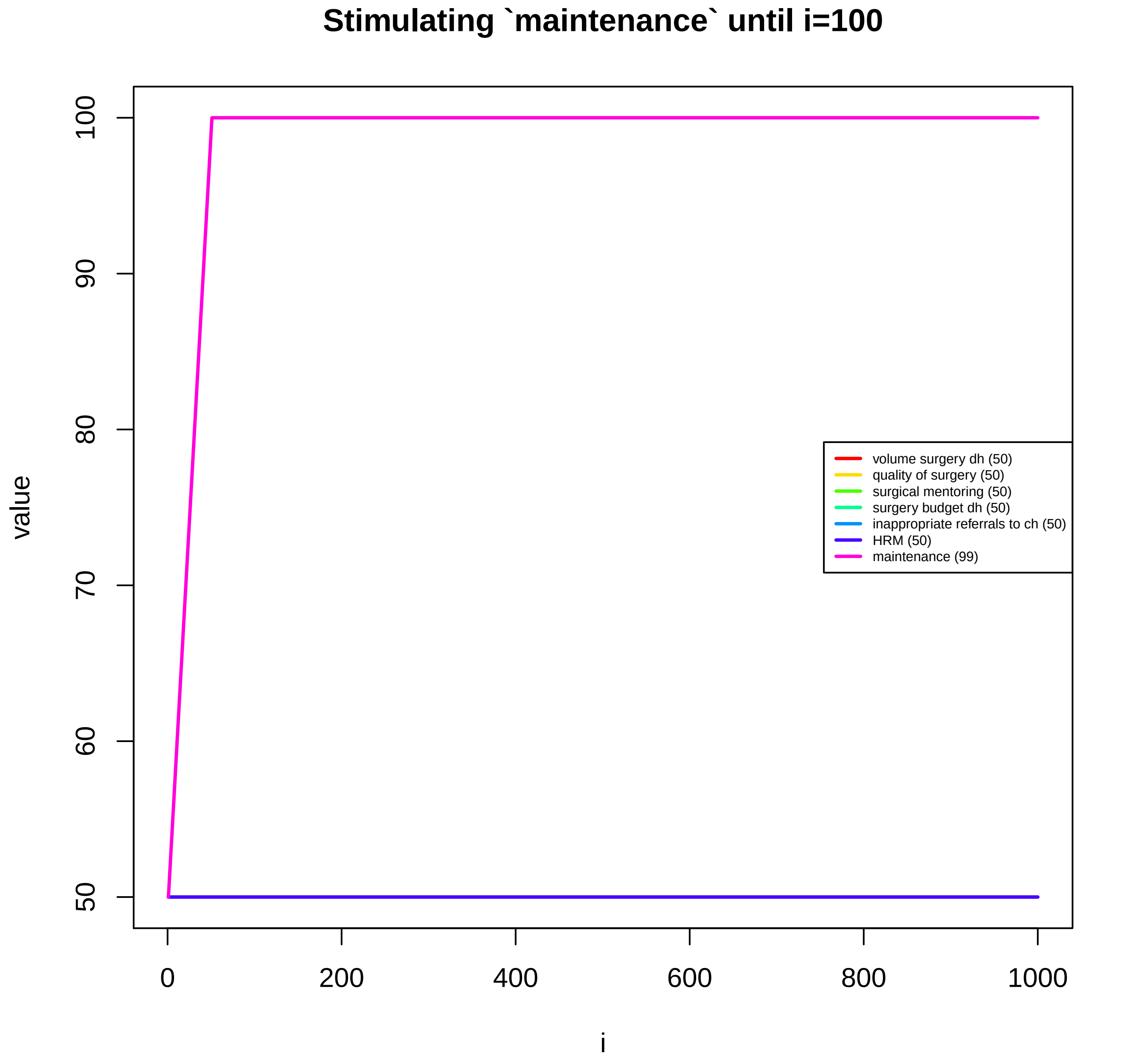

Stimulating `maintenance` until i=100
value
100
90
80
70
60
50
0
200
400
600
800
1000
i
volume surgery dh (50)
quality of surgery (50)
surgical mentoring (50)
surgery budget dh (50)
inappropriate referrals to ch (50)
HRM (50)
maintenance (99)

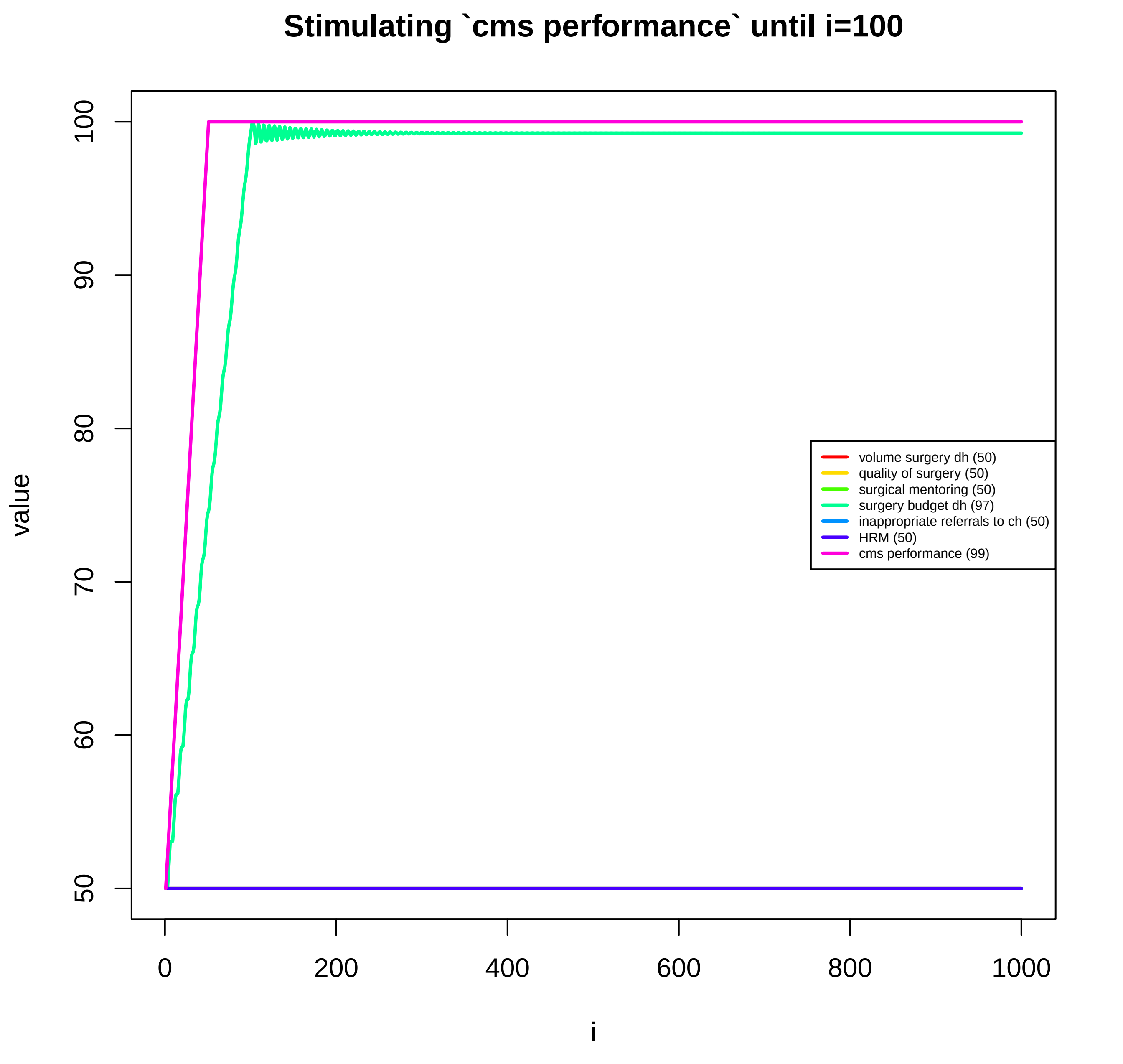

Stimulating `cms performance` until i=100
value
i
100
90
80
70
60
50
0
200
400
600
800
1000
volume surgery dh (50)
quality of surgery (50)
surgical mentoring (50)
surgery budget dh (97)
inappropriate referrals to ch (50)
HRM (50)
cms performance (99)

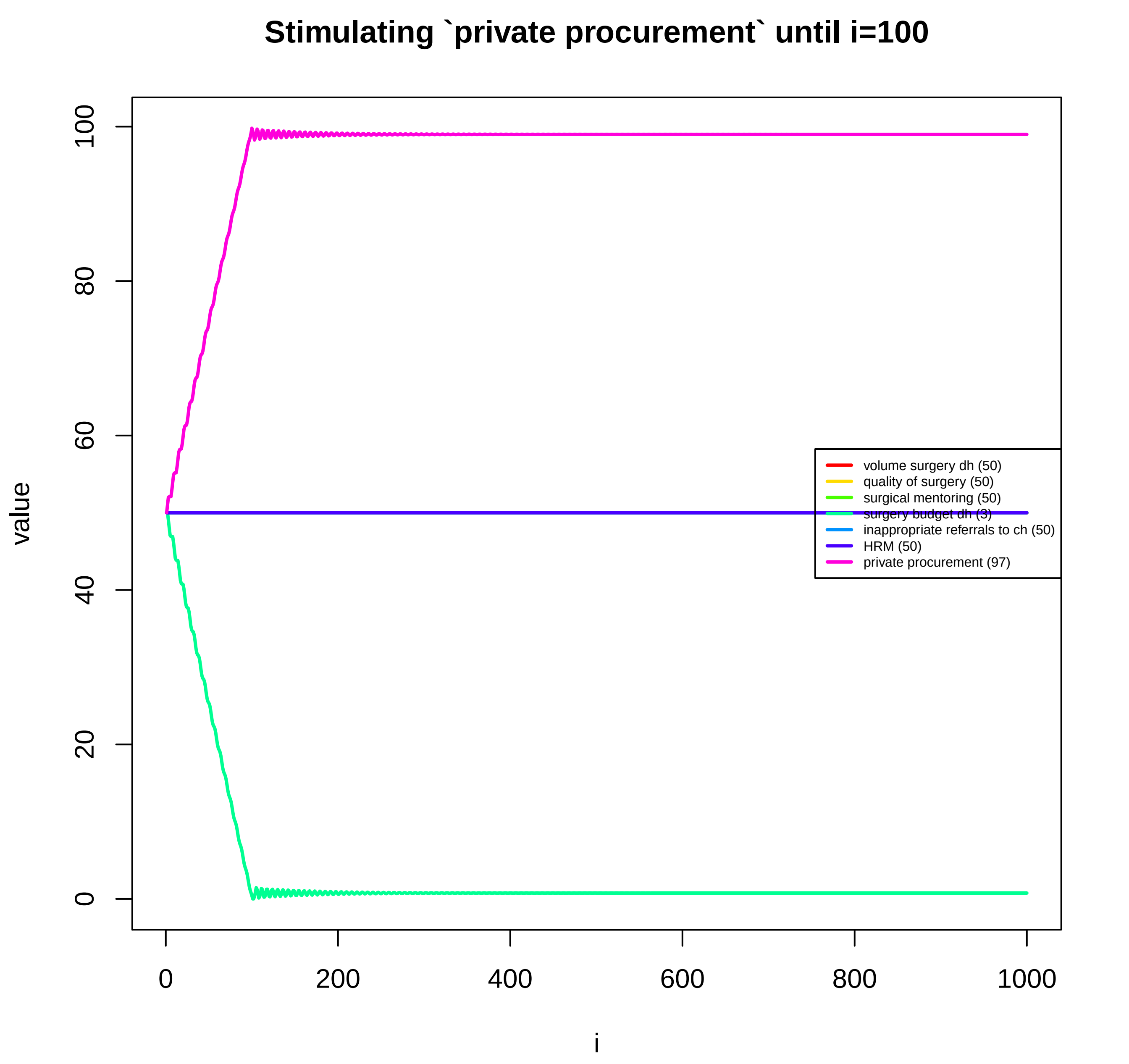

Stimulating `private procurement` until i=100
value
i
0
20
40
60
80
100
0
200
400
600
800
1000
volume surgery dh (50)
quality of surgery (50)
surgical mentoring (50)
surgery budget dh (3)
inappropriate referrals to ch (50)
HRM (50)
private procurement (97)

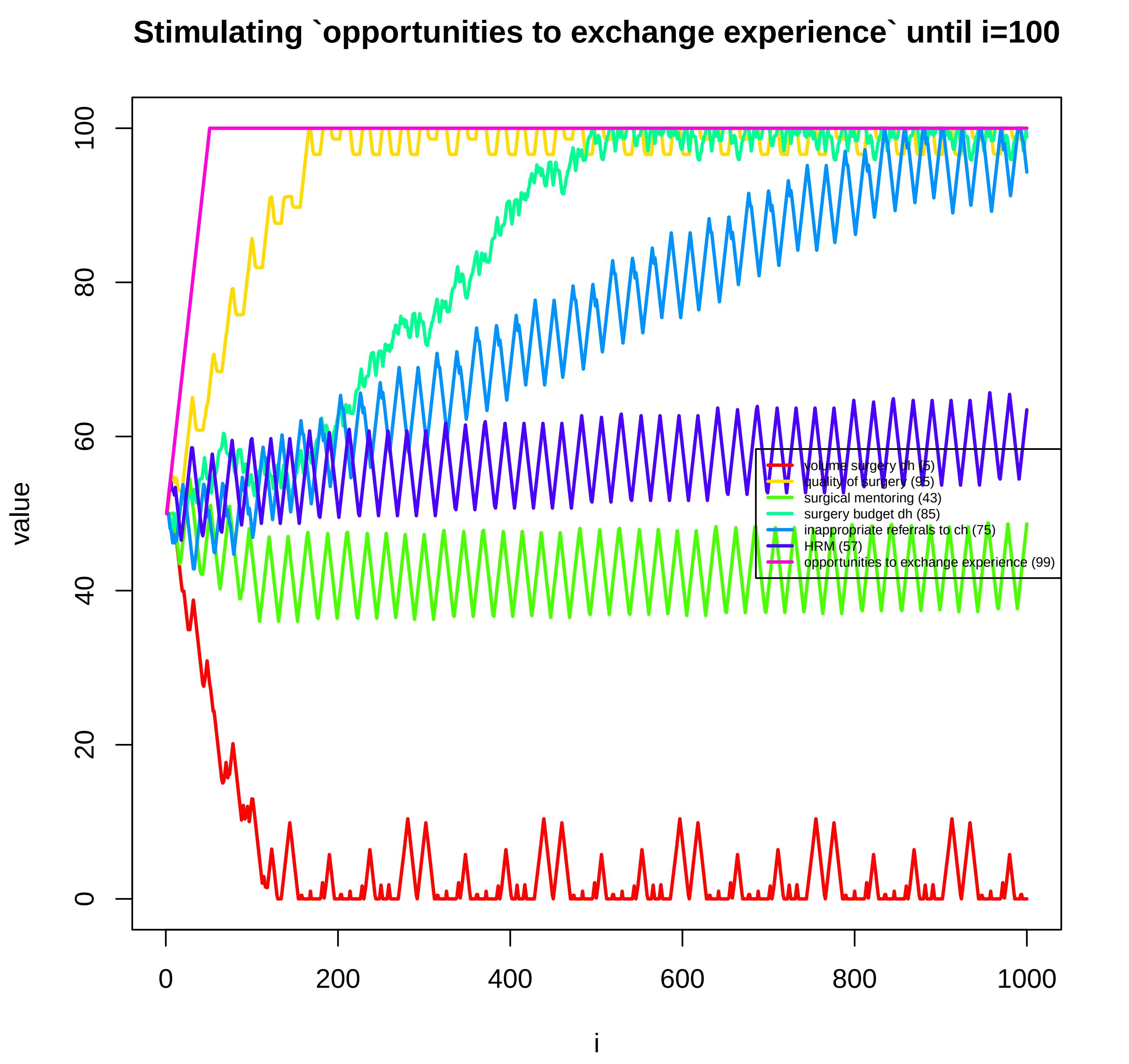

Stimulating `opportunities to exchange experience` until i=100
value
i
0
20
40
60
80
100
0
200
400
600
800
1000
volume surgery dh (5)
quality of surgery (95)
surgical mentoring (43)
surgery budget dh (85)
inappropriate referrals to ch (75)
HRM (57)
opportunities to exchange experience (99)

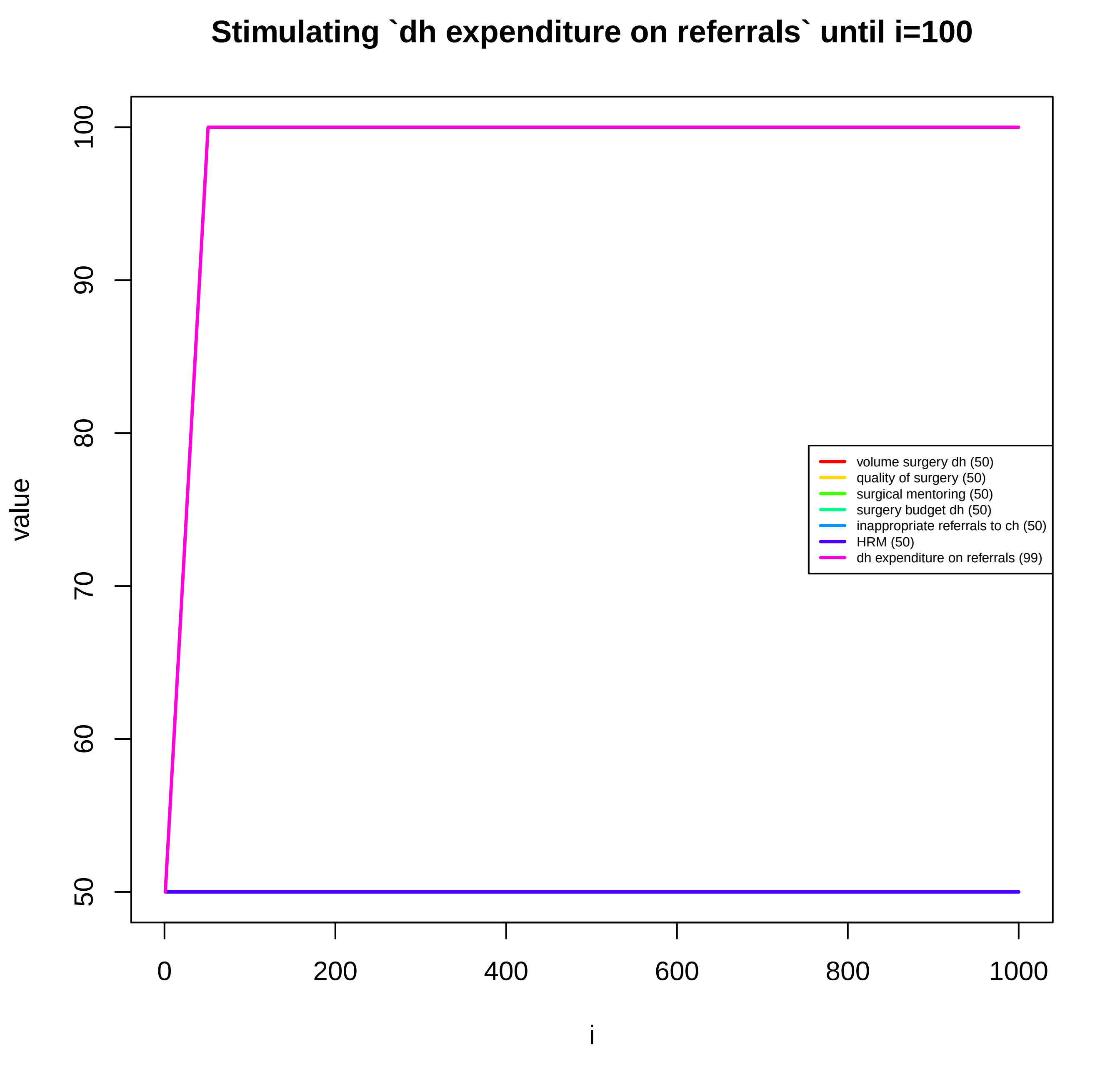

Stimulating `dh expenditure on referrals` until i=100
value
100
90
80
70
60
50
0
200
400
600
800
1000
i
volume surgery dh (50)
quality of surgery (50)
surgical mentoring (50)
surgery budget dh (50)
inappropriate referrals to ch (50)
HRM (50)
dh expenditure on referrals (99)

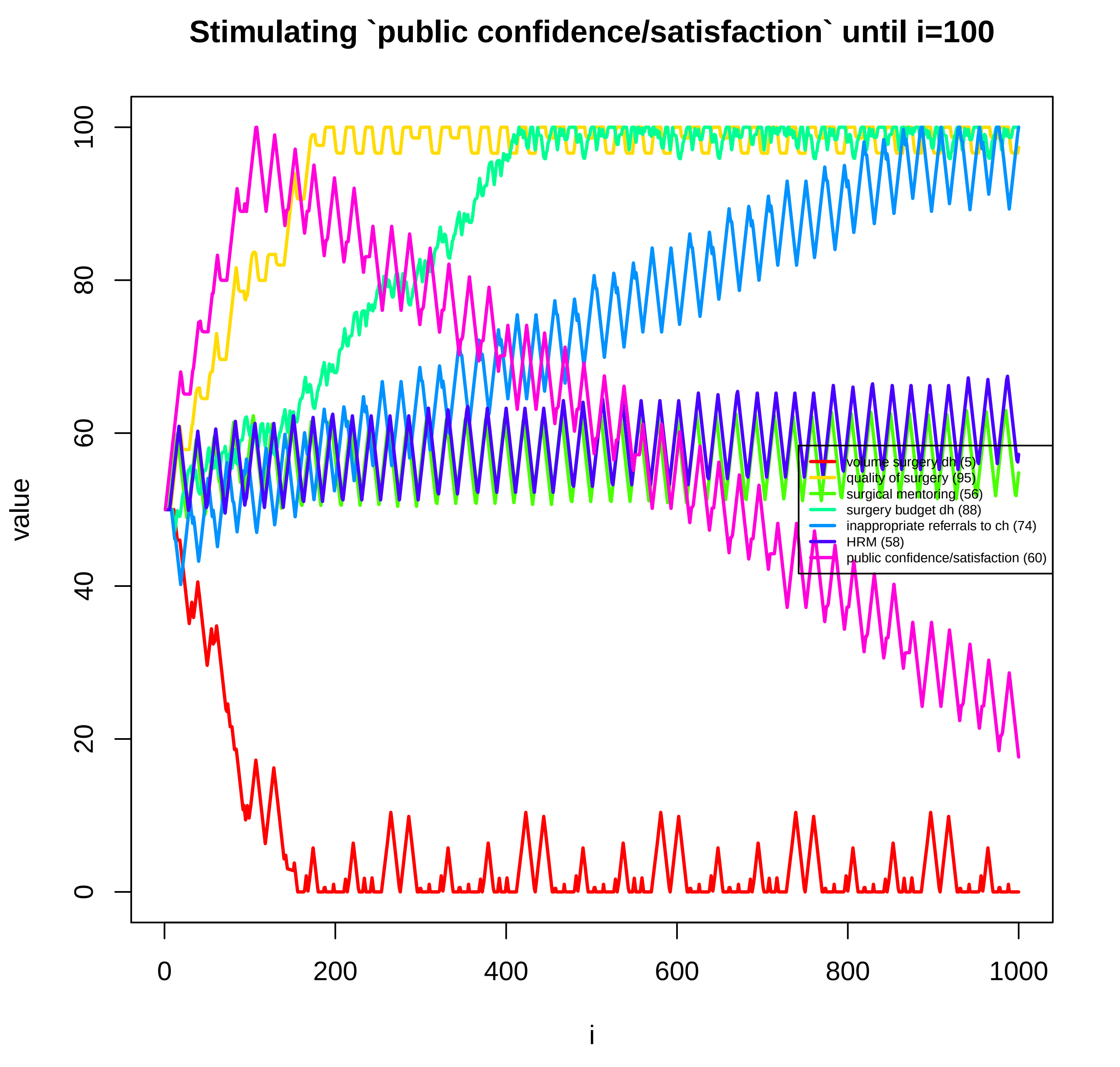
Stimulating `public confidence/satisfaction` until i=100
value
i
0
20
40
60
80
100
0
200
400
600
800
1000
volume surgery dh (5)
quality of surgery (95)
surgical mentoring (56)
surgery budget dh (88)
inappropriate referrals to ch (74)
HRM (58)
public confidence/satisfaction (60)

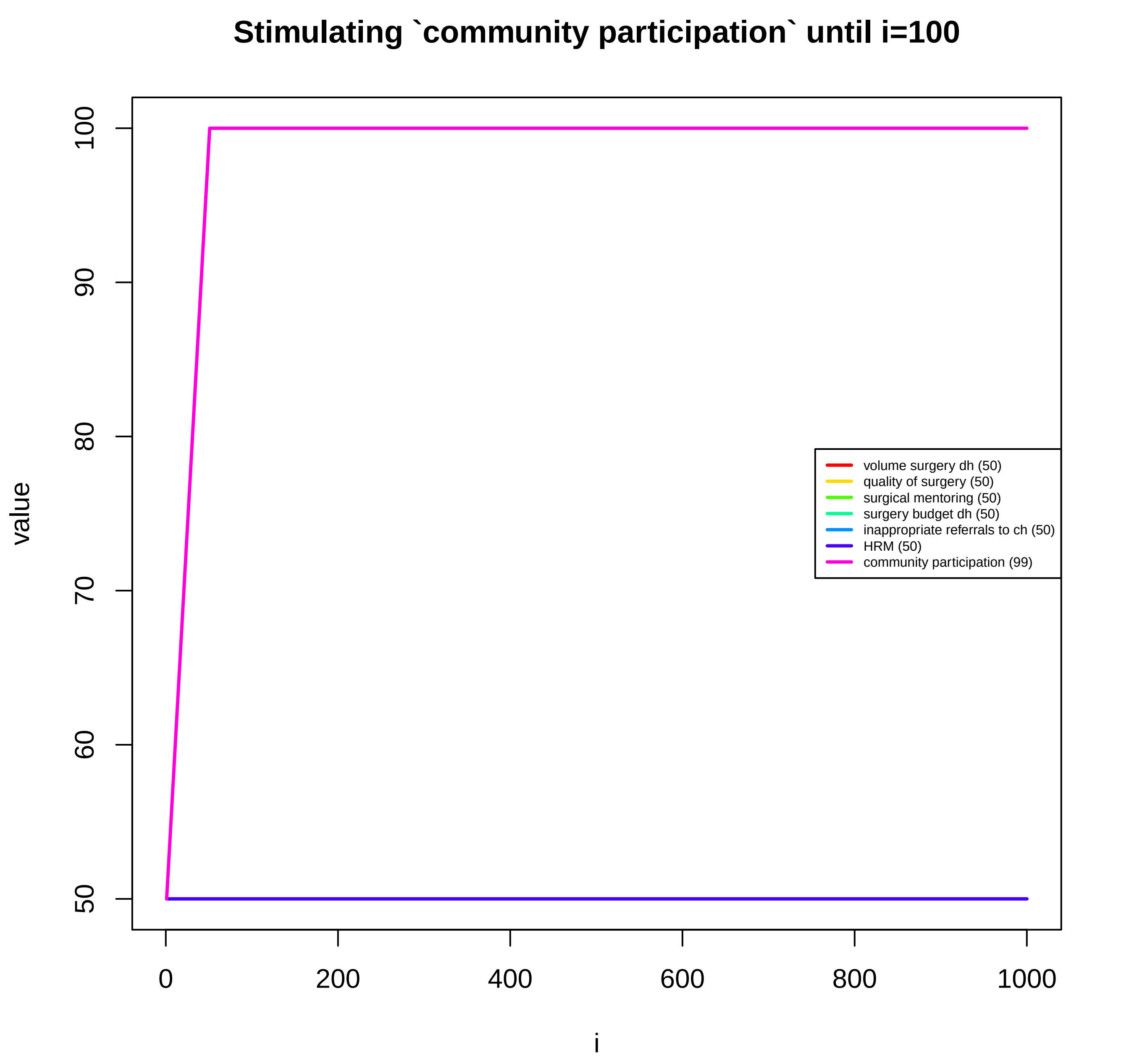
Stimulating `community participation` until i=100
value
i
100
90
80
70
60
50
0
200
400
600
800
1000
volume surgery dh (50)
quality of surgery (50)
surgical mentoring (50)
surgery budget dh (50)
inappropriate referrals to ch (50)
HRM (50)
community participation (99)

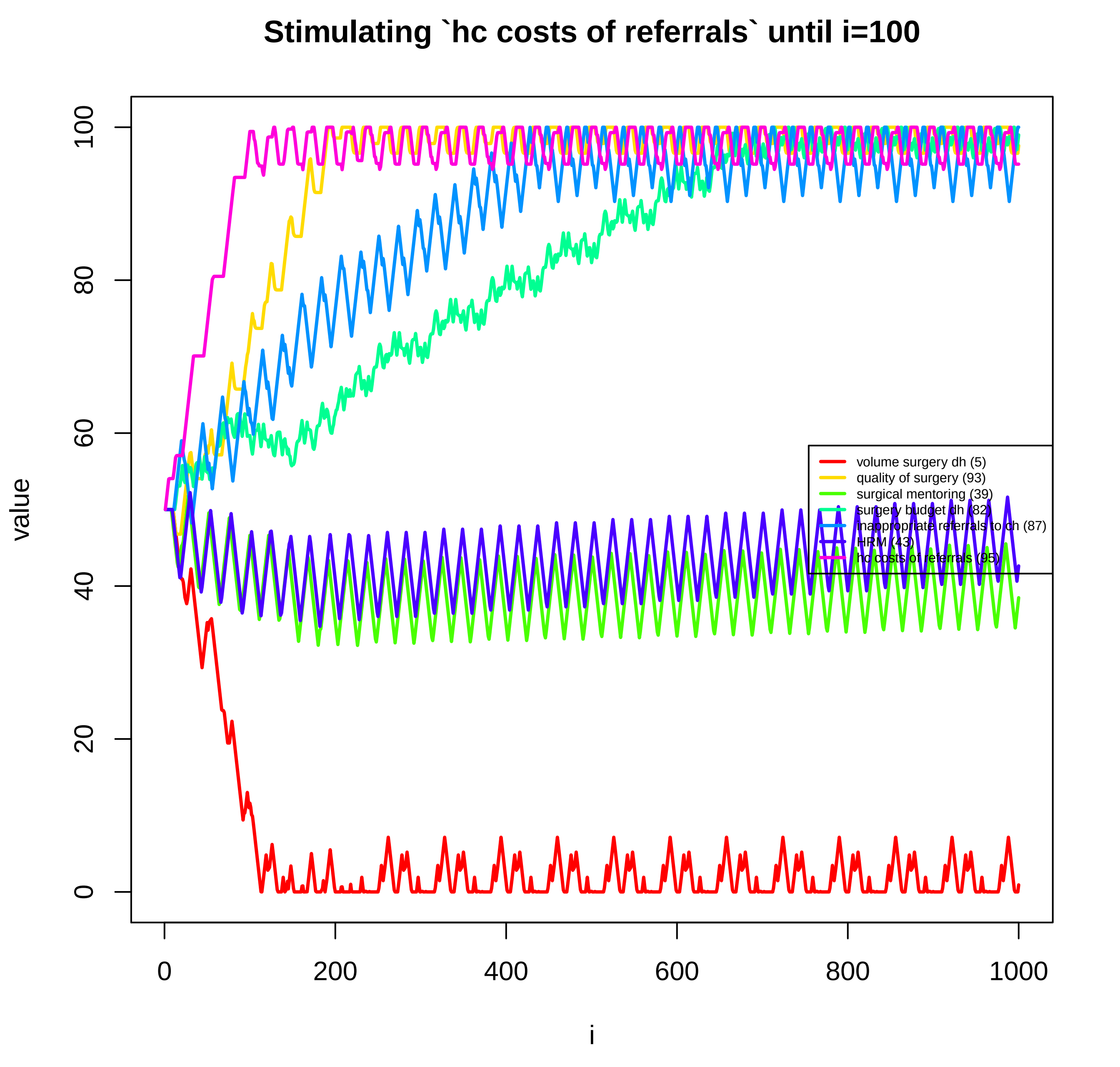
Stimulating `hc costs of referrals` until i=100
value
i
0
20
40
60
80
100
0
200
400
600
800
1000
volume surgery dh (5)
quality of surgery (93)
surgical mentoring (39)
surgery budget dh (82)
inappropriate referrals to dh (87)
HRM (43)
hc costs of referrals (95)

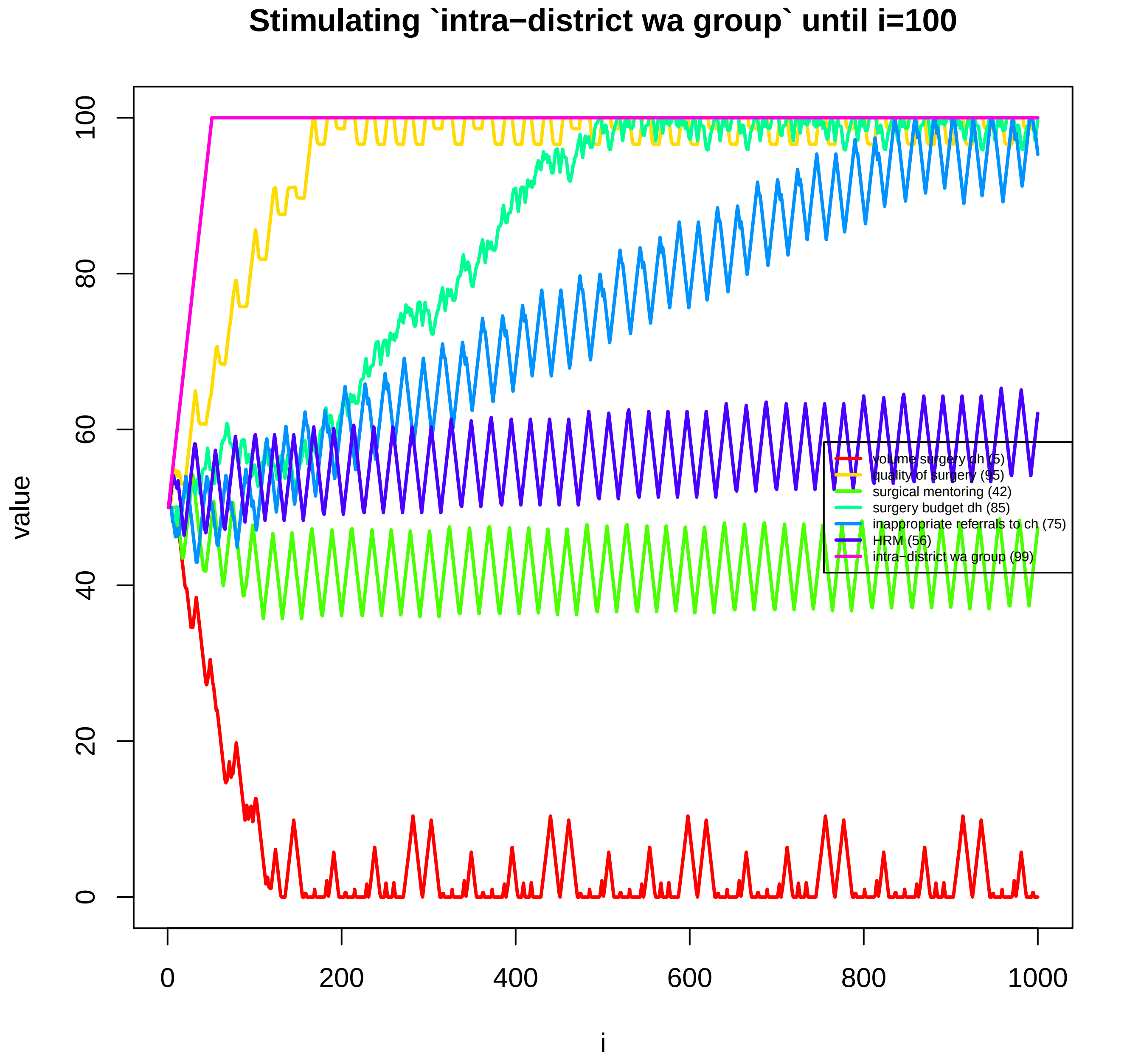

Stimulating `intra−district wa group` until i=100
value
i
0
20
40
60
80
100
0
200
400
600
800
1000
volume surgery dh (5)
quality of surgery (95)
surgical mentoring (42)
surgery budget dh (85)
inappropriate referrals to ch (75)
HRM (56)
intra−district wa group (99)

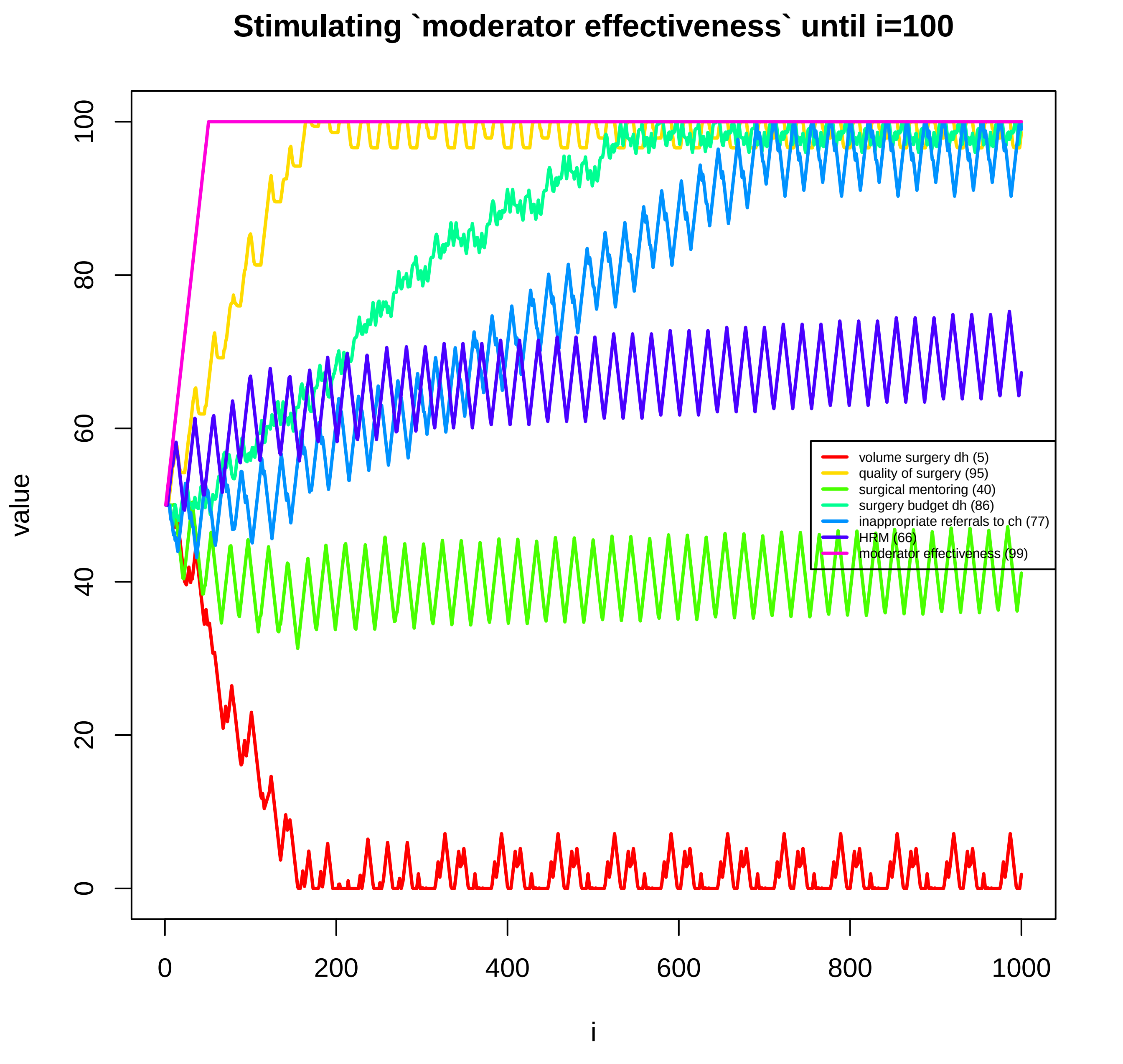

Stimulating `moderator effectiveness` until i=100
value
i
0
20
40
60
80
100
0
200
400
600
800
1000
volume surgery dh (5)
quality of surgery (95)
surgical mentoring (40)
surgery budget dh (86)
inappropriate referrals to ch (77)
HRM (66)
moderator effectiveness (99)

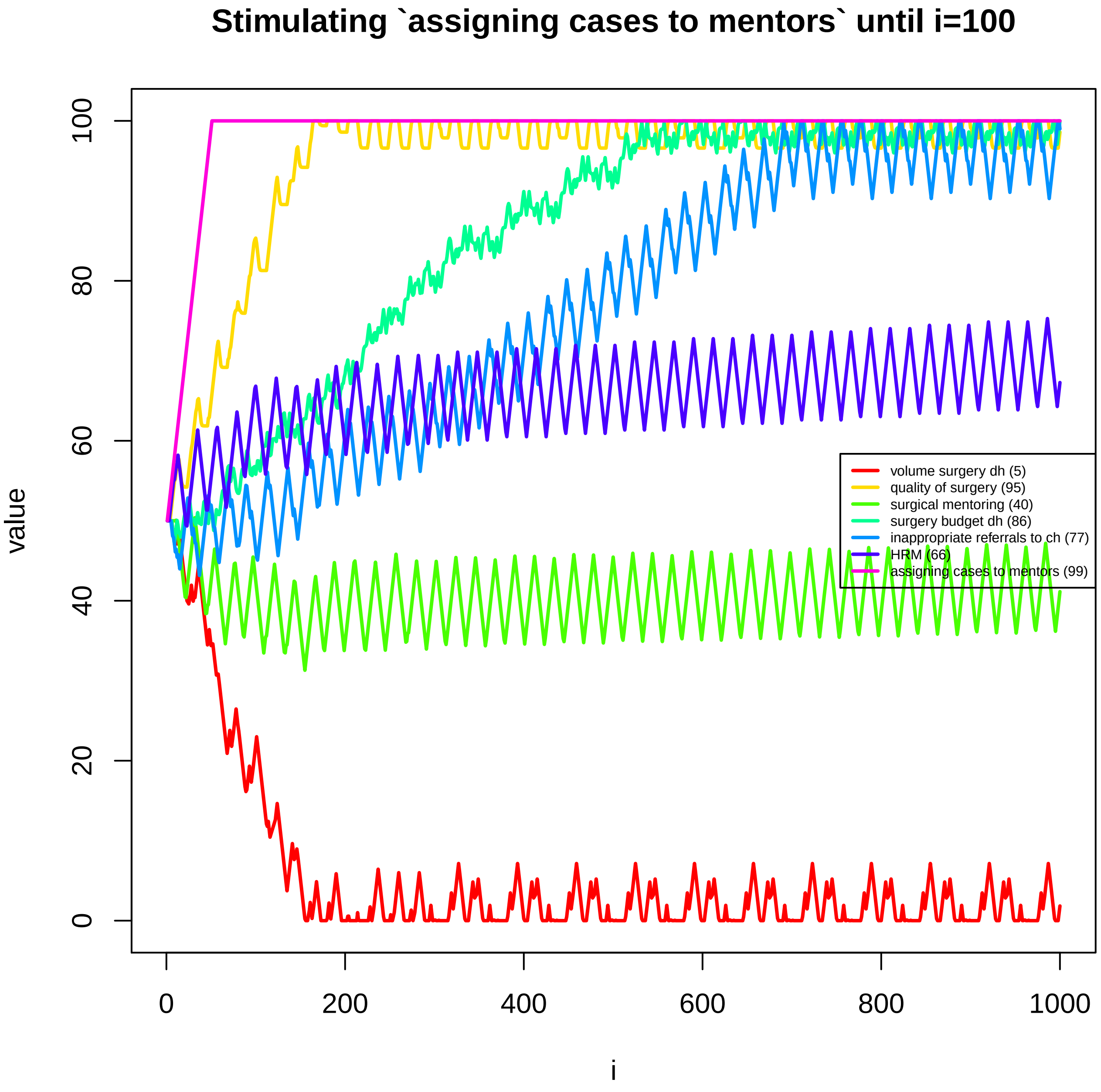

Stimulating `assigning cases to mentors` until i=100
value
i
0
20
40
60
80
100
0
200
400
600
800
1000
volume surgery dh (5)
quality of surgery (95)
surgical mentoring (40)
surgery budget dh (86)
inappropriate referrals to ch (77)
HRM (66)
assigning cases to mentors (99)

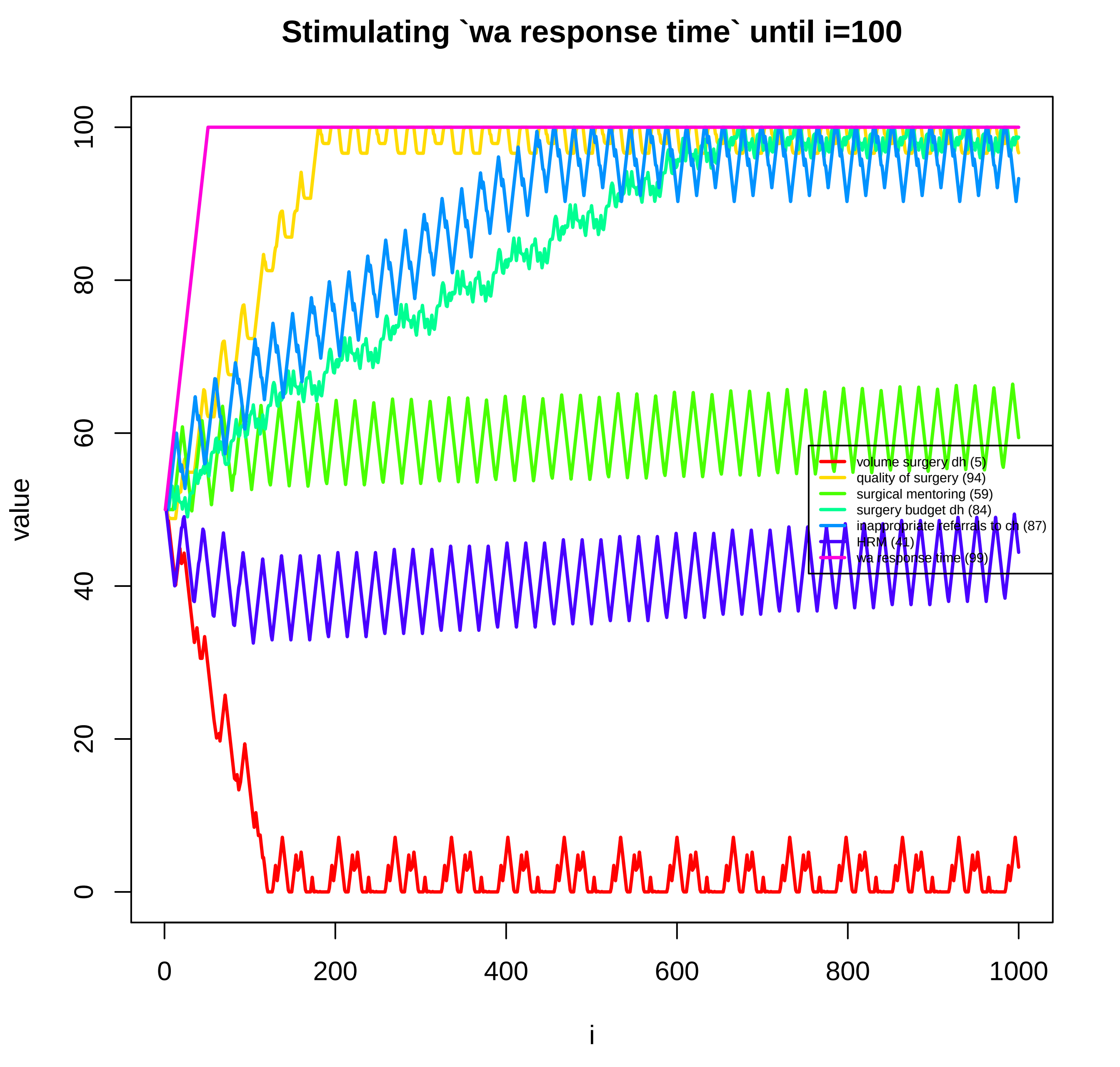

Stimulating `wa response time` until i=100
value
i
0
20
40
60
80
100
0
200
400
600
800
1000
volume surgery dh (5)
quality of surgery (94)
surgical mentoring (59)
surgery budget dh (84)
inappropriate referrals to ch (87)
HRM (41)
wa response time (99)

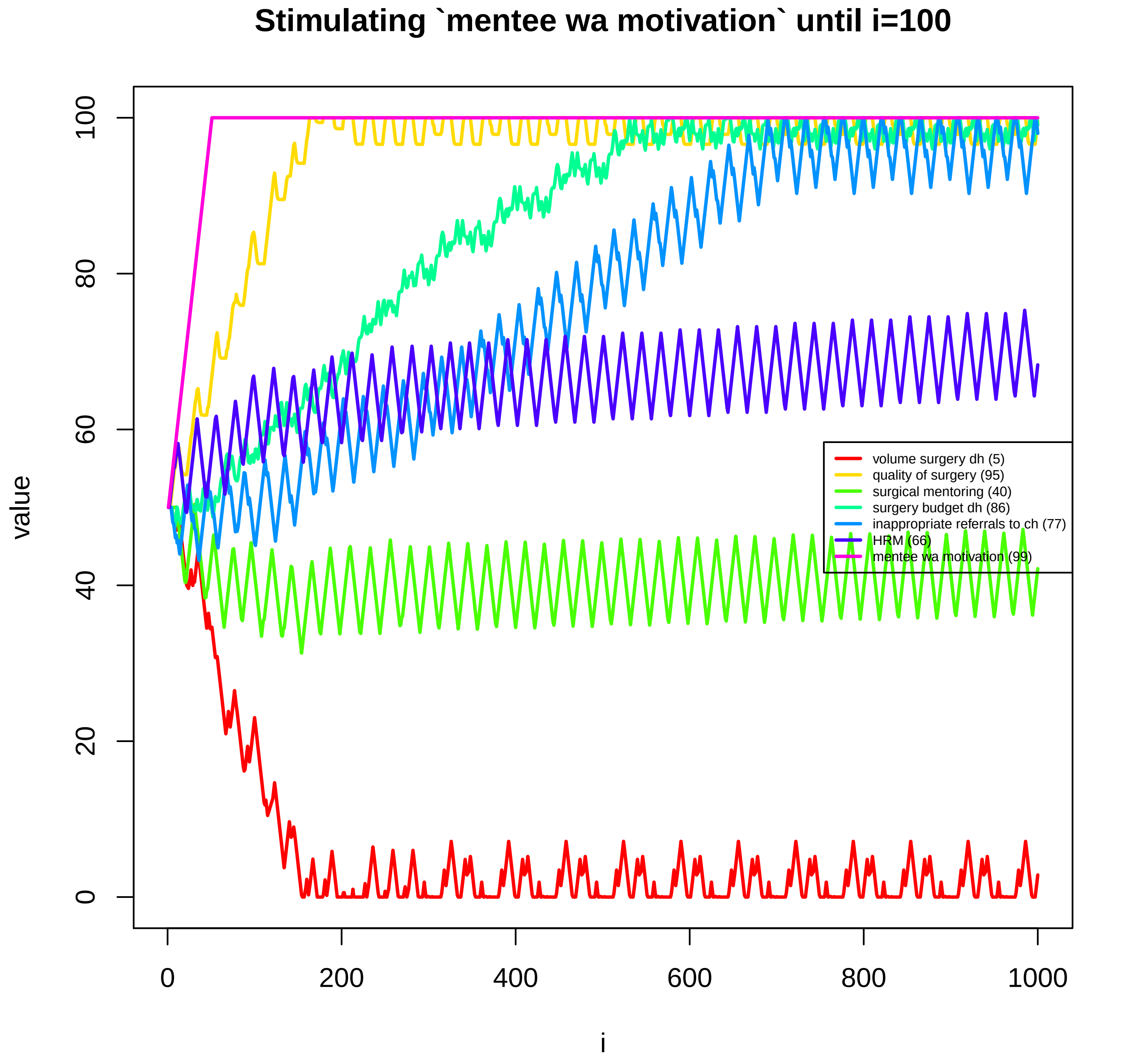
Stimulating `mentee wa motivation` until i=100
value
i
0
20
40
60
80
100
0
200
400
600
800
1000
volume surgery dh (5)
quality of surgery (95)
surgical mentoring (40)
surgery budget dh (86)
inappropriate referrals to ch (77)
HRM (66)
mentee wa motivation (99)

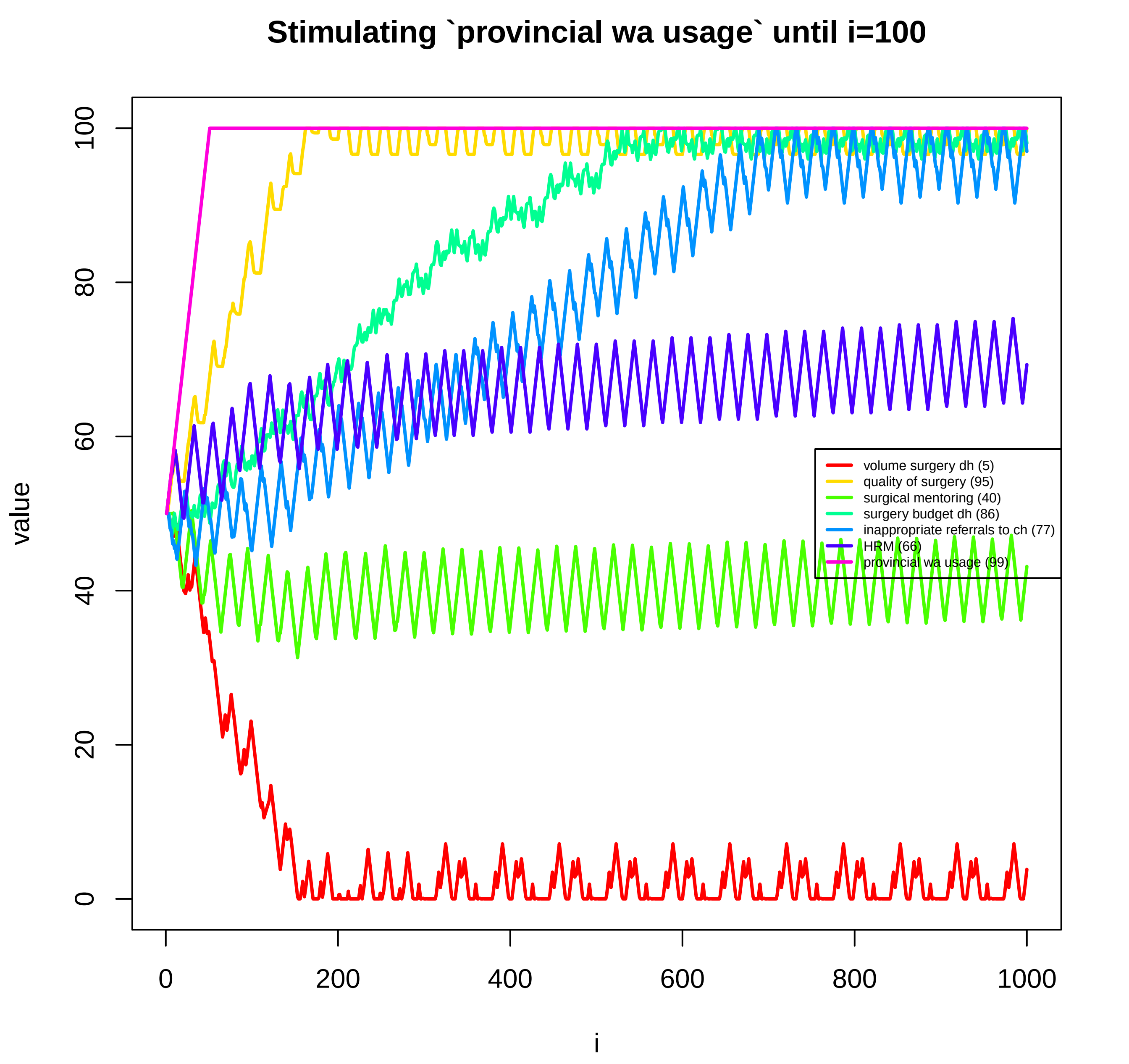

Stimulating `provincial wa usage` until i=100
value
i
0
20
40
60
80
100
0
200
400
600
800
1000
volume surgery dh (5)
quality of surgery (95)
surgical mentoring (40)
surgery budget dh (86)
inappropriate referrals to ch (77)
HRM (66)
provincial wa usage (99)

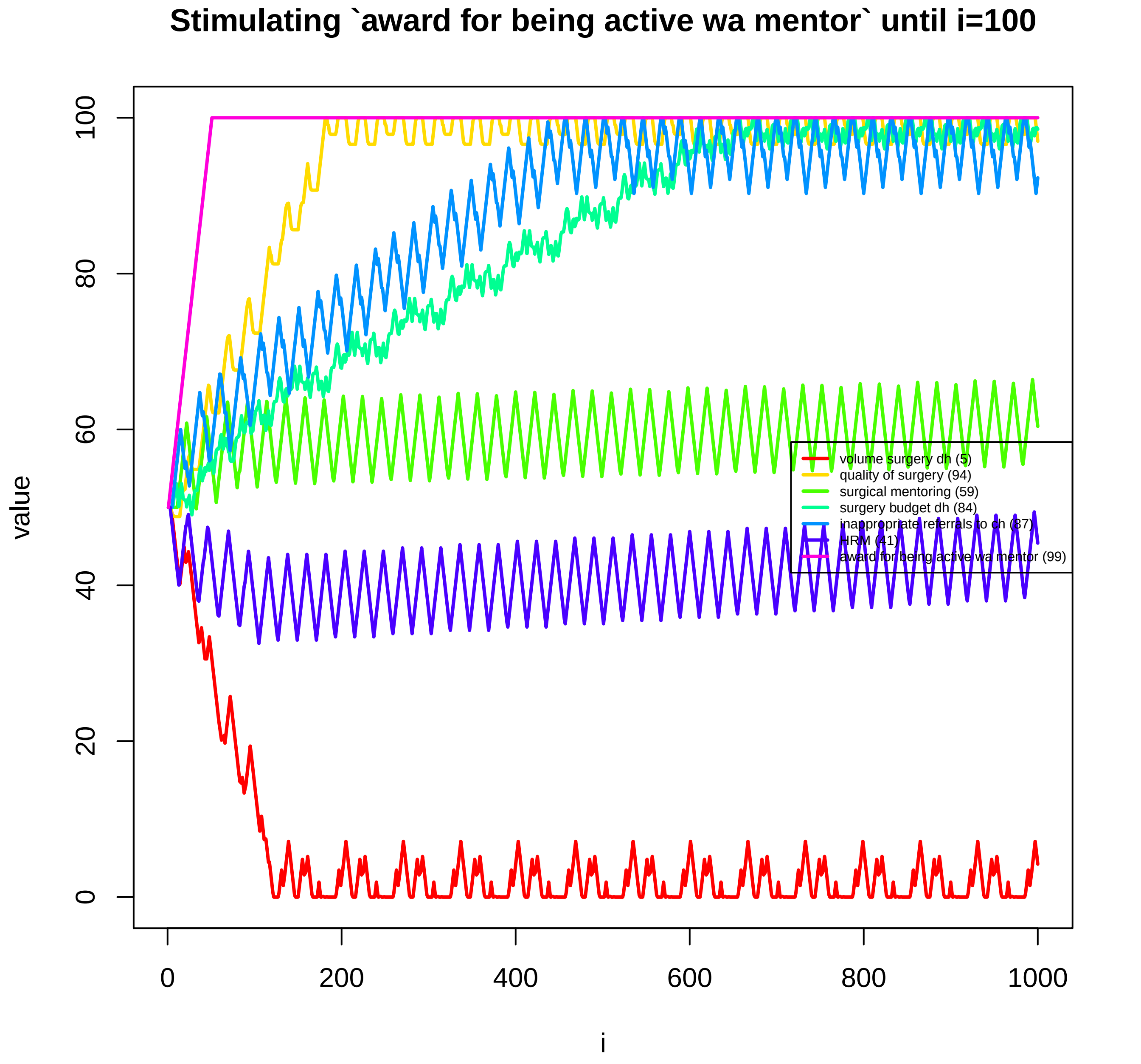
Stimulating `award for being active wa mentor` until i=100
value
i
0
20
40
60
80
100
0
200
400
600
800
1000
volume surgery dh (5)
quality of surgery (94)
surgical mentoring (59)
surgery budget dh (84)
inappropriate referrals to ch (87)
HRM (41)
award for being active wa mentor (99)

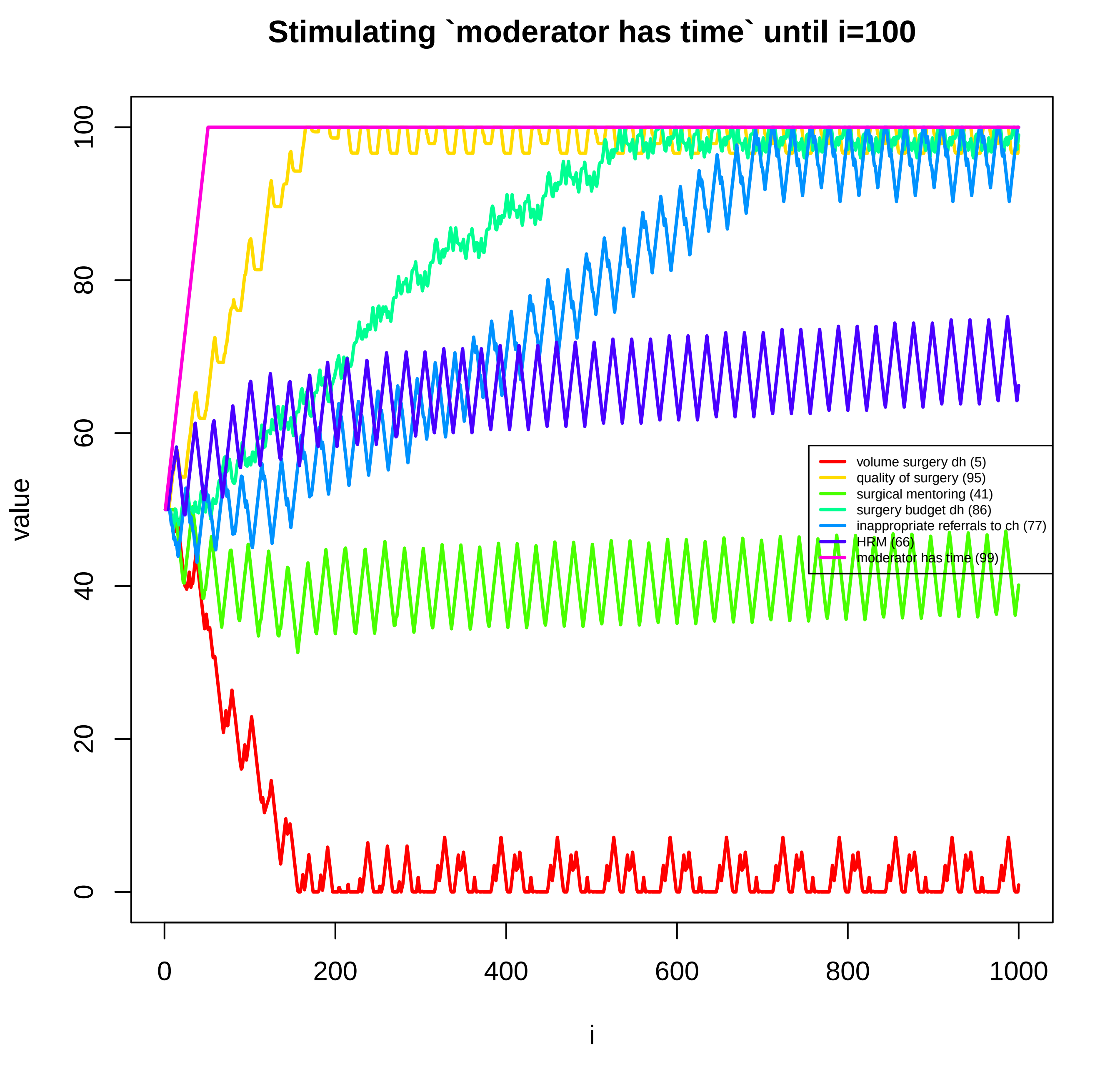
Stimulating `moderator has time` until i=100
value
i
0
20
40
60
80
100
0
200
400
600
800
1000
volume surgery dh (5)
quality of surgery (95)
surgical mentoring (41)
surgery budget dh (86)
inappropriate referrals to ch (77)
HRM (66)
moderator has time (99)

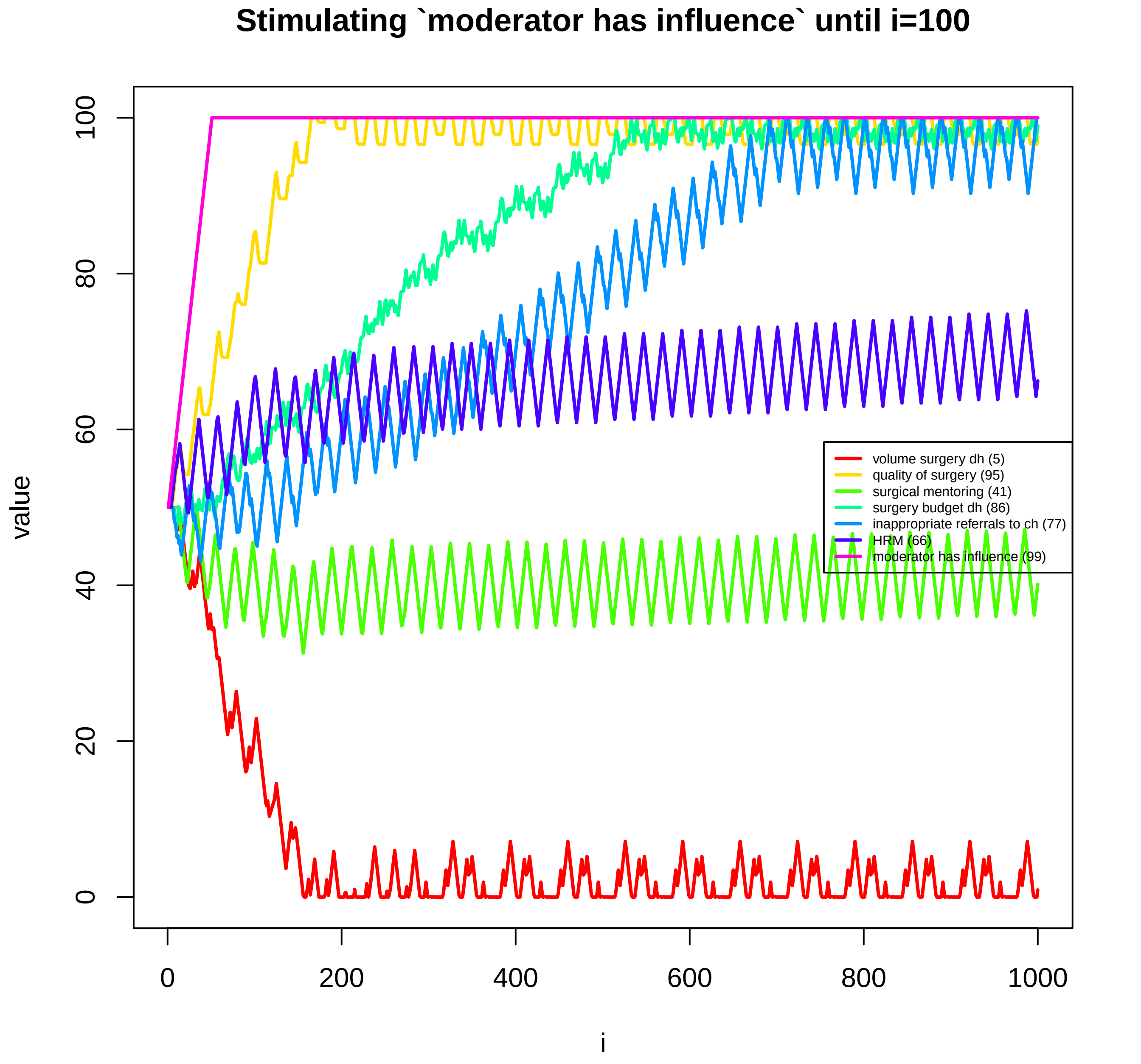

Stimulating `moderator has influence` until i=100
value
i
0
20
40
60
80
100
0
200
400
600
800
1000
volume surgery dh (5)
quality of surgery (95)
surgical mentoring (41)
surgery budget dh (86)
inappropriate referrals to ch (77)
HRM (66)
moderator has influence (99)

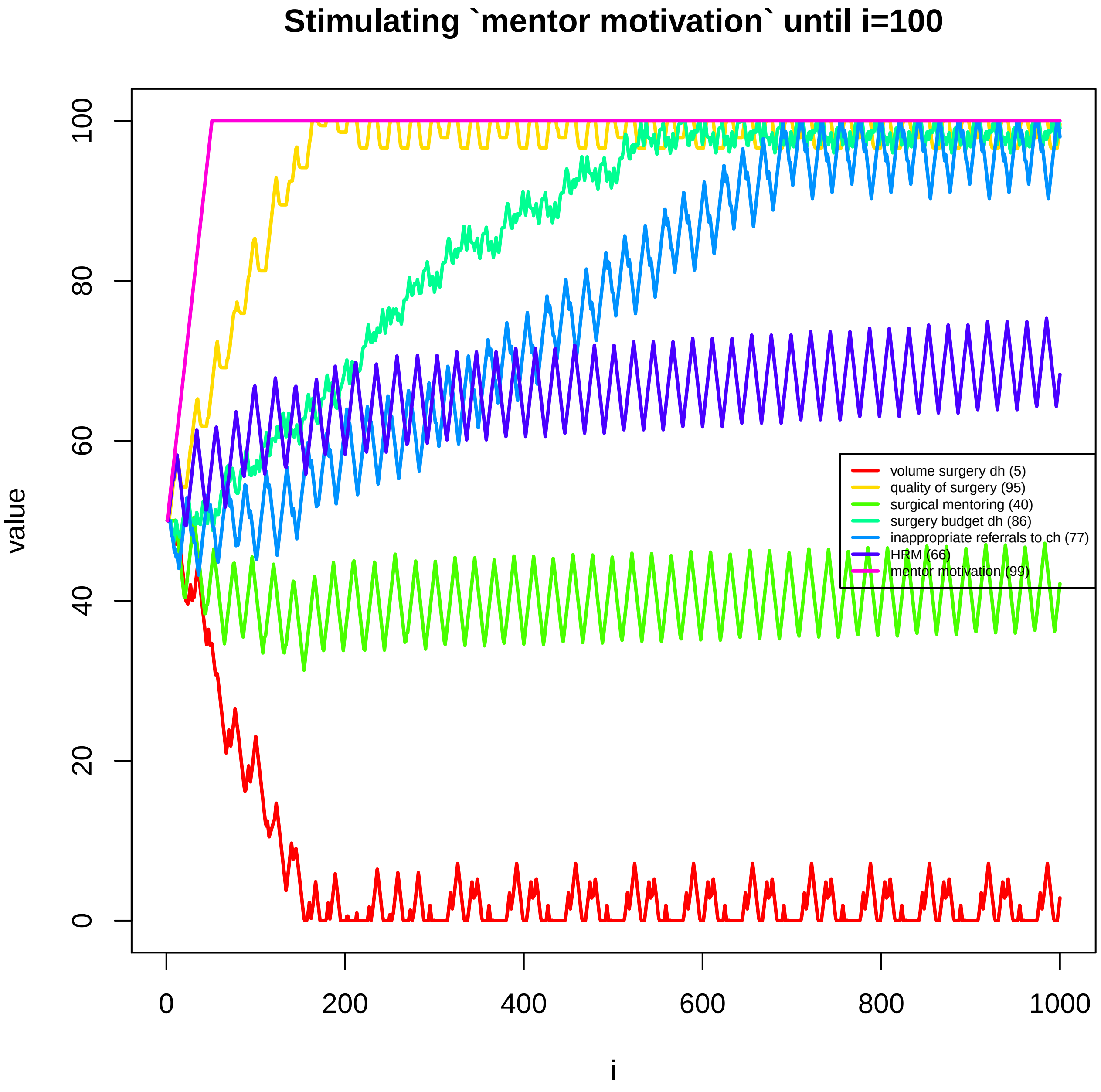
Stimulating `mentor motivation` until i=100
value
i
0
20
40
60
80
100
0
200
400
600
800
1000
volume surgery dh (5)
quality of surgery (95)
surgical mentoring (40)
surgery budget dh (86)
inappropriate referrals to ch (77)
HRM (66)
mentor motivation (99)

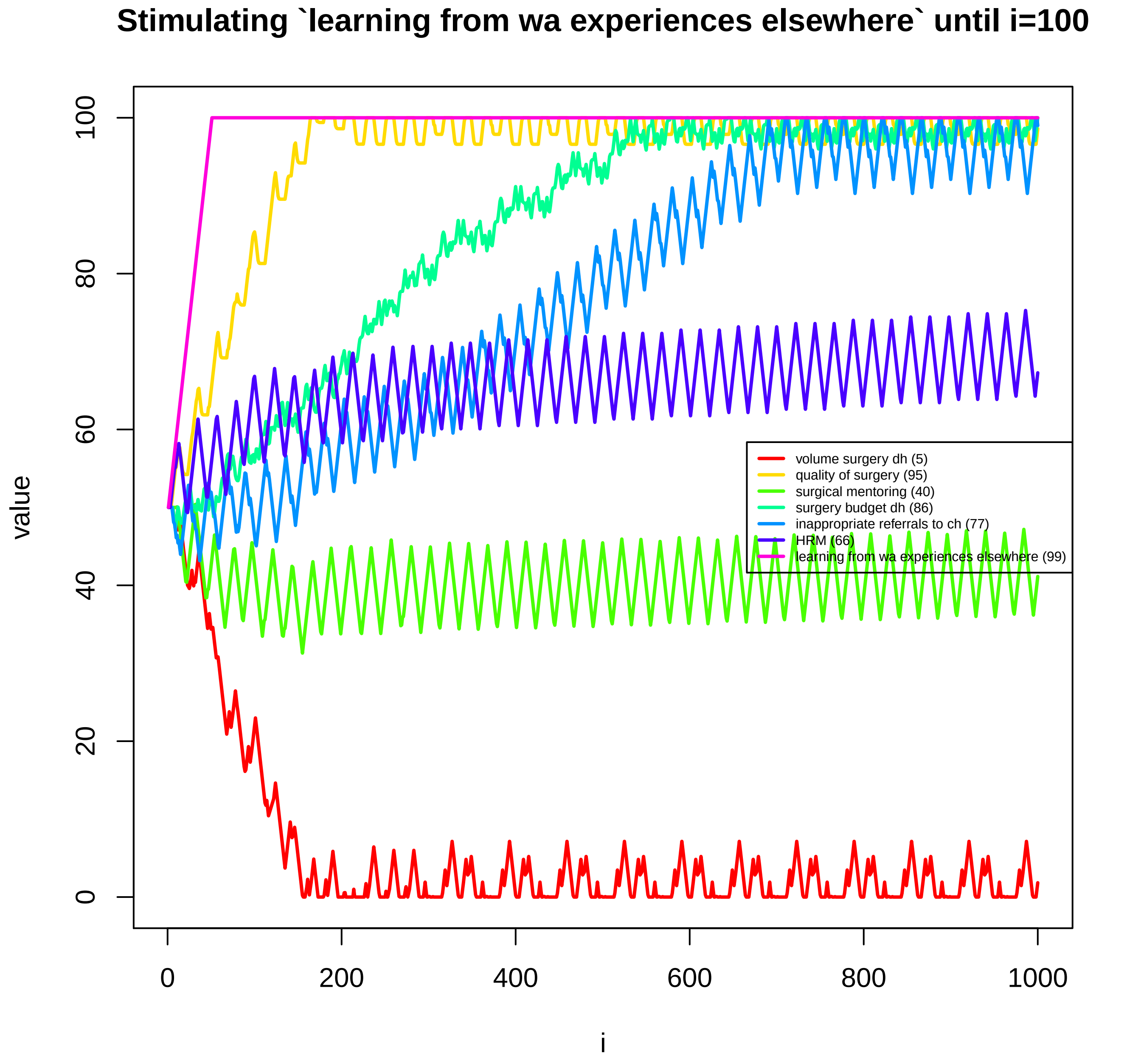
Stimulating `learning from wa experiences elsewhere` until i=100
value
i
0
20
40
60
80
100
0
200
400
600
800
1000
volume surgery dh (5)
quality of surgery (95)
surgical mentoring (40)
surgery budget dh (86)
inappropriate referrals to ch (77)
HRM (66)
learning from wa experiences elsewhere (99)

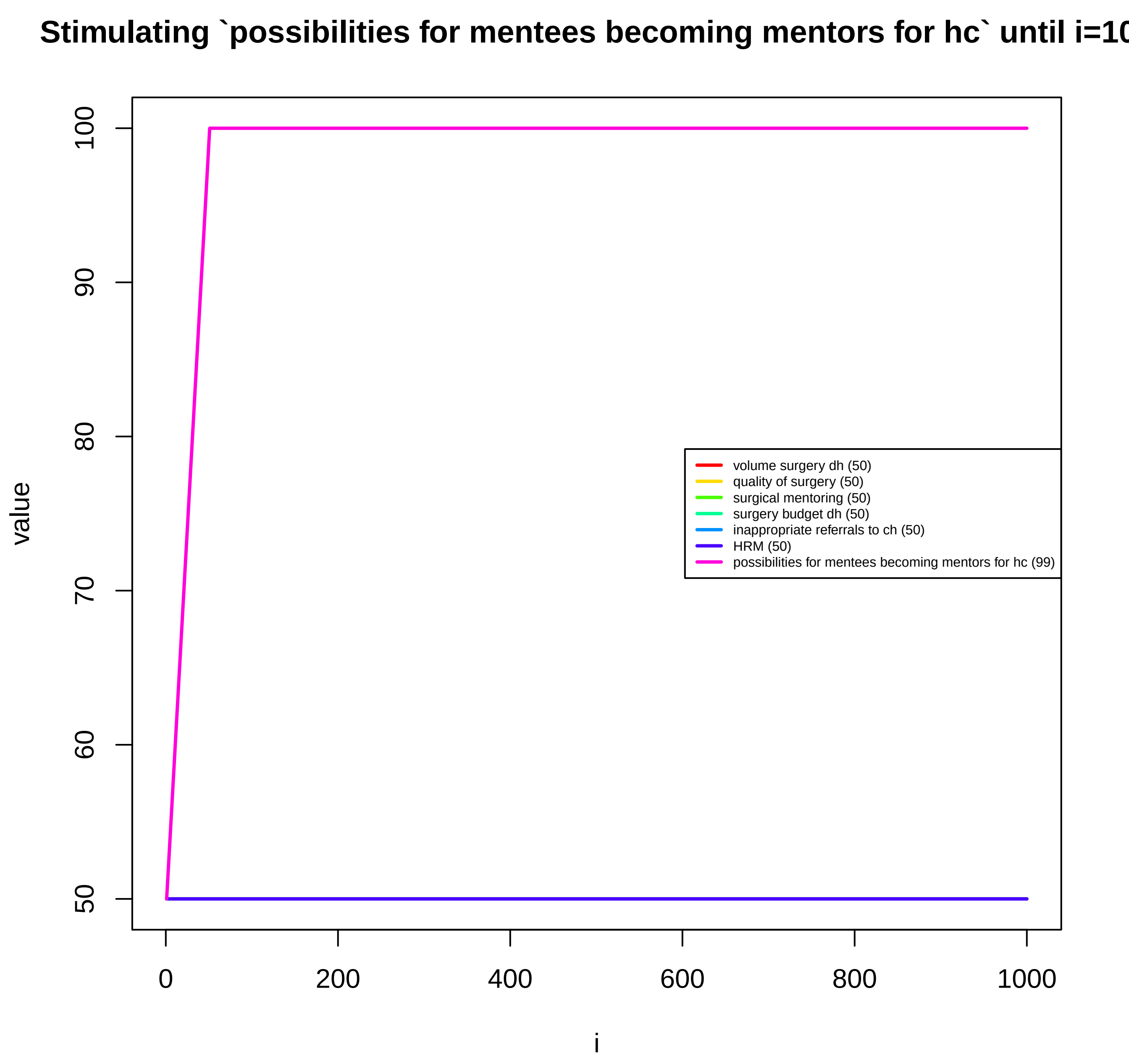

Stimulating `possibilities for mentees becoming mentors for hc` until i=10
value
100
90
80
70
60
50
0
200
400
600
800
1000
i
volume surgery dh (50)
quality of surgery (50)
surgical mentoring (50)
surgery budget dh (50)
inappropriate referrals to ch (50)
HRM (50)
possibilities for mentees becoming mentors for hc (99)

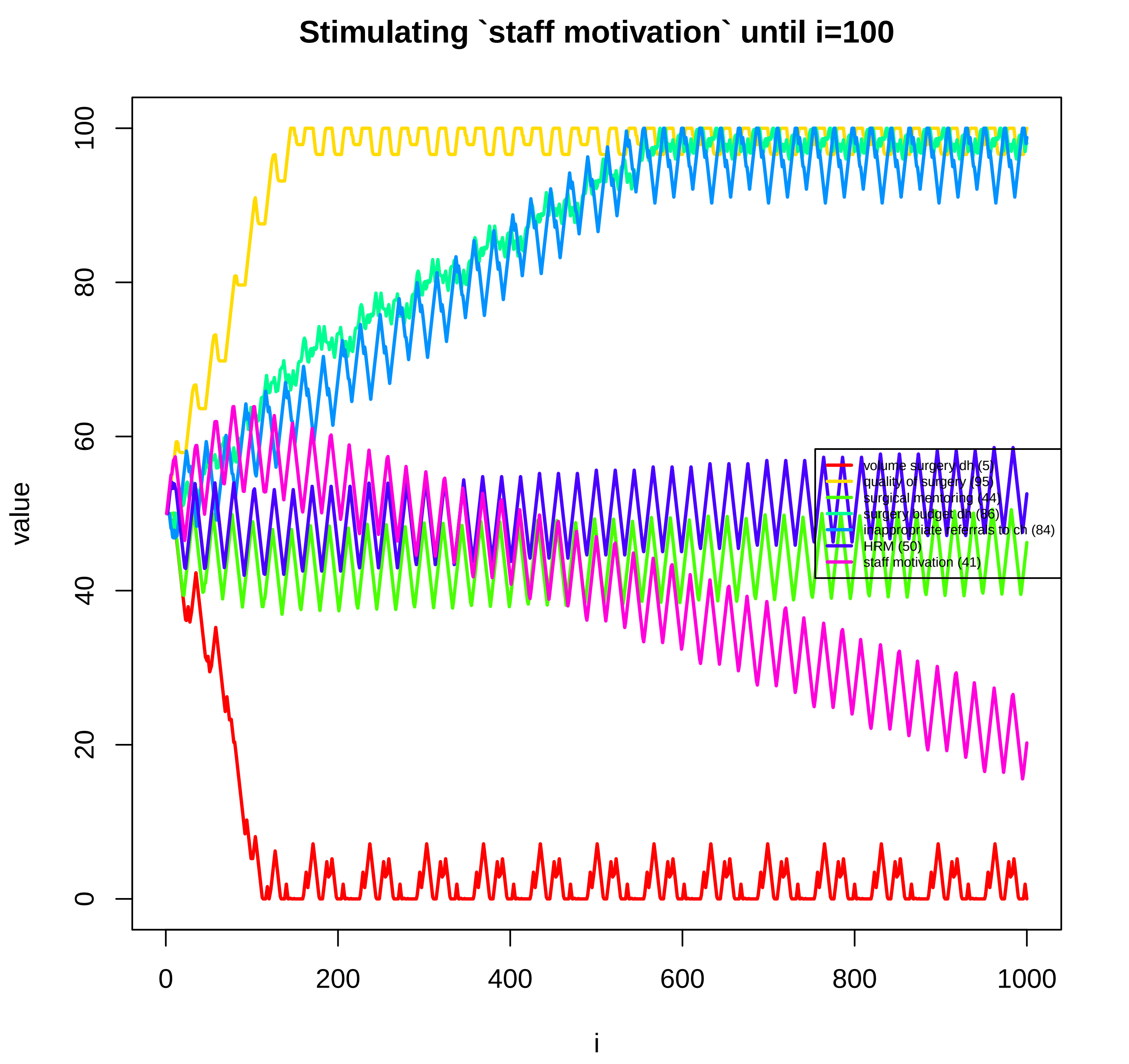

Stimulating `staff motivation` until i=100
value
i
0
20
40
60
80
100
0
200
400
600
800
1000
volume surgery dh (5)
quality of surgery (95)
surgical mentoring (44)
surgery budget dh (86)
inappropriate referrals to ch (84)
HRM (50)
staff motivation (41)

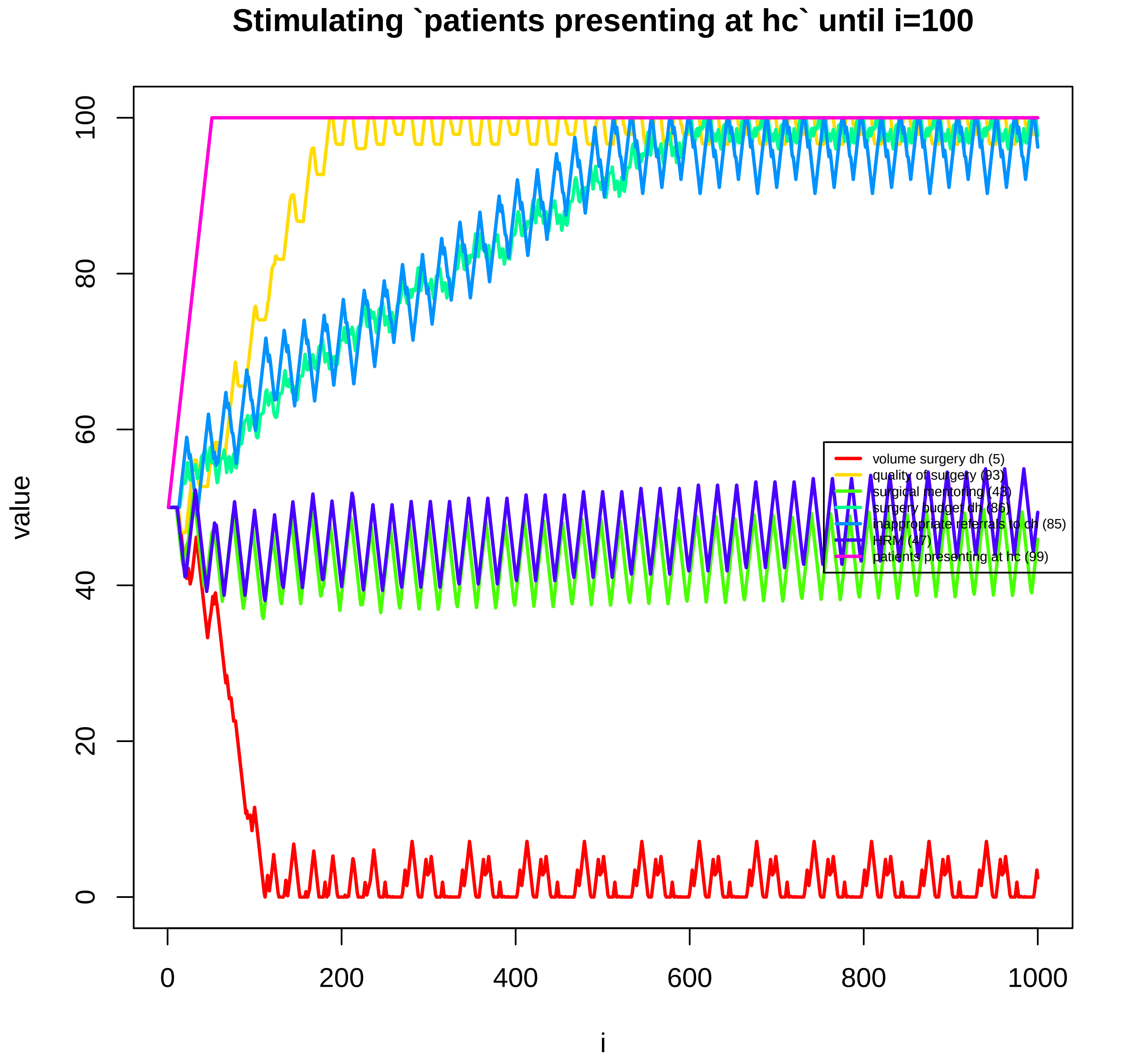

Stimulating `patients presenting at hc` until i=100
value
i
0
20
40
60
80
100
0
200
400
600
800
1000
volume surgery dh (5)
quality of surgery (93)
surgical mentoring (43)
surgery budget dh (86)
inappropriate referrals to dh (85)
HRM (47)
patients presenting at hc (99)

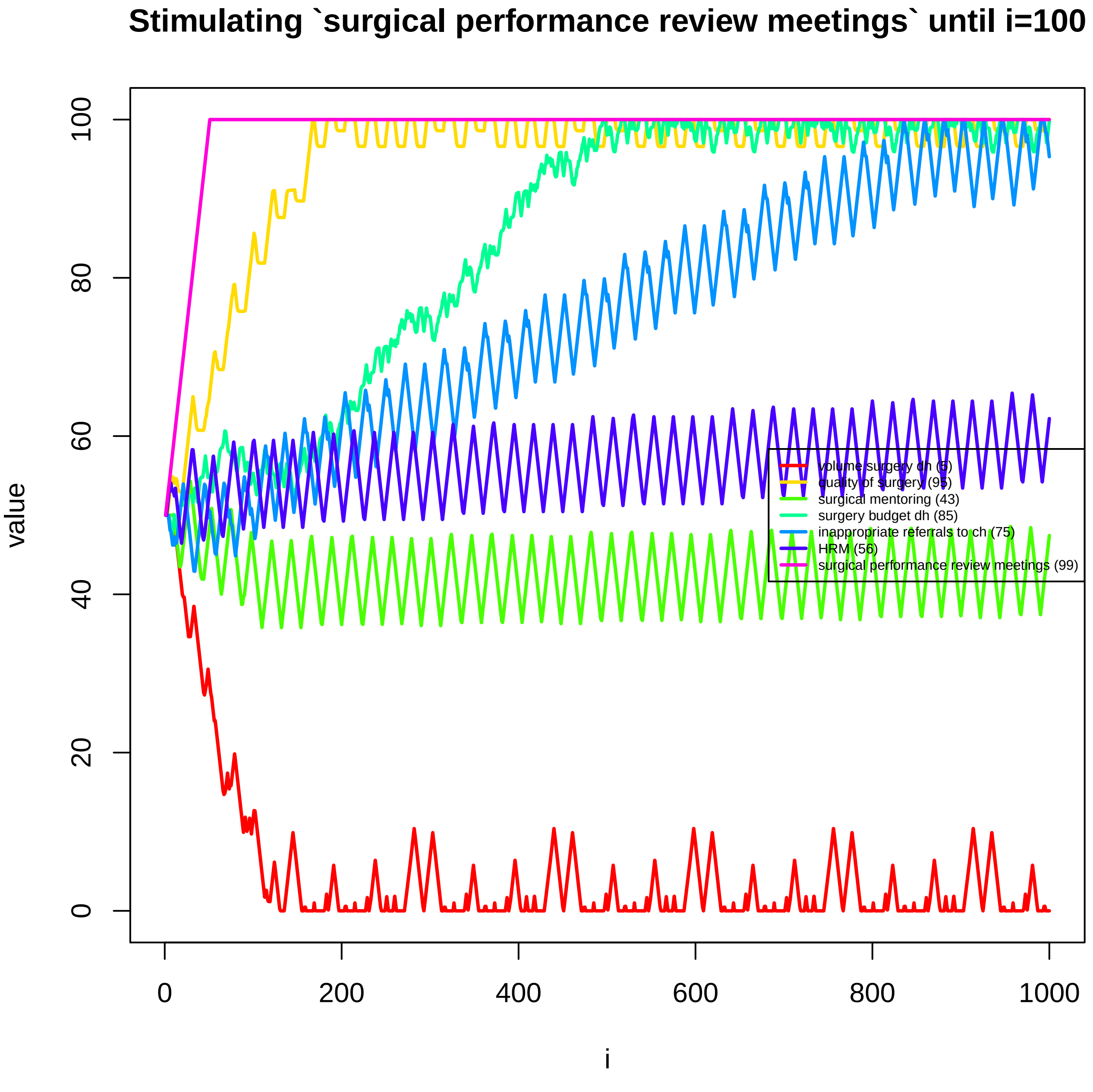

Stimulating `surgical performance review meetings` until i=100
value
i
0
20
40
60
80
100
0
200
400
600
800
1000
volume surgery dh (5)
quality of surgery (95)
surgical mentoring (43)
surgery budget dh (85)
inappropriate referrals to ch (75)
HRM (56)
surgical performance review meetings (99)

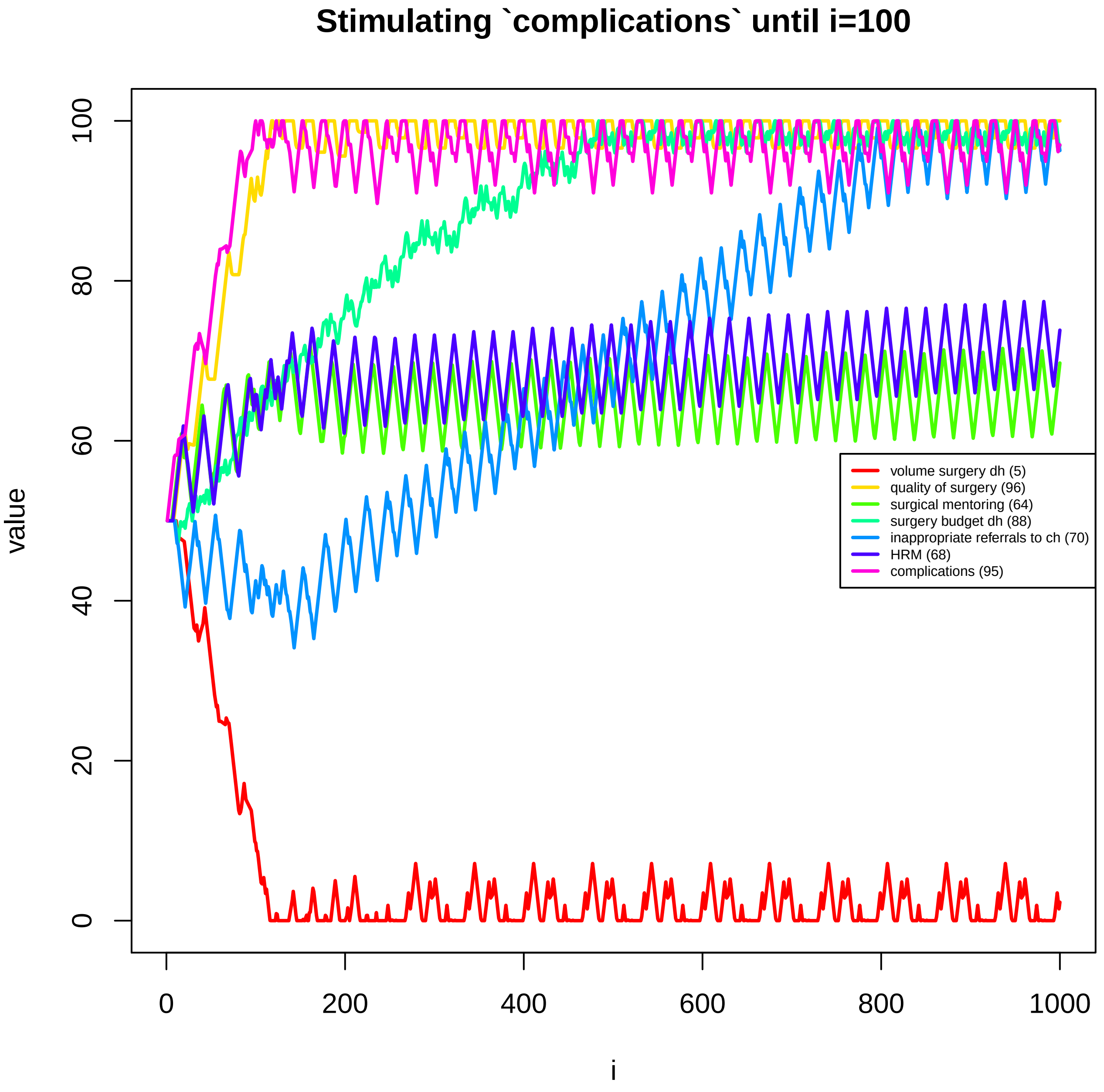

Stimulating `complications` until i=100
value
i
0
20
40
60
80
100
0
200
400
600
800
1000
volume surgery dh (5)
quality of surgery (96)
surgical mentoring (64)
surgery budget dh (88)
inappropriate referrals to ch (70)
HRM (68)
complications (95)

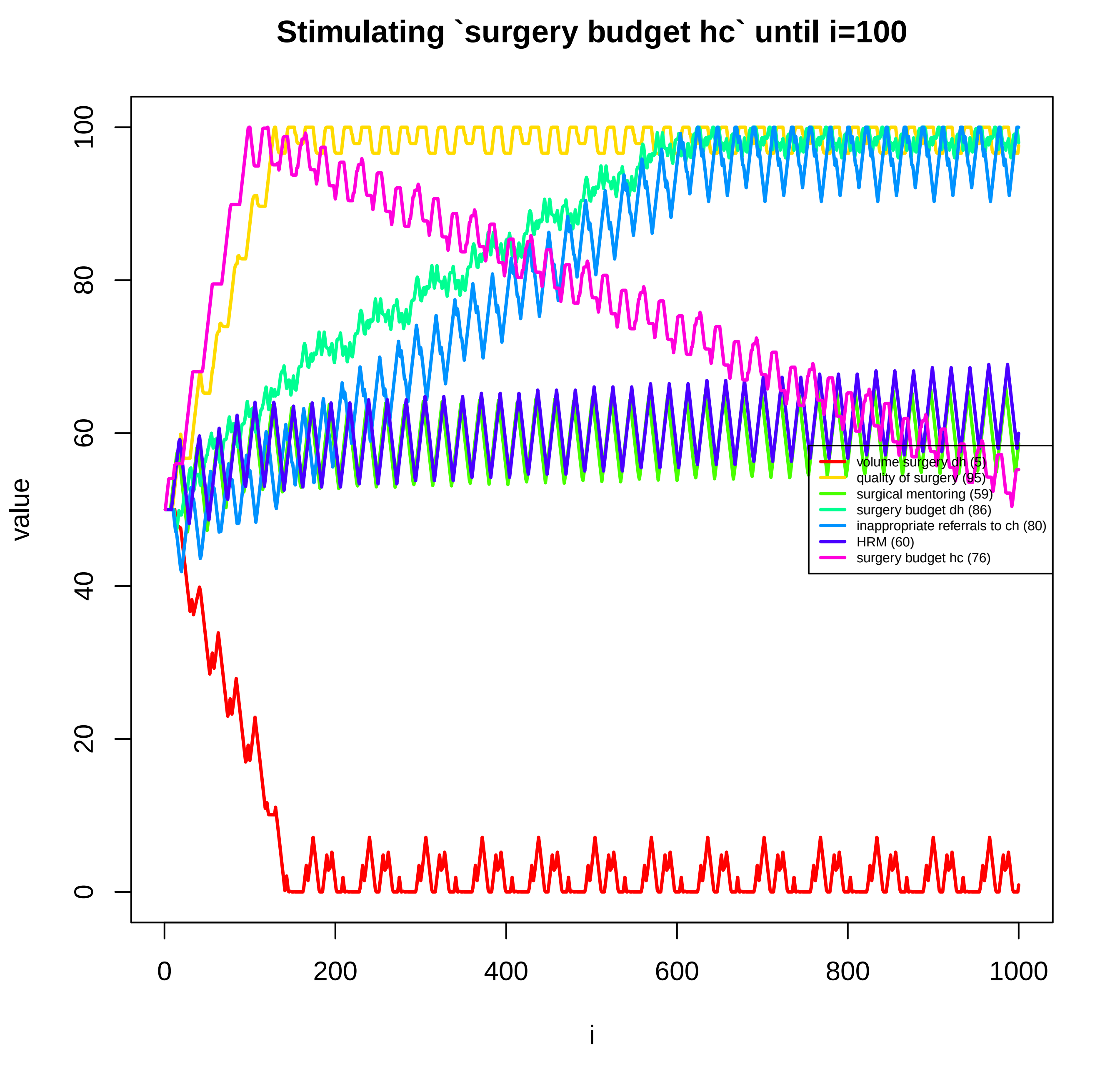

Stimulating `surgery budget hc` until i=100
value
i
0
20
40
60
80
100
0
200
400
600
800
1000
volume surgery dh (5)
quality of surgery (95)
surgical mentoring (59)
surgery budget dh (86)
inappropriate referrals to ch (80)
HRM (60)
surgery budget hc (76)

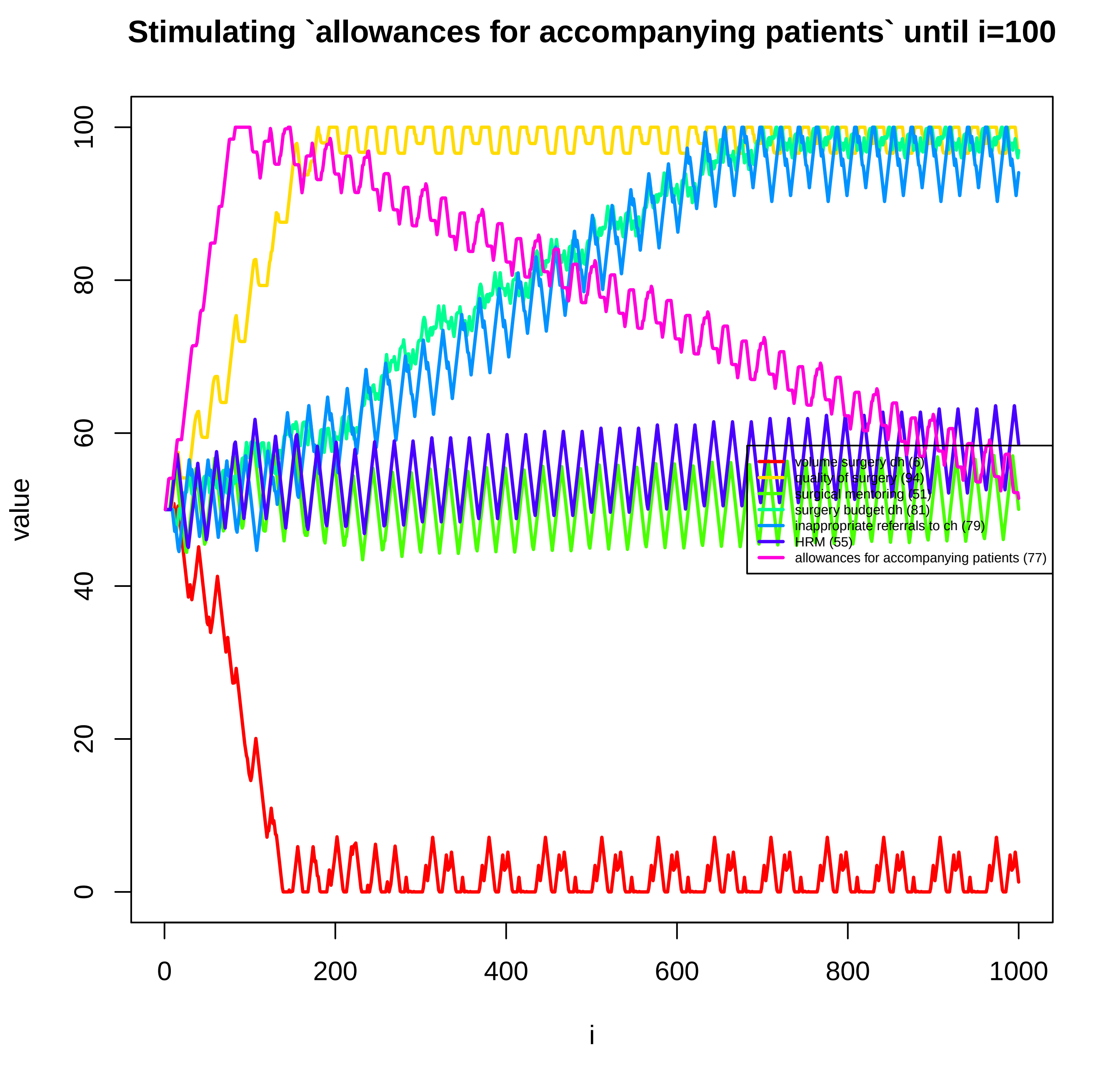

Stimulating `allowances for accompanying patients` until i=100
value
i
0
20
40
60
80
100
0
200
400
600
800
1000
volume surgery ch (6)
quality of surgery (94)
surgical mentoring (51)
surgery budget dh (81)
inappropriate referrals to ch (79)
HRM (55)
allowances for accompanying patients (77)

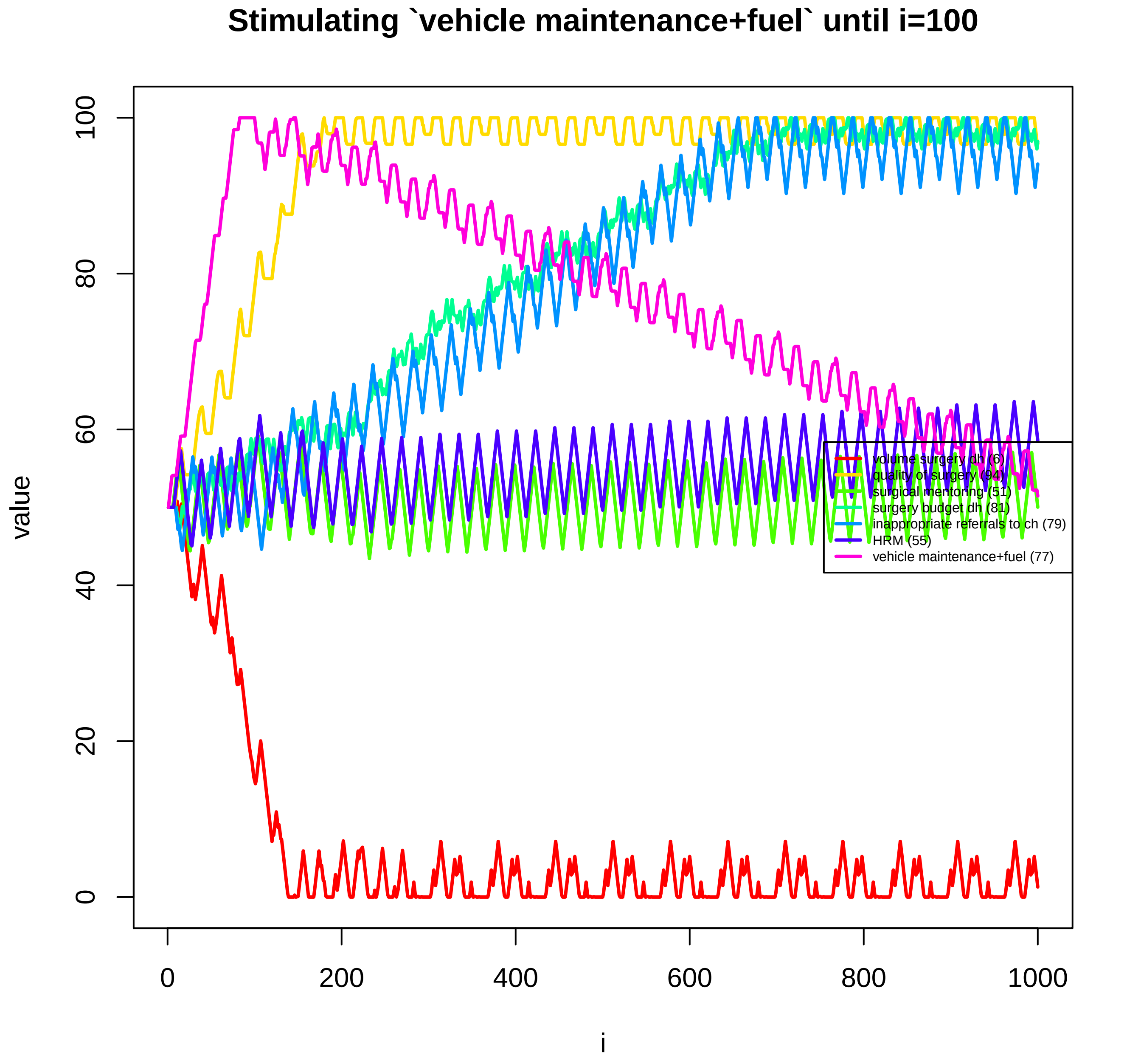
Stimulating `vehicle maintenance+fuel` until i=100
value
i
0
20
40
60
80
100
0
200
400
600
800
1000
volume surgery dh (6)
quality of surgery (94)
surgical mentoring (51)
surgery budget dh (81)
inappropriate referrals to ch (79)
HRM (55)
vehicle maintenance+fuel (77)

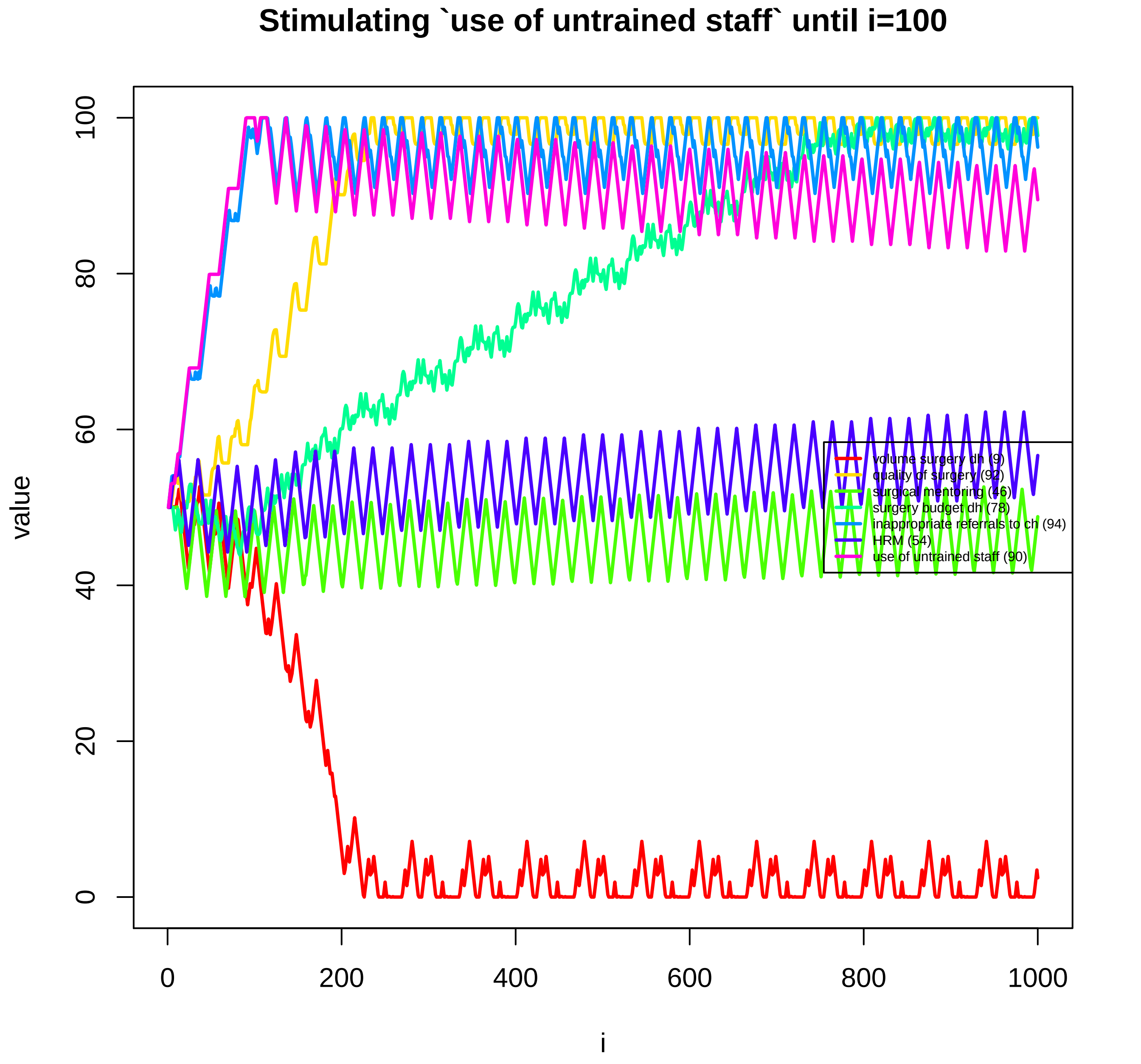

Stimulating `use of untrained staff` until i=100
value
i
0
20
40
60
80
100
0
200
400
600
800
1000
volume surgery dh (9)
quality of surgery (92)
surgical mentoring (46)
surgery budget dh (78)
inappropriate referrals to ch (94)
HRM (54)
use of untrained staff (90)

Stimulating `self−referrals to ch` until i=100

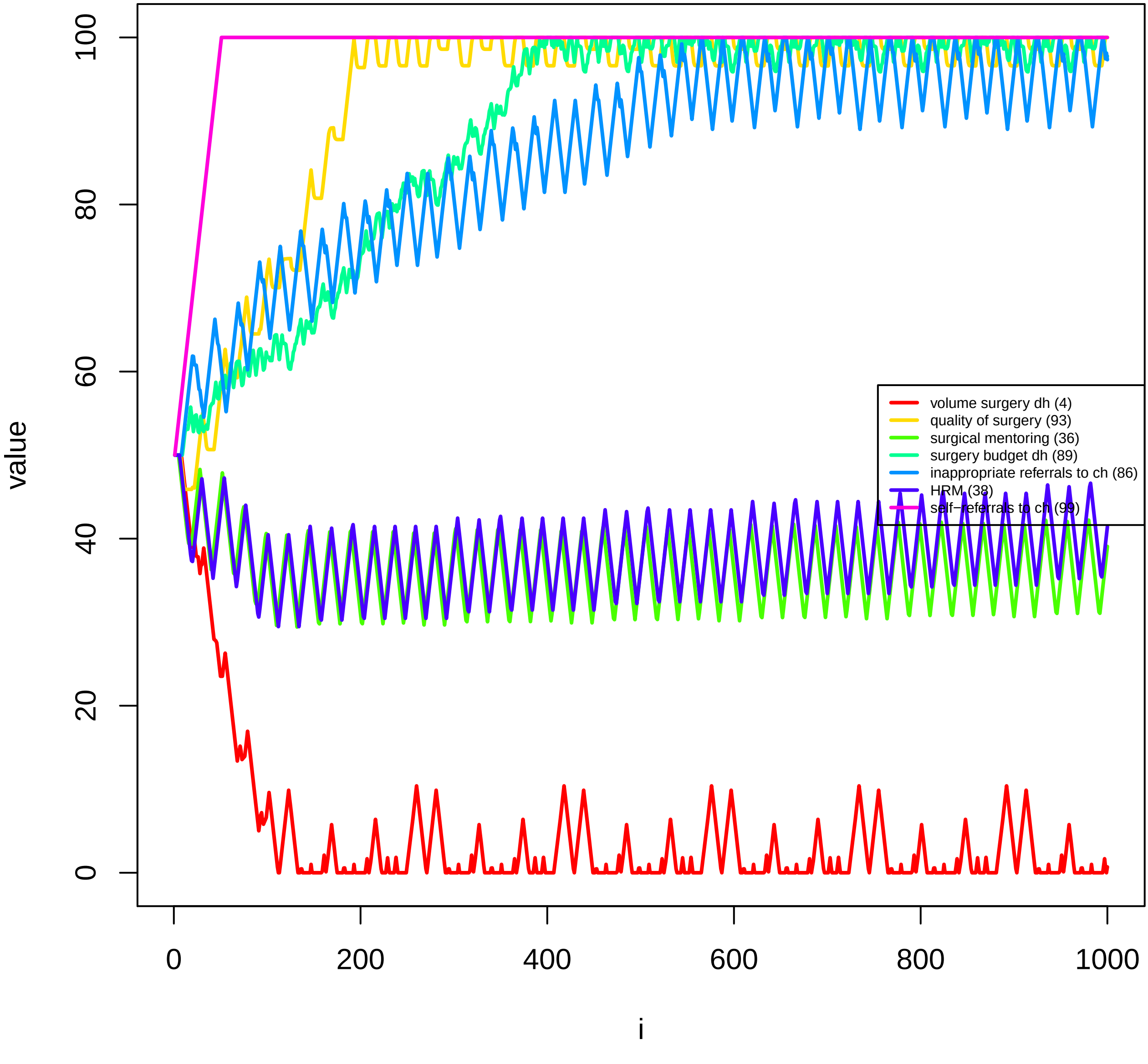

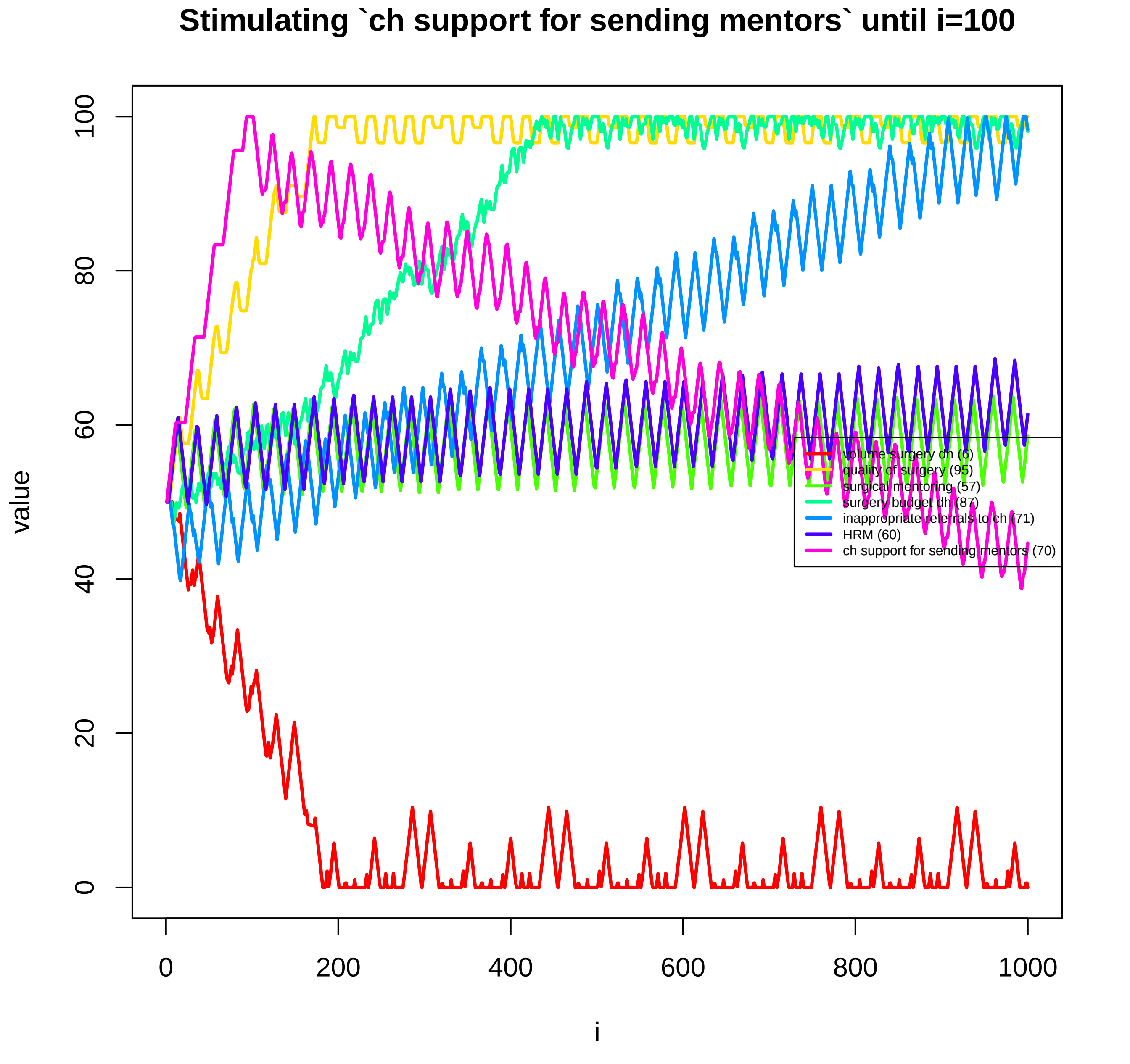
Stimulating `ch support for sending mentors` until i=100
value
i
0
20
40
60
80
100
0
200
400
600
800
1000
volume surgery dh (6)
quality of surgery (95)
surgical mentoring (57)
surgery budget dh (87)
inappropriate referrals to ch (71)
HRM (60)
ch support for sending mentors (70)

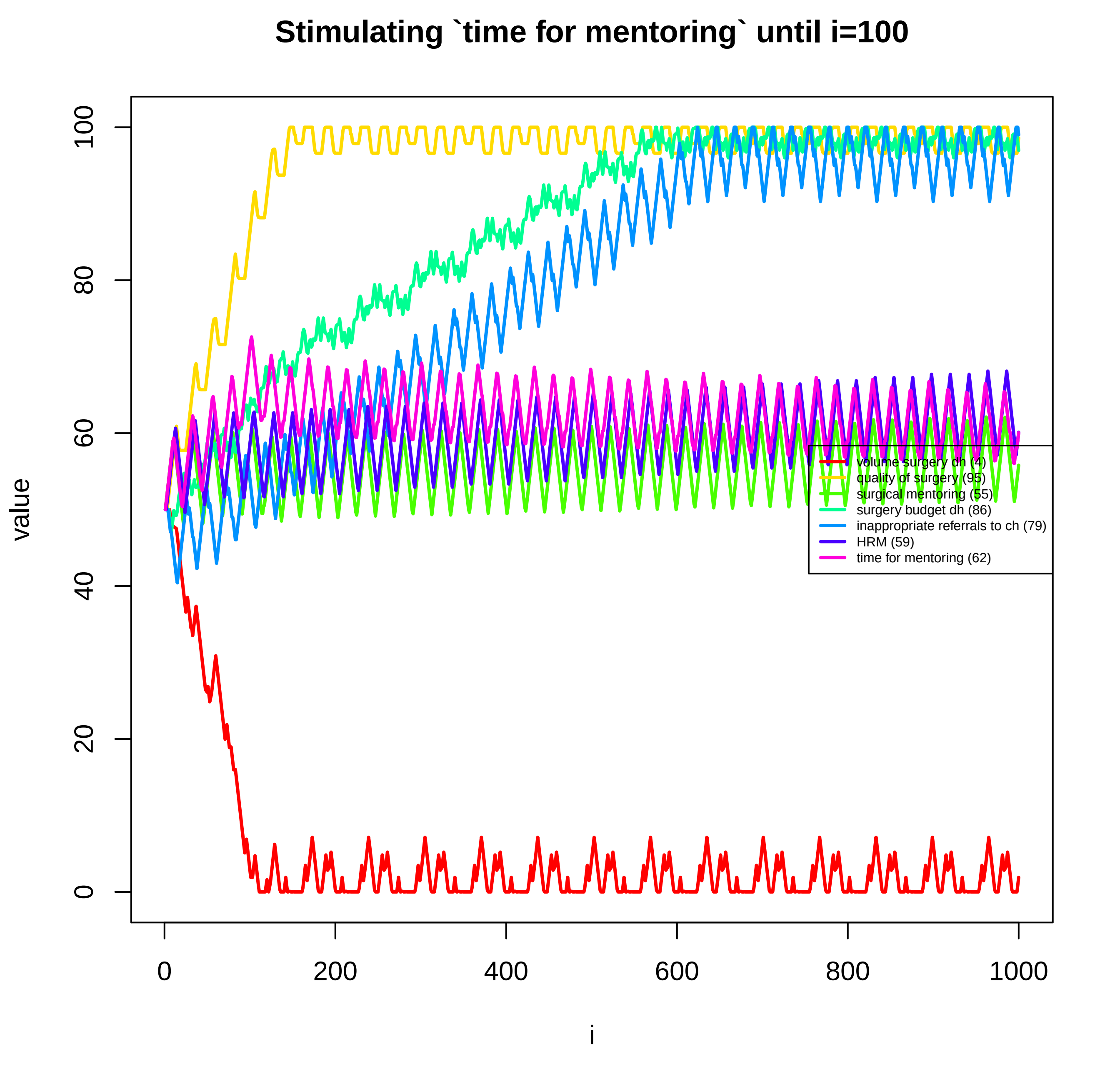
Stimulating `time for mentoring` until i=100
value
i
0
20
40
60
80
100
0
200
400
600
800
1000
volume surgery dh (4)
quality of surgery (95)
surgical mentoring (55)
surgery budget dh (86)
inappropriate referrals to ch (79)
HRM (59)
time for mentoring (62)

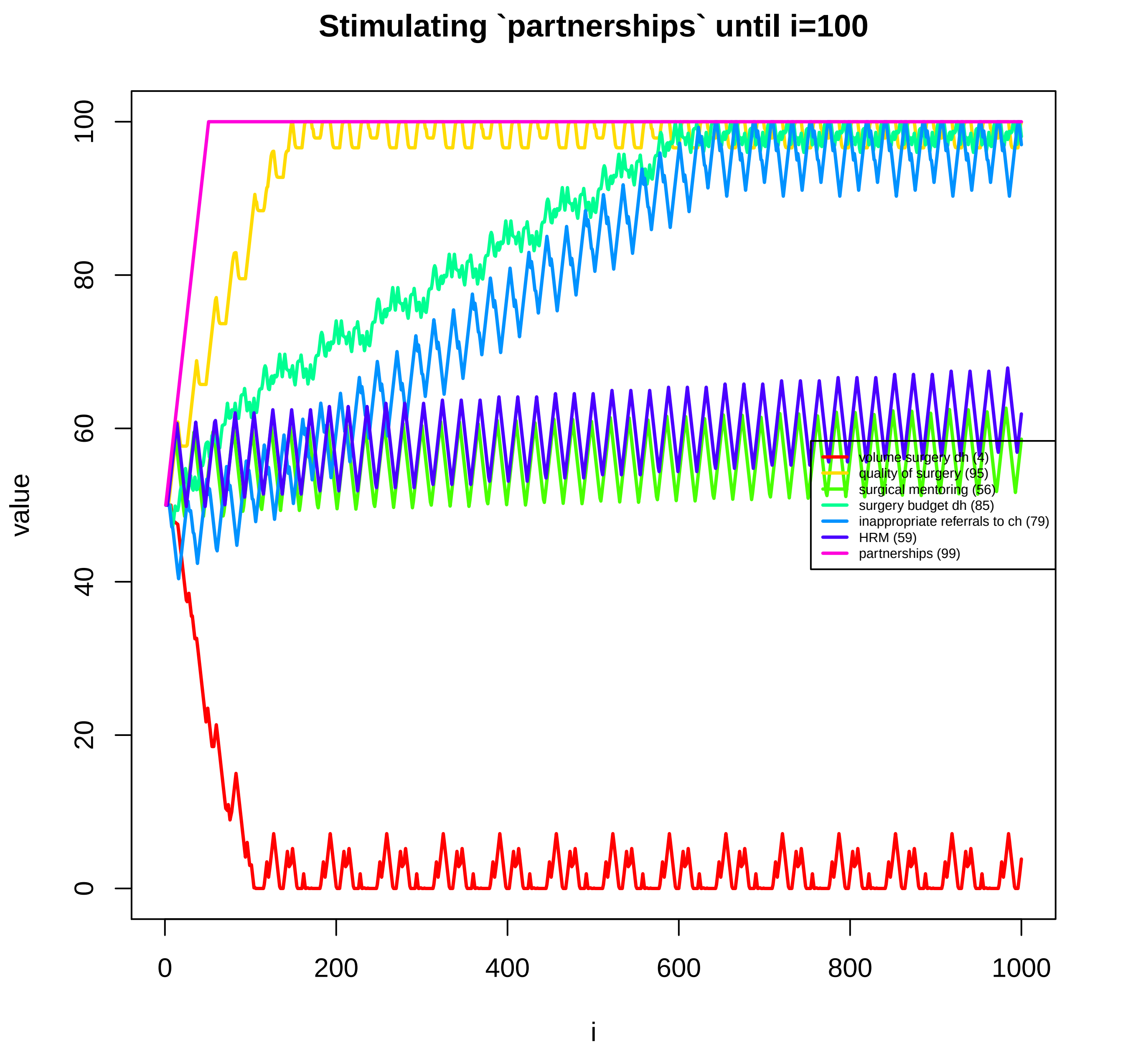

Stimulating `partnerships` until i=100
value
i
0
20
40
60
80
100
0
200
400
600
800
1000
volume surgery dh (4)
quality of surgery (95)
surgical mentoring (56)
surgery budget dh (85)
inappropriate referrals to ch (79)
HRM (59)
partnerships (99)

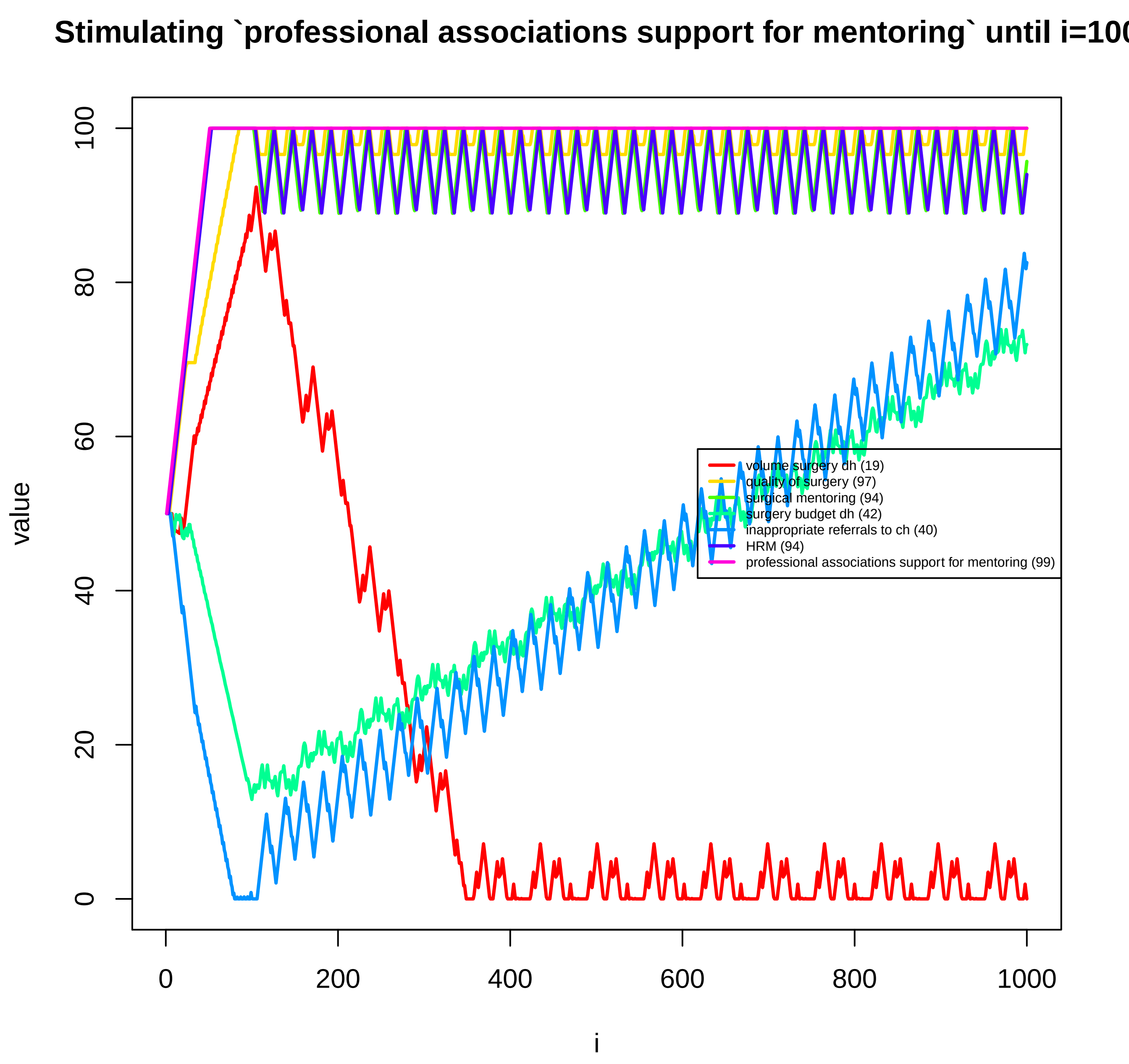
Stimulating `professional associations support for mentoring` until i=100
value
i
0
20
40
60
80
100
0
200
400
600
800
1000
volume surgery dh (19)
quality of surgery (97)
surgical mentoring (94)
surgery budget dh (42)
inappropriate referrals to ch (40)
HRM (94)
professional associations support for mentoring (99)

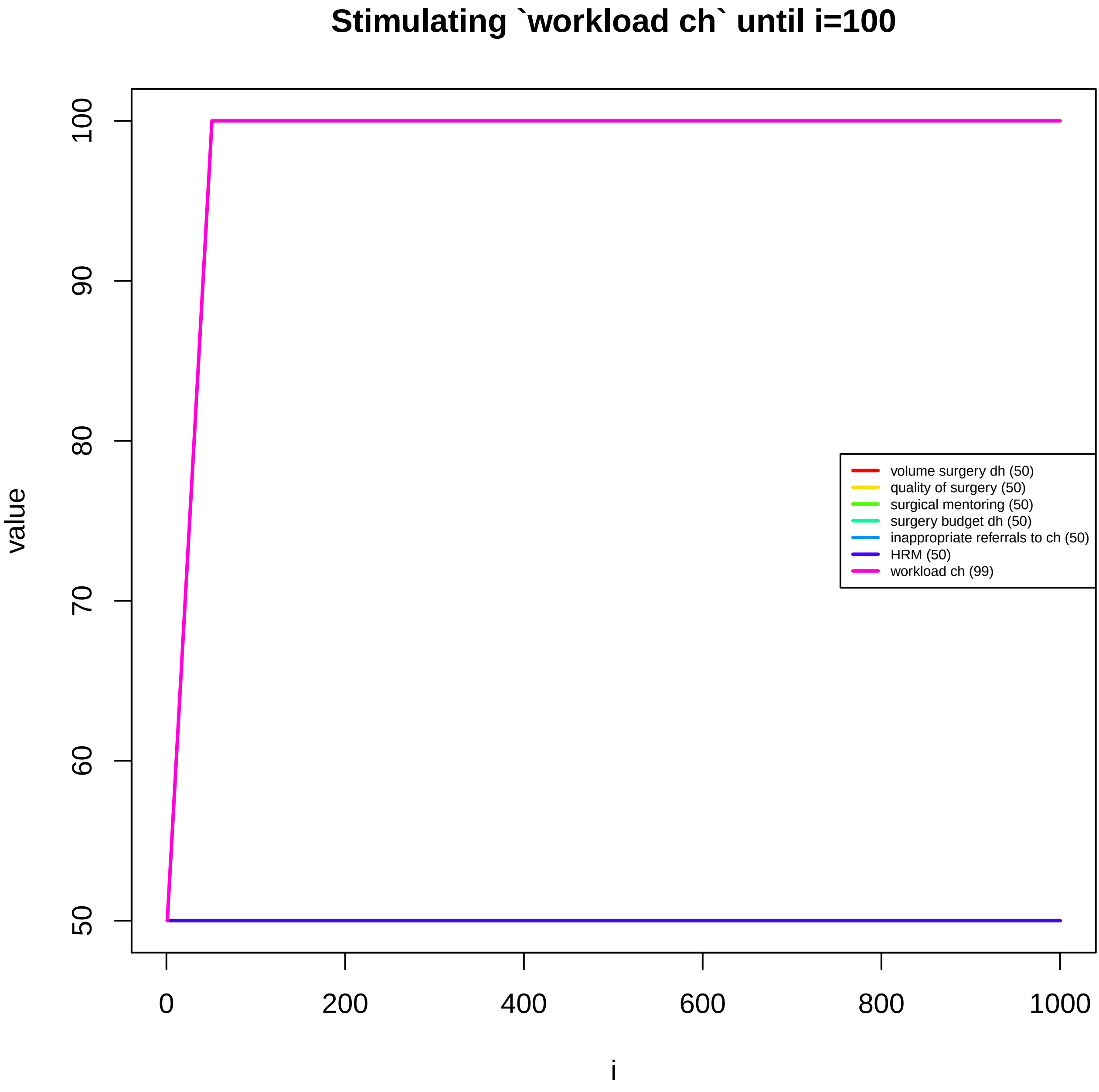

Stimulating `workload ch` until i=100
value
i
100
90
80
70
60
50
0
200
400
600
800
1000
volume surgery dh (50)
quality of surgery (50)
surgical mentoring (50)
surgery budget dh (50)
inappropriate referrals to ch (50)
HRM (50)
workload ch (99)

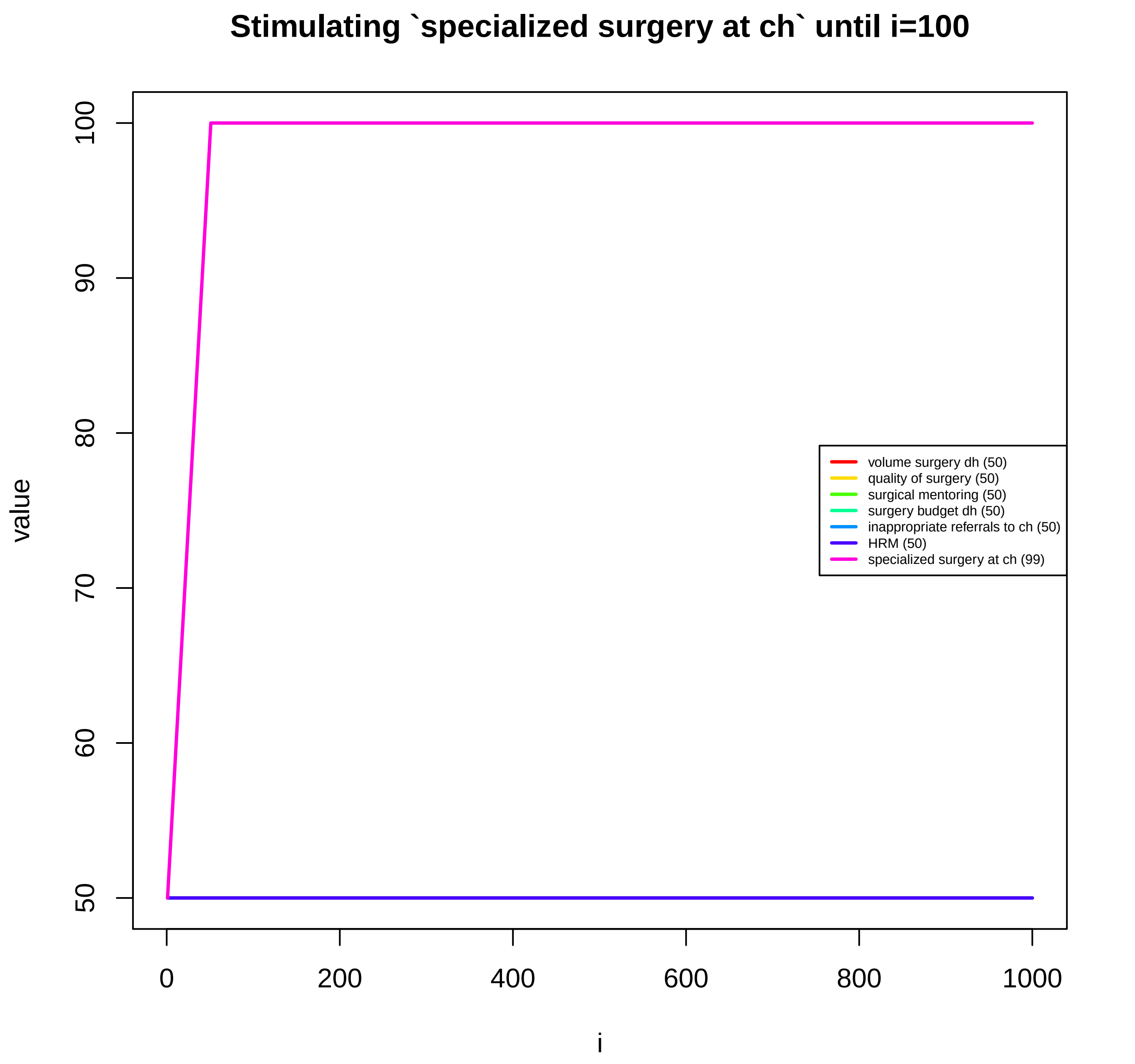

Stimulating `specialized surgery at ch` until i=100
value
i
100
90
80
70
60
50
0
200
400
600
800
1000
volume surgery dh (50)
quality of surgery (50)
surgical mentoring (50)
surgery budget dh (50)
inappropriate referrals to ch (50)
HRM (50)
specialized surgery at ch (99)

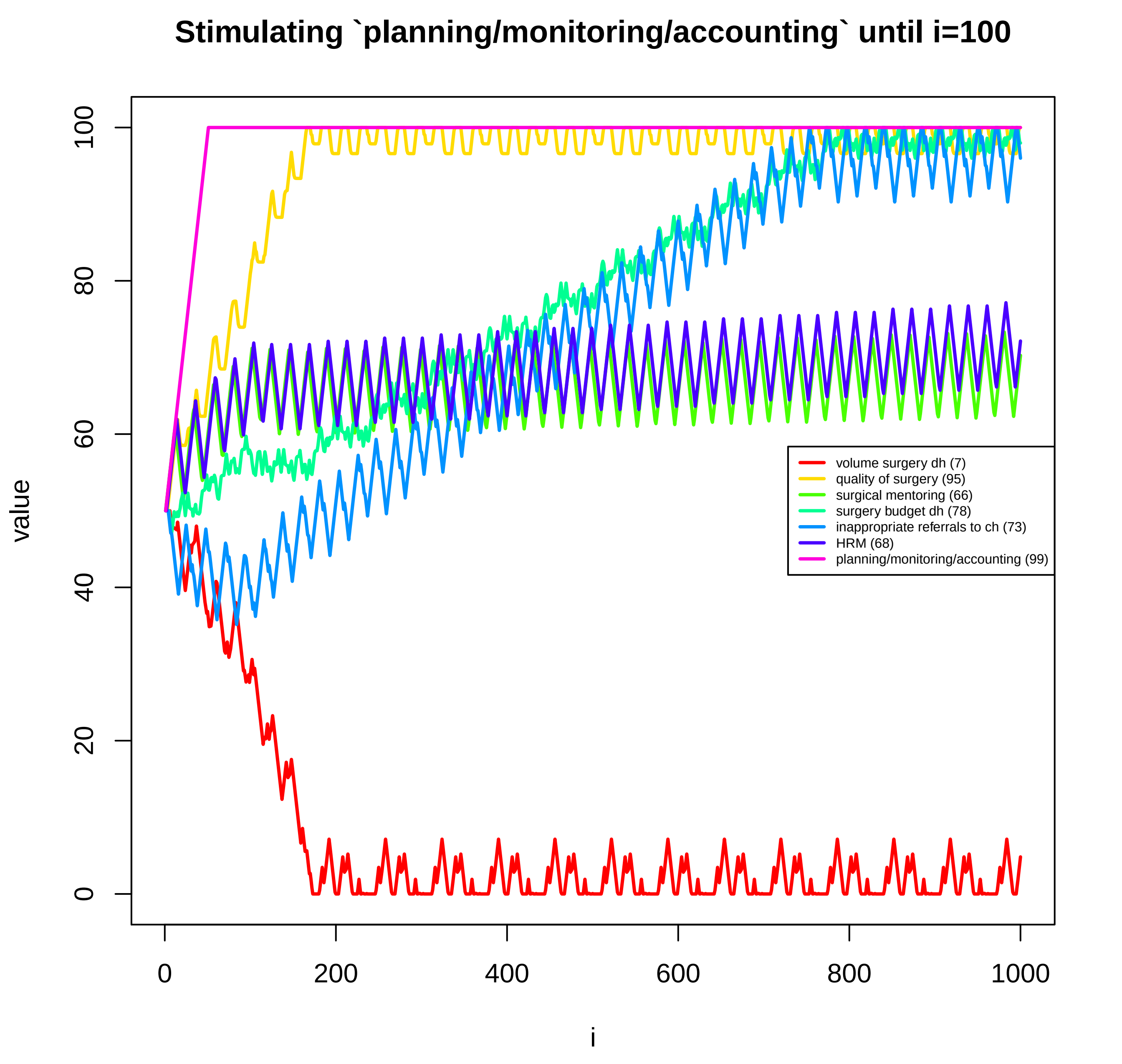

Stimulating `planning/monitoring/accounting` until i=100
value
i
0
20
40
60
80
100
0
200
400
600
800
1000
volume surgery dh (7)
quality of surgery (95)
surgical mentoring (66)
surgery budget dh (78)
inappropriate referrals to ch (73)
HRM (68)
planning/monitoring/accounting (99)

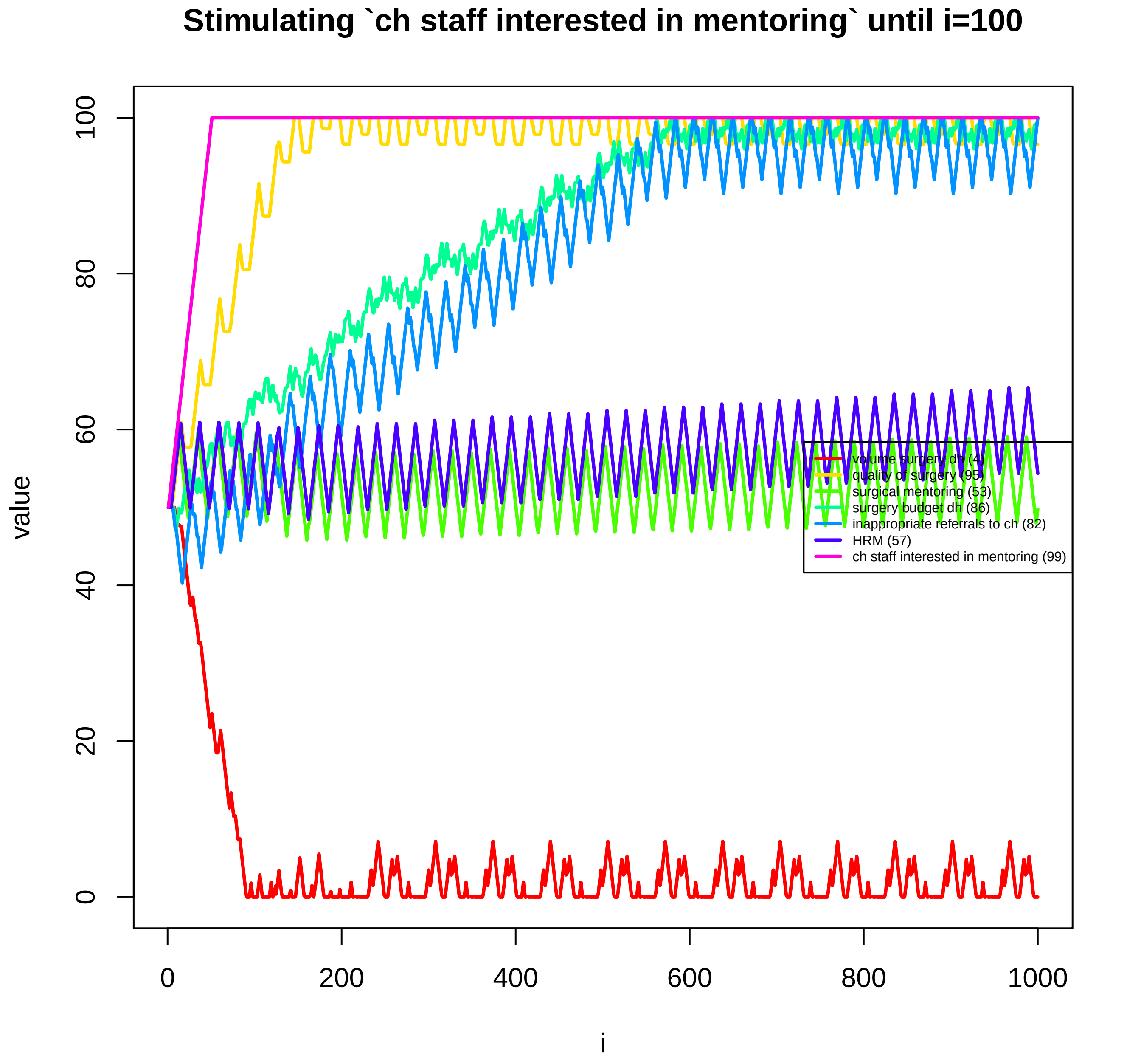

Stimulating `ch staff interested in mentoring` until i=100
value
i
0
20
40
60
80
100
0
200
400
600
800
1000
volume surgery dh (4)
quality of surgery (95)
surgical mentoring (53)
surgery budget dh (86)
inappropriate referrals to ch (82)
HRM (57)
ch staff interested in mentoring (99)

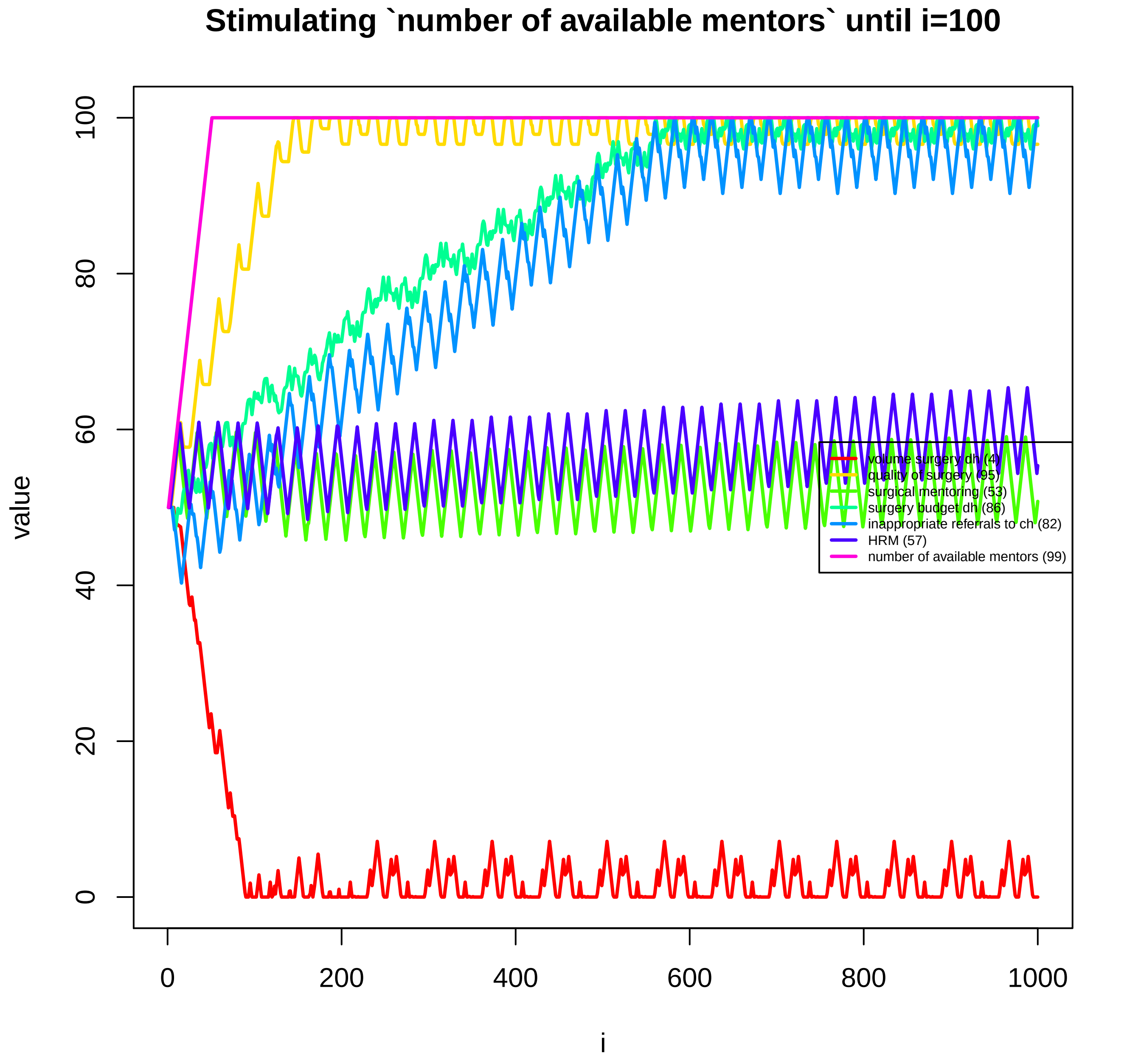

Stimulating `number of available mentors` until i=100
value
i
0
20
40
60
80
100
0
200
400
600
800
1000
volume surgery dh (4)
quality of surgery (95)
surgical mentoring (53)
surgery budget dh (86)
inappropriate referrals to ch (82)
HRM (57)
number of available mentors (99)

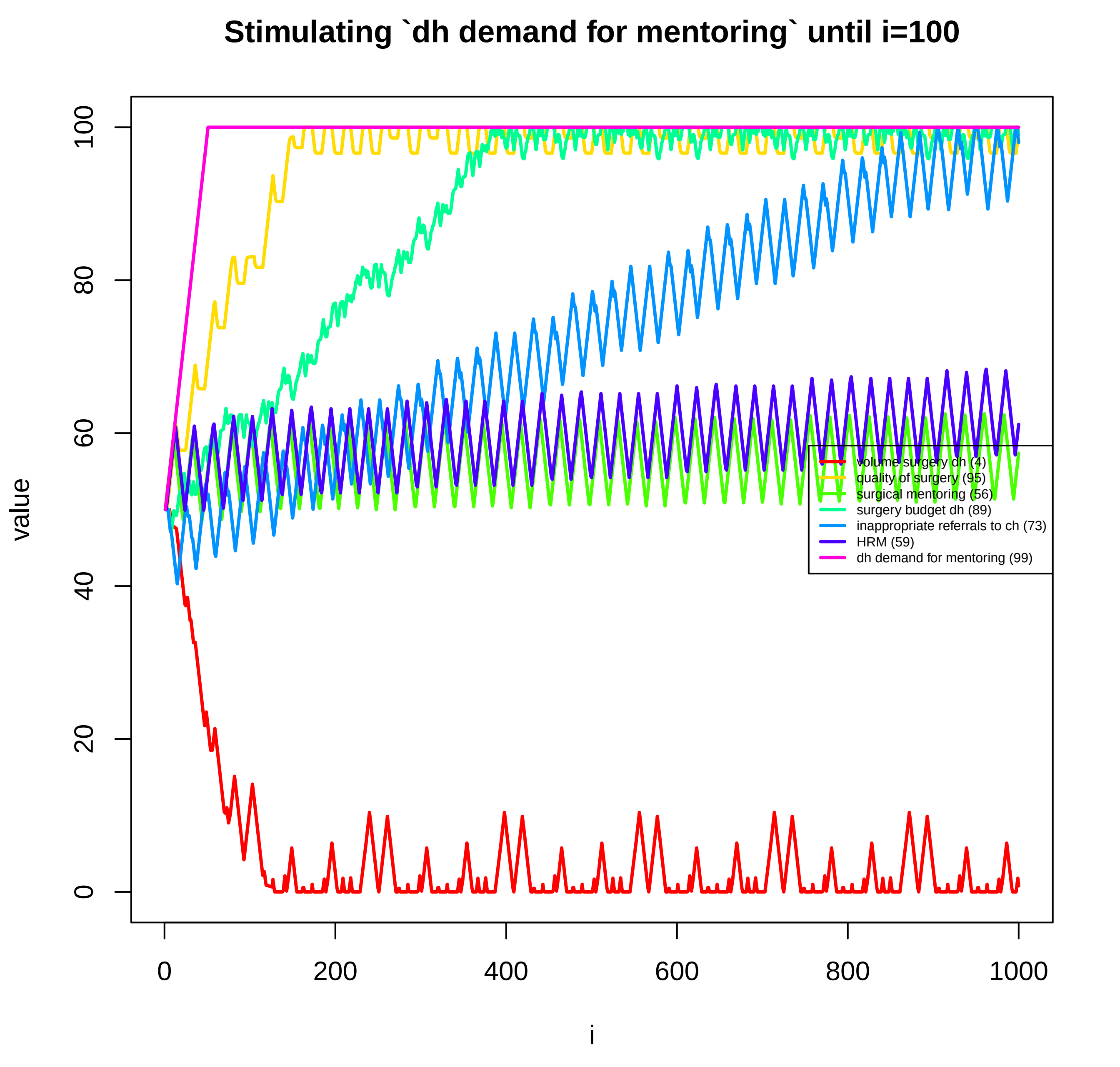
Stimulating `dh demand for mentoring` until i=100
value
i
0
20
40
60
80
100
0
200
400
600
800
1000
volume surgery dh (4)
quality of surgery (95)
surgical mentoring (56)
surgery budget dh (89)
inappropriate referrals to ch (73)
HRM (59)
dh demand for mentoring (99)

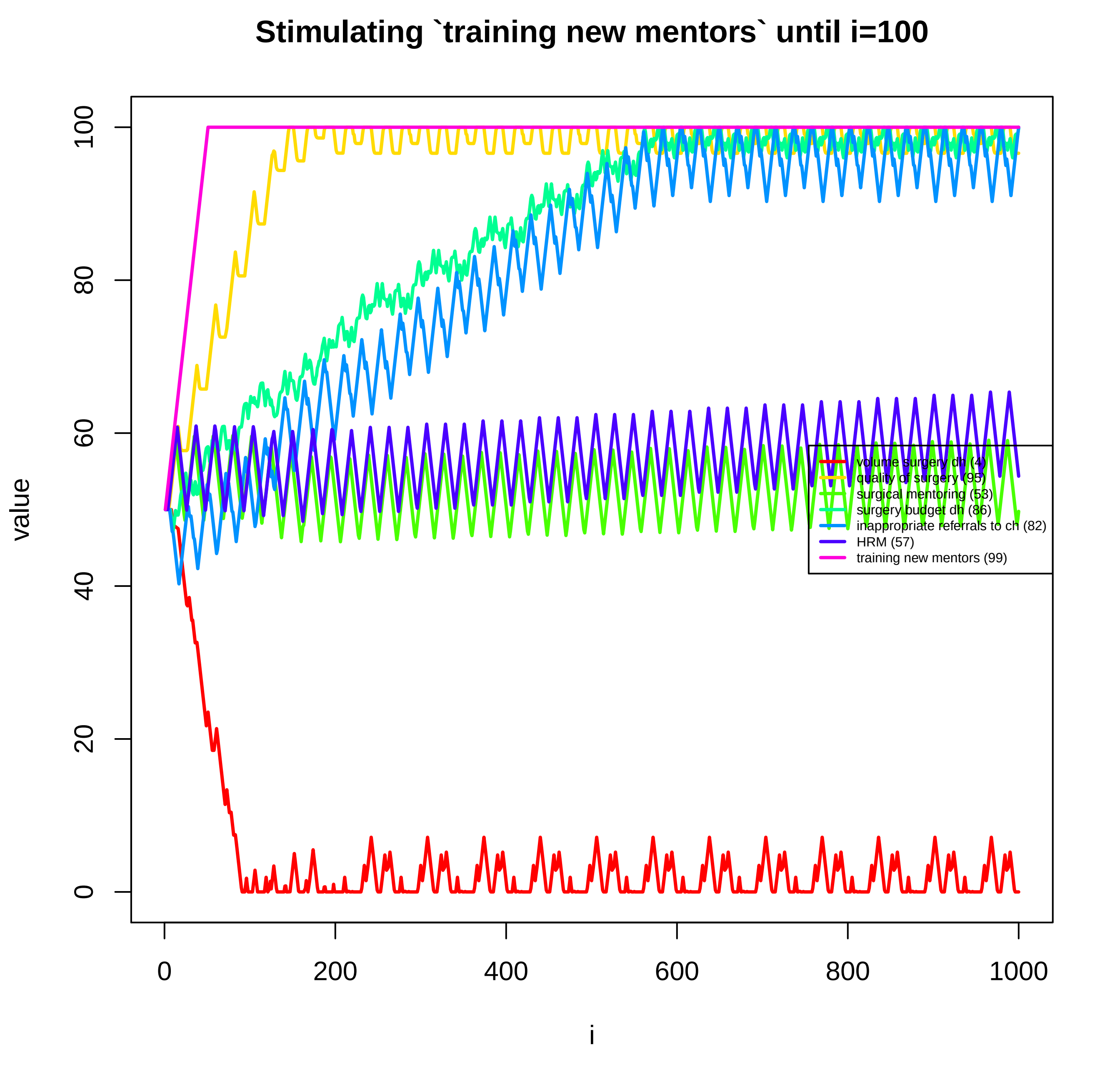
Stimulating `training new mentors` until i=100
value
i
0
20
40
60
80
100
0
200
400
600
800
1000
volume surgery dh (4)
quality of surgery (95)
surgical mentoring (53)
surgery budget dh (86)
inappropriate referrals to ch (82)
HRM (57)
training new mentors (99)

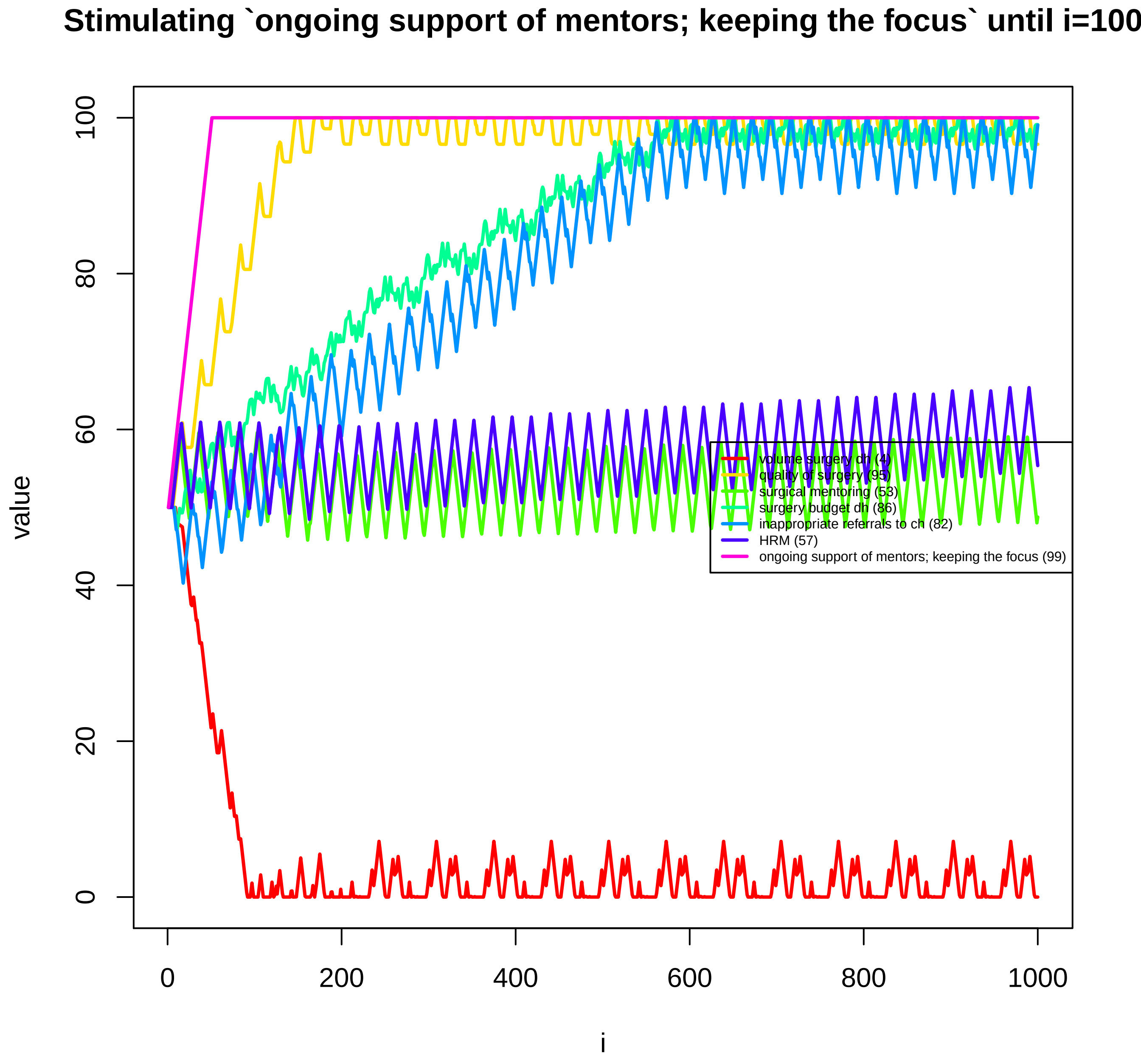
Stimulating `ongoing support of mentors; keeping the focus` until i=100
value
i
0
20
40
60
80
100
0
200
400
600
800
1000
volume surgery dh (4)
quality of surgery (95)
surgical mentoring (53)
surgery budget dh (86)
inappropriate referrals to ch (82)
HRM (57)
ongoing support of mentors; keeping the focus (99)

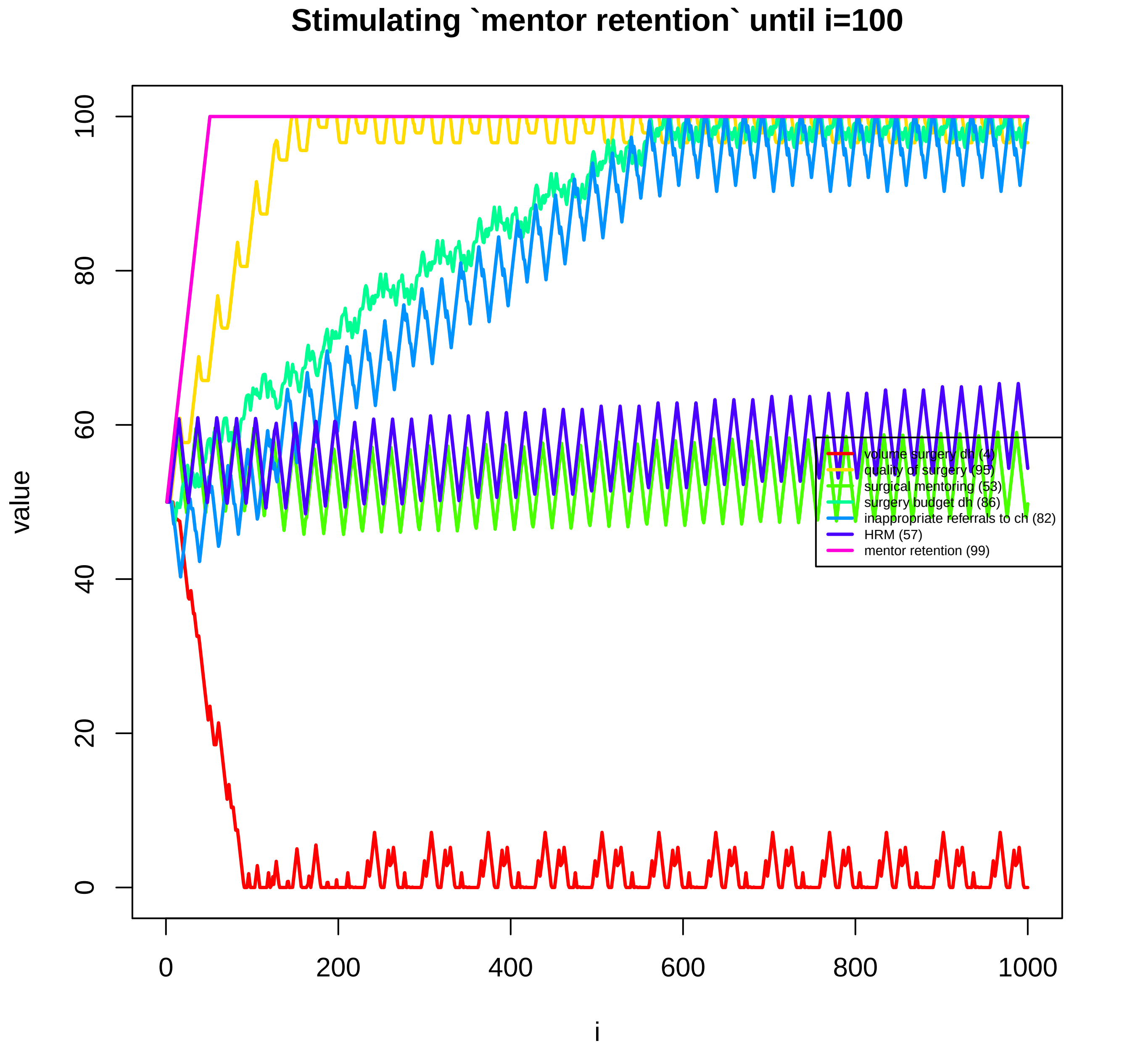

Stimulating `mentor retention` until i=100
value
i
0
20
40
60
80
100
0
200
400
600
800
1000
volume surgery dh (4)
quality of surgery (95)
surgical mentoring (53)
surgery budget dh (86)
inappropriate referrals to ch (82)
HRM (57)
mentor retention (99)

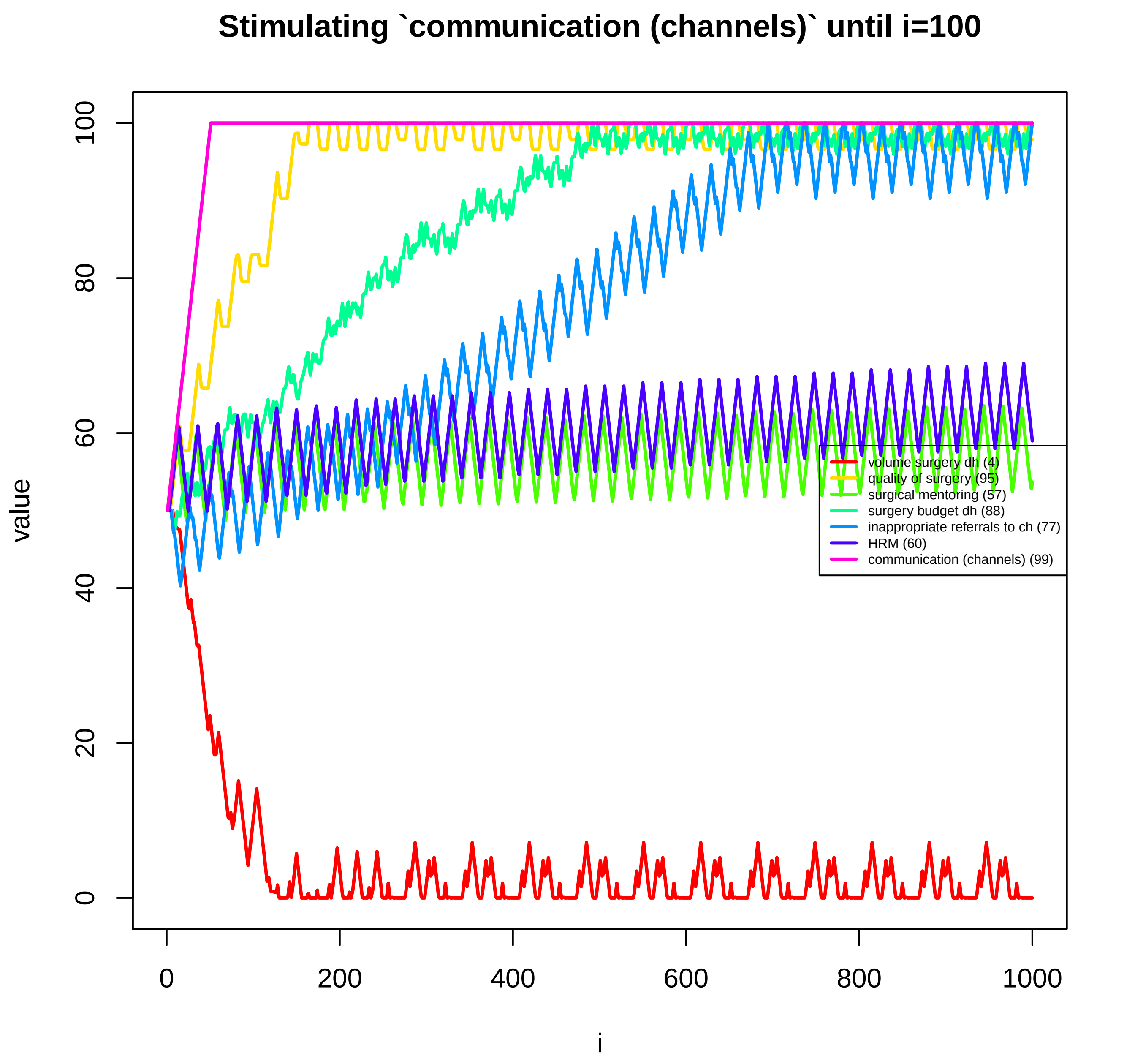

Stimulating `communication (channels)` until i=100
value
i
0
20
40
60
80
100
0
200
400
600
800
1000
volume surgery dh (4)
quality of surgery (95)
surgical mentoring (57)
surgery budget dh (88)
inappropriate referrals to ch (77)
HRM (60)
communication (channels) (99)

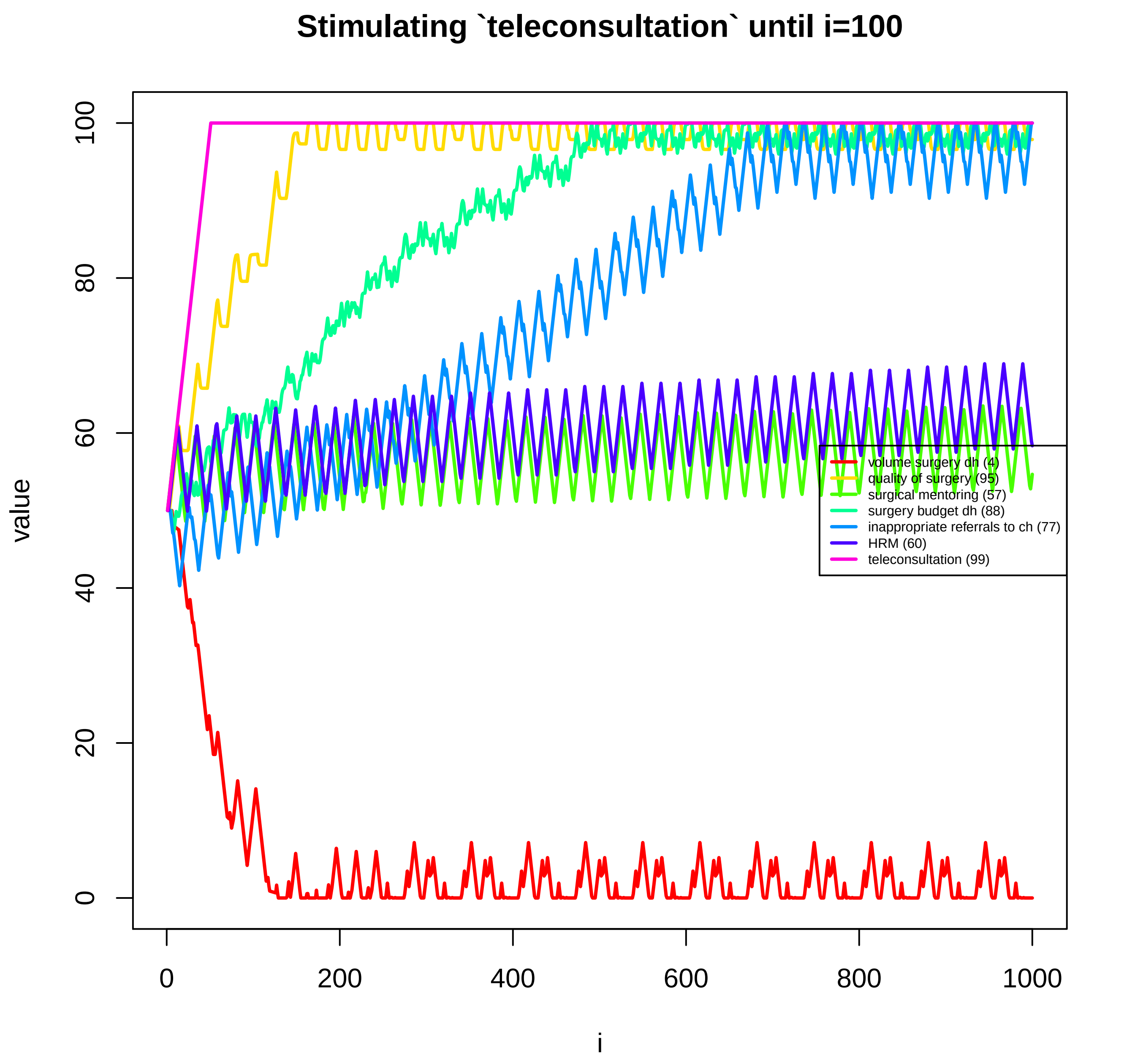

Stimulating `teleconsultation` until i=100
value
i
0
20
40
60
80
100
0
200
400
600
800
1000
volume surgery dh (4)
quality of surgery (95)
surgical mentoring (57)
surgery budget dh (88)
inappropriate referrals to ch (77)
HRM (60)
teleconsultation (99)

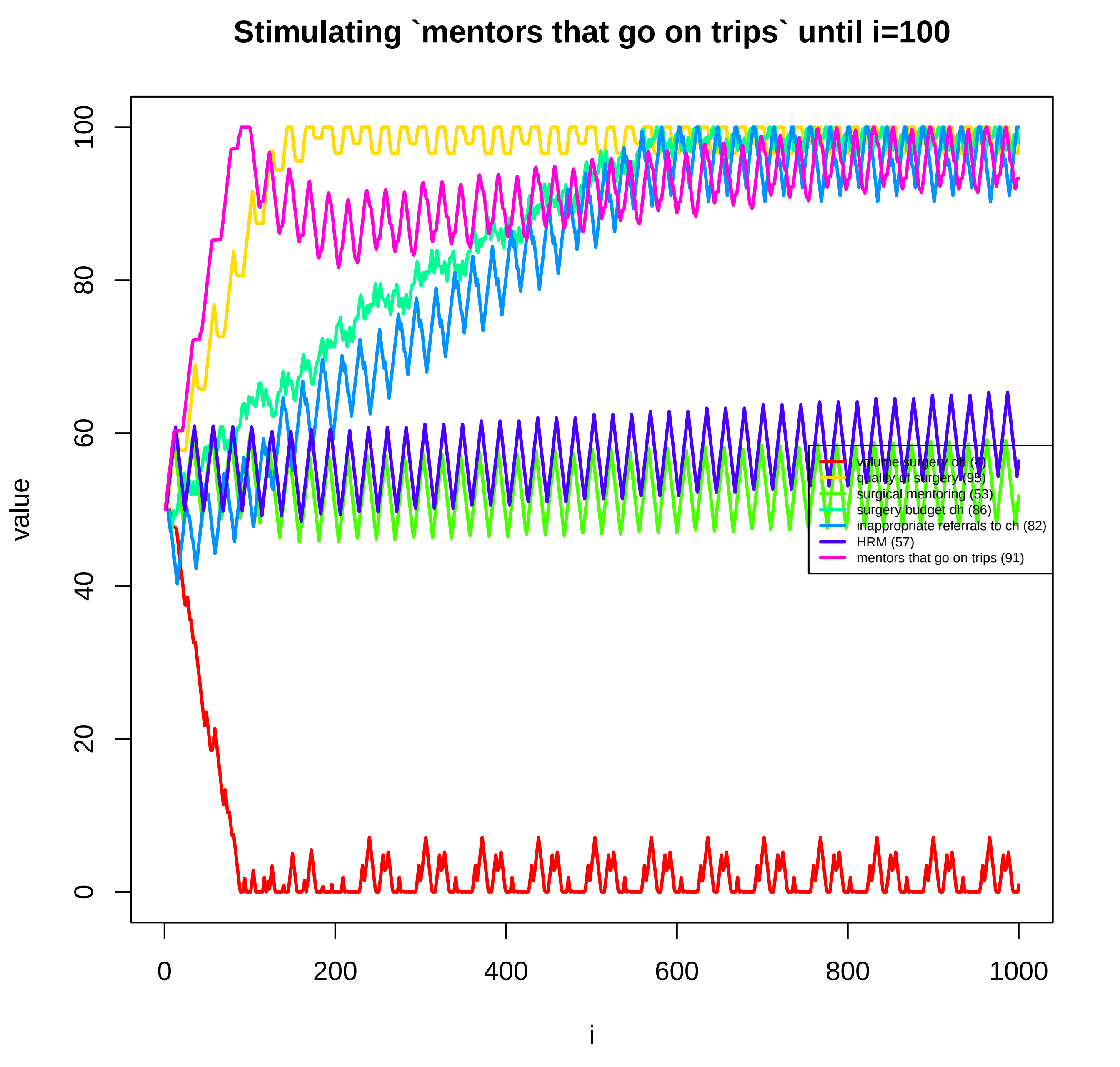
Stimulating `mentors that go on trips` until i=100
value
i
0
20
40
60
80
100
0
200
400
600
800
1000
volume surgery dh (4)
quality of surgery (95)
surgical mentoring (53)
surgery budget dh (86)
inappropriate referrals to ch (82)
HRM (57)
mentors that go on trips (91)

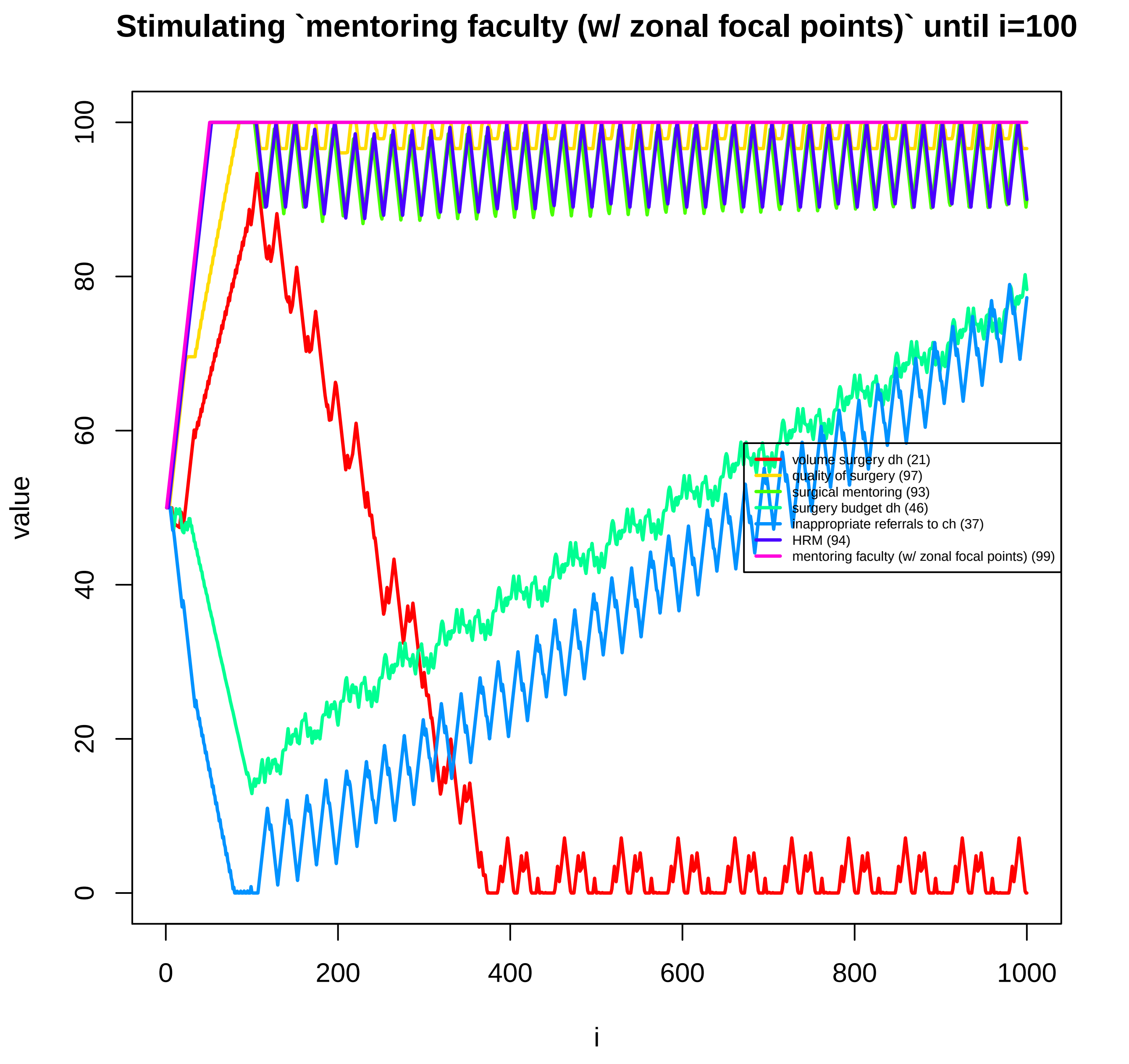

Stimulating `mentoring faculty (w/ zonal focal points)` until i=100
value
i
0
20
40
60
80
100
0
200
400
600
800
1000
volume surgery dh (21)
quality of surgery (97)
surgical mentoring (93)
surgery budget dh (46)
inappropriate referrals to ch (37)
HRM (94)
mentoring faculty (w/ zonal focal points) (99)

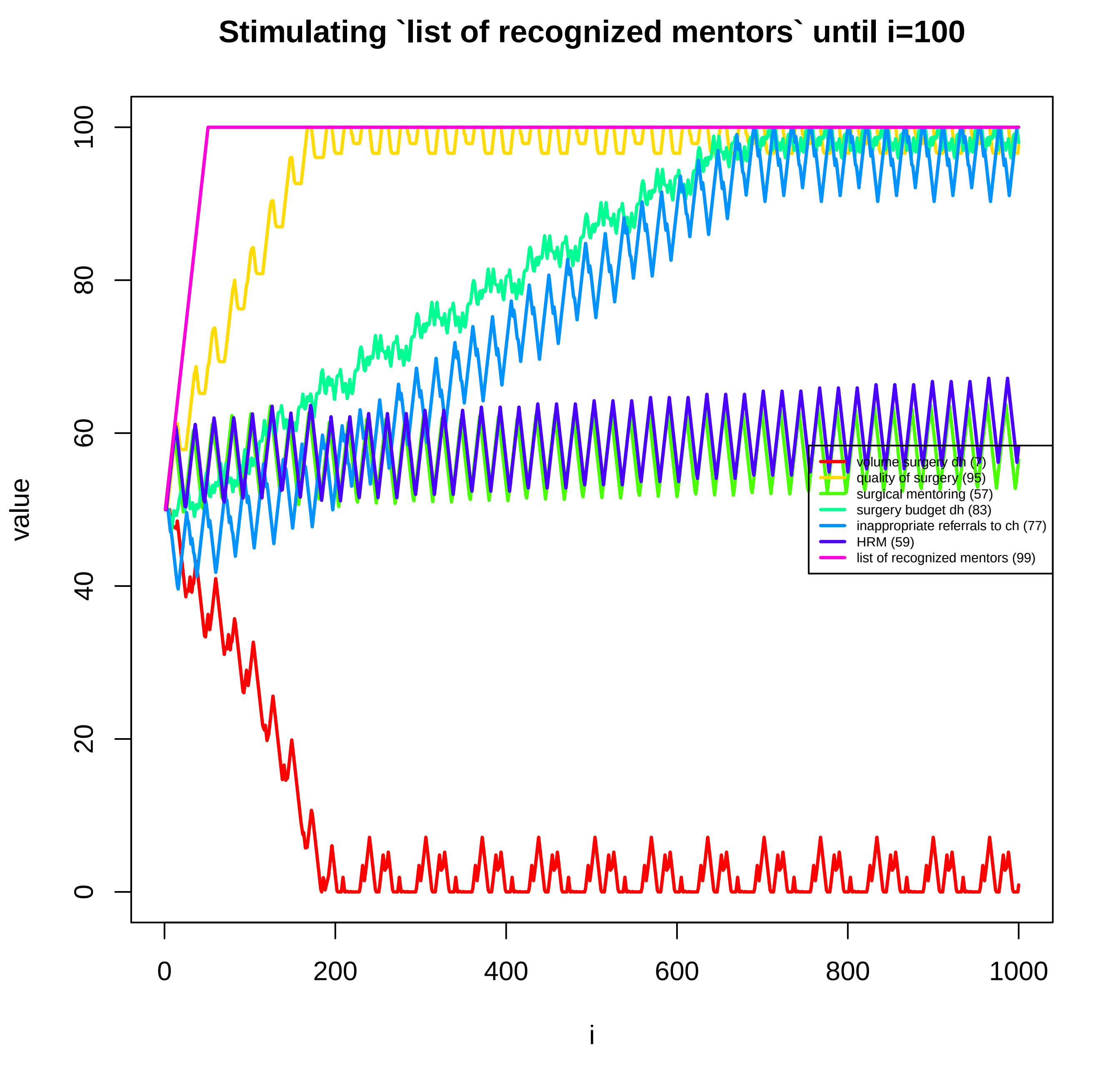

Stimulating `list of recognized mentors` until i=100
value
i
0
20
40
60
80
100
0
200
400
600
800
1000
volume surgery dh (7)
quality of surgery (95)
surgical mentoring (57)
surgery budget dh (83)
inappropriate referrals to ch (77)
HRM (59)
list of recognized mentors (99)

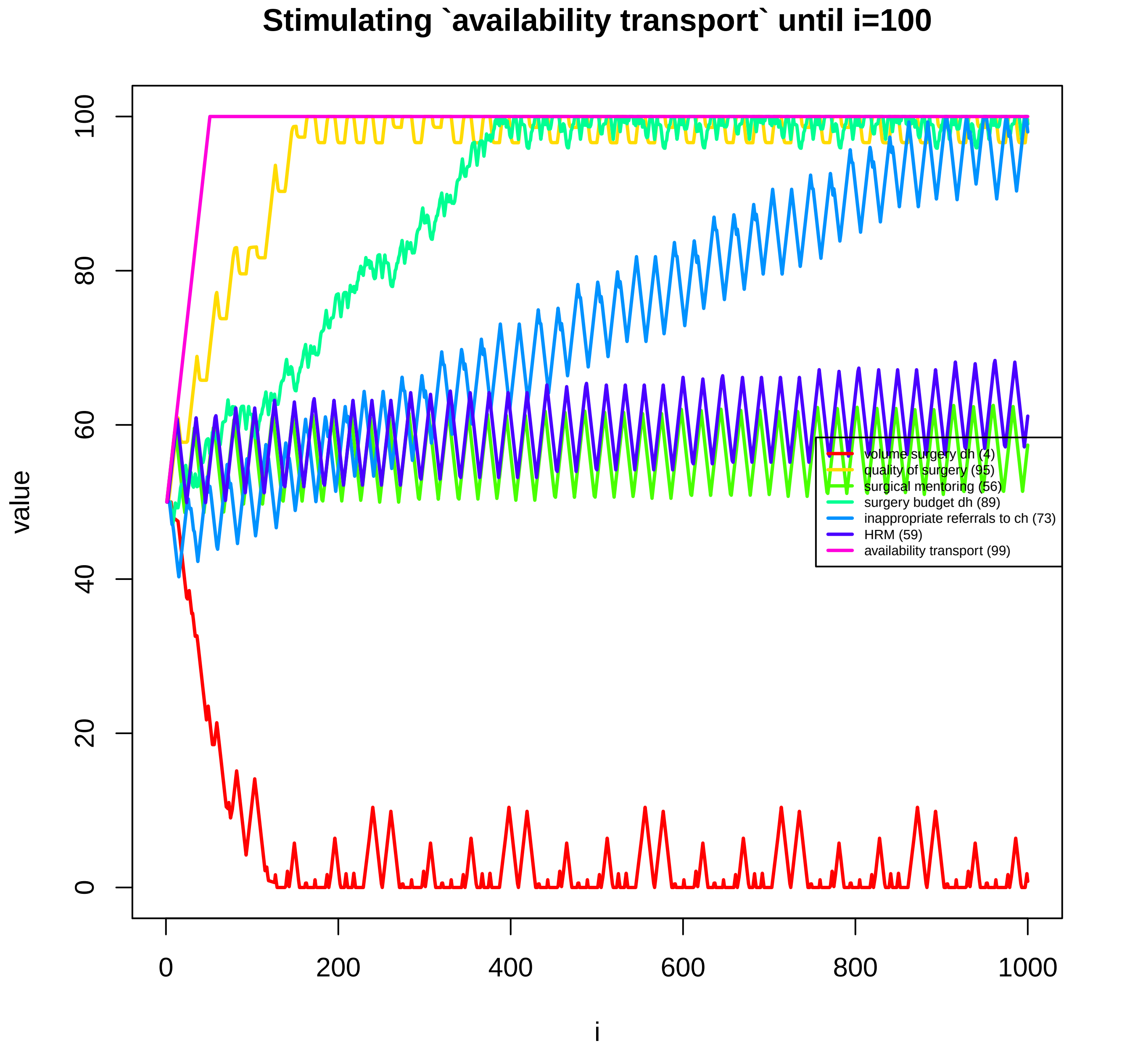
Stimulating `availability transport` until i=100
value
i
0
20
40
60
80
100
0
200
400
600
800
1000
volume surgery dh (4)
quality of surgery (95)
surgical mentoring (56)
surgery budget dh (89)
inappropriate referrals to ch (73)
HRM (59)
availability transport (99)

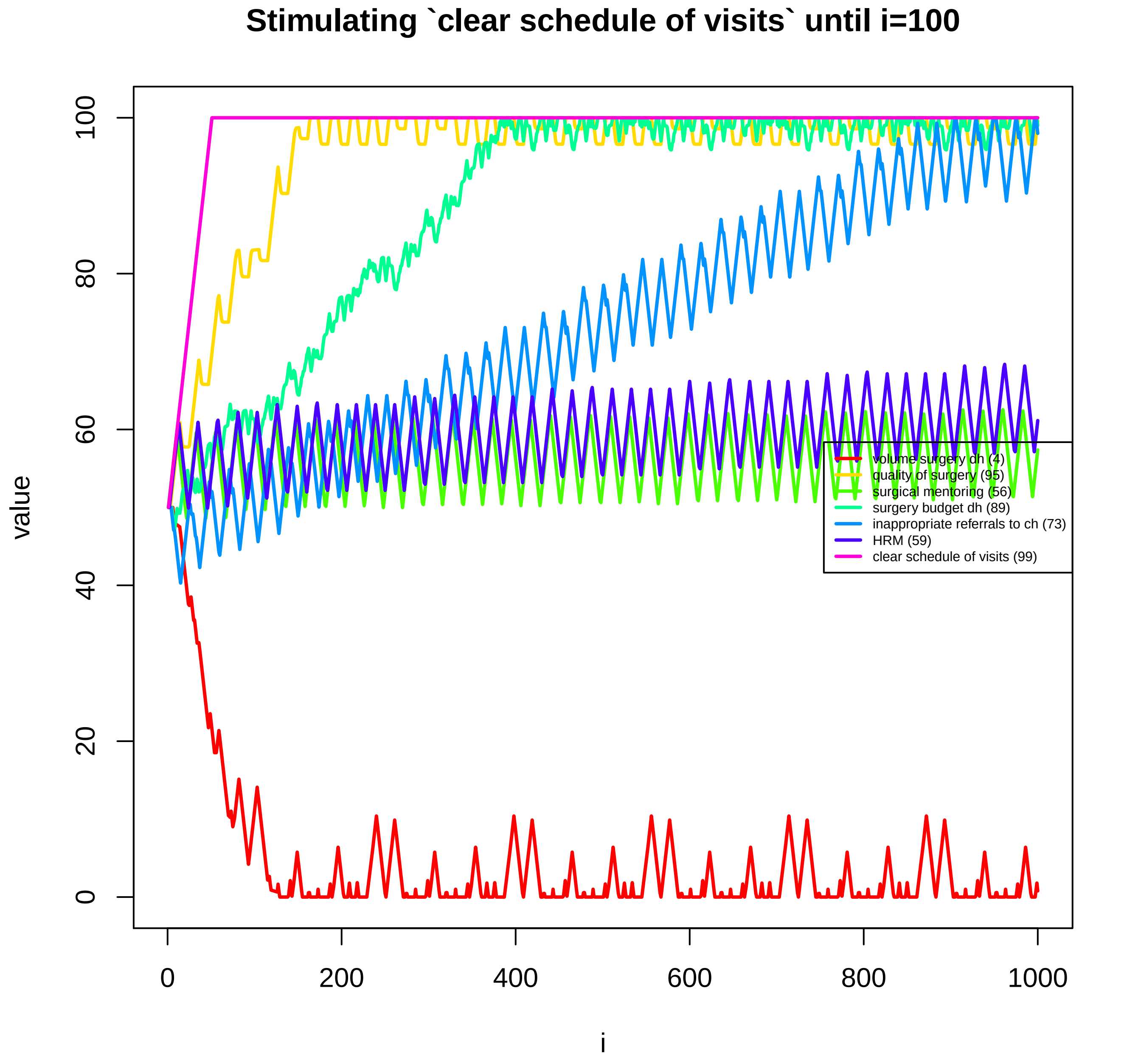

Stimulating `clear schedule of visits` until i=100
value
i
0
20
40
60
80
100
0
200
400
600
800
1000
volume surgery dh (4)
quality of surgery (95)
surgical mentoring (56)
surgery budget dh (89)
inappropriate referrals to ch (73)
HRM (59)
clear schedule of visits (99)

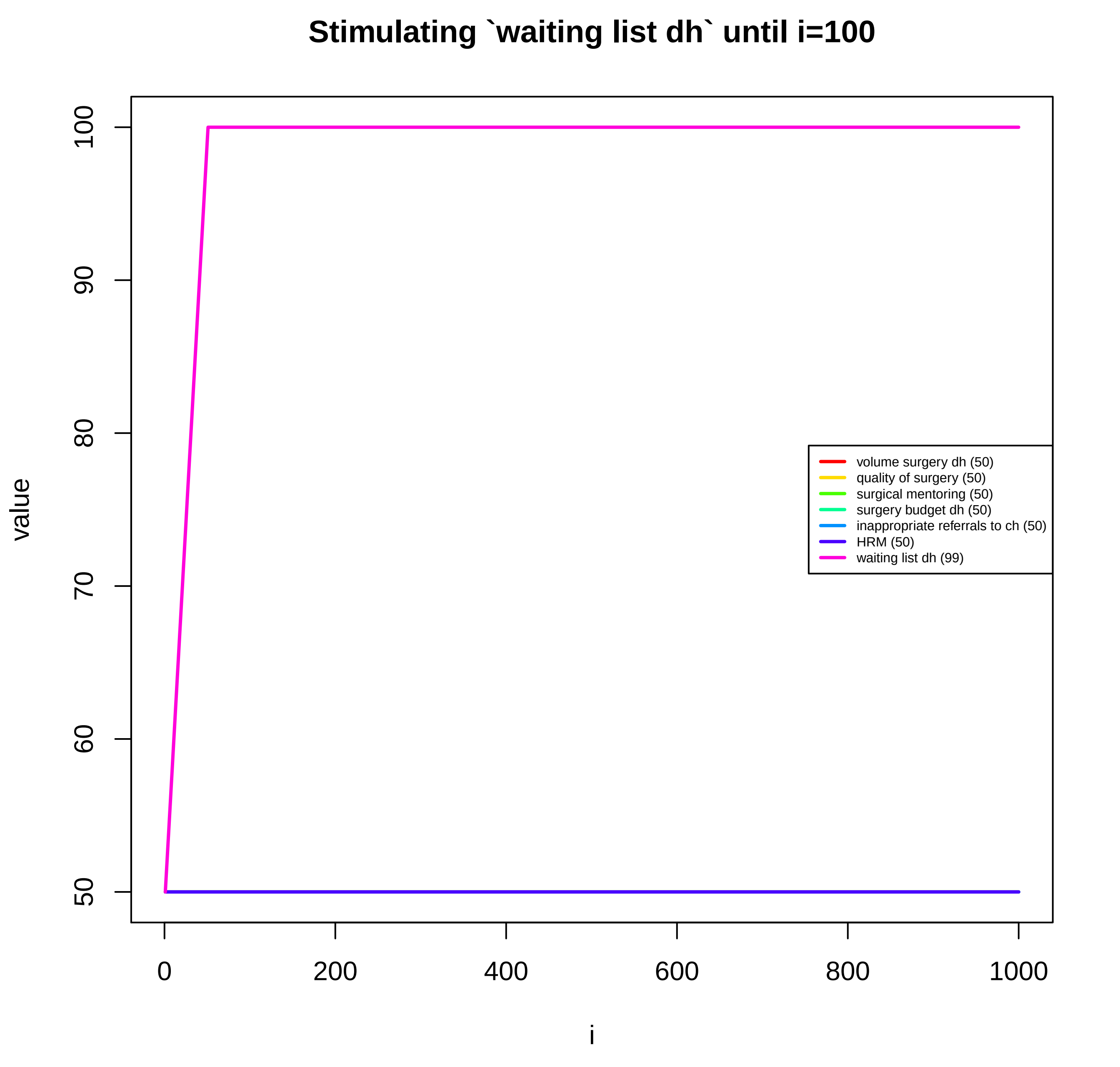
Stimulating `waiting list dh` until i=100
value
i
100
90
80
70
60
50
0
200
400
600
800
1000
volume surgery dh (50)
quality of surgery (50)
surgical mentoring (50)
surgery budget dh (50)
inappropriate referrals to ch (50)
HRM (50)
waiting list dh (99)

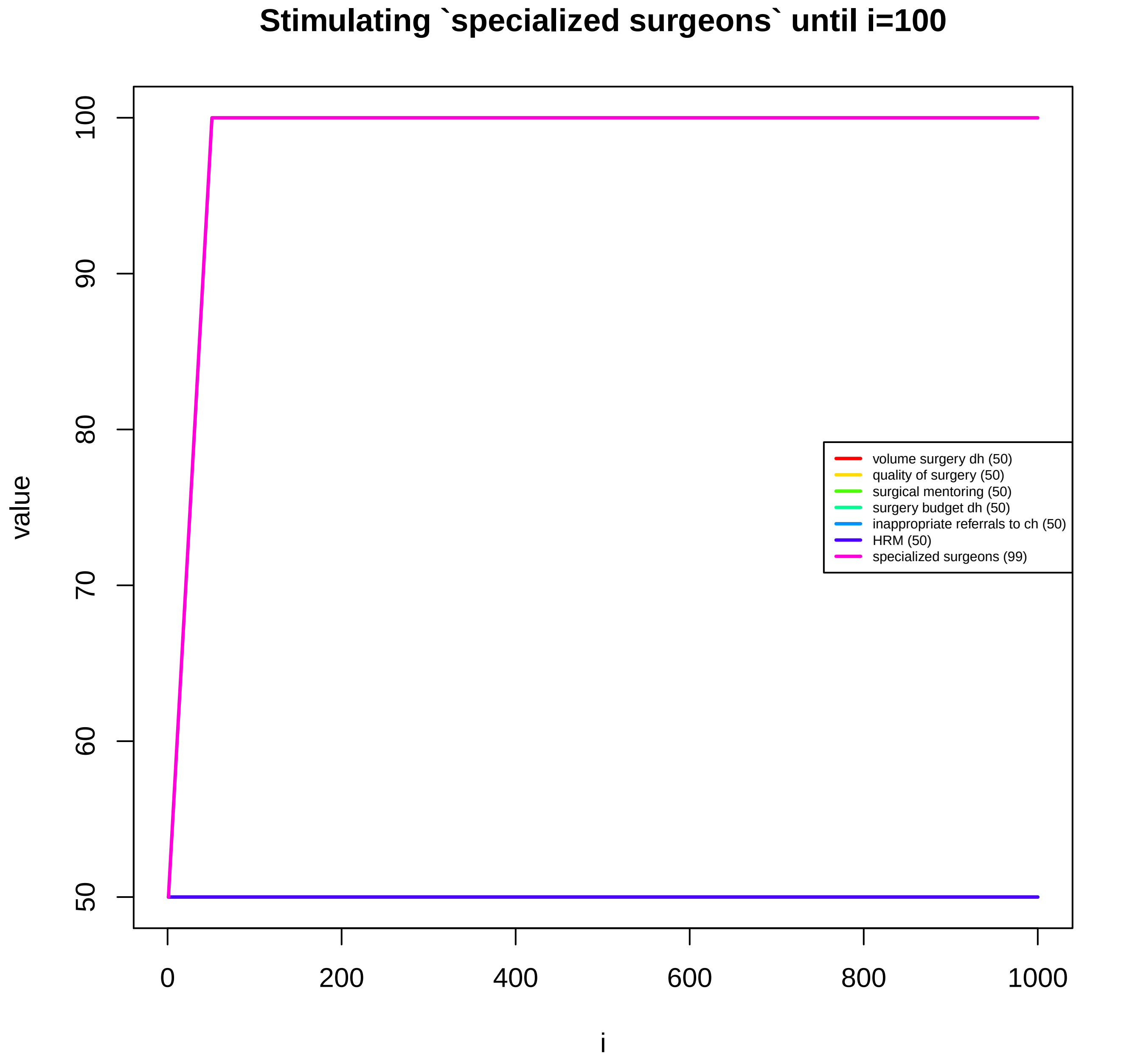

Stimulating `specialized surgeons` until i=100
value
100
90
80
70
60
50
0
200
400
600
800
1000
i
volume surgery dh (50)
quality of surgery (50)
surgical mentoring (50)
surgery budget dh (50)
inappropriate referrals to ch (50)
HRM (50)
specialized surgeons (99)

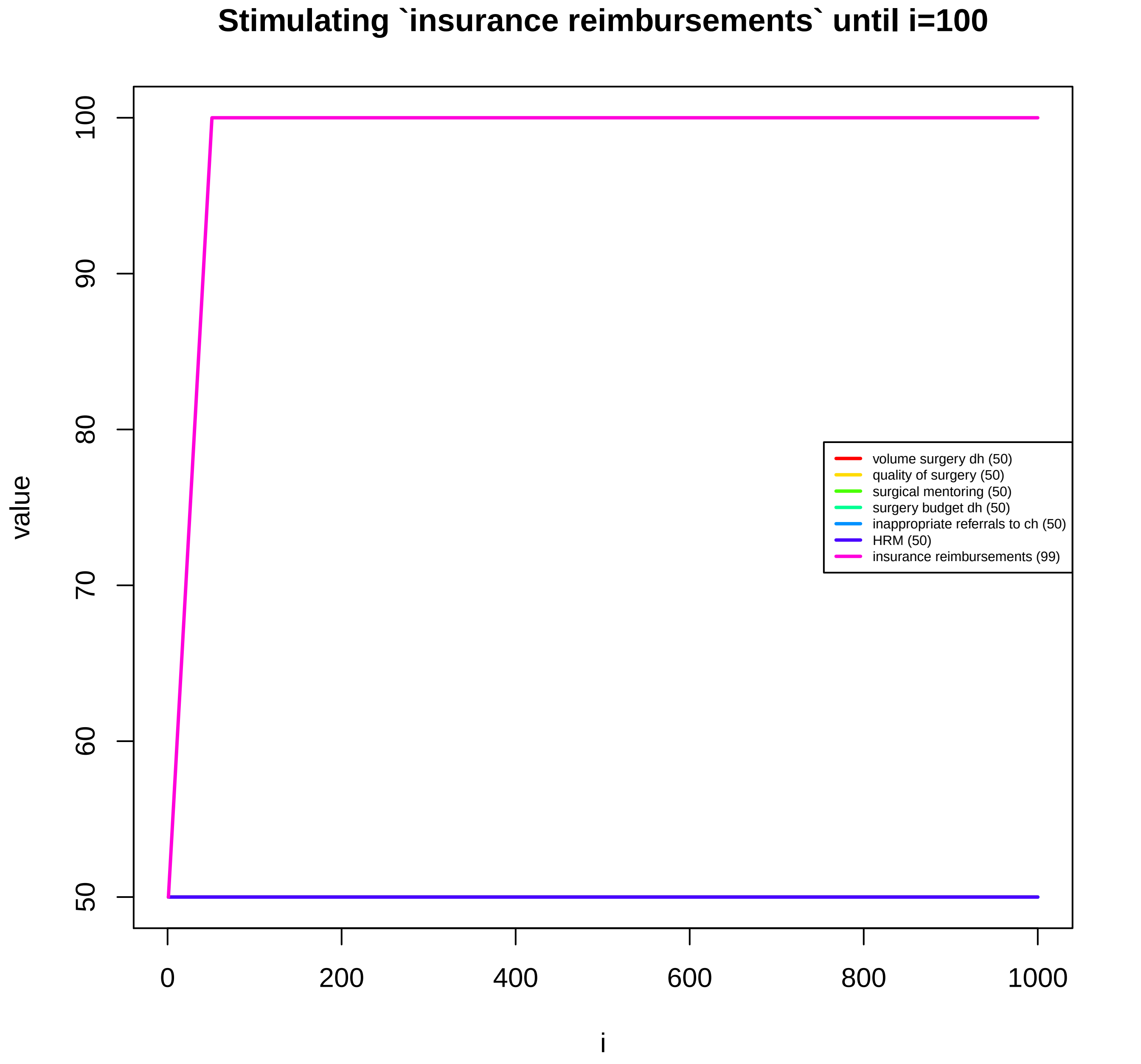
Stimulating `insurance reimbursements` until i=100
value
i
100
90
80
70
60
50
0
200
400
600
800
1000
volume surgery dh (50)
quality of surgery (50)
surgical mentoring (50)
surgery budget dh (50)
inappropriate referrals to ch (50)
HRM (50)
insurance reimbursements (99)

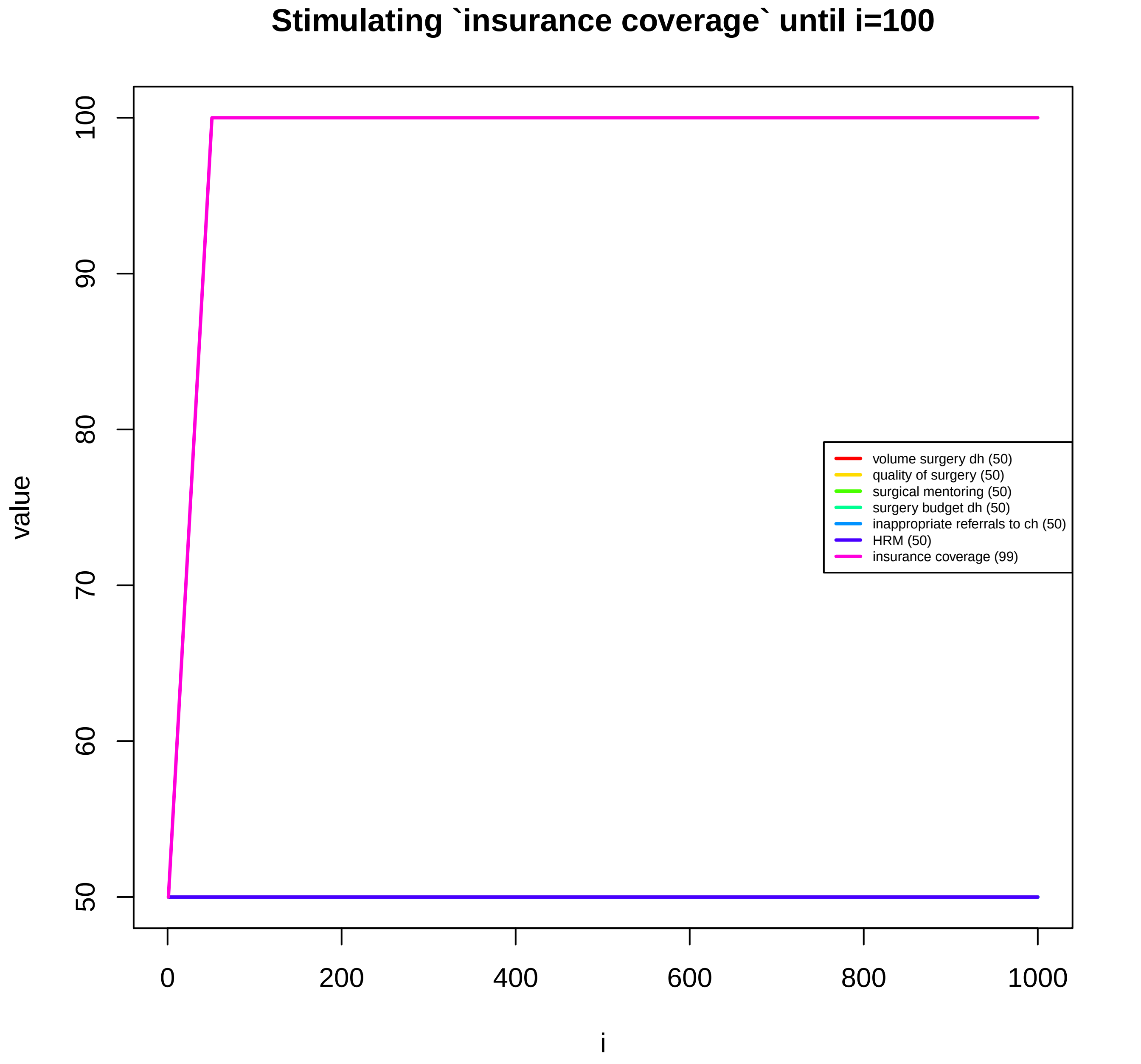

Stimulating `insurance coverage` until i=100
value
100
90
80
70
60
50
0
200
400
600
800
1000
i
volume surgery dh (50)
quality of surgery (50)
surgical mentoring (50)
surgery budget dh (50)
inappropriate referrals to ch (50)
HRM (50)
insurance coverage (99)

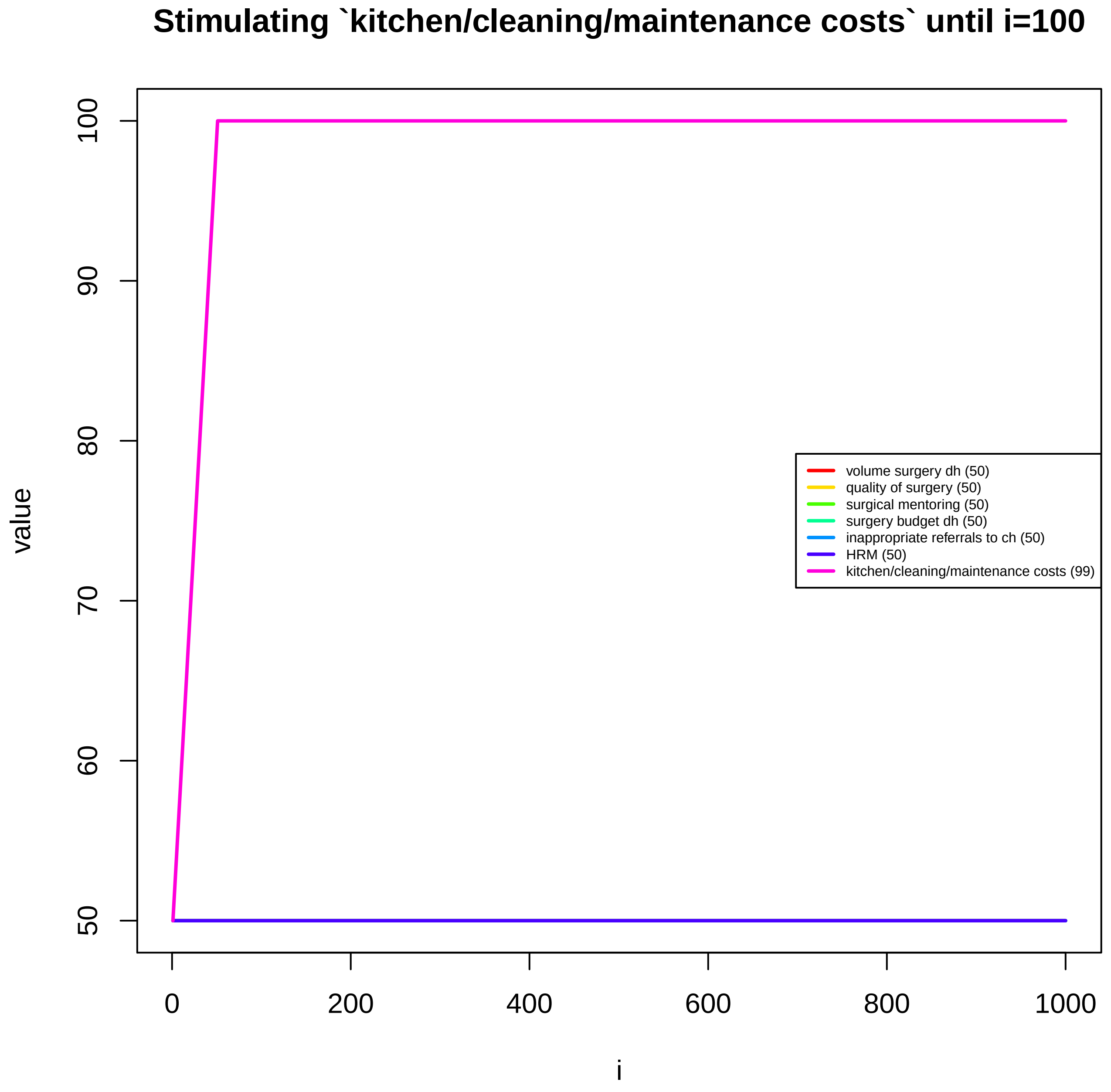
Stimulating `kitchen/cleaning/maintenance costs` until i=100
value
i
100
90
80
70
60
50
0
200
400
600
800
1000
volume surgery dh (50)
quality of surgery (50)
surgical mentoring (50)
surgery budget dh (50)
inappropriate referrals to ch (50)
HRM (50)
kitchen/cleaning/maintenance costs (99)

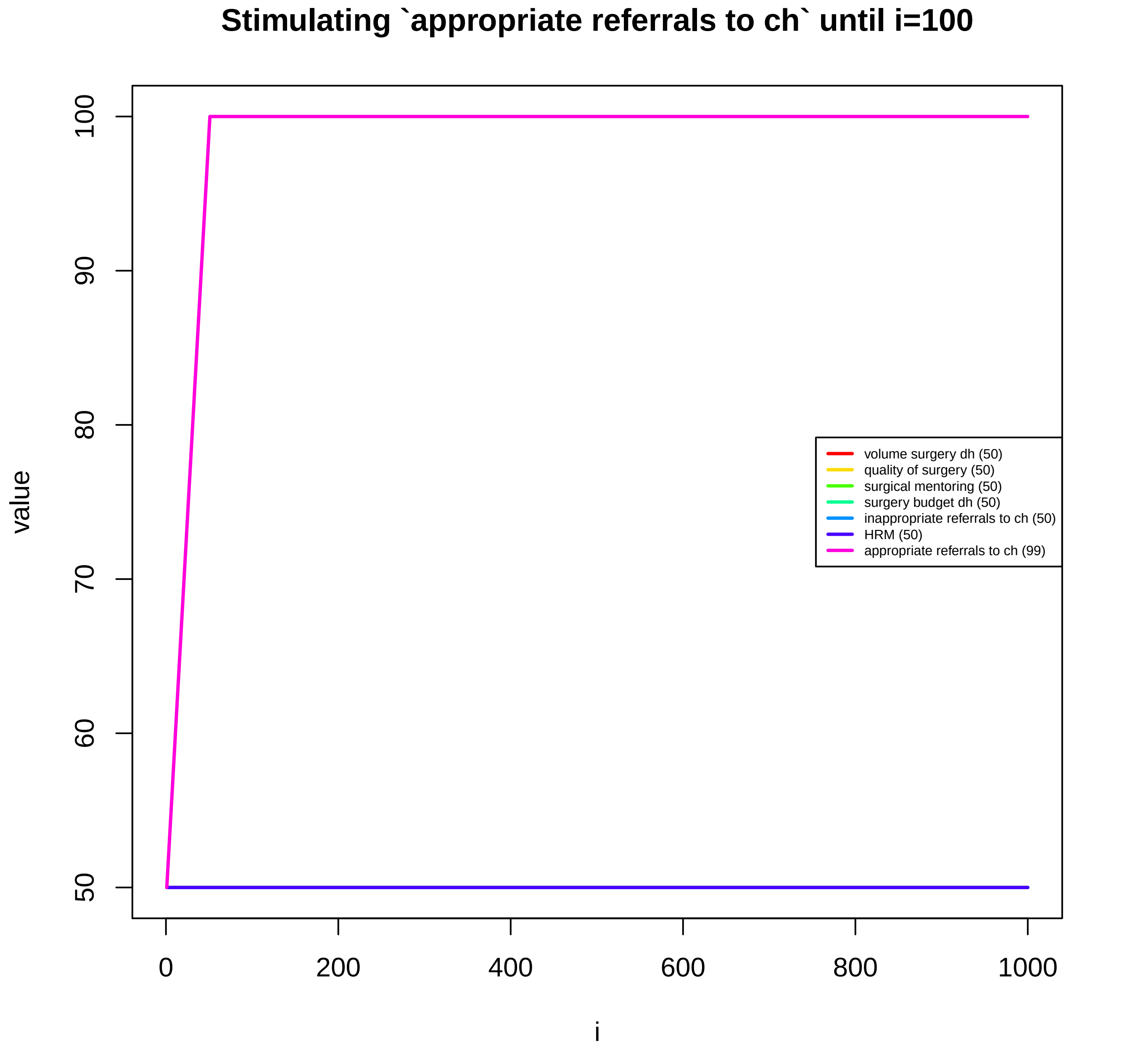
Stimulating `appropriate referrals to ch` until i=100
value
100
90
80
70
60
50
0
200
400
600
800
1000
i
volume surgery dh (50)
quality of surgery (50)
surgical mentoring (50)
surgery budget dh (50)
inappropriate referrals to ch (50)
HRM (50)
appropriate referrals to ch (99)

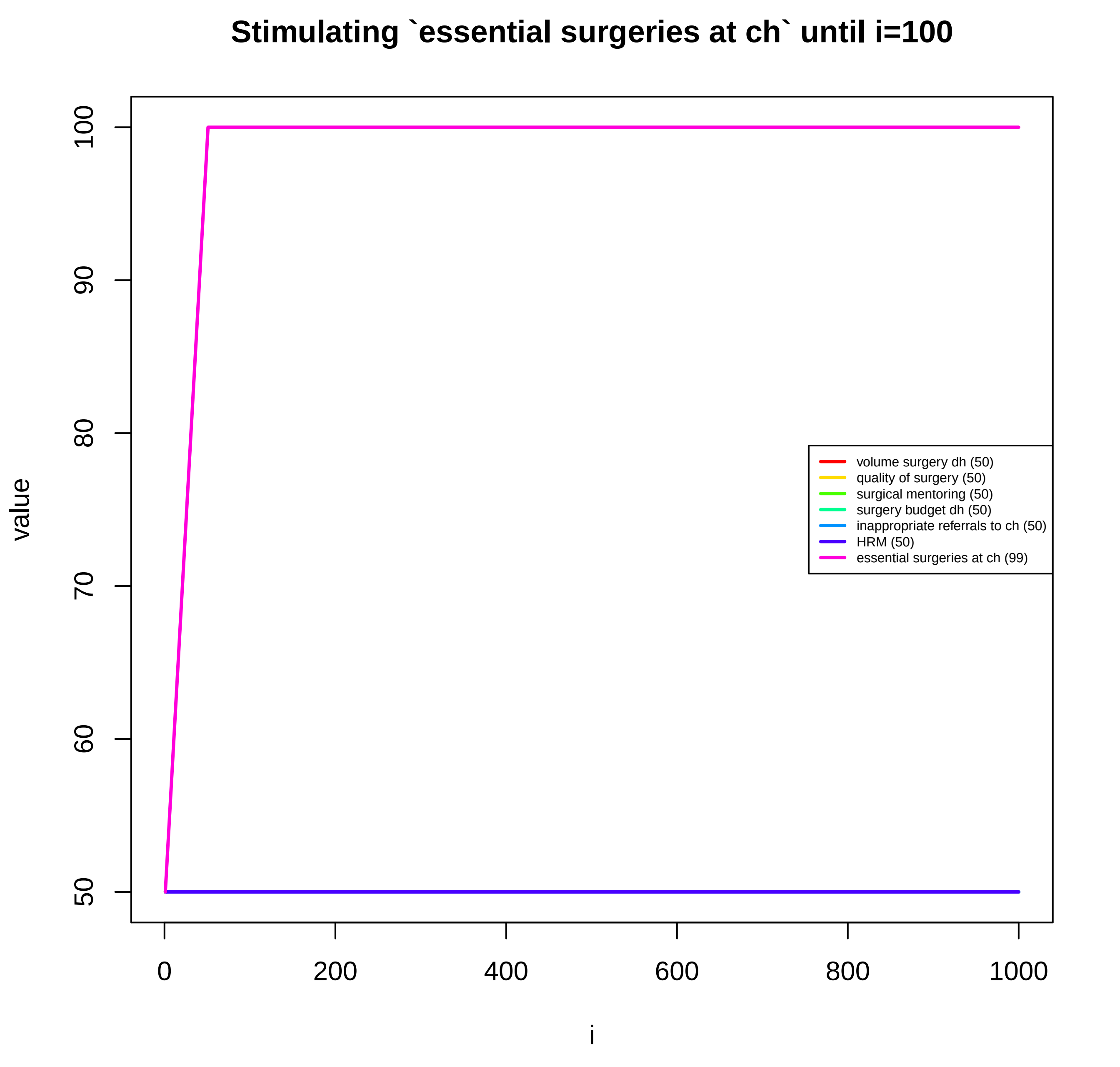

Stimulating `essential surgeries at ch` until i=100
value
100
90
80
70
60
50
0
200
400
600
800
1000
i
volume surgery dh (50)
quality of surgery (50)
surgical mentoring (50)
surgery budget dh (50)
inappropriate referrals to ch (50)
HRM (50)
essential surgeries at ch (99)

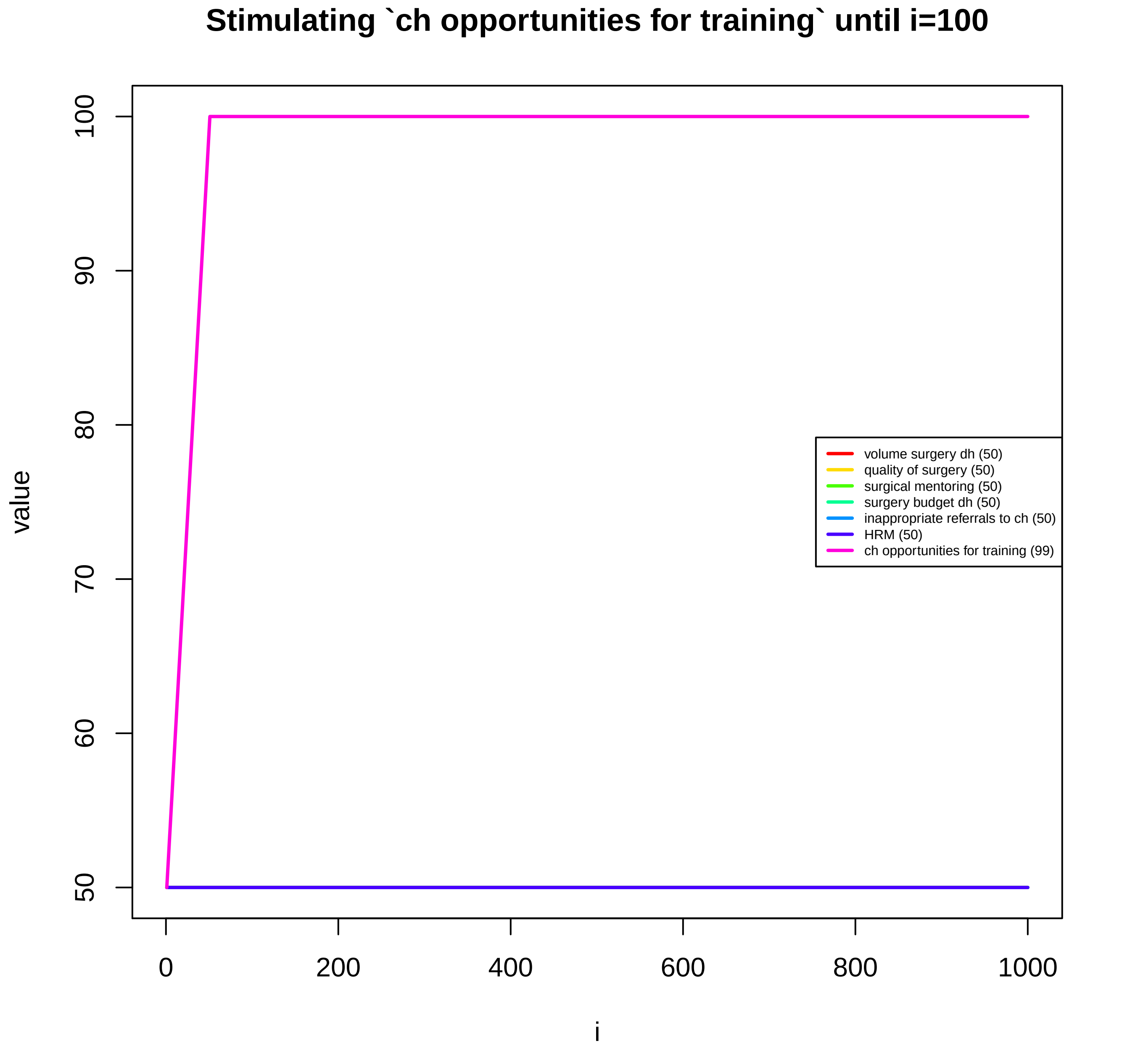
Stimulating `ch opportunities for training` until i=100
value
i
100
90
80
70
60
50
0
200
400
600
800
1000
volume surgery dh (50)
quality of surgery (50)
surgical mentoring (50)
surgery budget dh (50)
inappropriate referrals to ch (50)
HRM (50)
ch opportunities for training (99)

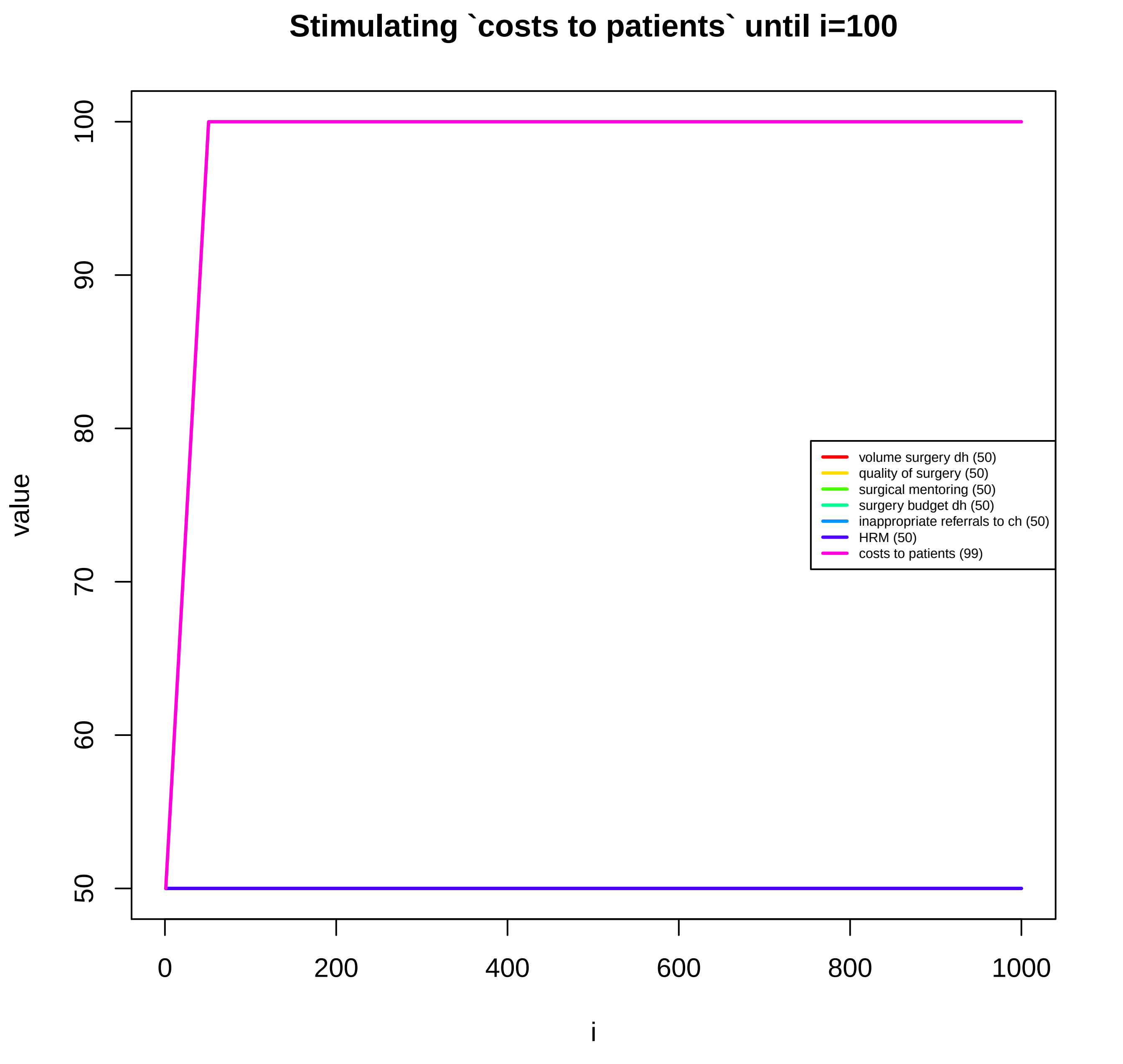

Stimulating `costs to patients` until i=100
value
i
0
200
400
600
800
1000
50
60
70
80
90
100
volume surgery dh (50)
quality of surgery (50)
surgical mentoring (50)
surgery budget dh (50)
inappropriate referrals to ch (50)
HRM (50)
costs to patients (99)

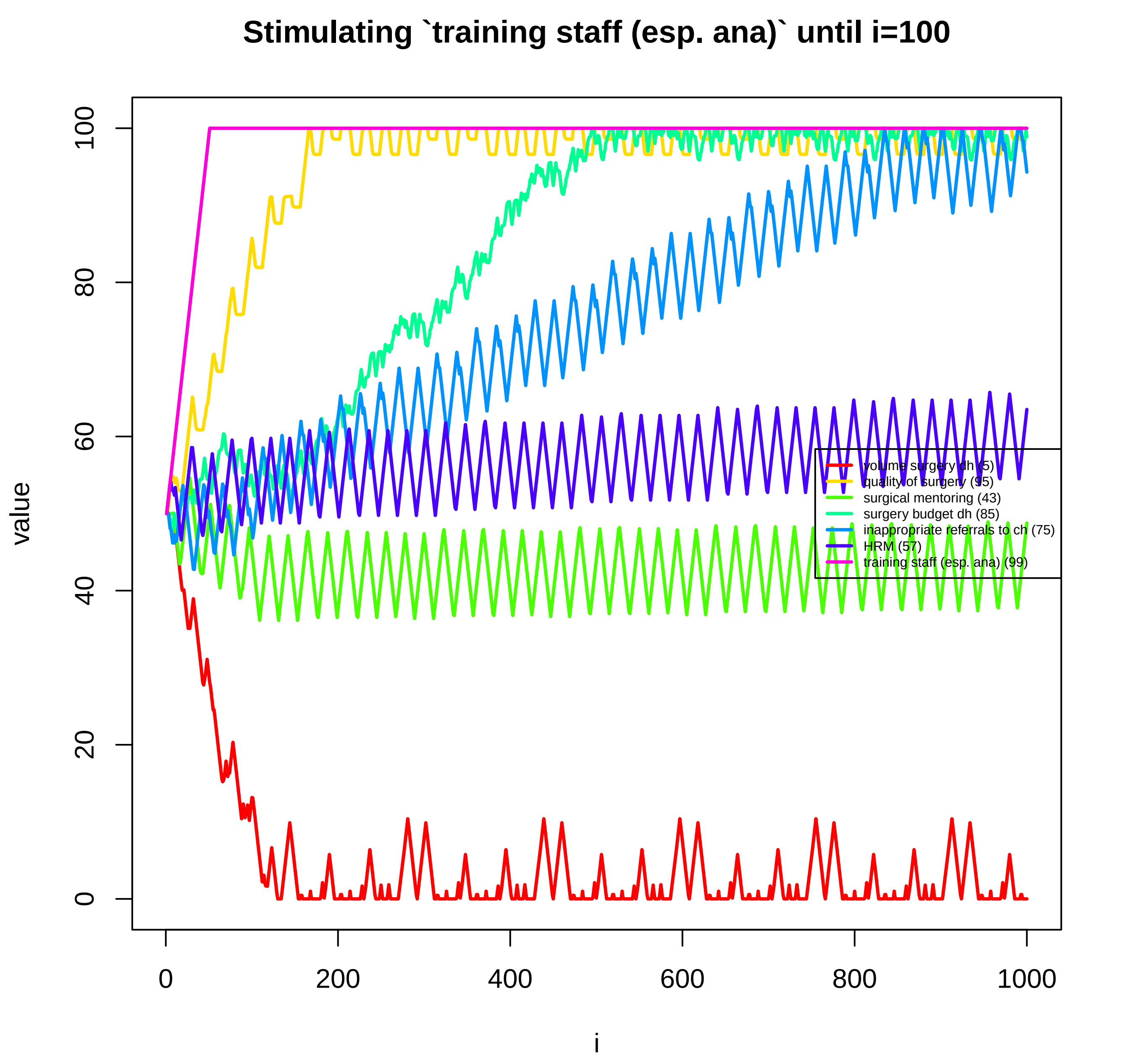
Stimulating `training staff (esp. ana)` until i=100
value
i
0
20
40
60
80
100
0
200
400
600
800
1000
volume surgery dh (5)
quality of surgery (95)
surgical mentoring (43)
surgery budget dh (85)
inappropriate referrals to ch (75)
HRM (57)
training staff (esp. ana) (99)

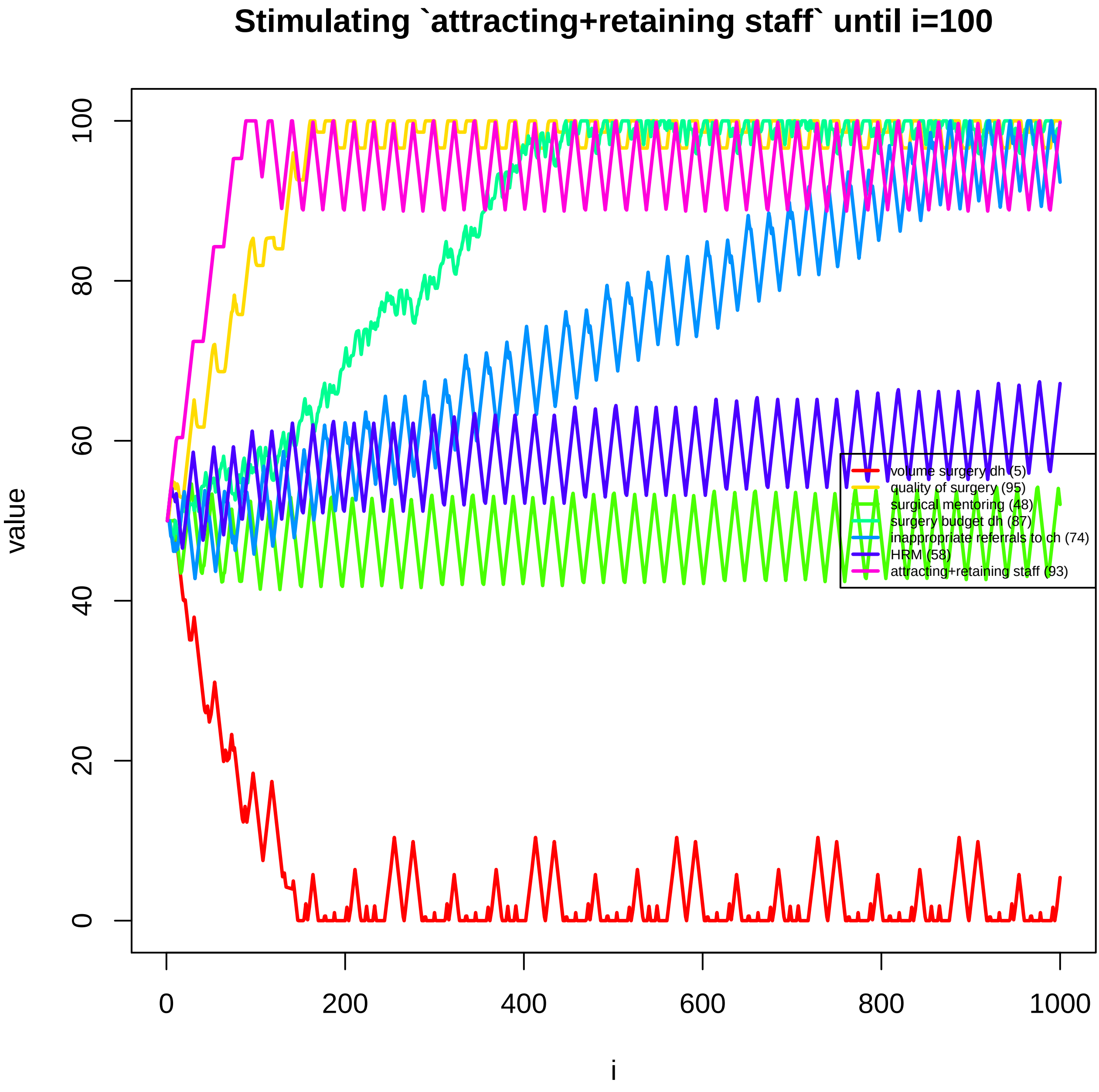
Stimulating `attracting+retaining staff` until i=100
value
i
0
20
40
60
80
100
0
200
400
600
800
1000
volume surgery dh (5)
quality of surgery (95)
surgical mentoring (48)
surgery budget dh (87)
inappropriate referrals to ch (74)
HRM (58)
attracting+retaining staff (93)

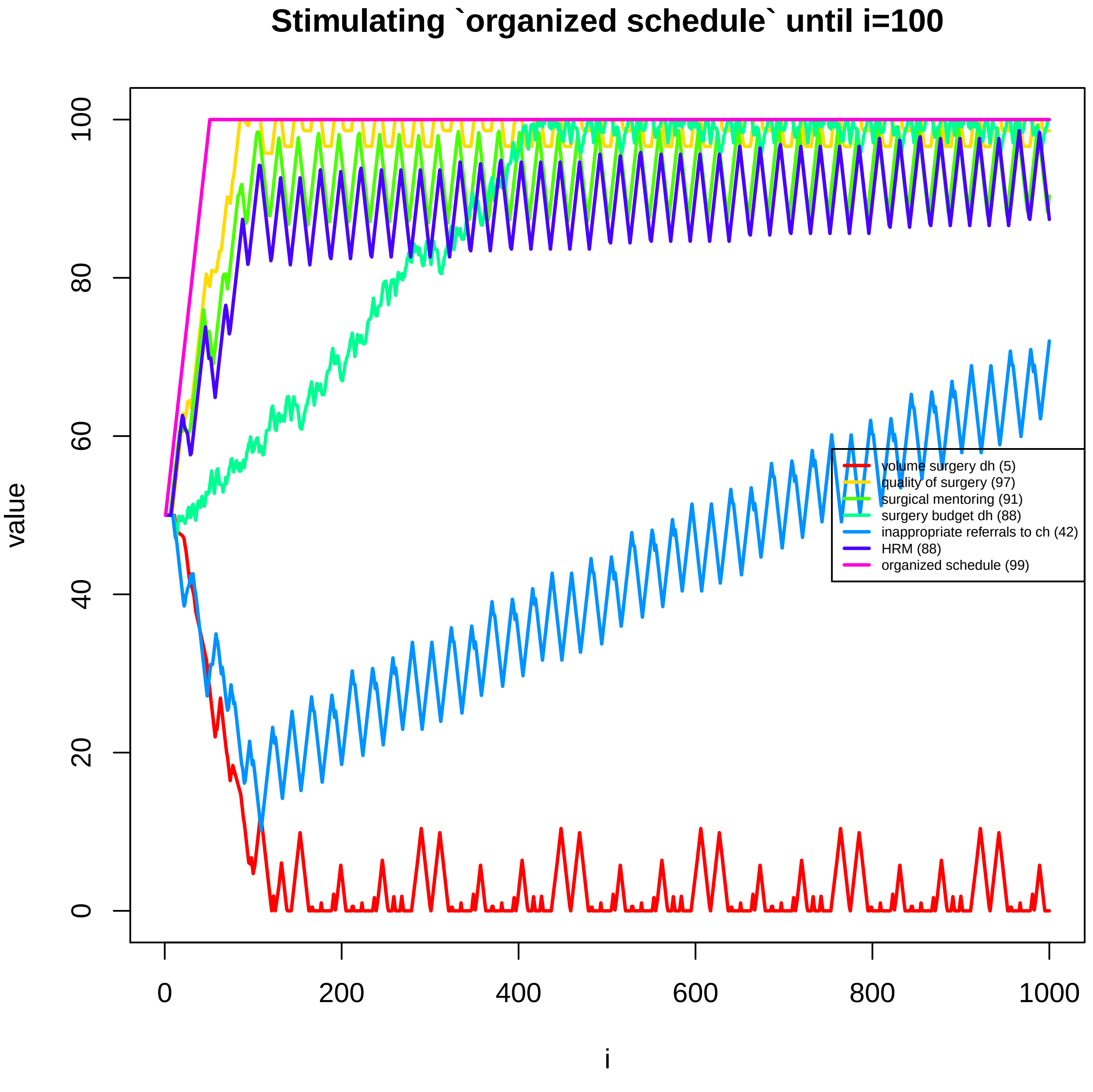

Stimulating `organized schedule` until i=100
value
i
0
20
40
60
80
100
0
200
400
600
800
1000
volume surgery dh (5)
quality of surgery (97)
surgical mentoring (91)
surgery budget dh (88)
inappropriate referrals to ch (42)
HRM (88)
organized schedule (99)

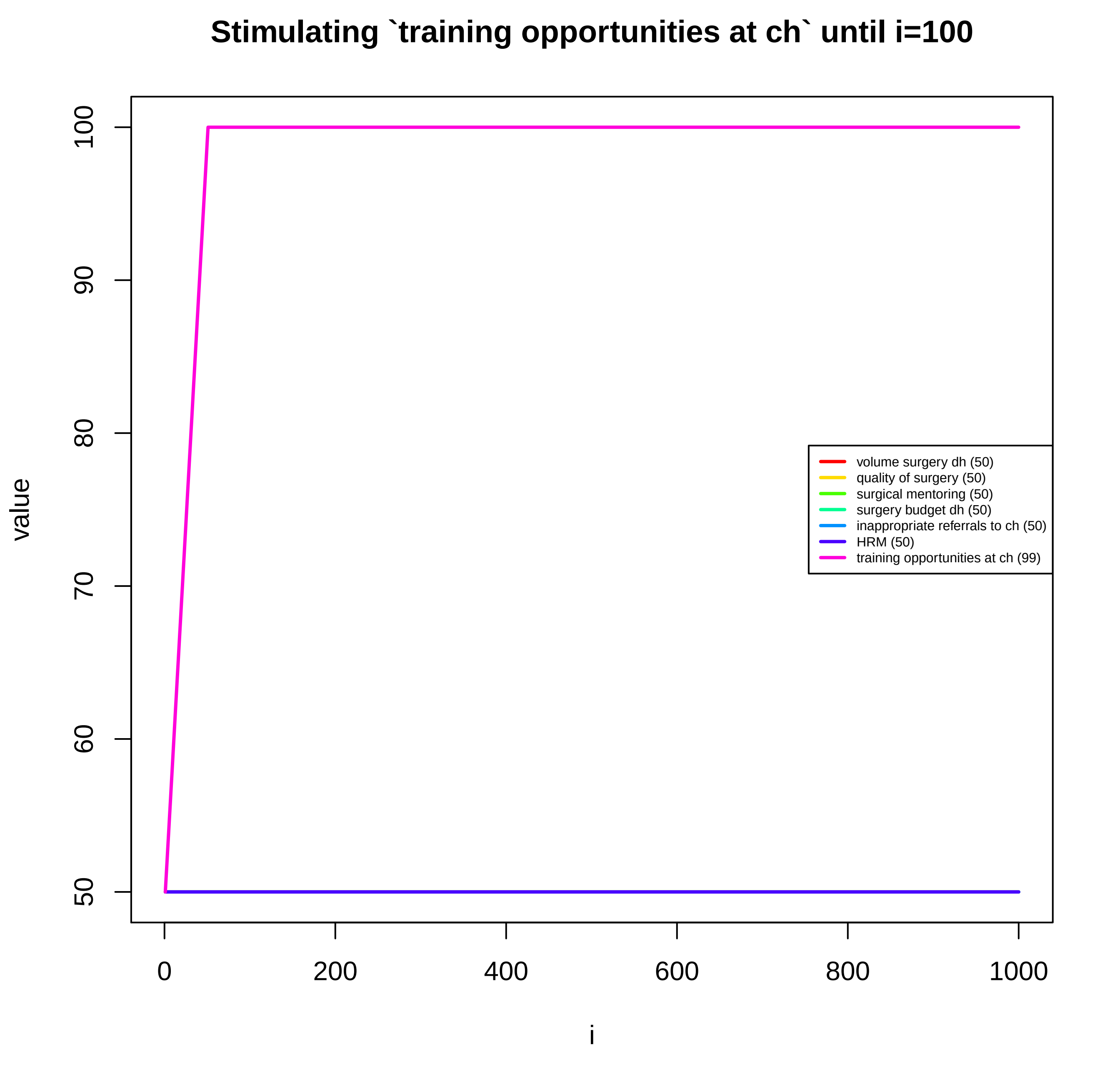
Stimulating `training opportunities at ch` until i=100
value
i
100
90
80
70
60
50
0
200
400
600
800
1000
volume surgery dh (50)
quality of surgery (50)
surgical mentoring (50)
surgery budget dh (50)
inappropriate referrals to ch (50)
HRM (50)
training opportunities at ch (99)

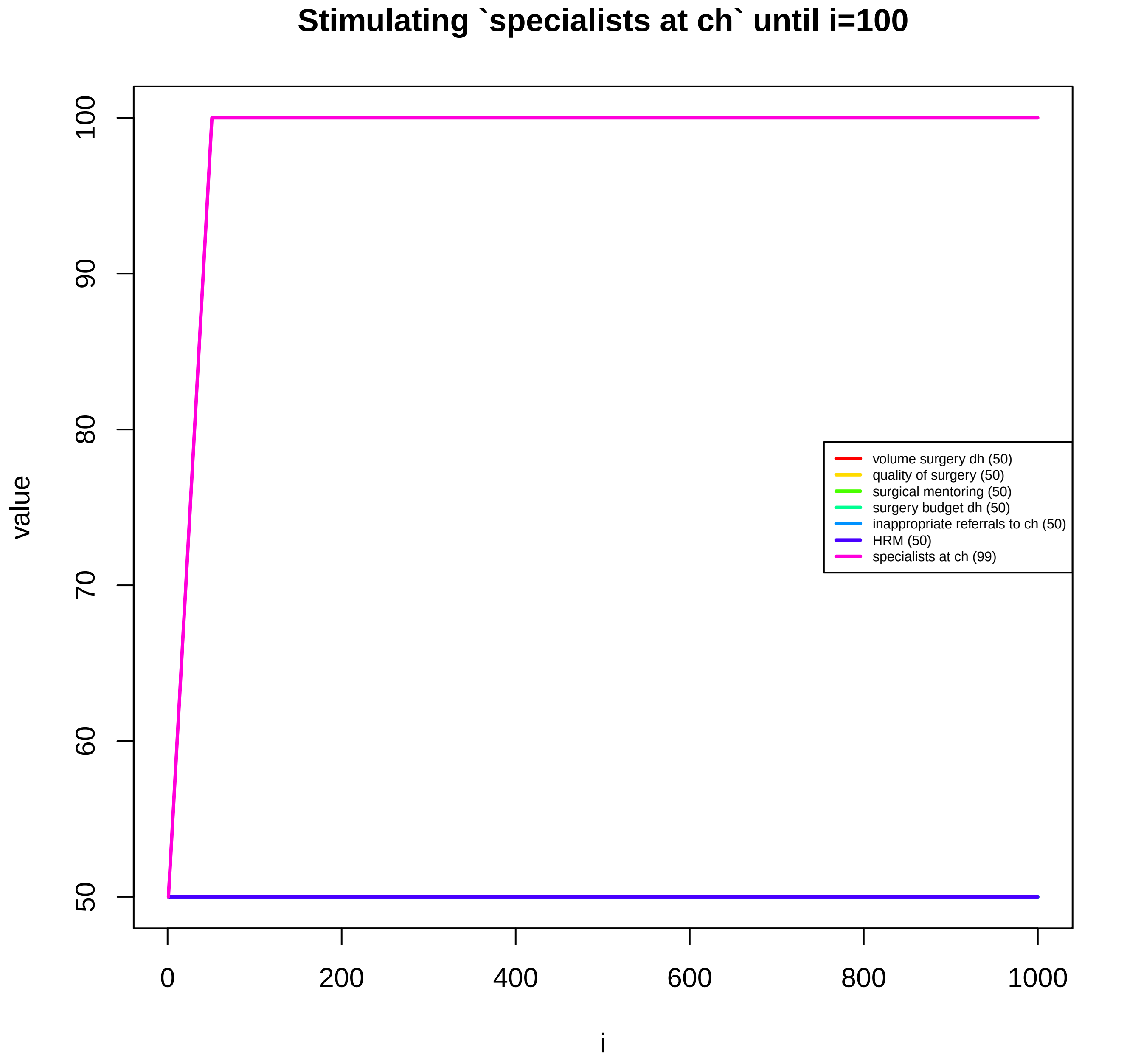

Stimulating `specialists at ch` until i=100
value
i
100
90
80
70
60
50
0
200
400
600
800
1000
volume surgery dh (50)
quality of surgery (50)
surgical mentoring (50)
surgery budget dh (50)
inappropriate referrals to ch (50)
HRM (50)
specialists at ch (99)

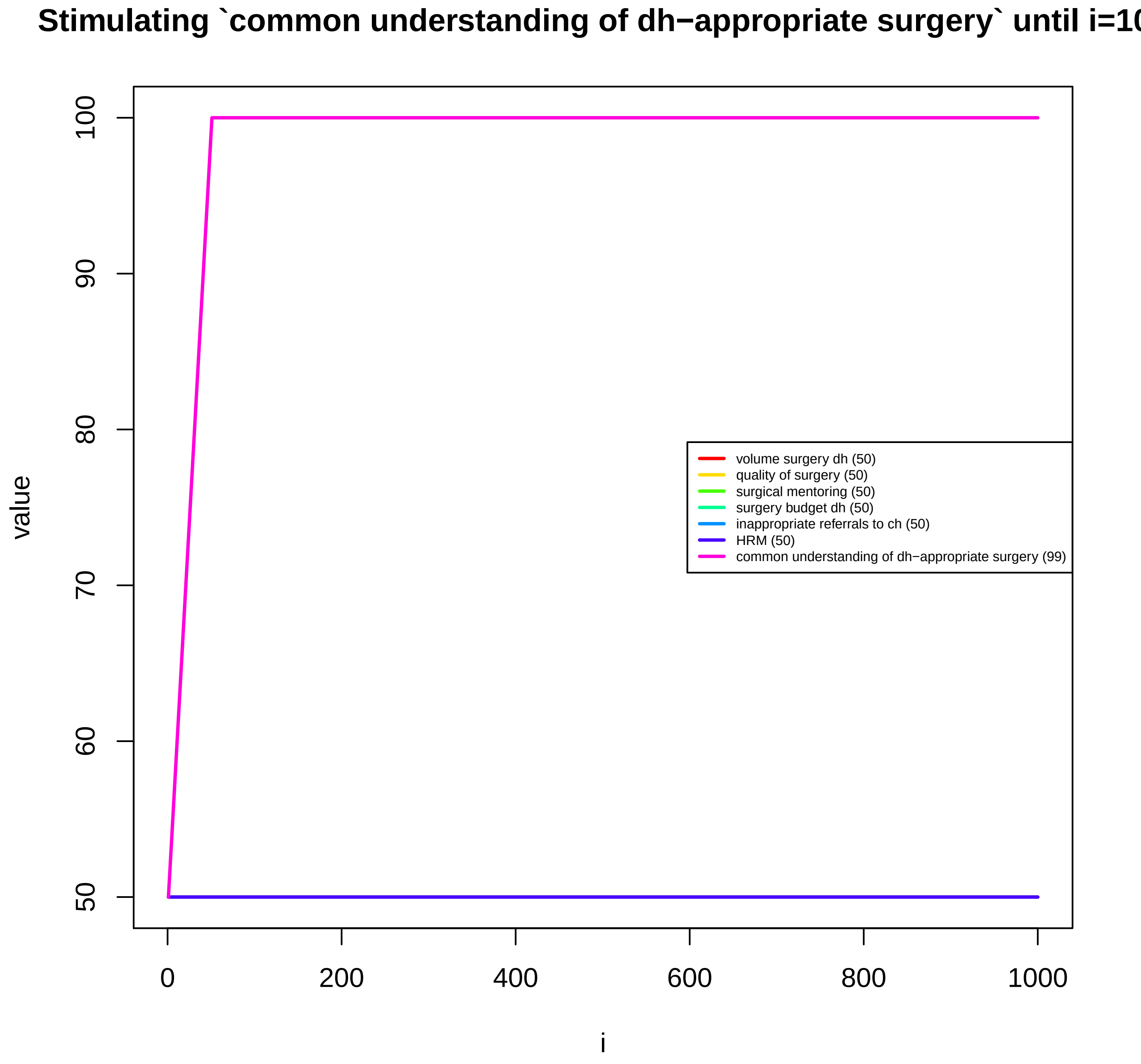
Stimulating `common understanding of dh−appropriate surgery` until i=10
100
90
80
70
60
50
value
0
200
400
600
800
1000
i
volume surgery dh (50)
quality of surgery (50)
surgical mentoring (50)
surgery budget dh (50)
inappropriate referrals to ch (50)
HRM (50)
common understanding of dh−appropriate surgery (99)

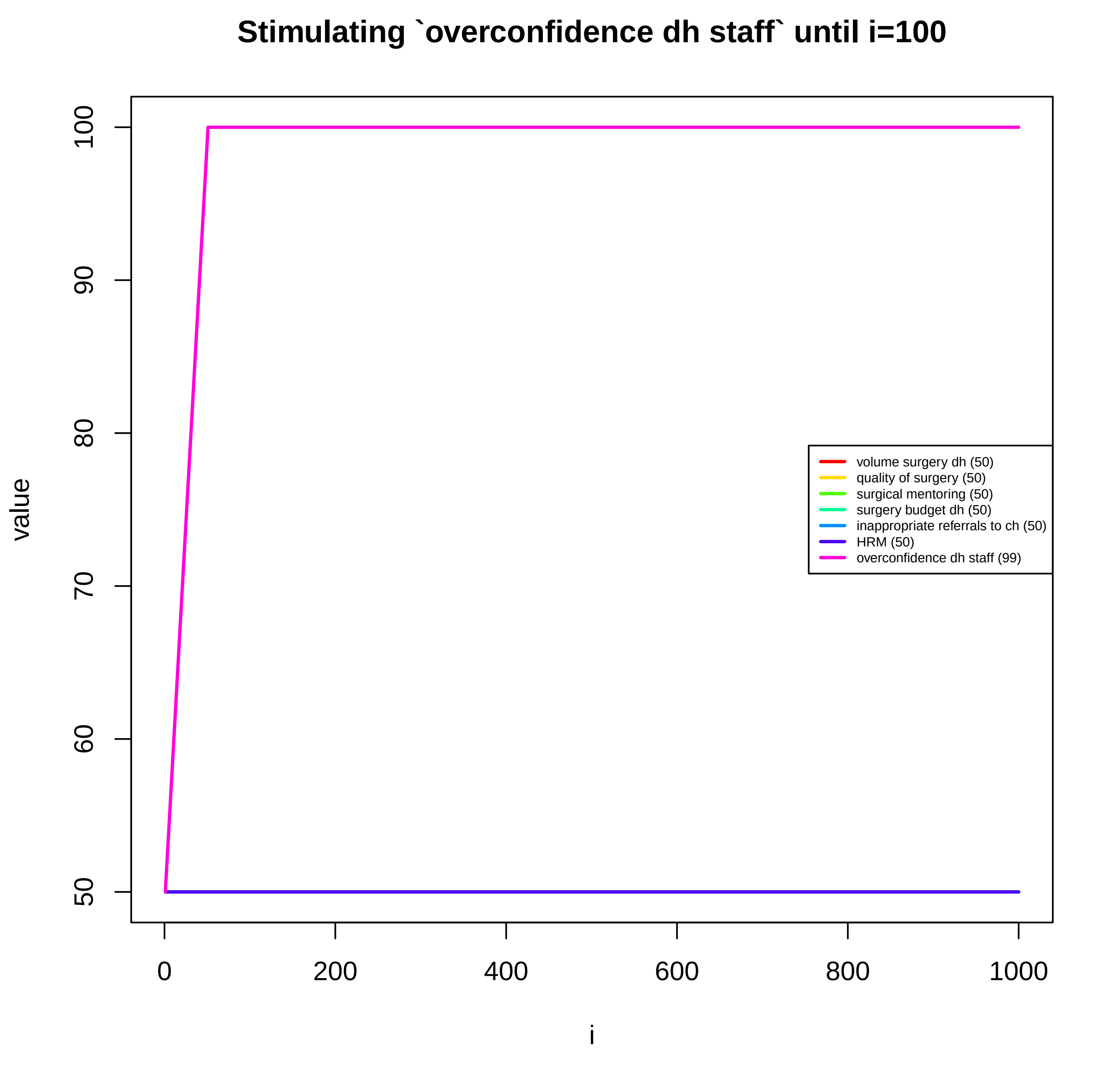
Stimulating `overconfidence dh staff` until i=100
value
100
90
80
70
60
50
0
200
400
600
800
1000
i
volume surgery dh (50)
quality of surgery (50)
surgical mentoring (50)
surgery budget dh (50)
inappropriate referrals to ch (50)
HRM (50)
overconfidence dh staff (99)
