# Supplementary figures and images for: Policy options for surgical mentoring: Lessons from Zambia based on stakeholder consultation and systems science
Source: PLoS One. 2021 Sep 29;16(9):e0257597. doi: 10.1371/journal.pone.0257597 (PMC8480833; doi:10.1371/journal.pone.0257597)

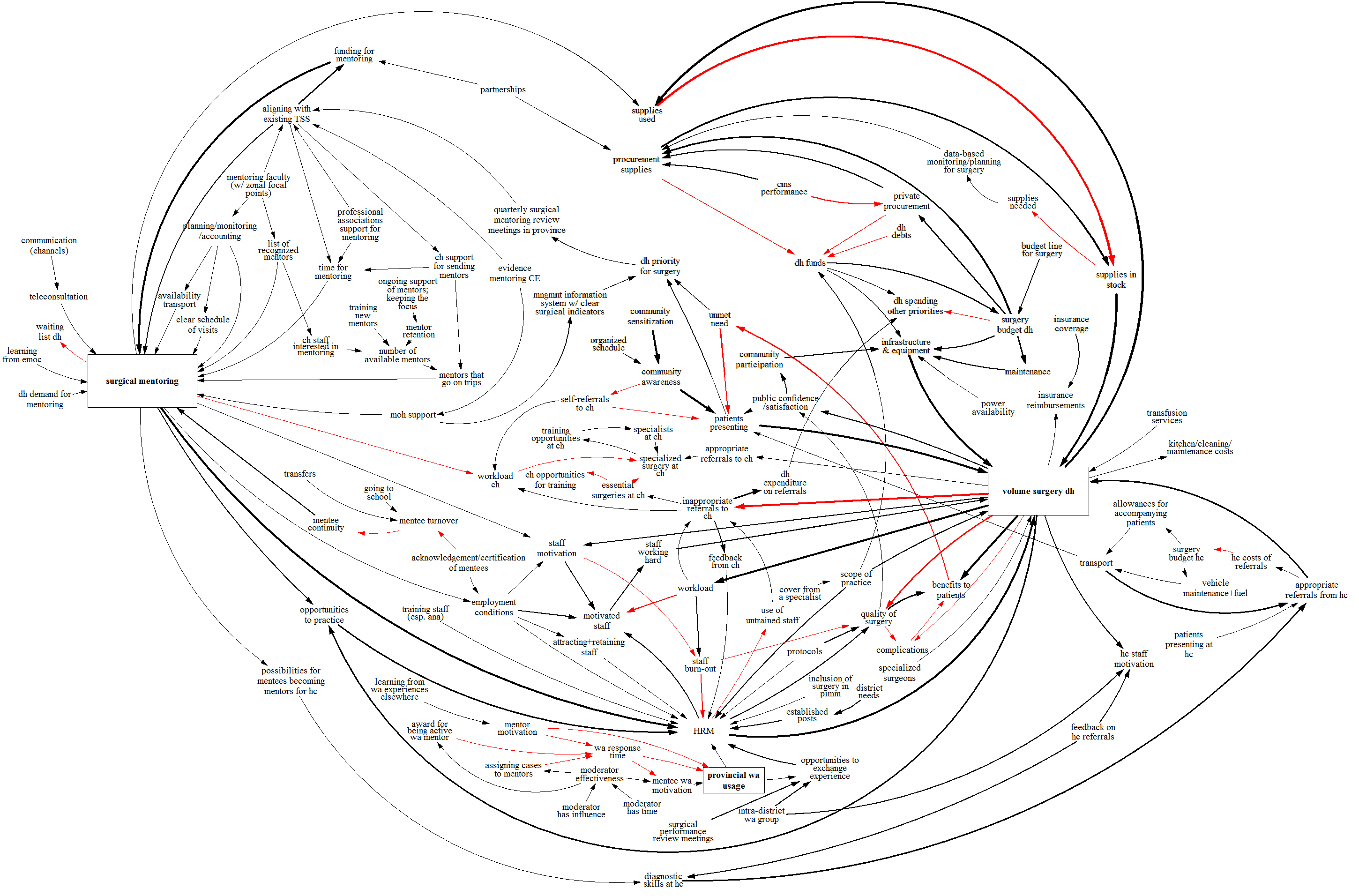

Supplement: S1 Data — (ZIP) [file pone.0257597.s001.zip › Neat joint CLDs/Figure_6.png]

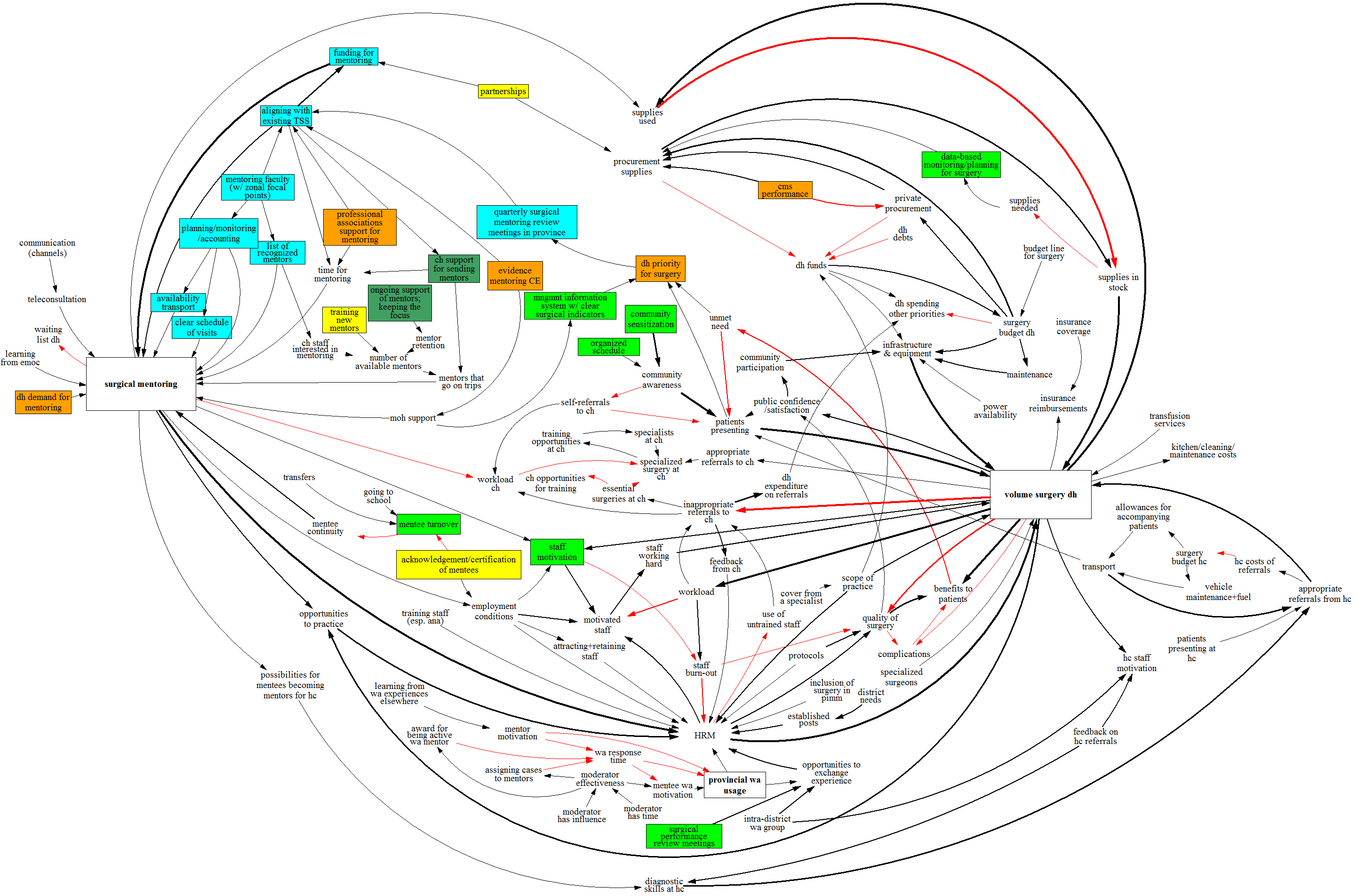

Supplement: S1 Data — (ZIP) [file pone.0257597.s001.zip › Neat joint CLDs/Figure_9.png]

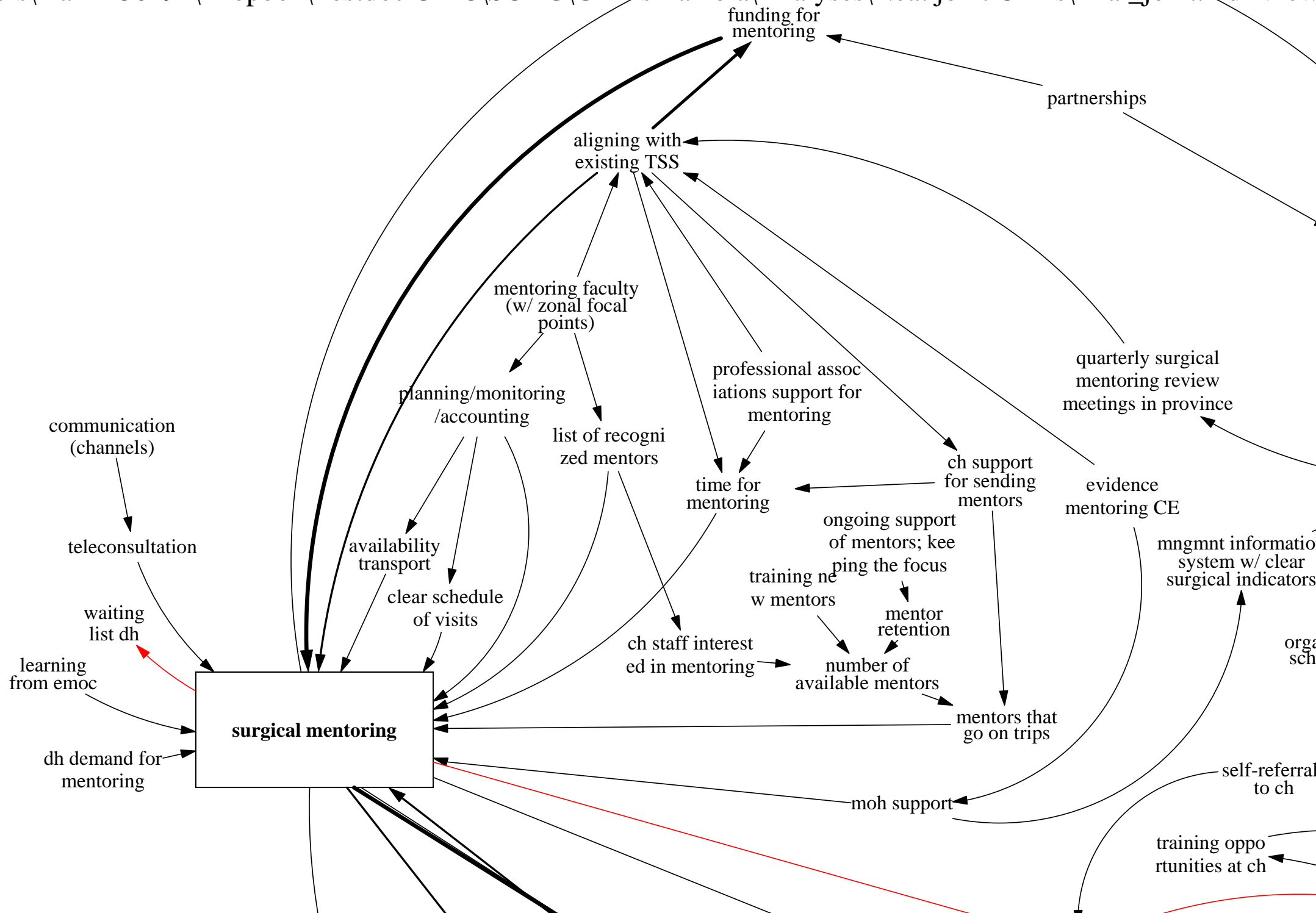

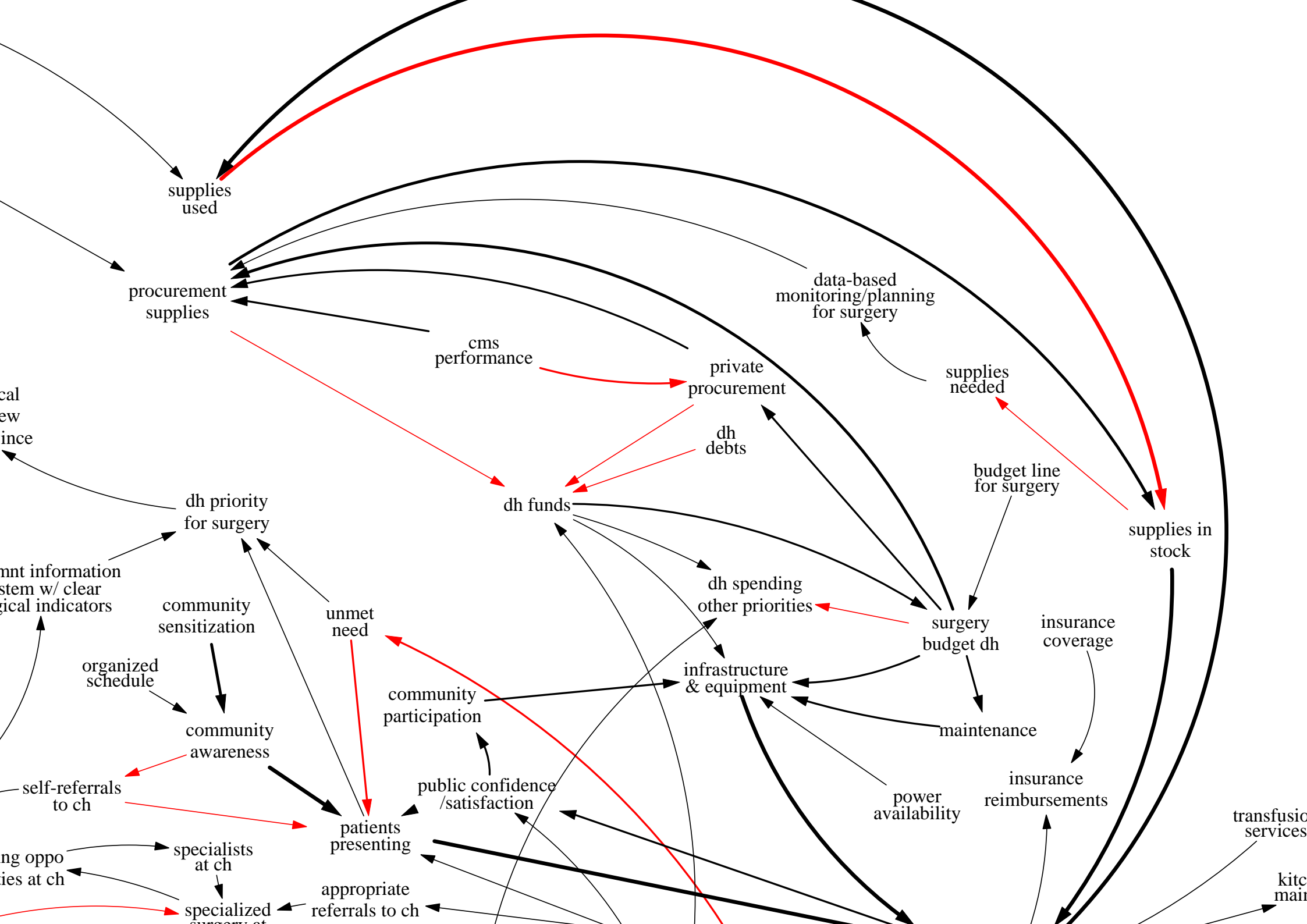

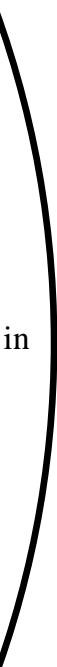

in

transfusion  
services

kitchen/cleaning/  
maintenance costs

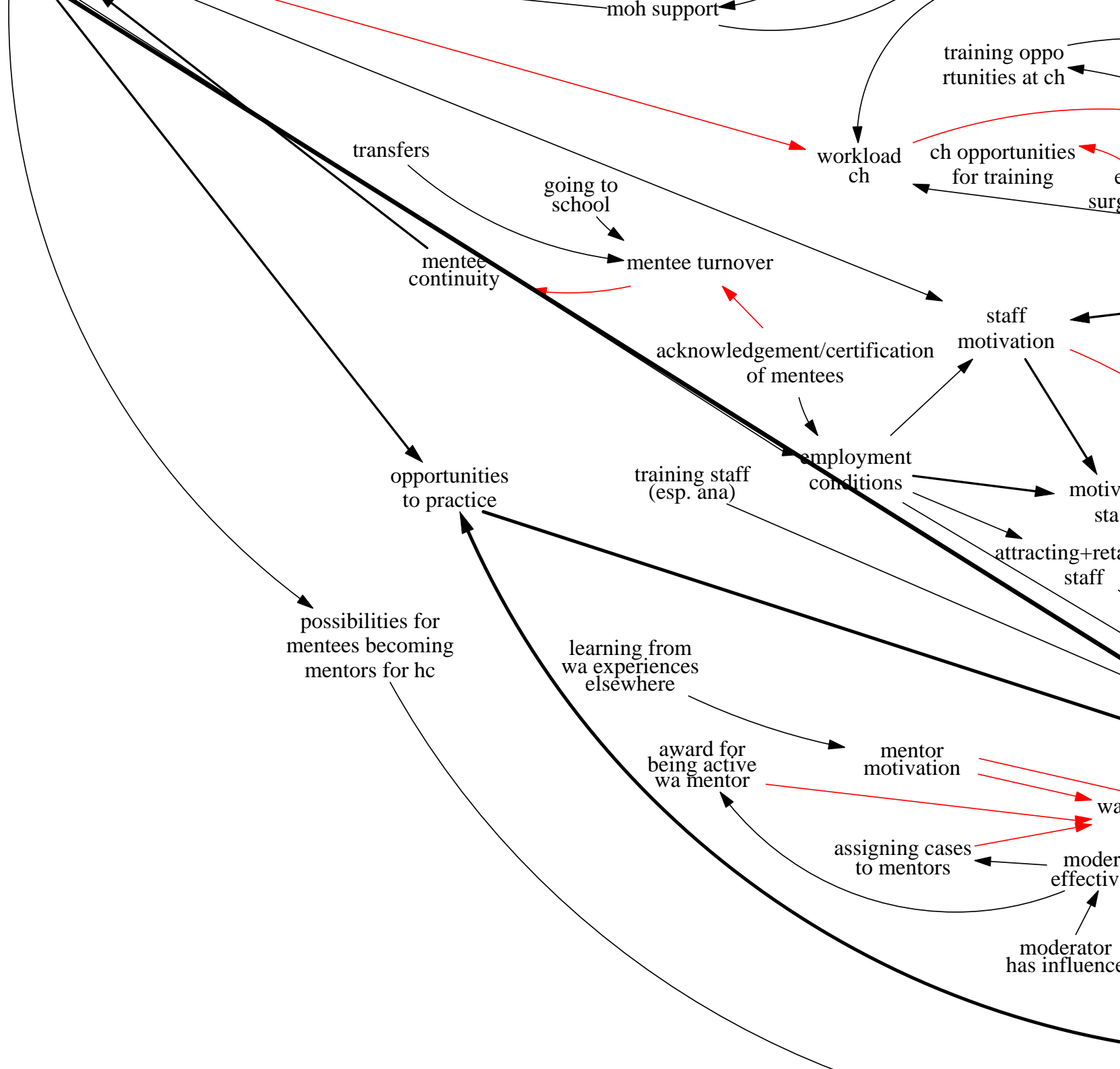

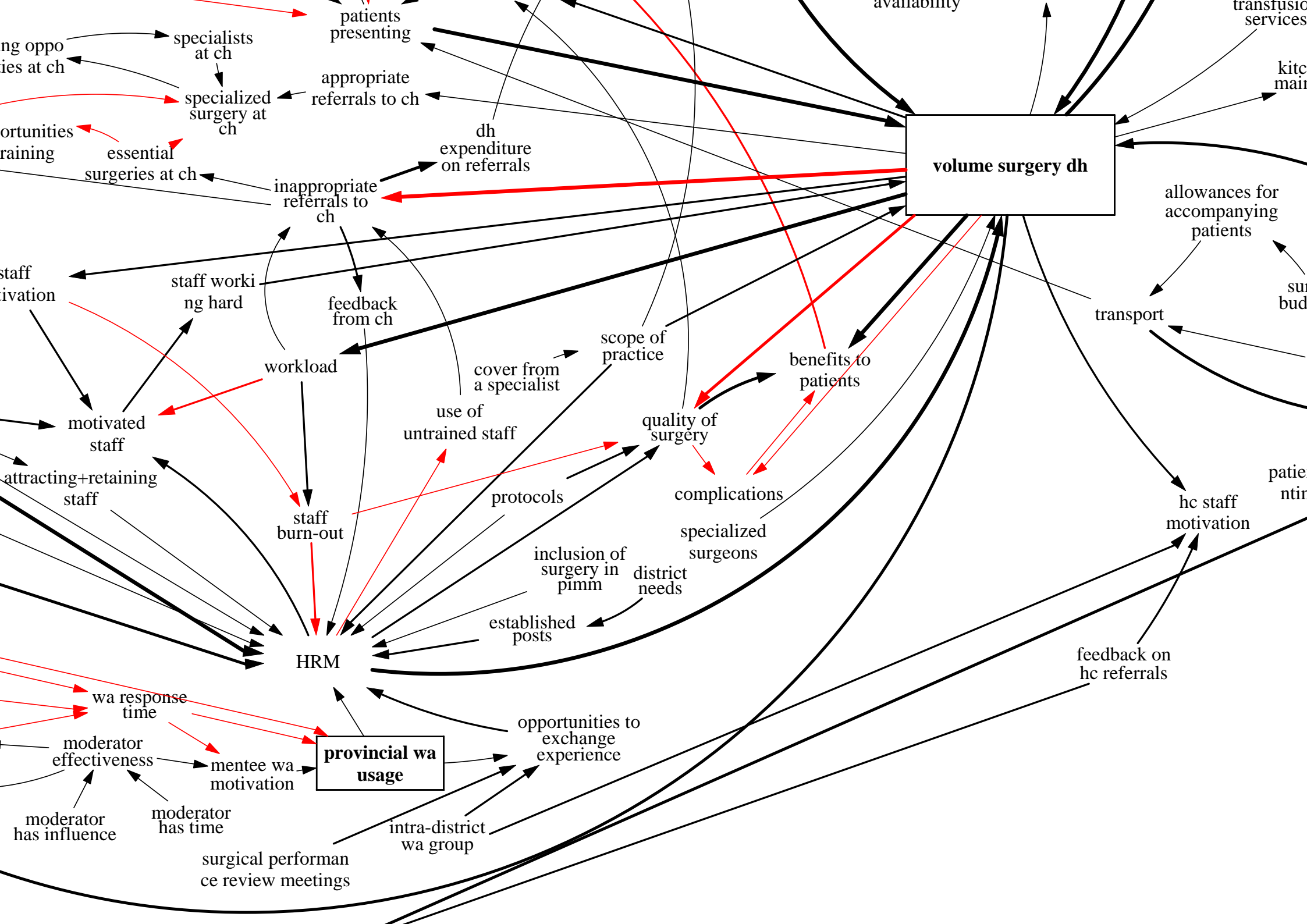

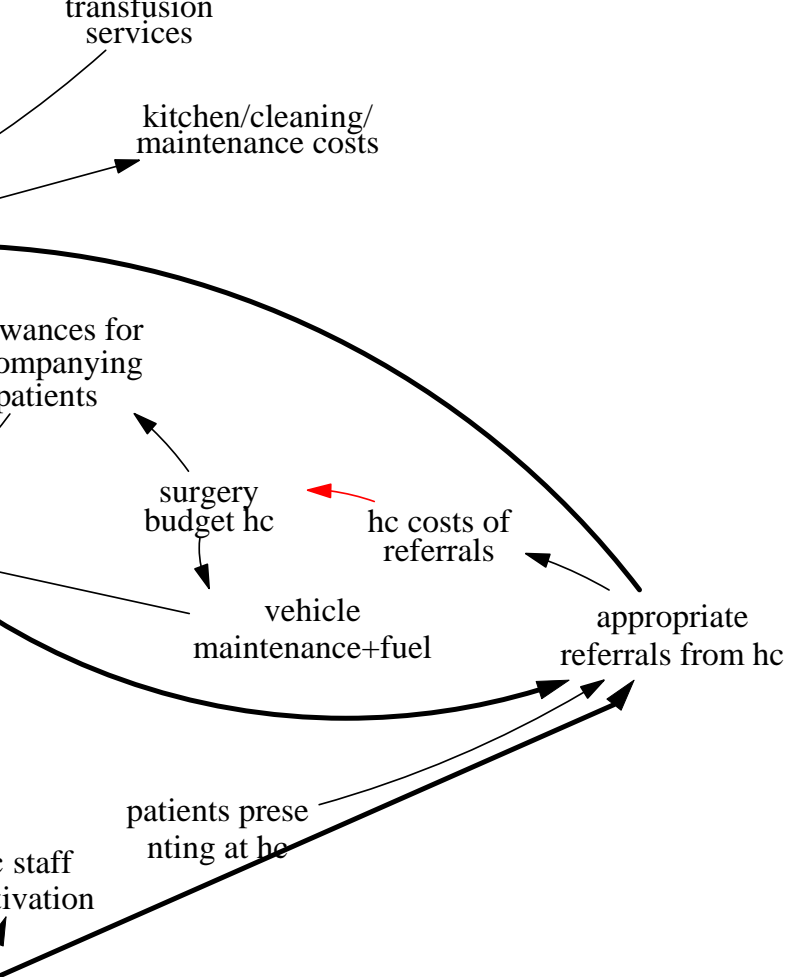

moderator  
has influence

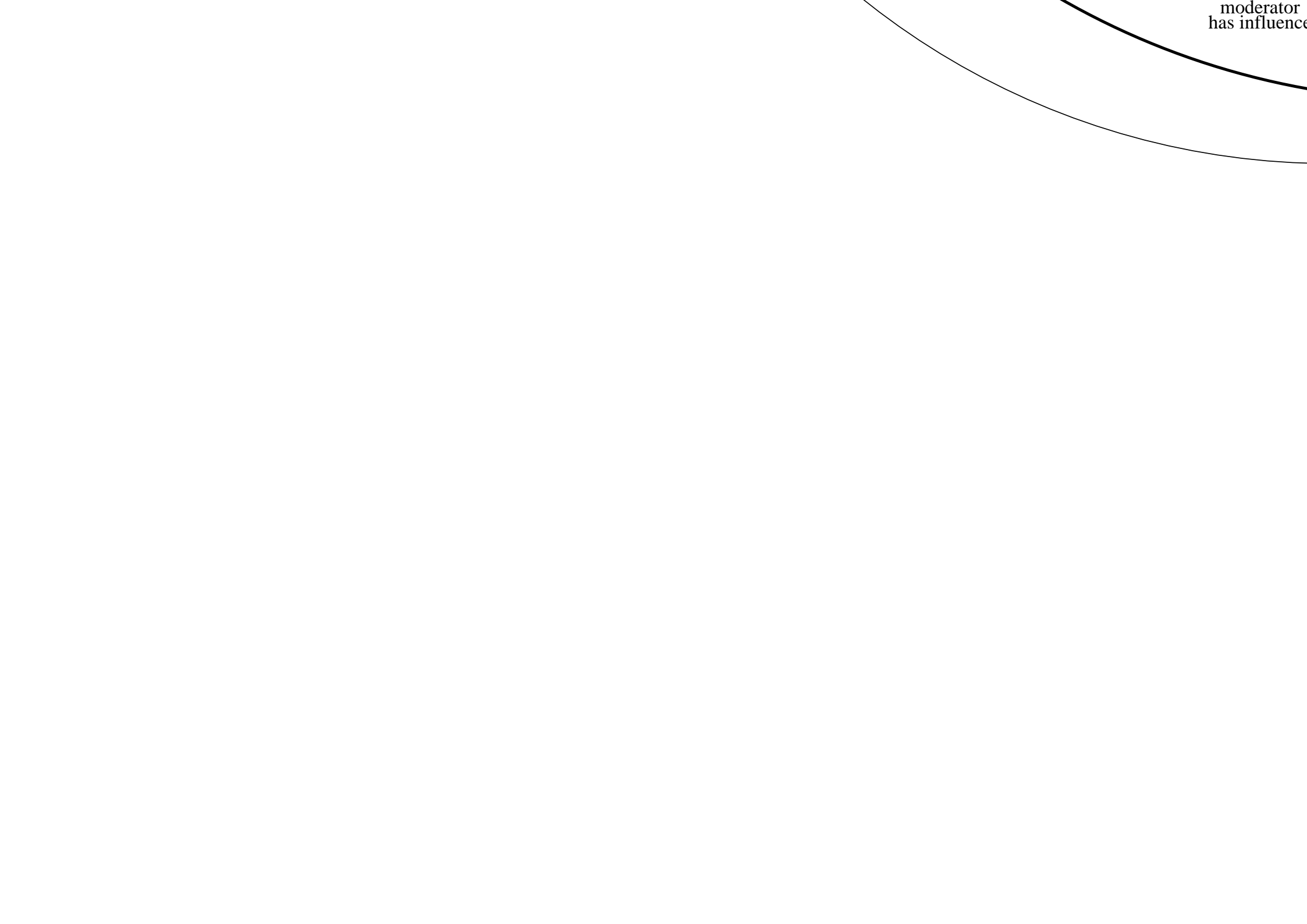

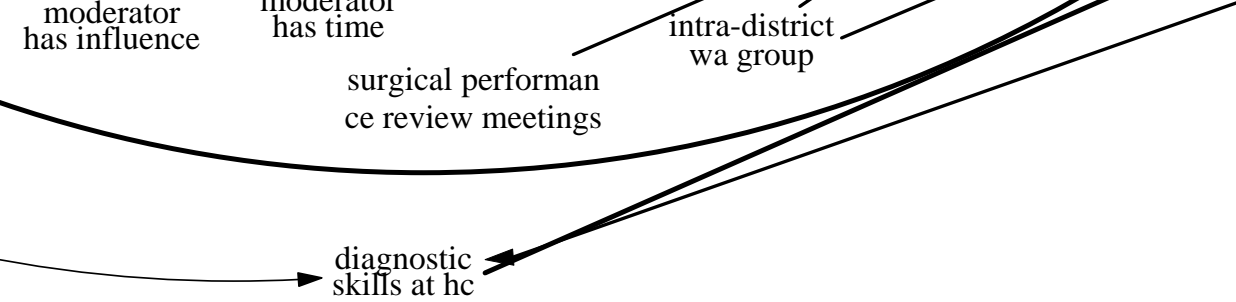

Supplement: S1 Data — (ZIP) [file pone.0257597.s001.zip › Neat joint CLDs/Vensim.pdf]
